# Transcriptional programming in a *Bacteroides* consortium

**DOI:** 10.1038/s41467-022-31614-8

**Published:** 2022-07-06

**Authors:** Brian D. Huang, Thomas M. Groseclose, Corey J. Wilson

**Affiliations:** Georgia Institute of Technology, School of Chemical & Biomolecular Engineering, Atlanta, US

**Keywords:** Synthetic biology, Protein design

## Abstract

*Bacteroides* species are prominent members of the human gut microbiota. The prevalence and stability of *Bacteroides* in humans make them ideal candidates to engineer as programmable living therapeutics. Here we report a biotic decision-making technology in a community of *Bacteroides* (consortium transcriptional programming) with genetic circuit compression. Circuit compression requires systematic pairing of engineered transcription factors with cognate regulatable promoters. In turn, we demonstrate the compression workflow by designing, building, and testing all fundamental two-input logic gates dependent on the inputs isopropyl-β-D-1-thiogalactopyranoside and D-ribose. We then deploy complete sets of logical operations in five human donor *Bacteroides*, with which we demonstrate sequential gain-of-function control in co-culture. Finally, we couple transcriptional programs with CRISPR interference to achieve loss-of-function regulation of endogenous genes—demonstrating complex control over community composition in co-culture. This work provides a powerful toolkit to program gene expression in *Bacteroides* for the development of bespoke therapeutic bacteria.

## Abstract (web summary)

*Bacteroides* species are prominent members of the human gut microbiota. Here the authors develop a platform technology (consortium transcriptional programming) in five human donor Bacteroides chassis cells—enabling complex decision-making within said community, which can be used for the development of living therapeutics.

## Introduction

The human gastrointestinal (GI) tract harbors a microbial ecosystem of enormous complexity that contributes significantly to the health of the host^1^. Evidence continues to emerge connecting the GI microbiota with health and disease states not only in the immediate vicinity of the GI tract^1–3^, but systemically as well^4–6^. Many studies involving the GI microbiota leverage metagenomic data to investigate how its highly variable composition across age and demographics can be connected to health conditions^7–9^. In contrast, several studies have investigated the impact of individual species on the microbiota through functional genomics and targeted manipulation of GI communities^10–14^. As our understanding of the gut microbiota expands in scope and depth, it is conceivable that we will one day be able to engineer intelligent microbial consortia (Supplementary Note 1) that reside in the human body. However, the vast majority of microbes inhabiting the GI tract are obligate anaerobes that are not readily amenable to genetic manipulation. This poses a challenge to synthetic biologists who seek to reprogram these microbes to perform useful functions beyond native capabilities. *Bacteroides* spp. have emerged as promising chassis cells for genetic engineering as a result of knowledge gained over several decades of studies^15^. Their long-term stability in the human colon^8,16^ make *Bacteroides* attractive candidates for engineering as therapeutic bacteria that could modulate their host’s immune system by executing bespoke genetic programs, in addition to facilitating the programmed delivery of therapeutic payloads. While living therapeutics have been developed using bacteria such as *Escherichia coli* (*E. coli*) Nissle 1917^17–19^ and *Lactococcus lactis*^20,21^, these strains are typically cleared from the host within days to weeks^22–24^, limiting their long-term utility. Accordingly, there is an impetus to develop a universal programming structure in *Bacteroides* for use as complex diagnostic tools, living-therapeutics, or for the study of these important contributors to the human microbiota, as *Bacteroides* can function for months to years in situ. Recent efforts have focused on developing genetic regulatory tools specifically for *Bacteroides thetaiotaomicron* (*B. thetaiotaomicron*)^25–28^, as parts developed in *E. coli* tend to be incompatible with the transcription-translation machinery of *Bacteroides*^29,30^. With the intent of engineering select *Bacteroides* as putative chassis cells for further development and study, a small number of inducible promoters regulated by transcription factors have been reported, as well as promoters regulated by dCas9-sgRNA repression^25–27^. Notably, Cello genetic circuit design software was recently implemented in *B. thetaiotaomicron*^27^, demonstrating that higher-order transcriptional logic could be achieved in this chassis cell.

We have recently reported the partial development of an application-agnostic decision-making technology (transcriptional programming) deployed in *E. coli* that leverages systems of engineered transcription factors and accompanying non-natural regulated promoters^31,32^. Here, we report the transference of transcriptional programming and the development of all 16 fundamental logical operations in *B. thetaiotaomicron* in addition to four additional *Bacteroides* species (*B. fragilis*, *B. ovatus*, *B. uniformis*, and *B. vulgatus*)—forming a programmable *Bacteroides* consortium. By combining networks of BUFFER and NOT gates in the form of single transcription factors, we systematically constructed all 16 two-input logic gates regulating a luciferase output—representing a gain-of-function programming structure. Compared to state-of-the-art genetic circuits with similar control features^27,33^, the logic gates reported here are notably compressed in terms of regulated promoters and genetic parts required to build them—while possessing high performance in terms of dynamic range. In addition, we coupled our transcriptional programming system with CRISPR interference (CRISPRi)^34^ to extend our control to both heterologous and endogenous genes—i.e., as a programmable loss-of-function (knockdown) technology. Moreover, we can deploy said transcriptional programming technologies in co-culture to form concurrent, asymmetric, and sequential decision-making within consortia of chassis cells. First, we demonstrated the utility of a set of non-congruent transcriptional programs paired with CRISPRi in a simple consortium to regulate the asymmetric fitness of individual species in co-culture. In turn, we demonstrated that we could achieve sequential asymmetric programming to confer gain-of-function in a separate consortium. The consortium-based transcriptional programming framework presented here will serve as a foundation for next-generation living therapeutics, and provides a powerful technology to advance the general study of the *Bacteroides* genus.

## Results

### Conferring repression and complementary anti-repression in *B. thetaiotaomicron* using engineered transcription factors

**Fig. 1:**
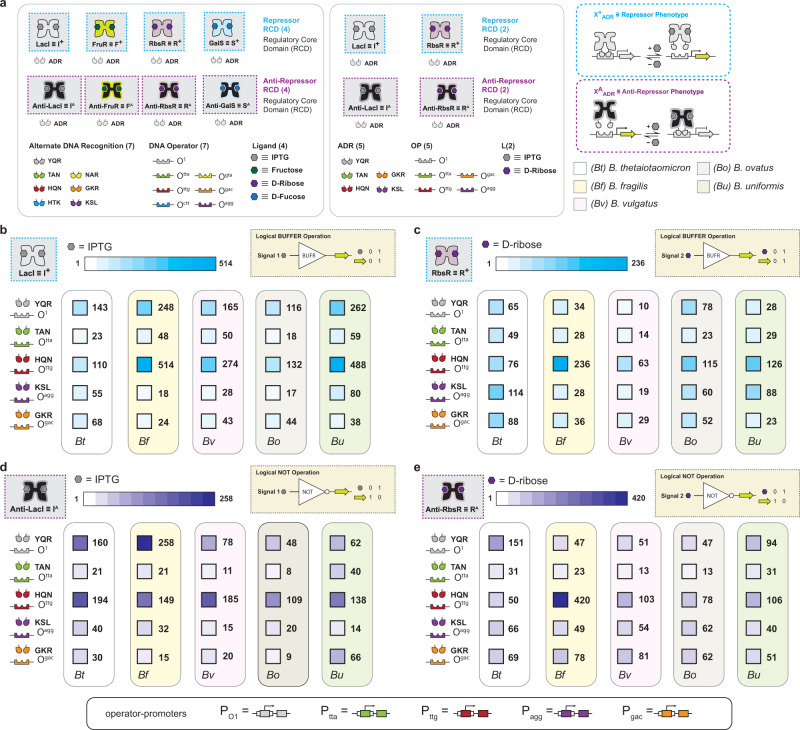
Regulatory performance of transcription factors in *Bacteroides* species. **a** The initial set of transcription factors (TFs) tested in *B. thetaiotaomicron* (left box). The four signal-distinct regulatory core domains (RCDs) are shown with their repressor and anti-repressor cartoons. The cognate ligand for each RCD is shown as a colored hexagon. Each RCD can be paired with one of seven DNA-binding domains (DBDs) that each recognize and bind to a unique cognate DNA operator. Each DBD is abbreviated with a three-letter code where the three letters correspond to the residues located at positions 17, 18, and 22 of the LacI DBD. Cognate DBD-operator interactions are shown as the same color. The reduced set of functional TFs in *B. thetaiotaomicron* is shown in the middle box. The repressor and anti-repressor phenotypes are illustrated in the right boxes. **b** Dynamic range of LacI (I^+^) TFs with alternate DNA recognition (ADR) when paired with cognate operators. Regulated promoters are illustrated at the bottom of the figure. Each box corresponds to the inducible promoter shown in the left columns when deployed in the *Bacteroides* species labeled below (*B. thetaiotaomicron* (*Bt*), *B. fragilis* (*Bf*), *B. vulgatus* (*Bv*), *B. ovatus* (*Bo*), *B. uniformis* (*Bu*)). The dynamic range of each promoter is presented next to its corresponding box which is shaded according to the legends at the top of each panel. Strains harboring inducible promoters were grown in the absence and presence of 10 mM inducer and assayed for luciferase activity (Methods). Dynamic range is presented as the high output state divided by the low output state. **c** Dynamic range of RbsR (R^+^) transcription factors when paired with cognate operators. **d** Dynamic range of anti-LacI (I^A^) transcription factors when paired with cognate operators. **e** Dynamic range of anti-RbsR (R^A^) transcription factors when paired with cognate operators. See Supplementary Figs. 1, 2 for extended data. Source data are provided as a Source Data file. Data represent the average of *n* = 6 biological replicates.

In previous studies, we engineered four sets of signal-distinct repressors^31^ and complementary anti-repressors^32,35^—based on the LacI/GalR topology—that could be directed to seven independent promoters in *E. coli*. (Fig. 1a). This collection of engineered transcription factors (TFs) resulted in 56 single-input logical operations constructed via the systematic pairing of a transcription factor and cognate DNA operator-promoter element—i.e., 28 BUFFER gates and 28 NOT gates. Each repressor and cognate operator-promoter can be regarded as a BUFFER logical operation, whereas each anti-repressor and corresponding genetic element can be regarded as a NOT logical operation. Previous studies demonstrated that the LacI transcription factor (i.e., our structural and mechanistic design template) was functional in *B. thetaiotaomicron*^25,27^. Accordingly, we posited that many (if not all) of the 56 logical operations developed and tested in *E. coli* would be functional in the *B. thetaiotaomicron* chassis cell. Our goal was to identify at least two sets of repressors and complementary anti-repressors to facilitate the full development of transcriptional programming (i.e., via the demonstration of the systematic design, build, and test of all 16 fundamental two-input logical operations) in the *B. thetaiotaomicron* chassis cell. Given that two sets of engineered transcription factors—i.e., LacI (I^+^_ADR_) + anti-LacI (I^A^_ADR_), and RbsR (R^+^_ADR_) + anti-RbsR (R^A^_ADR_)—resulted in significant performance in *E. coli* we focused our search to this best performing subset of logical operations, see Supplementary Fig. 1 and Fig. 1a for a full description of nomenclature and description of the complete set of transcription factors. In brief, 5 out of the 7 alternate DNA-binding recognition (ADR) functions (i.e., ADR = YQR, TAN, HQN, GKR or KSL) for said transcription factors and cognate operator-promoters resulted in >20-fold dynamic range in *E. coli*—i.e., resulting in a putative set of 20 non-synonymous BUFFER gates, and a set of 20 non-synonymous NOT gates targeted for development in the *B. thetaiotaomicron* chassis cell.

Initially, we designed, built, and tested each operation as a standard single-operator promoter system in *B. thetaiotaomicron*. In addition, given that common reporters like green fluorescent protein are not amenable to maturation in anaerobic environments used to culture *B. thetaiotaomicron*^25^, we opted to use NanoLuc® luciferase as the regulated gene output interface. Most (>80%) of the transcription factors displayed inadequate fold-changes as single-operator promoter systems, regardless of the placement of the operator—i.e., whether at the core or proximal position alone (Supplementary Note 2). We surmised that because each DNA operator-promoter was restricted to a single (genome integrated) copy, the apparent affinity for protein-DNA interaction was affected. In turn, we leveraged an in-tandem operator-promoter in which two DNA operators were used, one intercalated between the -33 and -7 hexamer and the other proximal to the transcription start site (TSS) (Fig. 1 inset, Supplementary Fig. 2 and Supplementary Note 2). Using this alternate architecture to direct the cognate transcription factors resulted in the identification of two sets of complementary logical operations with satisfactory performance metrics (i.e., dynamic range > 20). Briefly, in the *B. thetaiotaomicron* chassis cell we identified 5 BUFFER gates responsive to isopropyl-β-D-1-thiogalactopyranoside (IPTG) (Fig. 1b), 5 BUFFER gates responsive to D-ribose (Fig. 1c), 5 NOT gates responsive to IPTG (Fig. 1d), and 5 NOT gates responsive to D-ribose (Fig. 1e). Notably, each set of transcription factors for a given logical operation could be independently directed to five separate cognate operator-promoters—i.e., P_O1_, P_tta_, P_ttg_, P_agg_, or P_gac_—without cross interaction (Fig. 1, Supplementary Fig. 3, and Supplementary Source Data File). In addition, we tested the dose response of I^+^_YQR_, R^+^_YQR_, I^A^_YQR_, and R^A^_YQR_ to verify the ligand concentrations that correlated to the ON-states and OFF-states for repressors and anti-repressors, respectively (Supplementary Fig. 4). Achieving this milestone facilitated the systematic transition from single-input logical operations to two-input (one layer) logical operations.

### Constructing fundamental sets of two-input single-output logical operations in *B. thetaiotaomicron*

**Fig. 2:**
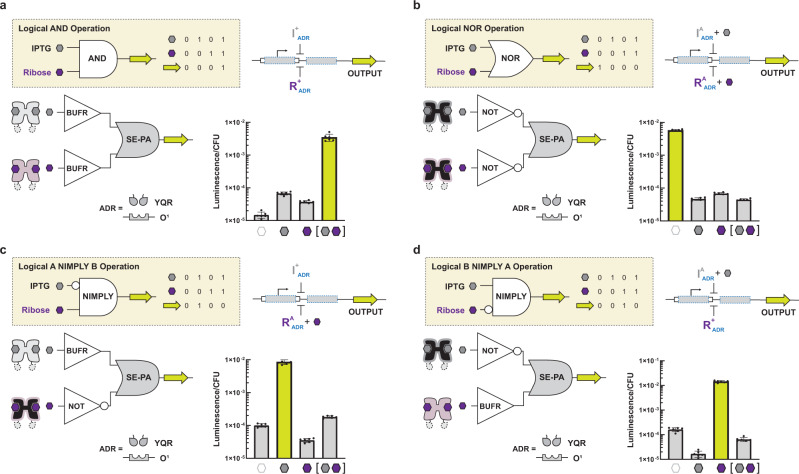
Single-promoter logic gates constructed in *B. thetaiotaomicron*. **a** The AND gate is constructed by directing two repressors to the same operator. We refer to the genetic architecture of directing two or more TFs to a single operator as series-parallel (SE-PA). Bar charts show luciferase activity presented as luminescence per colony forming unit (CFU) (Methods). Each bar corresponds to the ligand condition shown below each bar (empty, no ligand; gray, IPTG; purple, D-ribose; gray and purple, IPTG and D-ribose). Strains harboring logic gates were grown in the presence of all combinations of both inducers (each at a final concentration of 10 mM) and assayed for luciferase activity (Methods). The fold change (presented as the high output state divided by the low output state) is given in Supplementary Fig. 5 for each set of input combinations.  **b** The NOR gate is constructed by directing two anti-repressors to the same operator. **c** The A NIMPLY B gate is constructed by directing an I^+^ and a R^A^ to the same promoter. **d** The B NIMPLY A gate is constructed by directing an I^A^ and a R^+^ to the same promoter. Source data are provided as a Source Data file. Data represent the average of *n* = 6 biological replicates. Error bars correspond to the SEM of these measurements.

An important feature of our system of transcription factors is the ability to systematically pair two non-synonymous transcription factors via one regulated promoter (one layer) to construct fundamental two-input logical operations. In principle, using a single tandem operator-promoter genetic architecture we can construct four simple (one layer) two-input single-output combinational programs—i.e., (i) AND, (ii) NOR, (iii) A NIMPLY B, and (iv) B NIMPLY A. To construct a two-input AND gate in the *B. thetaiotaomicron* chassis cell, we paired two non-synonymous repressors I^+^_YQR_ and R^+^_YQR_ (i.e., two BUFFER gates that were responsive to different input signals) and directed both transcription factors to a single cognate P_O1_ tandem operator-promoter, which regulated a luciferase output (Fig. 2a). The corresponding phenotype objectively resulted in an AND logical operation, where the circuit only allowed the production of luciferase when IPTG and D-ribose were both present. Next, we constructed an antithetical NOR gate using the same P_O1_ tandem operator-promoter (Fig. 2b). However, for the NOR gate we paired I^A(9)^_YQR_ with R^A(1)^_YQR_, directing both anti-repressors (i.e., two non-synonymous NOT operations) to the same DNA regulatory element. The resulting two-input NOR logical operation functioned as expected—where the addition of IPTG or D-ribose resulted in the rejection of the luciferase output.

In addition, we mixed single-input BUFFER and NOT gates directed via the same P_O1_ tandem operator-promoter to form two-input NIMPLY logical operations. Namely, by pairing I^+^_YQR_ with R^A(1)^_YQR_ we generated an A NIMPLY B logical operation (Fig. 2c). Likewise, the B NIMPLY A logical operation was obtained via the complementary set of transcription factors—i.e., I^A(9)^_YQR_ with R^+^_YQR_ (Fig. 2d). To demonstrate that single-layer gate construction was generalizable when directed to different promoter elements, we constructed AND, NOR, A NIMPLY B, and B NIMPLY A via two additional tandem operator-promoters—P_tta_, and P_agg_—which are cognate to the TAN and KSL DNA binding domains of a given transcription factor, respectively, but orthogonal to one another (Supplementary Fig. 5 and Supplementary Source Data File). Qualitatively, all logical operations constructed from the transcription factors with alternate DNA-binding functions resulted in the same objective phenotypes observed for sets directed to the P_O1_ tandem operator-promoter. However, quantitatively the dynamic range was variable, and we posited that this was due to variation in promoter strength, in addition to any differences in the inherent protein-DNA interactions.

### Combinational (feedforward) programming in *B. thetaiotaomicron* and circuit compression

**Fig. 3:**
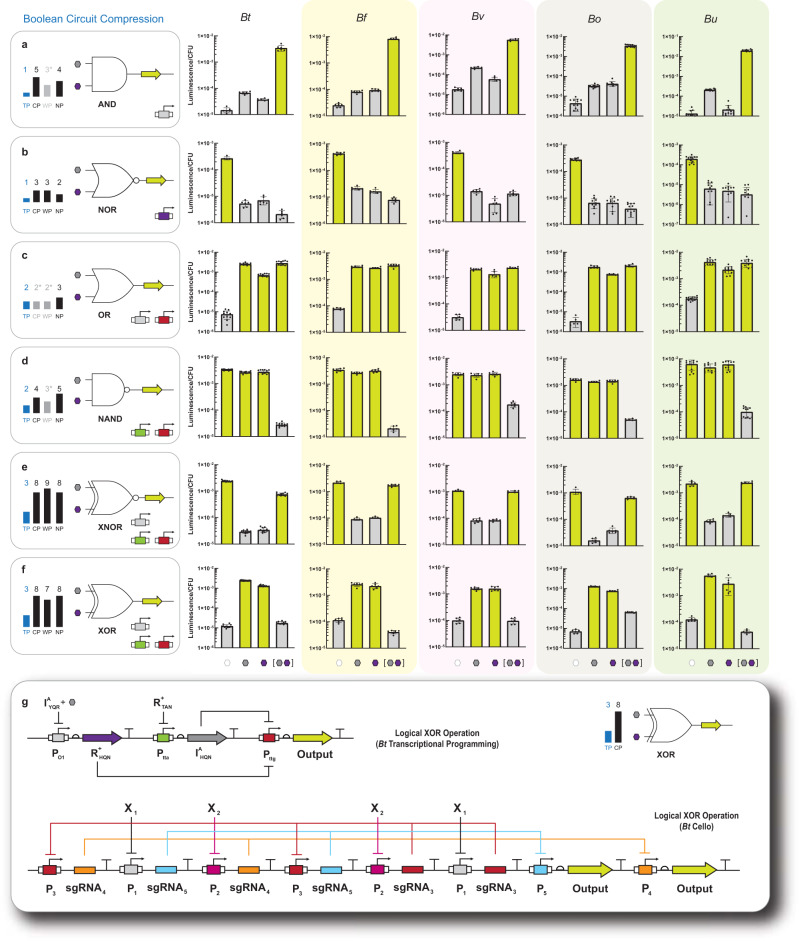
Logic gates and circuit compression in five *Bacteroides* species. **a** Degree of AND gate circuit compression compared to published circuits (left) along with AND gate performance in five *Bacteroides* species (right). Degree of circuit compression is represented by the number of regulated promoters required to construct the logic gate (TP transcriptional programming, CP Cello programming, WP multicellular wires programming, NP Boolean NOR programming). Note, the gray and (^*^) bars in the circuit compression summaries indicate an apparent or ad hoc gate. Strains harboring circuits were grown in the presence of all combinations of both inducers (each at a final concentration of 10 mM) and assayed for luciferase activity (Methods). **b** NOR gate compression and performance in five *Bacteroides*. **c** OR gate compression and performance in five *Bacteroides* (see Supplementary Fig. 8g for corresponding genetic architecture). **d** NAND gate compression and performance in five *Bacteroides* (see Supplementary Fig. 8h for corresponding genetic architecture). **e** XNOR gate compression and performance in five *Bacteroides* (see Supplementary Fig. 8l for corresponding genetic architecture). **f** XOR gate compression and performance in five *Bacteroides*. **g** Wiring diagram for XOR construction using transcriptional programming (top) compared to *Bt* Cello programming (bottom). Regulated promoters used for each logic gate are shown in the bottom right corner of each left-hand box (also see Fig. 1 for more detail). Source data are provided as a Source Data file. Data represent the average of *n* = 6 biological replicates. Error bars correspond to the SEM of these measurements.

In principle, given: (i) 2 non-synonymous repressors, (ii) 2 antithetical anti-repressors, (iii) 3 orthogonal operator-promoters, and (iv) the ability to feedforward information—we can systematically construct all 16 Boolean logic gates via transcriptional programming (Supplementary Fig. 6a). An important feature of this programming structure is that we can simplify (or compress) gate construction via coupled anti-repression—even in the context of feedforward processing—potentially resulting in the reduction of the endogenous resources required for operation. We define gate compression in terms of the number of inducible promoters for a given logical operation, relative to similar logic gates constructed in other biotic systems^27,33^—see Supplementary Note 4. To test these assertions, we designed, built, and tested all remaining two-input gates that required the use of feedforward processing in the *B. thetaiotaomicron* chassis cell—namely, OR, NAND, A IMPLY B, B IMPLY A, XOR, and XNOR (Fig. 3 and Supplementary Fig. 7). The rational design and construction of the remaining feedforward gates was informed by the performances of the individual transcription factors (Supplementary Fig. 2 and Supplementary Note 3). We initiated our design-build-test cycles focusing on the development of simple two-layer feedforward logical operations in which we paired: (i) a single-input single-output logical operation with the output defined as a single non-synonymous transcription factor, with (ii) a second layer that contained a regulatable promoter and luciferase output. Using this general workflow, we constructed OR, NAND, (Fig. 3c, d and Supplementary Fig. 8g, h) and both IMPLY logical operations (Supplementary Fig. 7c, d and Supplementary Fig. 8i, j). Next, we constructed and tested complex feedforward logical operations composed of three layers in which (i) 2 single-input single-output circuits (where the output for each primary layer was defined as a non-synonymous transcription factor) operated in parallel, relative to (ii) a second layer composed of a regulatable promoter (upstream of a luciferase output) with the capacity to direct and couple the transcription factors from the previous layer. This workflow allowed us to construct two of the most complex logic gates from a Boolean perspective—XNOR and XOR (Fig. 3e, f and Supplementary Fig. 8k, l). Qualitatively, all representative two-input feedforward gates resulted in the correct input-output phenotype, with dynamic ranges > 50 (also see Supplementary Source Data File).

To illustrate circuit compression, we conducted a relative comparison of transcriptionally programmed circuits to Cello circuits (the state-of-the-art in gene circuit design)^33^, a chemical wires approach that utilized multiple chassis cells^36^, and general Boolean NOR layering (logical axiom)^37^, (Fig. 3 and Supplementary Fig. 8). The relative comparison was achieved by way of counting the number of regulated promoters used to construct a given circuit—also see Supplementary Note 4. However, in the case of abstract Boolean NOR layering we regarded an input node as a promoter equivalent. In every two-input case, transcriptional programming achieved significant circuit compression over chemical wires and general Boolean NOR layering. While the chemical wires and Boolean approaches provide a broader context for the evaluation of circuit compression, the most meaningful relative comparison was to the Cello genetic circuits—as this technology contains a given circuit to a single chassis cell, akin to transcriptional programming (Fig. 3, Supplementary Fig. 7, and Supplementary Fig. 8). In all cases (with the exception of BUFFER and OR) transcriptional programming resulted in significant circuit compression—i.e., required significantly fewer promoters. In the case of the BUFFER and OR gates, transcriptional programming was on par with Cello—requiring one and two promoters, respectively. Notably, the OR gate is a unique (ad hoc) Cello construct composed of two tandem promoters. In other words, if the OR gate was constructed via inversion alone it would require a minimum of four promoters (Supplementary Fig. 8g and Supplementary Note 5). To illustrate circuit compression, we can use the XNOR and XOR gates as exemplars of the extent of gate compression that can be achieved via transcriptional programming—as these are the two most complex logical operations developed from a NOR programming perspective. In both cases we achieved approximately a three-fold decrease in circuit complexity (promoter requirements) via transcriptional programming—relative to Cello circuit designs. Notably, the XOR logical operation represents the most direct comparison between Cello and transcriptional programming in the same chassis cell—as of this study both gates have been constructed in *B. thetaiotaomicron*. Namely, the Cello XOR gate was composed of eight regulated promoters and two output genes^27^ while the XOR gate constructed via transcriptional programming only required 3 promoters and one output gene (Fig. 3g).

### Transferring transcriptional programming to human donor *Bacteroides* chassis cells

Once we established transcriptional programming in *B. thetaiotaomicron*, we posited that our programming edifice could be extended to other *Bacteroides*. Accordingly, we tested all single-input (BUFFER and NOT) logical operations in four additional *Bacteroides* species that are commonly found in humans—i.e., *B. fragilis*, *B. ovatus*, *B. uniformis*, and *B. vulgatus* (Fig. 1). Namely, we tested 10 non-synonymous BUFFER gates composed of the 5 I^+^ repressors and 5 R^+^ repressors and 5 cognate and orthogonal operator-promoters—i.e., the same set developed in *B. thetaiotaomicron* (Fig. 1b, c and Supplementary Fig. 2). Qualitatively, all 10 BUFFER gates were functional in *B. fragilis*, *B. ovatus*, *B. uniformis*, and *B. vulgatus*. Moreover, the general performance of each BUFFER operation was similar between *Bacteroides* strains and comparable to the performances and trends observed with variation in DNA-binding function in *B. thetaiotaomicron*. Next, we tested each of the antithetical NOT unit operations in *B. fragilis*, *B. ovatus*, *B. uniformis*, and *B. vulgatus*. In this experiment, we tested the individual performances of the corresponding 5 I^A(9)^ and 5 R^A(1)^ anti-repressors using the same 5 cognate operator-promoters (Fig. 1d, e and Supplementary Fig. 2). Congruent with our previous observation, the NOT unit operations had comparable performances to those observed in *B. thetaiotaomicron*.

Given that both single-input logical operations (BUFFER and NOT) functioned in *B. fragilis*, *B. ovatus*, *B. uniformis*, and *B. vulgatus*, we posited that the corresponding single-layer two-input logical operations—(i) AND, (ii) NOR, (iii) A NIMPLY B, and (iv) B NIMPLY A—could be constructed via the same circuit design rules used in the *B. thetaiotaomicron* chassis cell (Supplementary Fig. 8c–f). Accordingly, we transferred the archetypal AND gate (i.e., pairing I^+^_YQR_ and R^+^_YQR_ with the P_O1_ operator-promoter) into each of the four representative *Bacteroides* (Fig. 3a). Constraining the composition of the AND program allowed us to conduct a relative comparison between representative chassis cells to assess to what extent the transcriptional program was impacted by the genetic and metabolic differences between *Bacteroides* strains. Qualitatively, all AND gates resulted in the correct truth table—i.e., all requiring two inputs to induce the expression of luciferase. Quantitatively, all AND gates displayed large dynamic ranges (>200) when comparing zero-input expression to two-input expression (Supplementary Source Data File). The AND gate in *B. uniformis* was the best performing logical operation followed by *B. ovatus*, with dynamic ranges of 1468 and 781, respectively. The AND gates in *B. fragilis* and *B. vulgatus* were nearly on par as logical operations, with dynamic ranges of 325 and 310, respectively (under the same conditions).

Next, we tested an antithetical NOR gate (i.e., pairing I^A(9)^_KSL_ and R^A(1)^_KSL_ with the P_agg_ operator-promoter) in *B. fragilis*, *B. ovatus*, *B. uniformis*, and *B. vulgatus* (Fig. 3b). Each representative NOR gate resulted in the same qualitative outcome—i.e., rejecting the output in the presence of one or both input signal(s) (IPTG and D-ribose). All NOR gates had >50-fold dynamic range for zero inputs (ON-state) relative to both inputs (OFF-state), with the exception of *B. vulgatus* which had a dynamic range of 35—which was consistent with single-input logical operation performances. Congruent with the aforementioned single-layer two-input logical operations, A NIMPLY B (Supplementary Fig. 7a), and B NIMPLY A (Supplementary Fig. 7b) were functional across all representative *Bacteroides* chassis cells.

Once we demonstrated that all fundamental single-layer logical operations were functional in the four representative human donor *Bacteroides*, we tested the remaining two-input feedforward logic gates—i.e., OR, NAND, XNOR, XOR, A IMPLY B, B IMPLY A (Fig. 3c–f and Supplementary Fig. 7c, d). Here we used the same circuit designs outlined in Supplementary Fig. 8g–l. In general, all compressed two-input logic gates functioned as expected in *B. fragilis*, *B. ovatus*, *B. uniformis*, and *B. vulgatus*—and objectively resulted in the correct truth tables. No apparent trends emerged between chassis cells, and the moderate differences in performance between logic gates in a given chassis cell were interpreted as the result of differences in metabolic potential inherent to a given *Bacteroides* strain. Collectively, these results demonstrated that our compressed logic gates could generally be imbued broadly across a panoply of *Bacteroides* chassis cells—representing a robust tool for programmable gain-of-function that can be employed within a consortium.

### Transcriptional programming paired with CRISPR interference in *Bacteroides* chassis cells

**Fig. 4:**
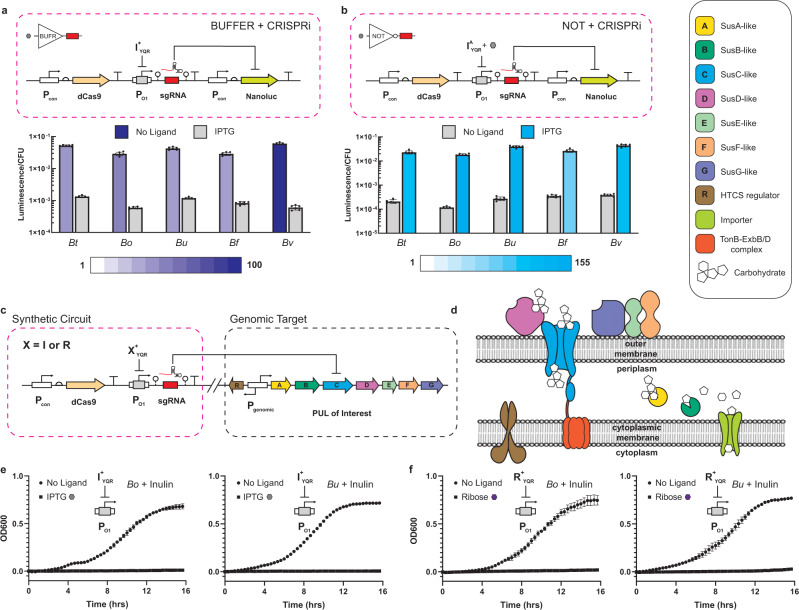
Coupling transcriptional programming with CRISPR interference. **a** Wiring diagram for NanoLuc knockdown based on a BUFFER gate controlling a sgRNA (top). I^+^_YQR_ was used to regulate a sgRNA targeting the NanoLuc gene. Strains harboring the circuit were grown in the absence and presence of 10 mM IPTG and assayed for luciferase activity (bottom) (Methods). Dynamic range is illustrated with shading according to the legend below the graph (purple representing anti-induction, blue representing induction) and is calculated as described previously. **b** Wiring diagram for NanoLuc knockdown based on a NOT gate controlling a sgRNA (top). I^A^_YQR_ was used to regulate a sgRNA targeting the NanoLuc gene. Strains harboring the circuit were assayed as in **a** to determine performance (bottom). **c** Wiring diagram of CRISPRi circuit targeting endogenous SusC-like genes. Strains were created with X^+^_YQR_ (X = I or R) regulating a sgRNA specific to the SusC homolog of interest. **d** Cartoon of PUL organization, highlighting the function of the SusC-like importer. Colors of proteins and complexes correspond to the genes shown in **c** and are listed in the legend at the top right corner. **e** Growth curves of *B. ovatus* (left) and *B. uniformis* (right) harboring circuits shown in **c** with I^+^_YQR_ as the sgRNA regulator. Strains harboring CRISPRi circuits were grown in the absence and presence of 10 mM IPTG in minimal media containing inulin as the only carbon source (Methods). **f** Similar to **e**, but the sgRNA regulator is now R^+^_YQR_. Strains harboring these circuits were grown in the absence and presence of 1 mM D-ribose in inulin minimal media. Source data are provided as a Source Data file. For luciferase assays, data represent the average of *n* = 6 biological replicates. For OD600 growth curves, data represent the average of *n* = 3 biological replicates. Error bars correspond to the SEM of these measurements.

Given the high regulatory performance observed in our logic circuits (both simple and combinational) with inert luciferase outputs, we posited that we could effectively pair transcriptional programming with CRISPR interference (CRISPRi) technology—in each of the representative *Bacteroides* chassis cells. To test this assertion, we first designed, built, and tested iterations of regulated single guide RNA (sgRNA) that targeted the NanoLuc reading frame in *B. thetaiotaomicron* (Fig. 4a). Briefly, we developed a BUFFER gate (I^+^_YQR_ with cognate operator-promoter P_O1_) that regulated the production of the sgRNA transcript aimed at knocking down NanoLuc expression upon induction with IPTG. The P_cfxA_ promoter with tandem core and proximal operators was modified to allow for the production of functional sgRNAs. A hammerhead (HH) ribozyme was fused 4 bp downstream of the proximal operator, followed by the 102 bp sgRNA scaffold^34^ and a hepatitis delta virus (HDV) ribozyme. The inclusion of the ribozymes ensured that the minimal sgRNA was produced following transcription, cleaving extraneous 5' and 3' sequences^38^. For all CRISPRi experiments, dCas9 was constitutively expressed from the P_1_ promoter^27^ and no modification was made to the expression levels of transcription factor regulators. Congruent with our design goal, induction of the BUFFER gate resulted in a ~40-fold reduction of observed NanoLuc production—thus demonstrating that our system is an effective tool for knocking down a given gene in *B. thetaiotaomicron*. We posited that this circuit would be functional in the four additional *Bacteroides* species, given the universal results of our transcriptional programming circuits demonstrated in the aforementioned results (i.e., Figs. 1 and 3). To test this assertion, we introduced the said synthetic circuit into the remaining *Bacteroides* chassis cells and measured the inducible NanoLuc knockdown performances (Fig. 4a). In all cases, each of the engineered chassis cells performed the inducible knockdown of the heterologous luciferase on par or better than that observed in the *B. thetaiotaomicron* chassis cell (also see Supplementary Source Data File).

Next, we constructed the antithetical NOT gate paired with a CRISPRi genetic circuit in the *B. thetaiotaomicron* chassis cell. Here, the basic NOT operation was executed by the I^A(9)^_YQR_ anti-repressor and cognate operator-promoter P_O1_ (Fig. 4b). As anticipated, this simple synthetic circuit resulted in a reciprocal phenotype upon anti-induction via the introduction of the IPTG ligand with a 110-fold dynamic range. Likewise, the integration of this permissive maintenance-of-function circuit (i.e., with ligand) resulted in similar phenotypes in each of the disparate representative *Bacteroides* chassis cells—i.e., *B. fragilis*, *B. ovatus*, *B. uniformis*, and *B. vulgatus* (Fig. 4b).

As evidenced with previous results, the successful implementation of the I^+^_YQR_ (BUFFER gate) and I^A(9)^_YQR_ (NOT gate) with the cognate operator-promoter P_O1_ in a given chassis cell is a strong indicator that the broader transcriptional programming structure can be paired with CRISPR technologies. Here our justification was based on the observation that nearly all remaining single-input and two-input logical operations have similar or better fundamental performances relative to the tested circuits (Supplementary Source Data File). Moreover, given the results of the single-input systems we posited that all additional single-input and two-input logical operations could be used to regulate the production of any sgRNA transcript. Accordingly, we did not test additional iterations of this tool; rather, we demonstrated this assertion via case studies in which we manipulated carbon utilization in *Bacteroides*.

### Controlling carbon utilization in *Bacteroides* via single-input programming

*Bacteroides* possess the ability to degrade a large number of polysaccharides due to specialized gene clusters termed polysaccharide utilization loci (PULs), see Fig. 4d. *Bacteroides* species harbor many PULs, each of which contain genes involved in the recognition, import, and degradation of a specific class of polysaccharide^39–41^. Notably, different species in this genus often possess different catabolic abilities and cannot all utilize the same carbon sources^39,40,42^. It has been previously demonstrated that controlling dietary carbohydrate composition can allow for stable colonization of *Bacteroides* possessing the requisite PUL machinery^11^. This suggests that GI population dynamics may be directly manipulated via a combination of specialized diet and in situ activation of transcriptional programs linked to PUL expression.

To demonstrate the utility of our programming edifice paired with CRISPRi, we posited that we could design sgRNAs to target SusC homologs implicated in the extracellular import of two relevant polysaccharides (inulin and amylopectin) for *B. thetaiotaomicron, B. uniformis*, and *B. ovatus*. The archetypal *susC* gene (starch utilization system gene C) in *B. thetaiotaomicron* is necessary for growth of this species on starch^43^, and its homologs are highly conserved in *Bacteroides* PULs. The rationale for selecting these polysaccharides is that they represent two distinct classes of molecules implicated in GI microbiota homeostasis and are universally consumed by these three *Bacteroides*^44^. Accomplishing this objective would enable control over population dynamics in the presence of a common (communal) carbon source. At the outset, we conducted simple monoculture experiments in which we used a LacI BUFFER operation to regulate the production of a sgRNA targeting SusC homologs in separate *B. thetaiotaomicron, B. uniformis*, and *B. ovatus* chassis cells as monocultures (Fig. 4c, d). In *B. uniformis* and *B. ovatus*, we targeted the SusC homolog implicated in the uptake of inulin, whereas in *B. thetaiotaomicron* we targeted the SusC gene involved in the uptake of amylopectin. Using a simple synthetic circuit composed of an I^+^_YQR_ repressor and cognate regulated promoter P_O1_ (BUFFER gate), all three chassis cells resulted in the strong knockdown of the given PUL upon the addition of IPTG (Fig. 4e and Supplementary Fig. 9a–c). This was observed as a loss of fitness for each monoculture in which a single carbohydrate (i.e., inulin or amylopectin) was present in the defined minimal media. Next, we built and tested an analogous BUFFER CRISPRi synthetic circuit replacing the I^+^_YQR_ regulator with the R^+^_YQR_ repressor targeted at regulating the production of the same sgRNAs. Congruent with the previous synthetic circuit, the introduction of the input signal D-ribose (in the corresponding defined minimal media) resulted in a loss of fitness in all three *Bacteroides* chassis cells in monoculture (Fig. 4f and Supplementary Fig. 9d).

### Controlling carbon utilization in *Bacteroides* via combinational (two-input) programming in monoculture

**Fig. 5:**
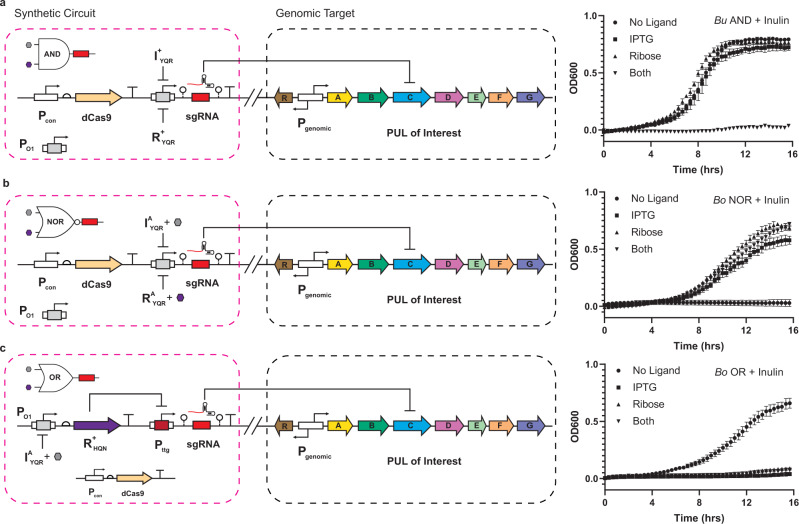
Advanced regulation of endogenous carbon utilization genes. **a** Wiring diagram for an AND gate controlling a sgRNA output targeted to an endogenous gene (left). The sgRNA is the same as in Fig. 4e. Growth curves are shown for *B. uniformis* harboring this circuit when grown in inulin minimal media in the presence of all combinations of both inducers (10 mM IPTG and 1 mM D-ribose) (right). **b** Similar to **a**, but for a NOR gate controlling a sgRNA targeted to the *B. ovatus* SusC-like gene involved in inulin import. **c** Similar to **b**, but for an OR gate controlling the sgRNA output. Source data are provided as a Source Data file. For OD600 growth curves, data represent the average of *n* = 3 biological replicates. Error bars correspond to the SEM of these measurements.

Given the strong performance of the single-input logical operations in managing the knockdown of a select PUL, we posited that we could construct fundamental two-input logic gates to demonstrate more complex decision-making in the context of carbon utilization in select *Bacteroides* chassis cells in monoculture. First, we built and tested a simple AND gate to regulate the uptake and utilization of inulin and amylopectin in *B. uniformis* and *B. thetaiotaomicron*, respectively. In addition, in the *B. ovatus* chassis cell we constructed and tested a NOR gate as well as an OR gate in which we regulated the production of the SusC transporter implicated in the uptake of inulin in monoculture. Congruent with the loss-of-function (fitness) via inverted logic imposed by BUFFER regulated CRISPRi, the AND gates resulted in loss of fitness only when both ligands were present (Fig. 5a and Supplementary Fig. 9e). In contrast, the NOR gate was permissive in the *B. ovatus* chassis cell when one or more ligands were present (Fig. 5b). In turn, the OR gate in the *B. ovatus* chassis cell only resulted in fitness of the monoculture when both ligands were absent (Fig. 5c). This set of experiments demonstrated the application of single-layer (AND, NOR) in addition to feedforward (OR) two-input logic gates as tools that can be used to manage the fitness of individual *Bacteroides* in monoculture.

### Controlling communal carbon utilization in *Bacteroides* via combinational programming in co-culture

**Fig. 6:**
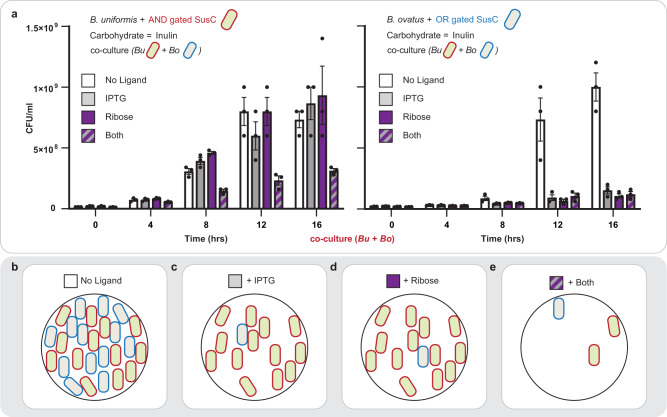
Controlling community composition in co-culture. **a** Demonstration of asymmetric programming of two species in co-culture. The *B. uniformis* strain described in Fig. 5a was co-cultured with the *B. ovatus* strain described in Fig. 5c in inulin minimal media. Each strain was intentionally constructed to harbor a different antibiotic resistance so that they could be differentiated when plated on BHI agar with appropriate antibiotics. The left bar chart shows the density of the *B. uniformis* strain over time as calculated by dilution plating (Methods). The right bar chart shows the density of *B. ovatus* over time. Four co-cultures were grown in parallel, each with a different combination of inducers. White bars represent medium with no ligand, gray bars represent medium with 10 mM IPTG, purple bars represent medium with 1 mM D-ribose, and striped bars represent medium with both inducers. **b** Cartoon illustrating the co-culture composition with no ligand present in the medium. **c** Cartoon illustrating the co-culture composition with IPTG present in the medium. **d** Cartoon illustrating the co-culture composition with D-ribose present in the medium. **e** Cartoon illustrating the co-culture composition with both IPTG and D-ribose present in the medium. Source data are provided as a Source Data file. Data represent the average of *n* = 3 technical replicates (see Supplementary Fig. 9 for additional data and information). Error bars correspond to the SEM of these measurements.

Finally, we constructed a simple consortium composed of *B. uniformis* with an AND gate and *B. ovatus* with an OR gate regulating the production of sgRNAs complementary to the SusC transporters involved in inulin uptake in each chassis cell (Fig. 6). The purpose of this experiment was to demonstrate our ability to asymmetrically program the fitness of a co-culture with a defined communal carbon source. To accomplish this experiment, we implemented a different assay in which we accounted for fitness of each *Bacteroides* species in co-culture via colony forming units (CFU)—given that the batch growth assay in liquid media was not amenable to distinguishing between chassis cells. Each chassis was intentionally constructed with a different antibiotic resistance to allow for their distinguishment when plated on selective BHI agar plates (Methods). We confirmed that the CFU assay for *B. uniformis* and *B. ovatus* in monoculture was congruent with the results obtained in batch growth—accordingly, we can regard the two assays as comparable (Supplementary Fig. 4f and Supplementary Fig. 9f, g). In brief, at 12 h or greater when the co-culture had no ligands present, *B. ovatus* and *B. uniformis* retained similar fitness in co-culture (Fig. 6). Upon the addition of IPTG or D-ribose, only *B. uniformis* retained fitness and could uptake inulin. However, upon the addition of both ligands, *B. ovatus* and *B. uniformis* both experienced loss-of-fitness in the presence of the sole carbon source inulin, as expected.

## Discussion

**Fig. 7:**
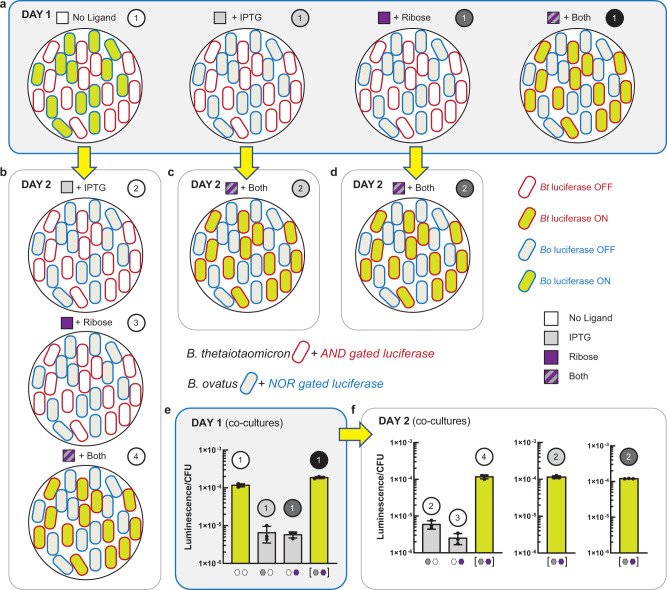
Sequential programming in co-culture. **a** Co-culture composed of *B. thetaiotaomicron* and *B. ovatus* harboring different logic gates. *B. thetaiotaomicron* integrated with an AND gate was grown in the same TYG culture as *B. ovatus* integrated with a NOR gate (both gates regulating luciferase expression). Co-cultures were grown in the presence of all combinations of inducers (each at a final concentration of 10 mM) yielding four total cultures which were independently assayed for luciferase activity (Methods). **b** Sequential programs derived from the “No Ligand” culture in **a**. The initial co-culture was used to seed fresh media containing additional inducers (i.e., IPTG, ribose, or both ligands). These new co-cultures were grown and assayed for luciferase activity (**f**, left bar chart). **c** Sequential program derived from the “+ IPTG” culture in **a**. The initial co-culture was used to seed fresh medium containing both ligands. This new co-culture was subsequently assayed for luciferase activity (**f**, middle bar chart). **d** Sequential program derived from the “+ Ribose” culture in **a**. The initial co-culture was used to seed fresh medium containing both ligands. This new co-culture was subsequently assayed for luciferase activity (**f**, right bar chart). **e** Luciferase activity of co-cultures on day 1. Numbered circles correspond to the co-cultures illustrated in **a**. **f** Luciferase activity of co-cultures on day 2. Numbered circles correspond to the co-cultures illustrated in **b**–**d**. Source data are provided as a Source Data file. Data represent the average of *n* = 3 biological replicates. Error bars correspond to the SEM of these measurements.

In the current study, given the constraint that one out of 16 Boolean logical operations (simple transcriptional programs) can be imbued in a given *Bacteroides* chassis cell, the programming space for two chassis cells in co-culture can be defined by 256 non-synonymous input-output sets (Supplementary Fig. 6). As presented, the input-output consortia sets can facilitate concurrent and sequential (repeated-addition) information processing for gain-of-function for a given *Bacteroides* co-culture. In addition, this programming structure allows the user to construct systems that can imbue symmetric and asymmetric gene regulation between two (or more) chassis cells. To demonstrate sequential programming with asymmetric gene regulation ability, we constructed a simple consortium composed of *B. thetaiotaomicron* and *B. ovatus* imbued with an AND logical operation and a NOR logical operation, respectively. Each logical operation regulated the production of luciferase as a proxy for gain-of-function in a given chassis cell (Fig. 7). Similar to gain-of-function transcriptional programming, we can program loss-of-function between chassis cells, asymmetrically, concurrently, and sequentially, also see Supplementary Note 6.

Consortium transcriptional programming offers a powerful tool that can potentially be used for the advanced study of the gut microbiota. Transcriptional programming can be regarded as universal, and we posit that we can imbue other consortia (beyond the human gut) with complex decision-making capabilities. In addition to our ability to use this platform to study community behavior, we can also use programmable *Bacteroides* communities as the foundation for the development of living therapeutics—which will be the focus of future studies. Notably, the simple sugars allolactose (the natural analog of IPTG) and D-ribose can be consumed and show no evidence of toxicity to *Bacteroides* or host (human) primary cells—in support of progressing this technology to an advanced living therapeutic.

## Methods

### Bacterial strains and media

*Bacteroides* strains used in this study were *B. thetaiotaomicron* (ATCC 29148), *B. fragilis* (ATCC 25285), *B. ovatus* (ATCC 8483), *B. uniformis* (ATCC 8492), and *B. vulgatus* (ATCC 8482). *Bacteroides* strains were routinely cultured anaerobically at 37 °C without shaking using TYG broth or BHI agar (Difco), unless otherwise specified. One liter of TYG broth contains: [10 g tryptone, 5 g yeast extract, 2.5 g D-glucose, 0.5 g L-cysteine, 13.6 g KH_2_PO_4_, 9.2 mg MgSO_4_, 1 g NaHCO_3_, 80 mg NaCl, 8 mg CaCl_2_, 1 mg menadione, 0.218 mg FeSO_4_, 5 μg vitamin B12, and 1 ml histidine hematin solution (1.2 mg/ml hematin in 0.2 M histidine, pH 8.0)]. L-cysteine was resuspended in water and sterile filtered (0.2 μm VWR 28145-477). Menadione was resuspended in 100% ethanol. L-cysteine and menadione were prepared and added to autoclaved media immediately prior to inoculation. Antibiotics for *Bacteroides* were used as appropriate: erythromycin (25 μg/ml), gentamicin (200 μg/ml), and tetracycline (2 μg/ml). IPTG and D-ribose were used as inducers at a final concentration of 10 mM, unless otherwise specified. *E. coli* strains used were EC100D pir-116 (for cloning) and S17-1 ƛ pir (for conjugation). *E. coli* harboring pNBU-based plasmids were routinely cultured aerobically in LB Miller Media at 37 °C with shaking, or on LB agar, supplemented with 100 μg/ml carbenicillin.

### Cloning and plasmid construction

The backbone vectors for pNBU1 and pNBU2 were kind gifts from C. Voigt (MIT). Transcription factors were cloned from in-house vectors while NanoLuc was provided on the pNBU2 vector from C. Voigt. All molecular cloning was performed in *E. coli* EC100D pir-116. Genetic constructs were created using Golden Gate assembly^45^ and Gibson cloning^46^. DNA modules were subcloned into a pUC-based vector for ease of manipulation before performing final assemblies. Q5 polymerase (NEB M0491L) was used for PCR involved in cloning while Phusion polymerase (NEB M0532L) was used for colony PCR. T4 DNA ligase (NEB M0202L) and BsmBI-v2 (R0739L) were used for Golden Gate cloning. NEBuilder HiFi DNA Assembly Master Mix (NEB E2621X) was used for Gibson cloning. All DNA primers were synthesized by Eurofins Genomics. The DNA sequences of all constructs were verified by Sanger sequencing (Eurofins Genomics). Plasmids were visualized using ApE software. Relevant plasmid maps are given in Supplementary Fig. 10.

### Conjugation of *Bacteroides*

*E. coli* S17-1 ƛ pir was used for conjugation of plasmids into *Bacteroides*. The pNBU1 vector harbors intN1 which mediates site-specific recombination of the attN1 site of pNBU1 and the attB1 site located at the 3' end of a tRNA-Leu gene in *Bacteroides* genomes. Similarly, the pNBU2 vector harbors intN2 which mediates site-specific recombination of the attN2 site of pNBU2 and one of two attB2 sites located at the 3' ends of tRNA-Ser genes in *Bacteroides* genomes. Simultaneous insertion of pNBU2 vectors at both sites was never observed, likely due to the necessity of having at least one functional tRNA-Ser gene. Thus, only single copy genetic circuits were stably delivered into *Bacteroides* genomes. Donor cultures of *E. coli* S17-1 ƛ pir transformed with the appropriate pNBU1 or pNBU2 construct and recipient cultures of *Bacteroides* were separately grown to OD600 ~0.5. For all strains except *B. fragilis*, 1 ml of donor culture and 1 ml of recipient culture were pelleted by centrifugation (5000 × *g,* 5 min.) separately and resuspended in 1 ml of PBS. This step was then repeated for a second wash. The cultures were then mixed at a ratio of 1:10 (donor:receiver) and pelleted again by centrifugation. Cells were resuspended in 100 μl PBS and spot plated on a BHI agar plate. The mating lawn was grown aerobically at 37 °C for >16 h before being scraped into 3 ml of PBS. Serial dilutions were plated on BHI agar supplemented with gentamicin and either erythromycin for pNBU2 constructs or tetracycline for pNBU1 constructs. Resultant colonies were picked into TYG after 24–48 h of anaerobic growth. Site-specific integration was confirmed using genome-specific primers. *B. fragilis* conjugation efficiency was significantly lower for unknown reasons. To remedy this, 2 ml of donor culture and 2 ml of recipient culture were combined 1:1 after the PBS wash steps. The remainder of the conjugation procedure was performed as described above.

### Luciferase assay

All luciferase assays were performed using TYG broth. Overnight TYG cultures of *Bacteroides* were diluted 1:100 into 200 μl fresh media in a conical bottom polystyrene 96-well microplate (Nunc 249952) with the appropriate combinations of inducers. The culture was incubated statically in a Mitsubishi rectangular jar equipped with anaerobic gas packs (Mitsubishi R685070) for ~12–14 h to achieve a final OD of ~0.5–0.8. 100 μl of culture was then transferred to a black, clear-bottom 96-well microplate (Corning 3631) to measure OD600. The remaining 100 μl culture was pelleted by centrifugation (4000 × *g* 10 min.) after which the supernatant was removed. The pellet was resuspended in 20 μl of Bugbuster Mastermix (Millipore 71456) and incubated at room temperature for 30 min to facilitate cell lysis. The Promega Nano-Glo assay kit was used to determine expression of NanoLuc. Assay buffer and substrate were mixed as per the manufacturer recommendation (1:50 ratio of substrate to buffer). 10 μl of this mixture was transferred to a well of a flat-bottom white 96-well microplate (Costar 3912) containing 80 μl DI water. Following cell lysis, 10 μl of lysate was added to the microplate well and mixed by pipetting. After 5 min of incubation, the luminescence was measured with a Spectramax M2e plate reader (Molecular Devices) with 800 v gain and 30 reads per well. Data was collected with SoftMax Pro Software. Background luminescence generated from Bugbuster with no cells was subtracted from each sample. Luminescence was then normalized to colony forming units (CFU) based on standard curves relating OD600 to CFU (due to the presence of heme in the growth media, OD600 measurements follow non-linear patterns when compared to CFU). For the CRISPRi luciferase knockdown experiments only, the precultures were grown in the presence and absence of inducer before being seeded into TYG with the same inducer conditions. All other precultures for luciferase assays were grown without inducer. Data was analyzed using Microsoft Excel and Graphpad Prism.

### Orthogonality of DNA-binding domains and operators

To determine potential non-cognate interactions between DNA-binding domains (DBD) and operators, all combinations of DBDs and operators were tested for each transcription factor, yielding a set of 80 “off-diagonal” combinations (in addition to the 20 cognate interactions). To facilitate testing these interactions, five reporter strains of *B. thetaiotaomicron* were created by integrating a pNBU2 plasmid containing the NanoLuc reporter gene fused to 1 of the 5 promoter/operator pairs. These reporter strains were then integrated with pNBU1 vectors containing each of the 16 transcription factors containing the DBD not associated with their specific NanoLuc operator. The expression of NanoLuc with and without inducer was measured as described above.

### CRISPRi growth curves and minimal media co-culture

Long-term anaerobic culture was performed in an anaerobic chamber (Whitley, DG250) with an atmosphere of 10% H_2_, 10% CO_2_, and 80% N_2_ (Airgas X03NI80C2000511). *Bacteroides* strains harboring CRISPRi circuits were first grown overnight in TYG broth (no inducer). The following morning these cultures were diluted 1:100 into fresh TYG with and without inducer(s) and grown until mid-log phase (~6 h). At this point the cultures were diluted 1:200 into defined minimal media (MM) containing the same inducer(s) present in the precultures. One liter of MM contains: [1.12 g (NH_4_)_2_SO_4_, 1 g NaHCO_3_, 13.6 g KH_2_PO_4_, 0.88 g NaCl, 5.55 mg CaCl_2_, 9.5 mg MgCl_2_, 1 mg menadione, 0.218 mg FeSO_4_, 5 μg vitamin B12, 0.5 g L-cysteine, 1 ml histidine hematin solution, and 5 g of defined carbohydrate source]. 10 mg/ml (2X) stocks of amylopectin and inulin were autoclaved and immediately mixed with the MM components (sterile filtered) before being placed in the anaerobic chamber. TYG and MM were pre-reduced in the anaerobic chamber for >24 h before being inoculated. IPTG was added to a final concentration of 10 mM and D-ribose was added to a final concentration of 1 mM when used as inducers. For continuous OD600 measurements, the final MM cultures were prepared in 200 μl volumes in black, clear-bottom 96-well plates. These plates were grown at 37 °C inside a portable spectrophotometer (Cerillo Stratus) placed inside the anaerobic chamber. OD600 was recorded every 20 min to generate growth curves. For co-culture experiments, separate precultures were grown for each species as described above. For these experiments, four precultures of each species were grown in parallel, each containing a different combination of IPTG and D-ribose (no inducer, IPTG only, D-ribose only, and both inducers). Prior to MM inoculation, the OD600 of each preculture was measured. *B. uniformis* and *B. ovatus* were then seeded together into four separate 2 ml MM cultures (containing the four combinations of inducers), with the appropriate precultures being used to seed each MM culture as described above. Based on the preculture OD600 measurements, each species was seeded at an initial density of OD600 ~0.005. The MM co-cultures were gently mixed with pipetting, and a 10 μl aliquot was removed to assess initial population density. Additional 10 μl aliquots were removed every 4 h for 16 h. At the time of removal, each 10 μl aliquot was 10-fold serially diluted in sterile PBS over 7 orders of magnitude. 5 μl of each dilution was spot plated in triplicate on separate BHI agar plates supplemented with erythromycin (to assess *B. uniformis* growth) or tetracycline (to assess *B. ovatus* growth). After 24 h of anaerobic growth, colonies were counted for each time point and species to generate separate growth curves.

### Sequential programming in co-culture

*B. thetaiotaomicron* and *B. ovatus* were precultured separately in TYG with no inducers for 8 h. After measuring the OD600 of each culture, fresh 1 ml cultures containing all combinations of inducers were seeded with both strains such that the initial OD600 of each species was ~0.005. These four cultures were grown for 12 h and then assayed for luciferase activity (Methods). At this time, the inducer-free culture was diluted 1:100 into three separate 1 ml cultures containing either IPTG, D-ribose, or both ligands. The IPTG-containing and D-ribose-containing cultures were similarly diluted 1:100 into new 1 ml cultures containing both ligands. These five new cultures were grown for 12 h and subsequently assayed for luciferase activity.

### Reporting summary

Further information on research design is available in the Nature Research Reporting Summary linked to this article.

## Supplementary information

Supplementary Information

Reporting Summary

## Data Availability

The authors declare that all data supporting the findings of this study are available within the paper and its supplementary information. The analyzed data and source data are available in Supplementary Data Files and Source Data. The sequences of the following plasmids are provided in GenBank and as Source Data with respective accession numbers: pBH001-pBH002 (ON060706-ON060707), pBH101-pBH120 (ON060708-ON060727), pBH201-pBH212 (ON060728-ON060739), pBH301-pBH306 (ON060740-ON060745), pBH501-pBH513 (ON060746-ON060758). Source data are provided with this paper.

## Source data

Source Data Files
