# Supplementary material for: Transcriptional programming in a Bacteroides consortium
Source: Nat Commun. 2022 Jul 6;13:3901. doi: 10.1038/s41467-022-31614-8 (PMC9259675; doi:10.1038/s41467-022-31614-8)
Supplement: Supplementary file 1 — Supplementary Information [file 41467_2022_31614_MOESM1_ESM.pdf]

Supplementary Information

Transcriptional Programming in a *Bacteroides* Consortium

Brian D. Huang[1], Thomas M. Groseclose[1], and Corey J. Wilson[1†]

[1]Georgia Institute of Technology, School of Chemical & Biomolecular Engineering

[†]To whom correspondence should be addressed: Corey J. Wilson, Georgia Institute of Technology, School of Chemical & Biomolecular Engineering, 311 Ferst Drive, Atlanta, GA 30332-0100. Telephone: 404-385-5397; E-Mail: corey.wilson@chbe.gatech.edu

TABLE OF CONTENTS

Supplementary Note 1

Supplementary Note 2

Supplementary Note 3

Supplementary Note 4

Supplementary Note 5

Supplementary Note 6

Figure S1: Components of modular transcription factors used for transcriptional programming

Figure S2: Regulatory performance of transcription factors in *Bacteroides* species

Figure S3: Demonstration of orthogonality between DNA-binding domains and synthetic operators

Figure S4: Transcription factor sensitivity and OD-CFU comparison

Figure S5: Additional single-promoter logic gates constructed in *B. thetaiotaomicron*

Figure S6: A complete set of 16 logic gates and consortium transcriptional programming charts

Figure S7: Circuit compression of NIMPLY and IMPLY gates

Figure S8: Direct comparison of compressed circuits with Cello programming

Figure S9: Demonstration of SusC knockdown in *B. thetaiotaomicron*

Figure S10: Maps of plasmids constructed in this study

Table S1: Plasmids constructed in this work

Table S2: Genetic parts used in this work

Supplemental References

Supplementary Note 1: Intelligent biotic system – definition. We define an intelligent biotic system as one or more chassis cells capable of (i) decision-making, (ii) coupled memory development, (iii) and communication between chassis cells and/or the host.

Supplementary Note 2: Low performing transcription factors in *B. thetaiotaomicron* – Justification and alternate design. Most of the transcription factors displayed inadequate fold-changes when regulating promoters with an operator at the core or proximal position alone. We generally observed weak repression evidenced by high basal expression levels when the transcription factor was bound to DNA. We posited that we could improve the performance of a given logical operation *via* increasing the apparent affinity of the transcription factor by doubling the number of DNA binding sites by way of tandem operators. The general design of the in-tandem operator-promoter was composed of two DNA operators, one intercalated between the -33 and -7 hexamer and the other proximal to the TSS (see **Supplementary Figs. 1-2**). In principle, this design maintains the ability to concurrently direct the binding of one or more cognate transcription factors while preserving orthogonal DNA binding. NOTE: The general form of this genetic architecture has been designated as a series-parallel (SE-PA) operator function in our previous reports[1,2].

Supplemental Note 3: Transcriptional programming and construction of feedforward gates. The development of a complete set of 16 logical operations *via* transcriptional programming is predicated on a definitive bottom-up combinational rule set. Specifically, single-input single-output operations (BUFFER and NOT) represent the fundamental binaries, that can be systematically combined to create all proper two-input single-output operations (AND, NOR, A NIMPLY B, B NIMPLY A, OR, NAND, A IMPLY B, B IMPLY A, XOR, and XNOR). Rational construction of feedforward gates was informed by the performances of the individual transcription factors (see **Supplementary Fig. 2**). The P_{O1} and P_{tta} promoters were chosen for the first layer due to their higher maximum output when compared to the other inducible promoters developed. This was done to ensure a saturating amount of transcription factor would be produced to act on the final output promoter. The P_{ttg} promoter was chosen as the final output promoter given its high performance (minimal "leaky" expression) when controlled by any of the four X_{HQN} transcription factors ($X = I^+$, I^A, R^+ or R^A).

Supplemental Note 4: Circuit compression and factors beyond the inducible promoters. We define circuit compression as a reduction in the number of inducible promoters between any two genetic circuits with comparable operation or function. We note that other factor such as the number of constitutive promoters that are required to operate the circuit are equivalent (or fewer) between said genetic circuits. The Cello circuits discussed in this study are constructed *via* in inversion[3] which will utilize equivalent numbers of constitutive promoters relative to transcriptional programming, or by way of the concurrent expression of dCas9[4], which will utilize an additional constitutive promoter relative to transcriptional programming (T-Pro). Accordingly, given that the number of constitutive promoters used in transcriptional programming will be equal or less than synonymous Cello circuits, the number of constitutive promoters was not factored in to the accounting for compression – although in such cases where this becomes significant constitutive promoters can be included.

We posit that given two synonymous circuits (*e.g.*, XOR – Cello vs. XOR – T-Pro, see **Fig. 3g**) the compressed circuit in which promoter strengths and RBS strengths on average (*i.e.*, translation and transcription) are *on par* between inducible promoters, the compressed circuit will utilize fewer cellular resources. To test this assertion would require an assessment of growth rates, ribosome profiling, and RNA-seq analysis – in addition to using approximately equivalent production machinery, *i.e.*, promoter strength and RBS strength, and perhaps normalizing protein lifetimes – which is beyond the scope of the current study.

Supplemental Note 5: Cello Gates. Cello circuit design[3] leverages tandem promoters to create OR and NOR gates that can be connected in a modular fashion. The OR gate was developed by placing two distinct, inducible promoters upstream of a sequence of interest such that induction of either or both promoters resulted in production of the downstream target[5] (see **Supplementary Fig. 8g**). The NOR gate was achieved

by inverting the OR gate and adding a second regulated output promoter (see **Supplementary Fig. 8d**). Specifically, the output of the OR gate is a repressor that acts on a second regulated promoter, controlling production of the final output. The tandem promoter setup allows for the construction of a 2-promoter OR gate rather than a 4-promoter OR gate which would be achieved using a pure layering approach (see **Supplementary Fig. 8g**). Additionally, the resulting NOR gate uses the same number of promoters (3) as would be required from a layering approach (see **Supplementary Fig. 8d**). The use of tandem promoters results in several technical challenges – evident from the general architecture. Namely, unequal output levels may be observed for an OR gate if the tandem promoters affect one another's activity[5]. Nielsen and coworkers describe one such phenomenon as "roadblocking", where a downstream promoter prevents the upstream promoter from transcribing the target sequence[3]. Accordingly, roadblocking limits the number of regulated promoters that can be used in a tandem fashion, adding an additional constraint to Cello-designed circuits.

Supplemental Note 6: Dynamics of repeated addition and concentration dependence. As demonstrated in this study, biological signal processing can be achieved by way of allosteric transcription factors (native and engineered). For example, in regulatory systems that utilize the lactose repressor, an input signal results in the induction of the transcription factor and objectively switches gene expression from an OFF-state to an ON-state. In the given biological system to revert the gene expression back to the OFF-state requires the aggressive dilution of the input signal which can take one or more days to achieve in a typical biotic system. Kinetic studies using our engineered BANDPASS and BANDSTOP transcription factors have shown that our collection of signal processing filters can switch between states of gene expression within a few minutes (opposed to days)[6]. We posit that given that I^{+}_{YQR}, R^{+}_{YQR}, I^{A}_{YQR}, and R^{A}_{YQR} are predicated on the same topology and basic functional mechanism our repeated addition programs will have similar dynamic features. In addition, the maintenance of an induced ON-state or OFF-state will require ligand concentrations of ~1mM or higher. Noting that said features will be important in subsequent implementation of this methodology. Given that our collection of transcription factors are only inducible at higher ligand concentrations than would be observed in native environments, the unintended activation of said genetic circuits is mitigated, see **Supplementary Fig. 4**. In addition, we anticipate that our transcriptional programs that involve repeated addition have the capacity to rapidly transition between states based on our observations of the dynamics of systems with similar mechanistic features[6].

Supplementary Fig. 1

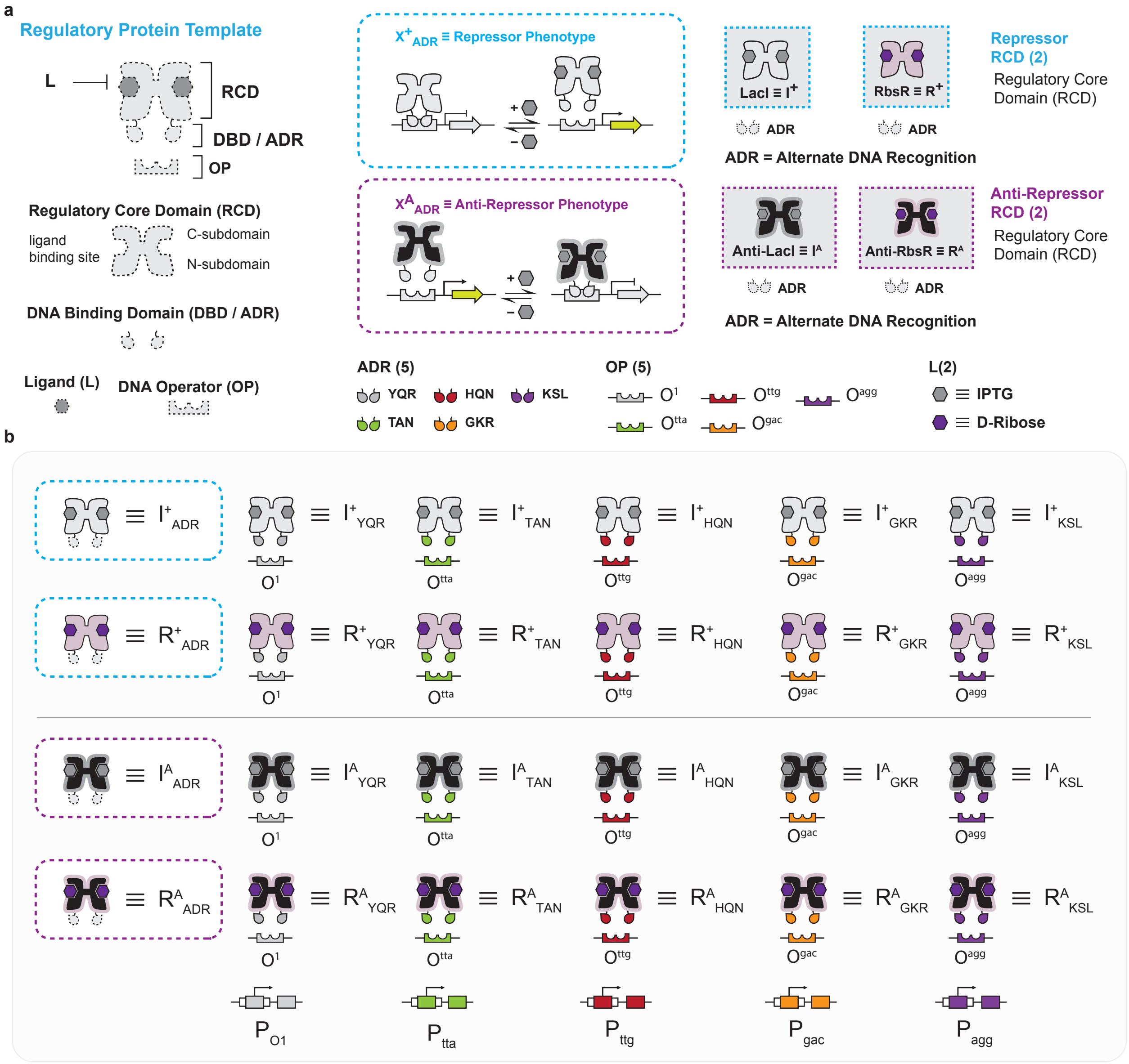

SI Fig. 1 | Components of modular transcription factors used for transcriptional programming. a, Summary of transcription factor architecture and nomenclature used in this study. Each transcription factor is composed of a regulatory core domain (RCD) that binds to a unique small molecule ligand and a DNA-binding domain (DBD) that binds to a specific operator (op). The cognate ligand for each RCD is shown as a colored hexagon. The repressor and anti-repressor phenotypes are illustrated in the middle. Transcription factors utilize alternate DNA recognition (ADR) which is comprised of 5 DBDs that recognize 5 unique operators which are color-coded. Each DBD is abbreviated with a three-letter code where the three letters correspond to the residues located at positions 17, 18, and 22 of the LacI DBD. Each operator is abbreviated with a three-letter code that corresponds to the critically-recognized bases of the synthetic DNA sequence. **b,** The full panel of 20 transcription factors used in this study. All 20 transcription factors are illustrated with corresponding abbreviations used in this study.

Supplementary Fig. 2

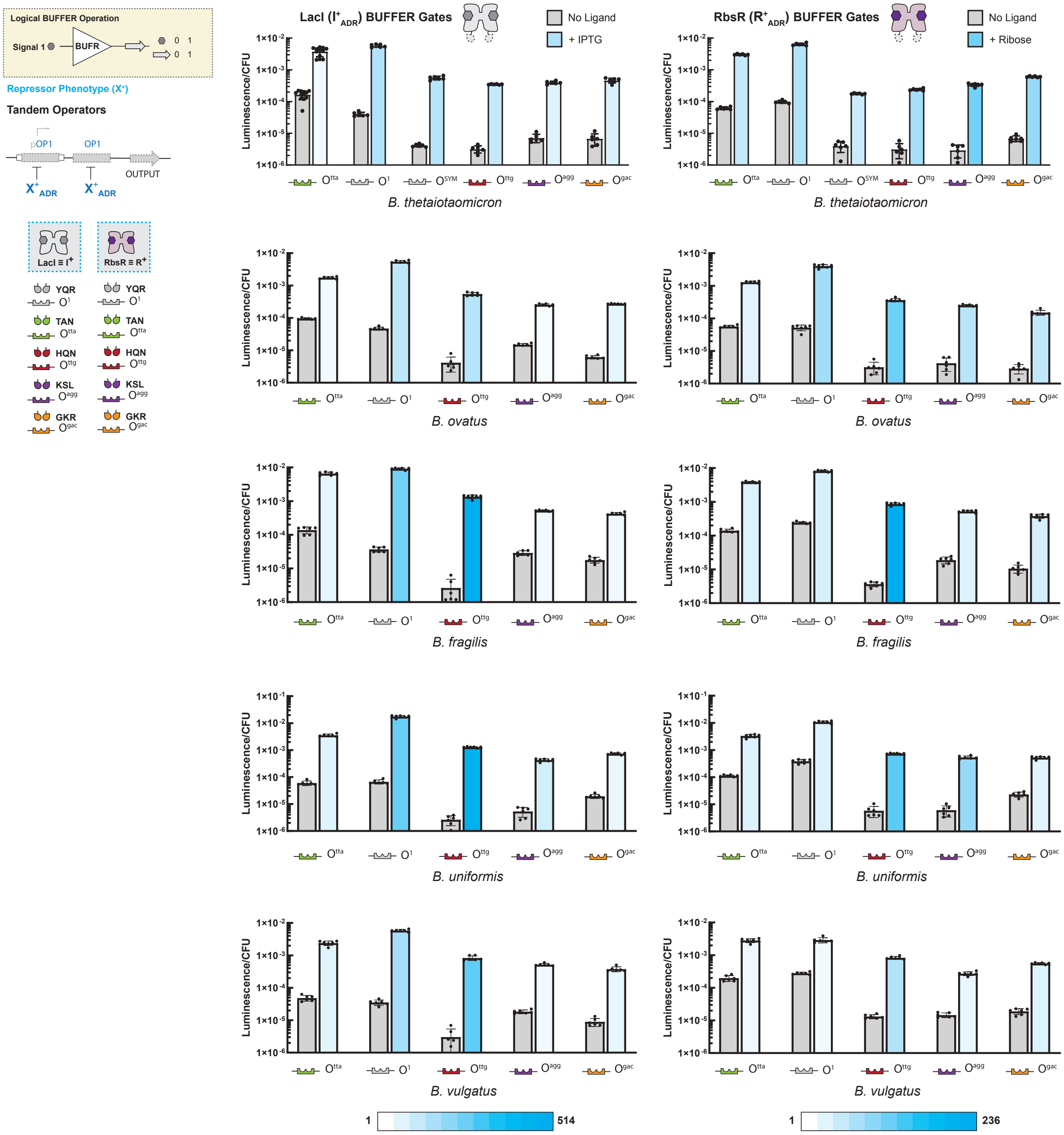

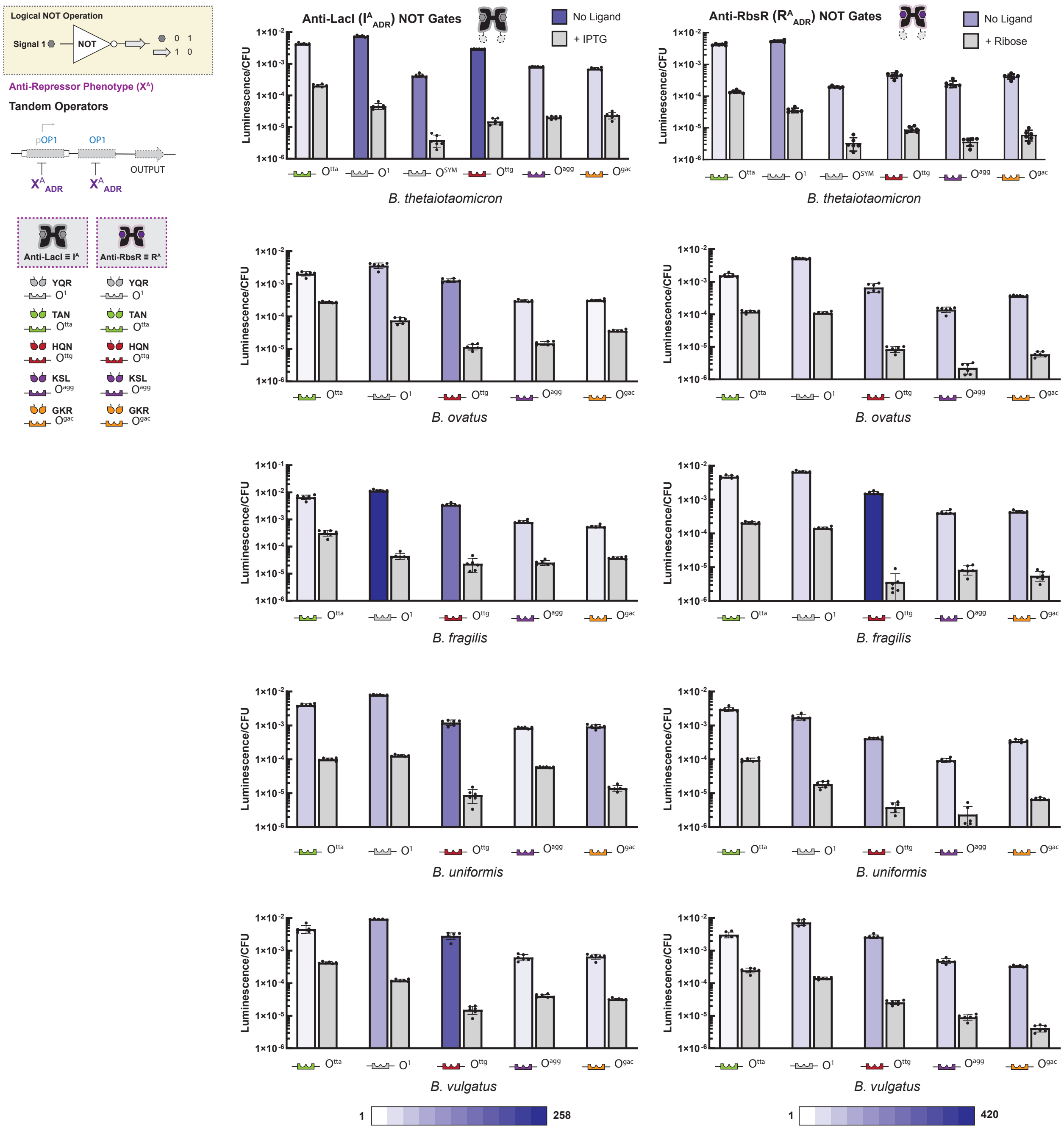

SI Fig. 2 | Regulatory performance of transcription factors in *Bacteroides* species. Extended data related to **Fig. 1**. Low and high states for every cognate TF-promoter pair are shown for the five *Bacteroides* species. Bar pairs correspond to the squares in **Fig. 1** with the dynamic range being the ratio of high and low states. To direct each transcription factor, we used in-tandem operator-promoters composed of two DNA operators, one intercalated between the -33 and -7 hexamer and the other proximal to the TSS, also see **Supplementary Note 2**. Each set of transcription factors for a given logical operation could be independently directed to five separate cognate operator-promoters – i.e., P_{O1}, P_{tta}, P_{ttg}, P_{agg}, or P_{gac} – without cross interaction (also see **Supplementary Fig. 3**). Data represent the average of $n = 6$ biological replicates. Error bars correspond to the SEM of these measurements. Induction of each promoter was determined to be statistically significant ($P < 0.001$) using Welch's two-tailed unequal variances *t*-test. Exact P-values can be found in the Source Data file.

Supplementary Fig. 3

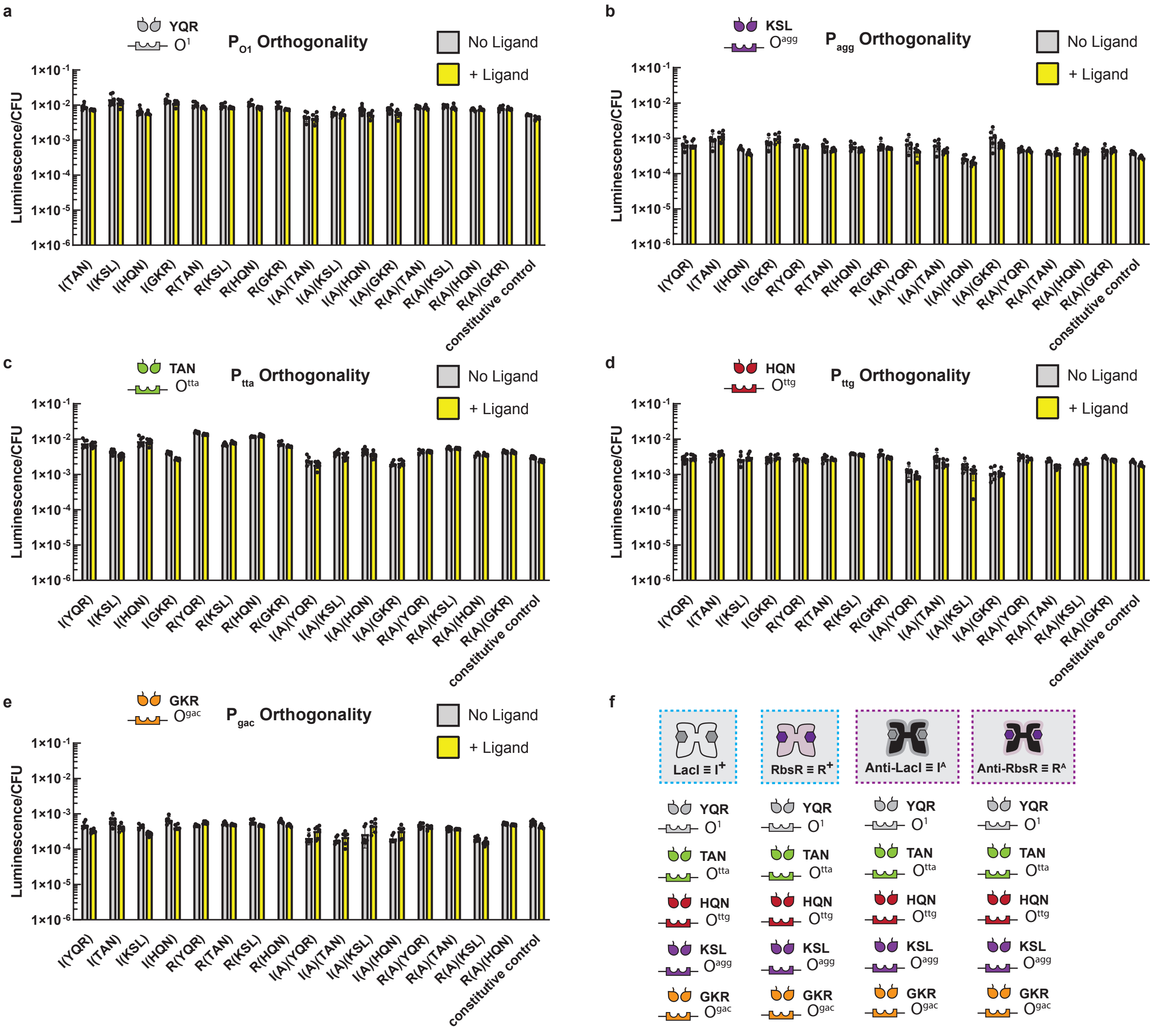

SI Fig. 3 | Demonstration of orthogonality between DNA-binding domains and synthetic operators. **a,** P_{O1} orthogonality test. The 16 non-cognate TFs were tested for their ability to regulate P_{O1}. Separate strains were created harboring the inducible promoter and each of the 16 non-cognate TFs. These strains were grown in the absence and presence of inducer and assayed for luciferase activity (**Methods**). The bars on the far right correspond to a strain harboring the promoter but no TF, serving as a constitutive control. **b,** P_{agg} orthogonality test. **c,** P_{tta} orthogonality test. **d,** P_{ttg} orthogonality test. **e,** P_{gac} orthogonality test. **f,** illustration of the 20 transcription factors used in this study. Data represent the average of n = 6 biological replicates. Error bars correspond to the SEM of these measurements.

Supplementary Fig. 4

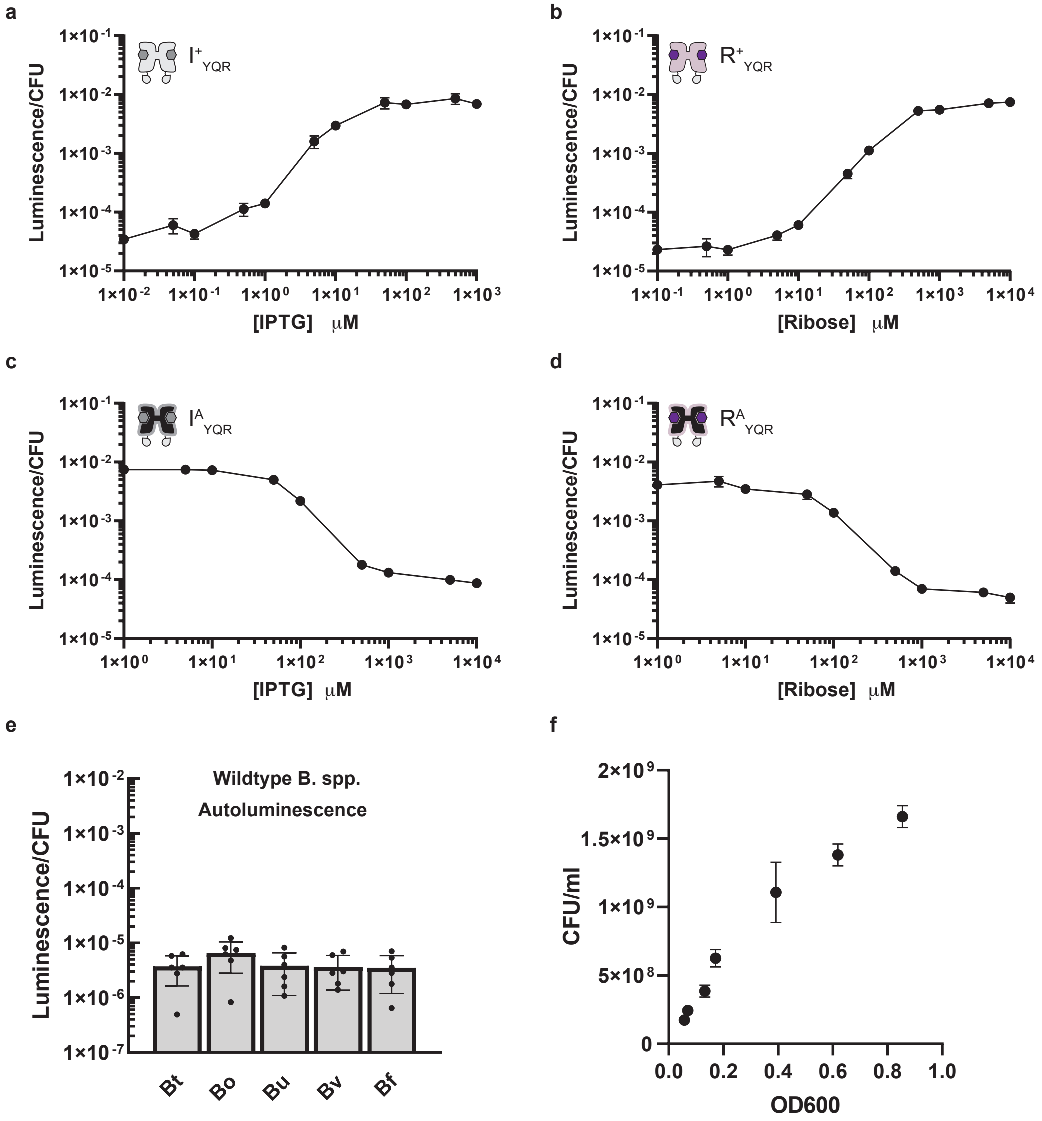

SI Fig. 4 | Transcription factor sensitivity and OD-CFU comparison. a, Dose response curve for I^{+}_{YQR} regulating P_{O1} in *B. thetaiotaomicron.* Cells were grown in TYG medium containing various concentrations of IPTG and assayed for luciferase activity (Methods) to assess transcription factor sensitivity. **b,** Dose response curve for R^{+}_{YQR} regulating P_{O1} in *B. thetaiotaomicron.* Cells were grown in TYG medium containing various concentrations of D-ribose and assayed for luciferase activity. **c,** Dose response curve for I^{A}_{YQR} regulating P_{O1} in *B. thetaiotaomicron.* Cells were grown in TYG medium containing various concentrations of IPTG and assayed for luciferase activity. **d,** Dose response curve for R^{A}_{YQR} regulating P_{O1} in *B. thetaiotaomicron.* Cells were grown in TYG medium containing various concentrations of D-ribose and assayed for luciferase activity. **e,** Autoluminescence of wildtype *Bacteroides.* Each species was grown in TYG medium and assayed for luminescence (Methods). **f,** OD-CFU curve. An example plot of OD600 of wildtype *B. thetaiotaomicron* grown in TYG medium converted to colony forming units (CFU). Cultures were grown for 16 hours with samples taken at regular intervals. Samples were serially diluted and plated on BHI agar to determine CFU. For luciferase assays, data represent the average of n = 6 biological replicates. Error bars correspond to the SEM of these measurements. For OD-CFU curves, data represent the average of n = 3 biological replicates. Error bars correspond to the SEM of these measurements.

Supplementary Fig. 5

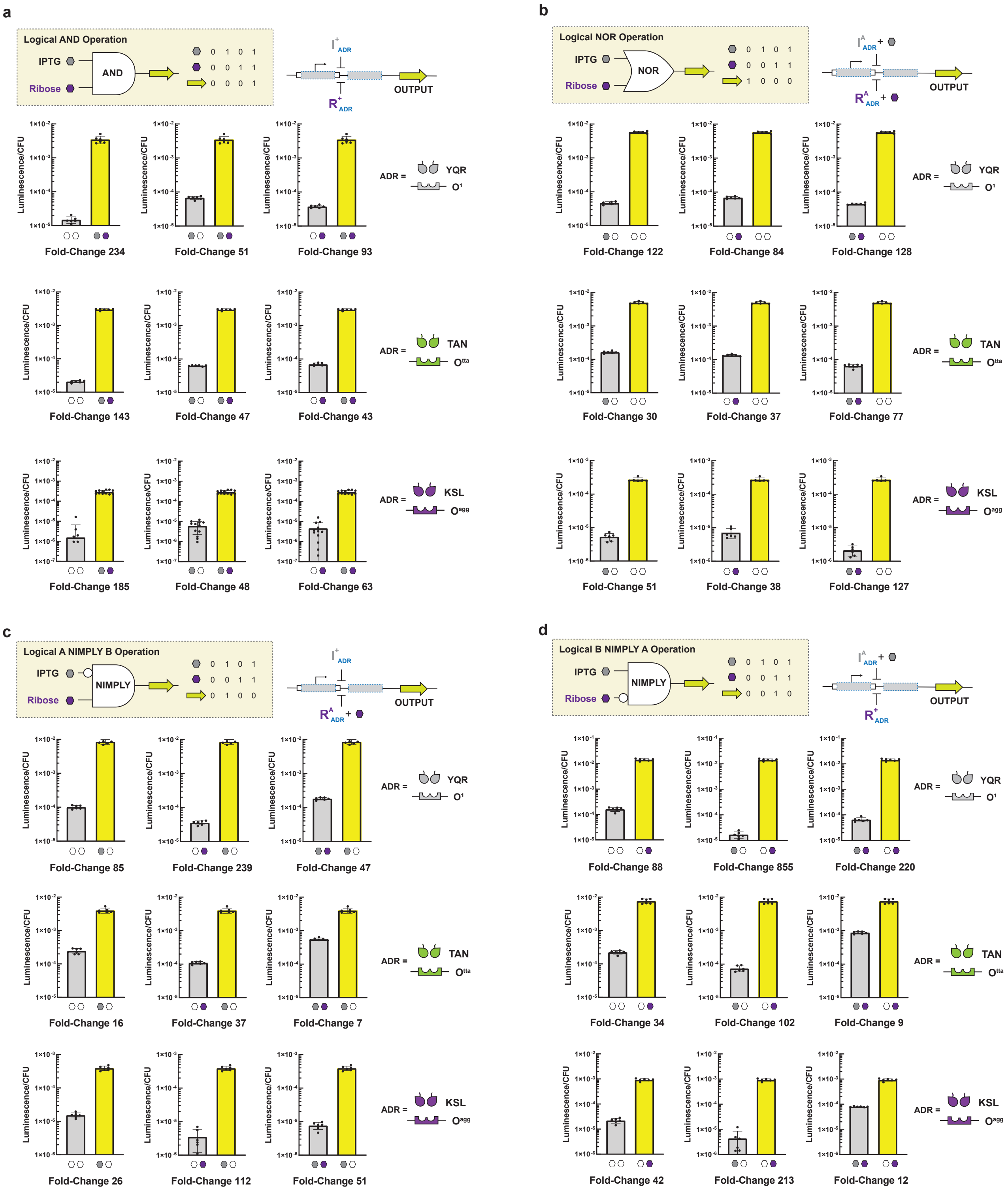

SI Fig. 5 | Additional single-promoter logic gates constructed in *B. thetaiotaomicron*. a, Additional AND gates created with different promoters. Each ligand condition is compared to the ON state with the corresponding fold-change in luciferase activity shown below. Cultures were assayed as described in **Fig.**

2. The ADR of the transcription factors used in each circuit are shown to the right of each row. **b,** Additional NOR gates created with different promoters. Each ligand condition is compared to the ON state with the corresponding fold-change in luciferase activity shown below. **c,** Additional A NIMPLY B gates created with different promoters. Each ligand condition is compared to the ON state with the corresponding fold-change in luciferase activity shown below. **d,** Additional B NIMPLY A gates created with different promoters. Each ligand condition is compared to the ON state with the corresponding fold-change in luciferase activity shown below. Data represent the average of n = 6 biological replicates. Error bars correspond to the SEM of these measurements.

Supplementary Fig. 6

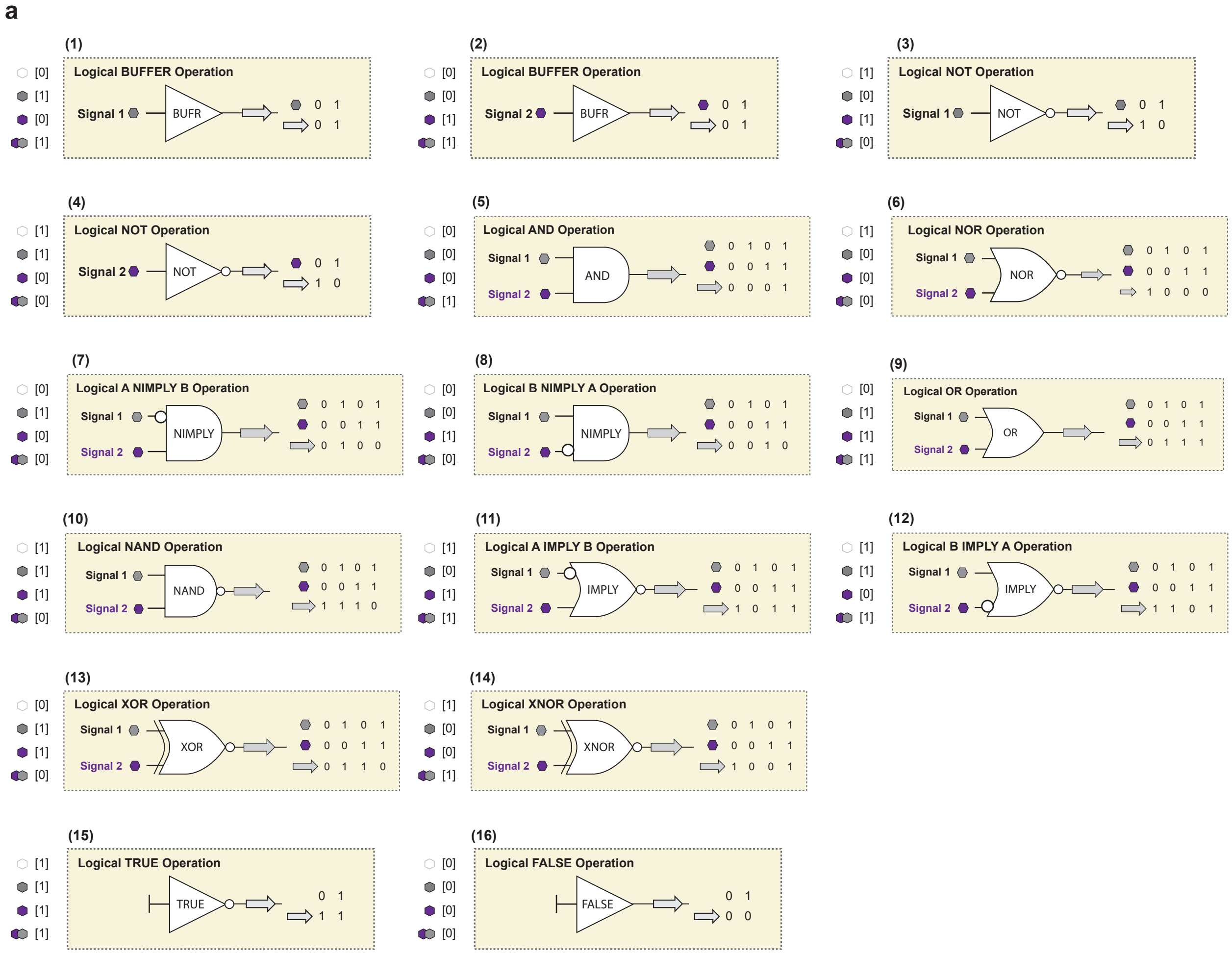

b

Gain-of-Function Consortium Transcriptional Programming Chart

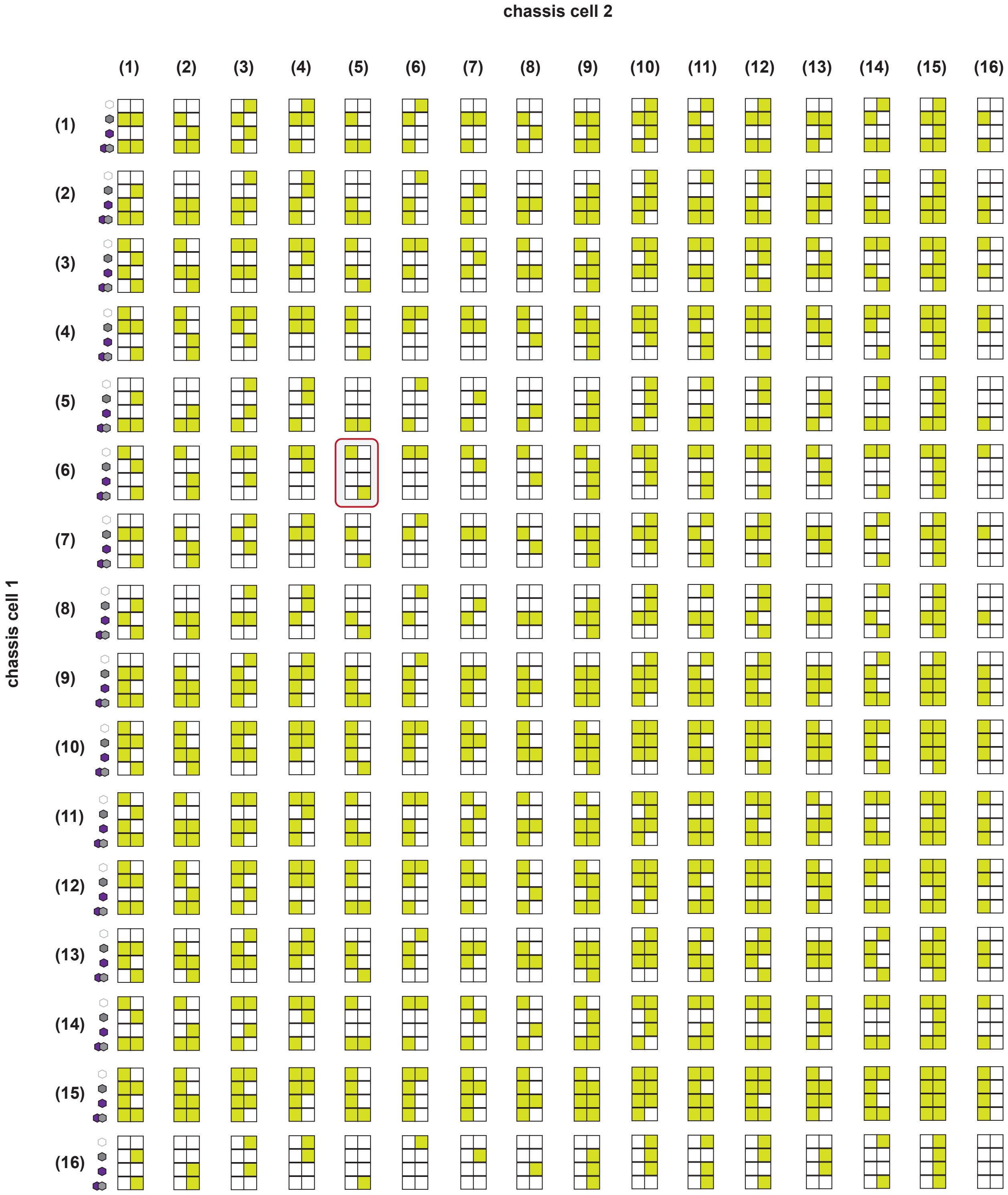

2 chassis cells = 16^2 (256)   3 chassis cells = 16^3 (4,096)   4 chassis cells = 16^4 (65,536)   5 chassis cells = 16^5 (1,048,576)

c

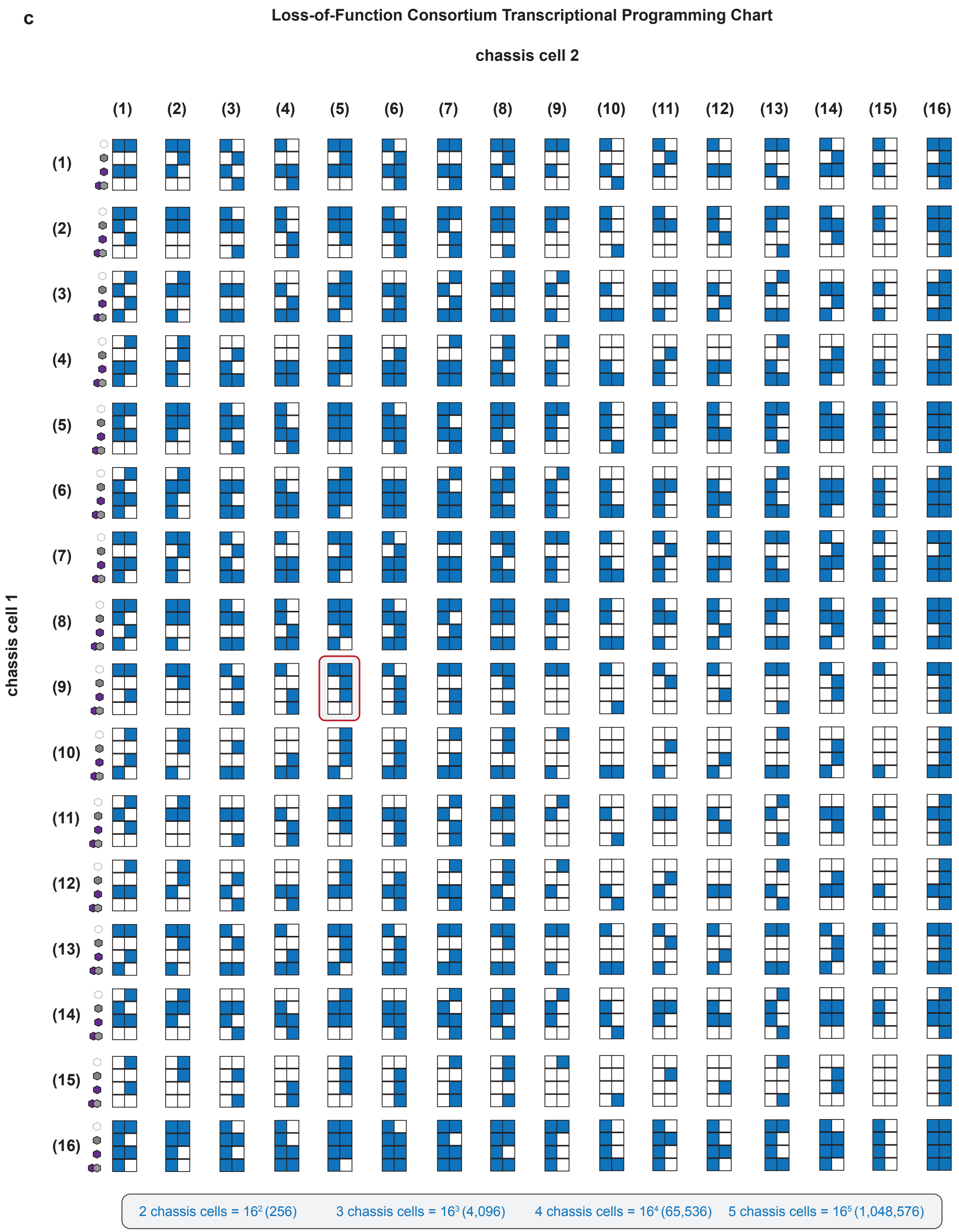

SI Fig. 6 | A complete set of 16 logic gates and consortium transcriptional programming charts. a, The full set of 16 two-input logic gates is presented for reference. Each gate has a corresponding truth table

shown to the right. **b**, Gain-of-function programming chart for co-culture. Each logic gate in **a** is listed vertically and horizontally to denote their potential use in chassis cell 1 or 2, respectively. The four input conditions (ligand combinations) are shown adjacent to the first column. A yellow box indicates that under the specified ligand condition gene expression will be activated in the corresponding chassis cell (chassis 1 is on the left and chassis 2 is on the right for each 2x4 grid). A white box indicates gene expression is off. Considering the co-culture experiment described in **Fig. 6**, we can designate *Bo* as chassis cell 1 and *Bt* as chassis cell 2. The red rectangle thus indicates the program used in **Fig. 6**. **c**, Loss-of-function programming chart for co-culture. This chart is analogous to **b**, but in the context of CRISPRi knockdown of a target gene. A blue box indicates a state of high gene expression while a white box indicates a knockdown state of gene expression. Considering the co-culture experiment described in **Fig. 7**, we can designate *Bo* as chassis cell 1 and *Bu* as chassis cell 2. The red rectangle indicates the program used in **Fig. 7**.

Supplementary Fig. 7

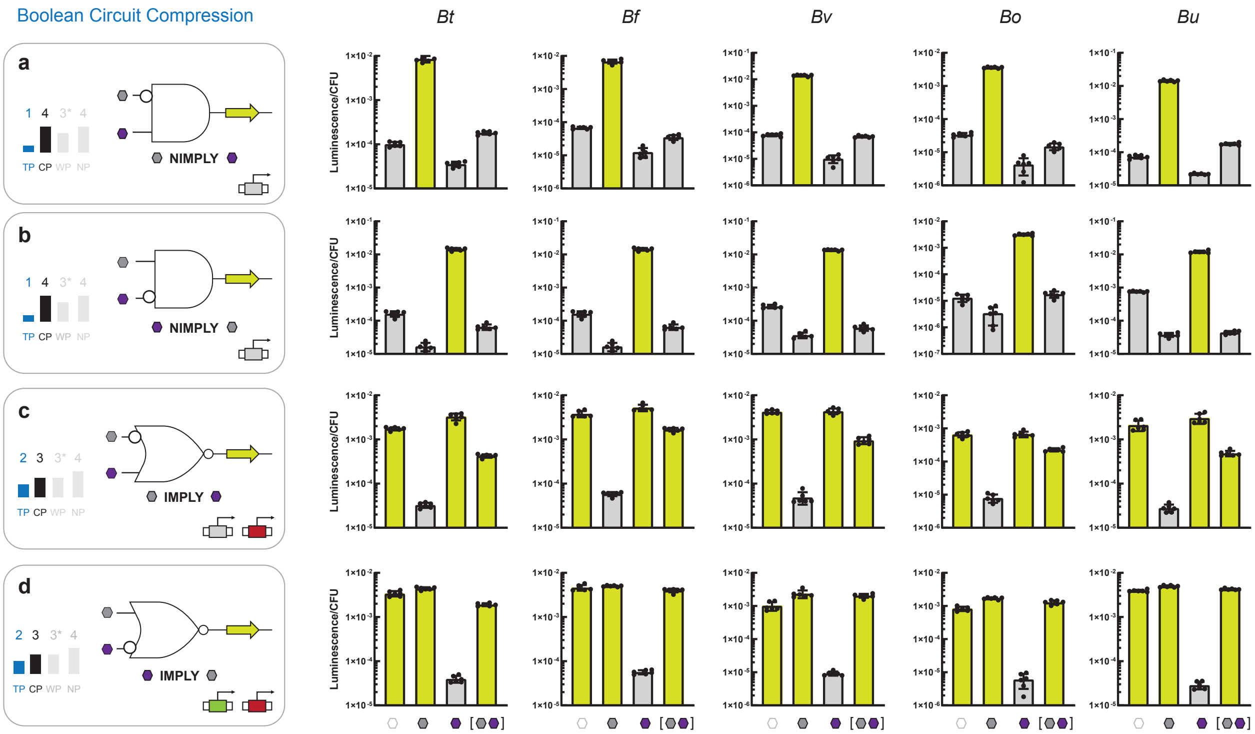

SI Fig. 7 | Circuit compression of NIMPLY and IMPLY gates. Related to **Fig. 3**, degree of compression (left) is presented alongside performance of logic gates in five *Bacteroides* species (right). **a-b,** NIMPLY gate compression and performance in five *Bacteroides*. See **Fig. 2** and **Supplementary Fig. 8e-f** for more details regarding gate construction. Degree of circuit compression is represented by the number of regulated promoters required to construct the logic gate. Strains harboring circuits were grown in the presence of all combinations of both inducers and assayed for luciferase activity (**Methods**). **c-d,** IMPLY gate compression and performance in five *Bacteroides*. See **Supplementary Fig. 8i-j** for details regarding gate construction. Regulated promoters used for each logic gate are shown in the bottom right corner of each left-hand box (also see **Fig. 1** for more detail). Data represent the average of n = 6 biological replicates. Error bars correspond to the SEM of these measurements.

Supplementary Fig. 8 (single-layer logic gates)

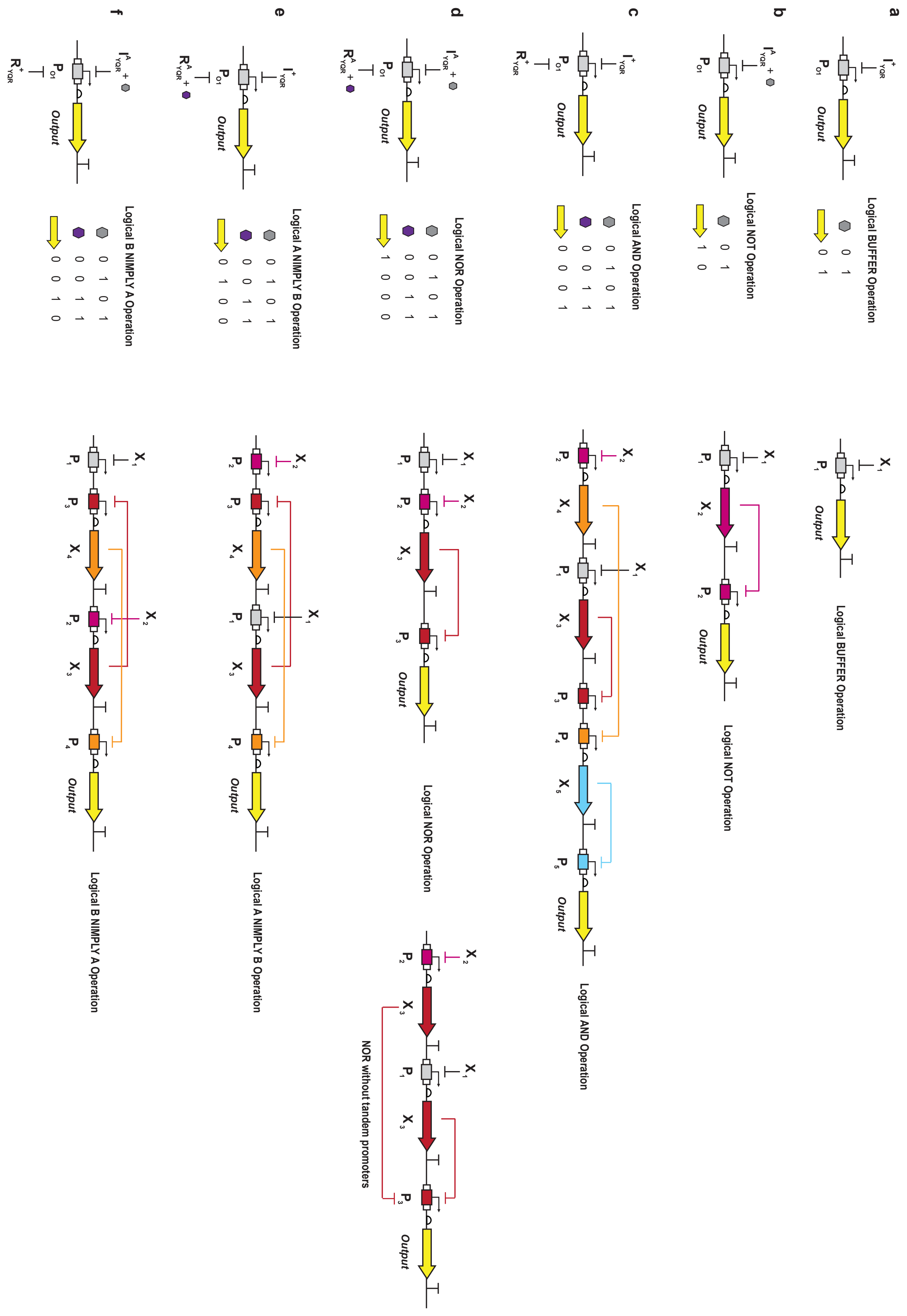

Supplementary Fig. 8 (two-layer logic gates)

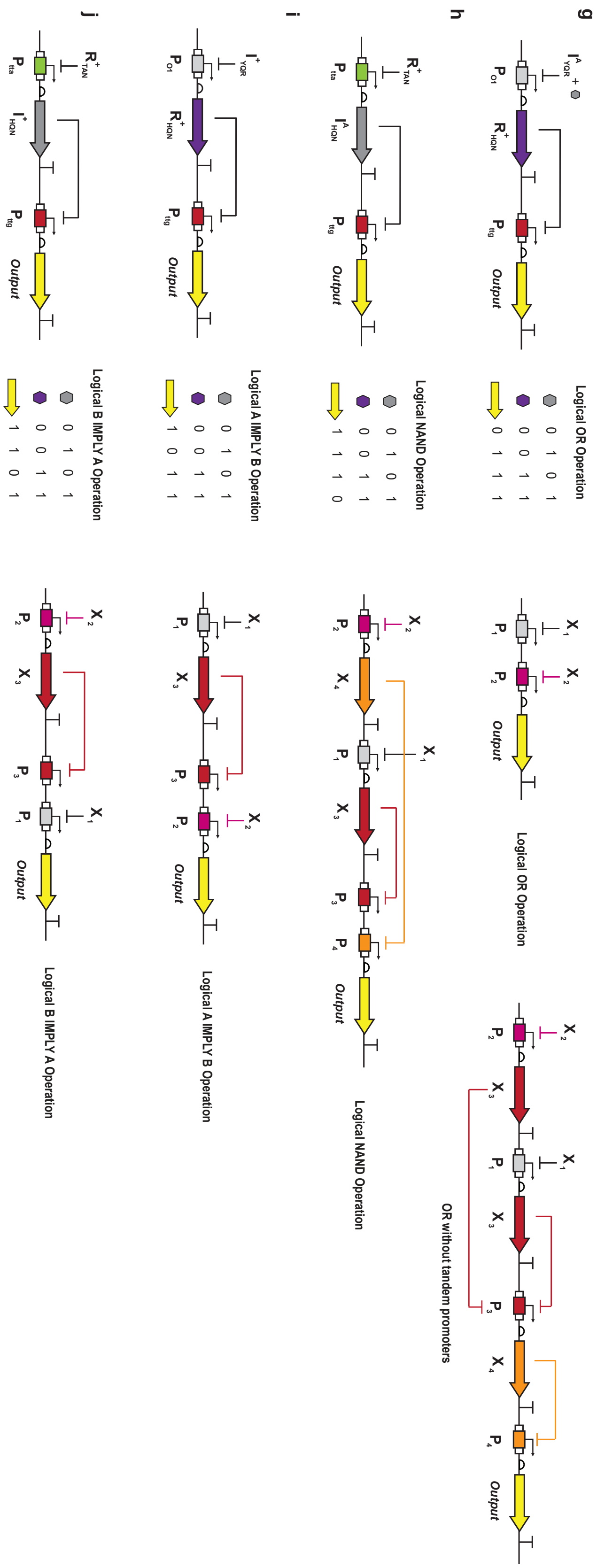

Supplementary Fig. 8 (three-layer logic gates)

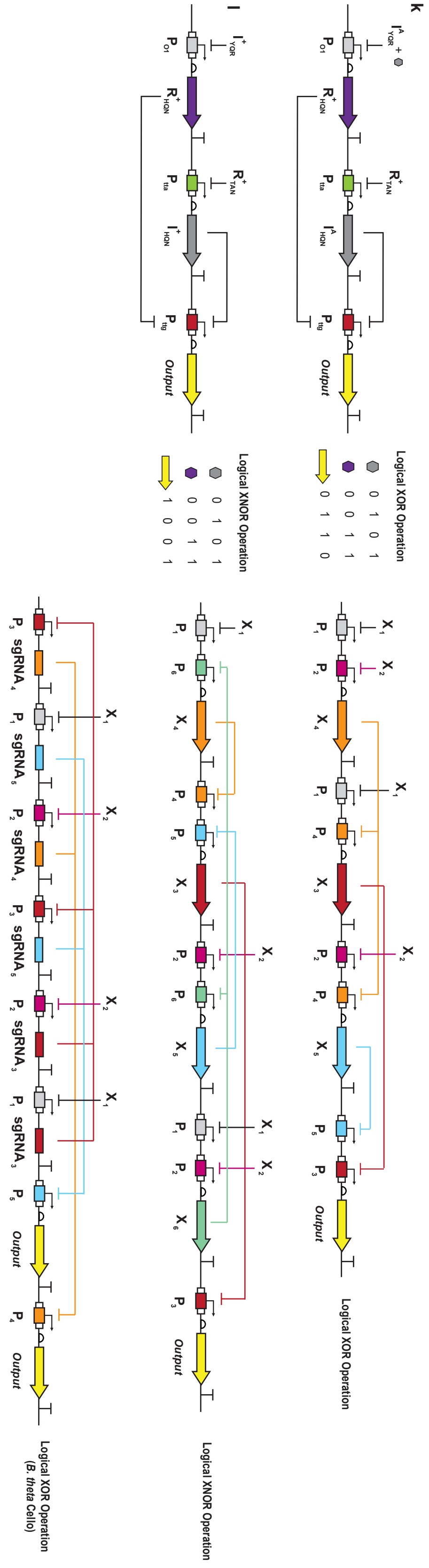

SI Fig. 8 | Direct comparison of compressed circuits with Cello programming (3 pages). Wiring diagrams are presented for all logic gates constructed in this study (left) as well as the equivalent logic gates reported in Nielsen *et al.*[3] (right). For circuits built in this study, the promoters correspond to those shown in **Fig. 1**. The binary truth table for the logic gate is shown to the right. For Cello circuits, X indicates a generic TF with the subscript indicating the cognate promoter it recognizes. Only X_1 and X_2 are used as inducible TFs, while all other TFs are used only to repress their cognate promoters. **a,** BUFFER gate. **b,** NOT gate. **c,** AND gate. **d,** NOR gate. The reported Cello NOR gate is shown (middle) along with a theoretical NOR gate constructed without tandem promoters (right). **e,** A NIMPLY B gate. **f,** B NIMPLY A gate. **g,** OR gate. The reported Cello OR gate is shown (middle) along with a theoretical OR gate constructed without tandem promoters (right). **h,** NAND gate. **i,** A IMPLY B gate. **j,** B IMPLY A gate. **k,** XOR gate. An additional apparent XOR gate reported by Taketani *et al.*[4] is displayed at the bottom. This circuit utilizes CRISPRi and requires a constitutively expressed dCas9 gene. X_1 and X_2 are inducible TFs that recognize P_1 and P_2, respectively. The circuit relies on sgRNAs to repress synthetic promoters and utilizes two output genes to achieve apparent XOR phenotype. **l,** XNOR gate.

Supplementary Fig. 9

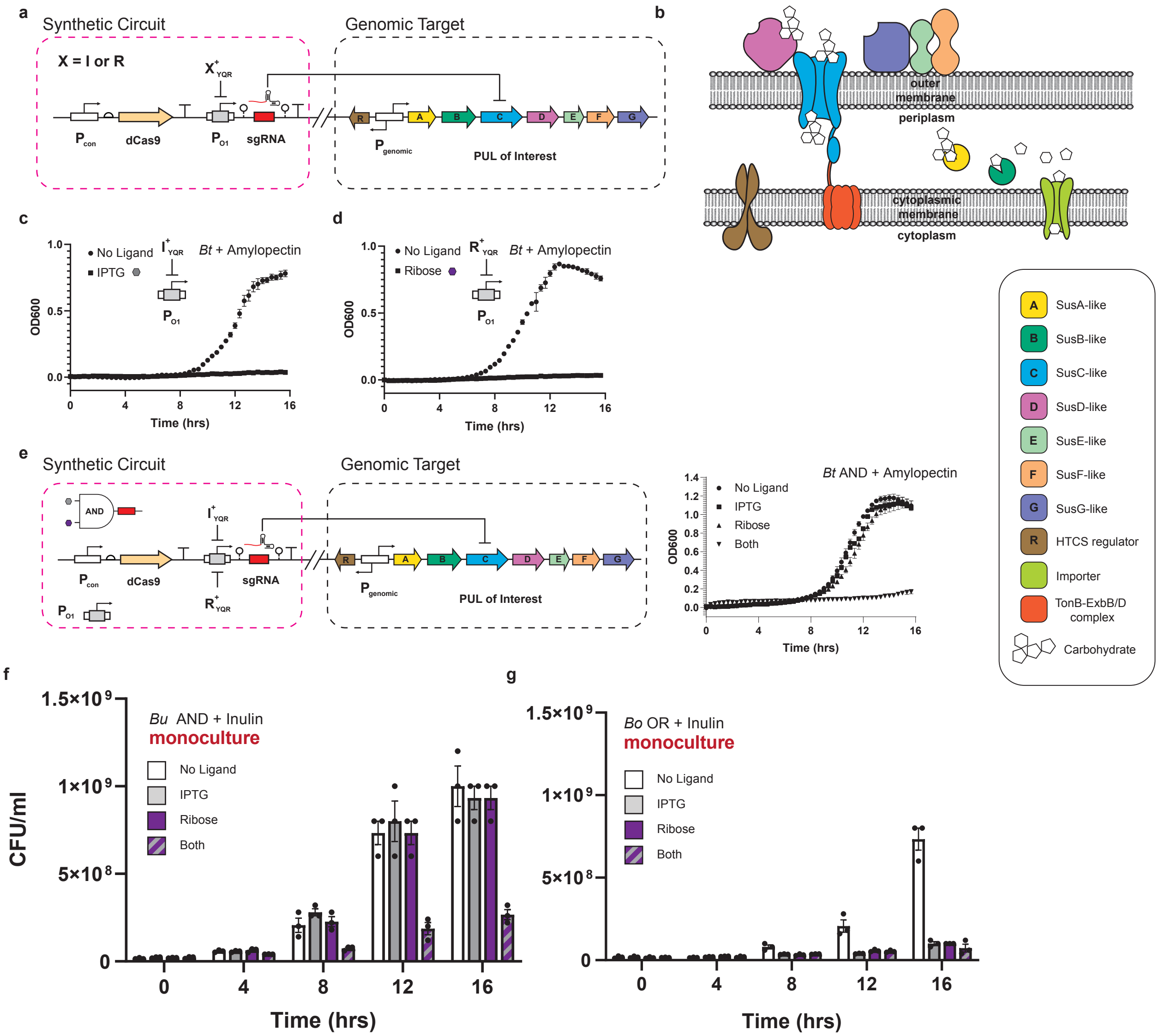

SI Fig. 9 | Demonstration of SusC knockdown in *B. thetaiotaomicron*. a, Wiring diagram of CRISPRi circuit targeting endogenous SusC-like gene. Strains were created with X^{+}_{YQR} regulating a sgRNA specific to the *B. thetaiotaomicron* amylopectin SusC gene. **b,** Cartoon of PUL organization, highlighting the function of the SusC-like importer. **c,** Growth curves of *B. thetaiotaomicron* harboring circuit shown in **a** with I^{+}_{YQR} as the sgRNA regulator. **d,** Growth curves of *B. thetaiotaomicron* harboring circuit shown in **a** with R^{+}_{YQR} as the sgRNA regulator. Strains harboring CRISPRi circuits were grown in the absence and presence of inducer in minimal media containing amylopectin as the only carbon source (Methods). **e,** Wiring diagram for an AND gate controlling the sgRNA targeting *B. thetaiotaomicron* amylopectin SusC gene (left). Growth curves of *B. thetaiotaomicron* harboring this circuit when grown in amylopectin minimal media containing all combinations of both inducers (right). For OD600 growth curves, data represent the average of n = 3 biological replicates. Error bars correspond to the SEM of these measurements. **f-g,** related to **Fig. 6**. The monoculture growth curve experiments presented in **Fig. 5a** and **Fig. 5c** were repeated using dilution plating to quantify culture density instead of OD600. Data represent the average of n = 3 technical replicates. Error bars correspond to the SEM of these measurements. This provides a direct comparison to the co-culture data presented in **Fig. 6**.

Supplementary Fig. 10

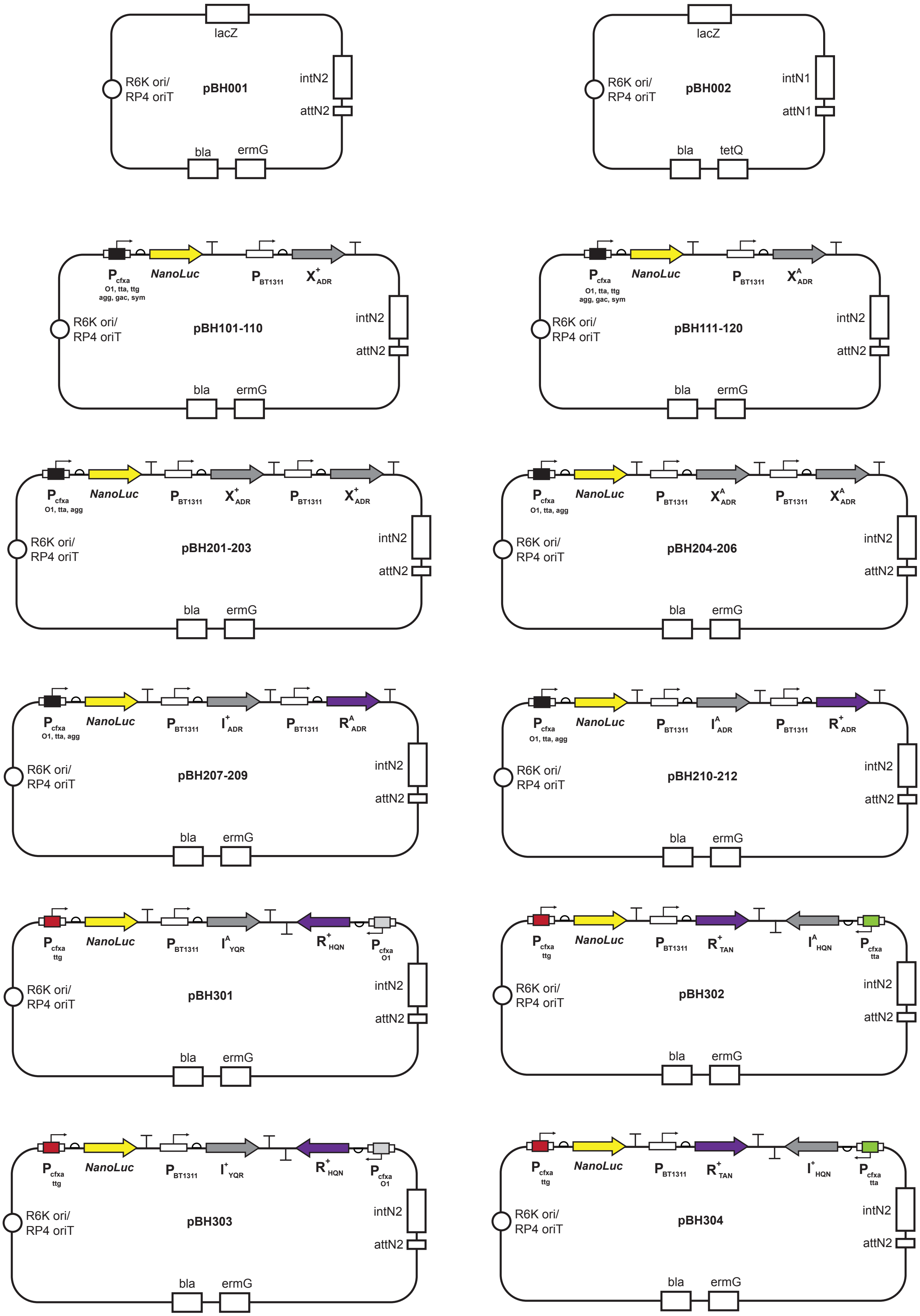

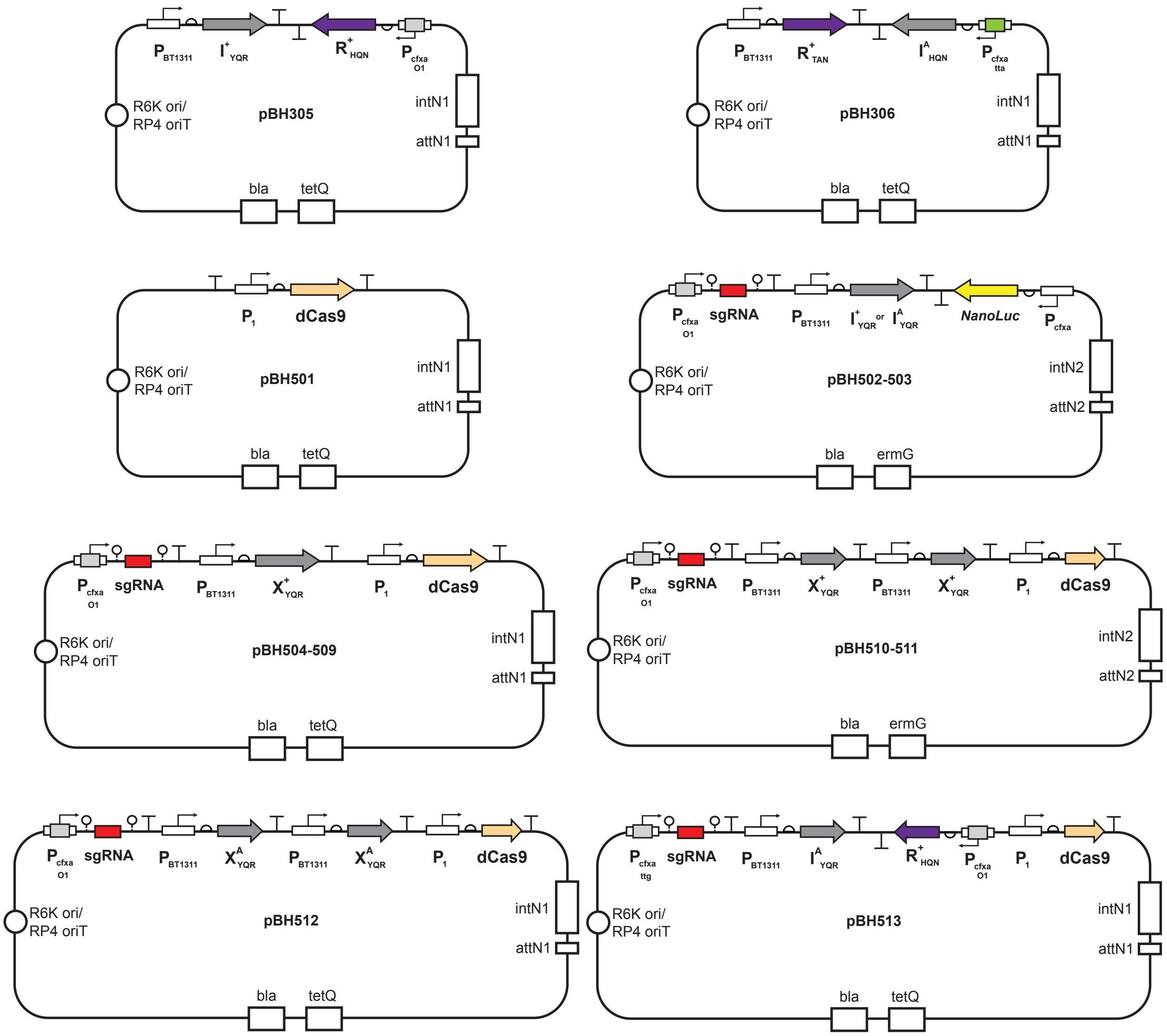

SI Fig. 10 | Maps of plasmids constructed in this study (2 pages). Plasmid names correspond to descriptions in **SI Table 1**.

Supplementary Table 1: Plasmids constructed in this work.

Designation	Genetic Parts	Backbone	Description
pBH001	intN2_ErmG_Bla_LacZ_BsmBIsites	NBU2	cloning vector
pBH002	intN1_TetQ_Bla_LacZ_BsmBIsites	NBU1	cloning vector
pBH101	LacI(YQR)_pCFXA_O1CP_nanoluc	NBU2	BUFFER Gate LacI(ADR)
pBH102	LacI(TAN)_pCFXA_OttaCP_nanoluc	NBU2	BUFFER Gate LacI(ADR)
pBH103	LacI(KSL)_pCFXA_OaggCP_nanoluc	NBU2	BUFFER Gate LacI(ADR)
pBH104	LacI(HQN)_pCFXA_OttgCP_nanoluc	NBU2	BUFFER Gate LacI(ADR)
pBH105	LacI(GKR)_pCFXA_OgacCP_nanoluc	NBU2	BUFFER Gate LacI(ADR)
pBH106	RbsR(YQR)_pCFXA_O1CP_nanoluc	NBU2	BUFFER Gate RbsR(ADR)
pBH107	RbsR(TAN)_pCFXA_OttaCP_nanoluc	NBU2	BUFFER Gate RbsR(ADR)
pBH108	RbsR(KSL)_pCFXA_OaggCP_nanoluc	NBU2	BUFFER Gate RbsR(ADR)
pBH109	RbsR(HQN)_pCFXA_OttgCP_nanoluc	NBU2	BUFFER Gate RbsR(ADR)
pBH110	RbsR(GKR)_pCFXA_OgacCP_nanoluc	NBU2	BUFFER Gate RbsR(ADR)
pBH111	IA(9)(YQR)_pCFXA_O1CP_nanoluc	NBU2	NOT Gate IA(9)(ADR)
pBH112	IA(9)(TAN)_pCFXA_OttaCP_nanoluc	NBU2	NOT Gate IA(9)(ADR)
pBH113	IA(9)(KSL)_pCFXA_OaggCP_nanoluc	NBU2	NOT Gate IA(9)(ADR)
pBH114	IA(9)(HQN)_pCFXA_OttgCP_nanoluc	NBU2	NOT Gate IA(9)(ADR)
pBH115	IA(9)(GKR)_pCFXA_OgacCP_nanoluc	NBU2	NOT Gate IA(9)(ADR)
pBH116	RA(1)(YQR)_pCFXA_O1CP_nanoluc	NBU2	NOT Gate RA(1)(ADR)
pBH117	RA(1)(TAN)_pCFXA_OttaCP_nanoluc	NBU2	NOT Gate RA(1)(ADR)
pBH118	RA(1)(KSL)_pCFXA_OaggCP_nanoluc	NBU2	NOT Gate RA(1)(ADR)
pBH119	RA(1)(HQN)_pCFXA_OttgCP_nanoluc	NBU2	NOT Gate RA(1)(ADR)
pBH120	RA(1)(GKR)_pCFXA_OgacCP_nanoluc	NBU2	NOT Gate RA(1)(ADR)
pBH201	LacI(YQR)_RbsR(YQR)_pCFXA_O1CP_nanoluc	NBU2	AND Gate
pBH202	LacI(TAN)_RbsR(TAN)_pCFXA_OttaCP_nanoluc	NBU2	AND Gate
pBH203	LacI(KSL)_RbsR(KSL)_pCFXA_OaggCP_nanoluc	NBU2	AND Gate
pBH204	IA(9)(YQR)_RA(1)(YQR)_pCFXA_O1CP_nanoluc	NBU2	NOR Gate
pBH205	IA(9)(TAN)_RA(1)(TAN)_pCFXA_OttaCP_nanoluc	NBU2	NOR Gate
pBH206	IA(9)(KSL)_RA(1)(KSL)_pCFXA_OaggCP_nanoluc	NBU2	NOR Gate
pBH207	LacI(YQR)_RA(1)(YQR)_pCFXA_O1CP_nanoluc	NBU2	I NIMPLY R Gate
pBH208	LacI(TAN)_RA(1)(TAN)_pCFXA_OttaCP_nanoluc	NBU2	I NIMPLY R Gate
pBH209	LacI(KSL)_RA(1)(KSL)_pCFXA_OaggCP_nanoluc	NBU2	I NIMPLY R Gate
pBH210	IA(9)(YQR)_RbsR(YQR)_pCFXA_O1CP_nanoluc	NBU2	R NIMPLY I Gate
pBH211	IA(9)(TAN)_RbsR(TAN)_pCFXA_OttaCP_nanoluc	NBU2	R NIMPLY I Gate
pBH212	IA(9)(KSL)_RbsR(KSL)_pCFXA_OaggCP_nanoluc	NBU2	R NIMPLY I Gate
pBH301	IA(9)(YQR)_pCFXA_O1CP_RbsR(HQN)_pCFXA_OttgCP_nanoluc	NBU2	OR Gate
pBH302	RbsR(TAN)_pCFXA_ttaCP_IA(9)(HQN)_pCFXA_OttgCP_nanoluc	NBU2	NAND Gate
pBH303	LacI(YQR)_pCFXA_O1CP_RbsR(HQN)_pCFXA_OttgCP_nanoluc	NBU2	I IMPLY R Gate

pBH304	RbsR(TAN)_pCFXA_ttaCP_LacI(HQN)_pCFXA_OttgCP_nanoluc	NBU2	R IMPLY I Gate, partial XNOR gate
pBH305	LacI(YQR)_pCFXA_O1CP_RbsR(HQN)	NBU1	XNOR Gate (partial)
pBH306	RbsR(TAN)_pCFXA_ttaCP_IA(9)(HQN)	NBU1	XOR Gate (partial)
pBH401	pCFXA_O1_nanoluc	NBU2	Reporter gene for off-diagonal testing
pBH402	pCFXA_Otta_nanoluc	NBU2	Reporter gene for off-diagonal testing
pBH403	pCFXA_Oagg_nanoluc	NBU2	Reporter gene for off-diagonal testing
pBH404	pCFXA_Ottg_nanoluc	NBU2	Reporter gene for off-diagonal testing
pBH405	pCFXA_Ogac_nanoluc	NBU2	Reporter gene for off-diagonal testing
pBH406	LacI(YQR)	NBU1	Single TF for off-diagonal testing
pBH407	LacI(TAN)	NBU1	Single TF for off-diagonal testing
pBH408	LacI(KSL)	NBU1	Single TF for off-diagonal testing
pBH409	LacI(HQN)	NBU1	Single TF for off-diagonal testing
pBH410	LacI(GKR)	NBU1	Single TF for off-diagonal testing
pBH411	IA(9)(YQR)	NBU1	Single TF for off-diagonal testing
pBH412	IA(9)(TAN)	NBU1	Single TF for off-diagonal testing
pBH413	IA(9)(KSL)	NBU1	Single TF for off-diagonal testing
pBH414	IA(9)(HQN)	NBU1	Single TF for off-diagonal testing
pBH415	IA(9)(GKR)	NBU1	Single TF for off-diagonal testing
pBH416	RbsR(YQR)	NBU1	Single TF for off-diagonal testing
pBH417	RbsR(TAN)	NBU1	Single TF for off-diagonal testing
pBH418	RbsR(KSL)	NBU1	Single TF for off-diagonal testing
pBH419	RbsR(HQN)	NBU1	Single TF for off-diagonal testing
pBH420	RbsR(GKR)	NBU1	Single TF for off-diagonal testing
pBH421	RA(1)(YQR)	NBU1	Single TF for off-diagonal testing
pBH422	RA(1)(TAN)	NBU1	Single TF for off-diagonal testing
pBH423	RA(1)(KSL)	NBU1	Single TF for off-diagonal testing
pBH424	RA(1)(HQN)	NBU1	Single TF for off-diagonal testing
pBH425	RA(1)(GKR)	NBU1	Single TF for off-diagonal testing
pBH501	p1_dCas9	NBU1	Constitutive dCas9
pBH502	LacI(YQR)_pCFXA_O1CP_nano4sgRNA_pCFXA_nanoluc	NBU2	LacI-controlled sgRNA for nanoluc knockdown
pBH503	IA(9)(YQR)_pCFXA_O1CP_nano4sgRNA_pCFXA_nanoluc	NBU2	IA(9)-controlled sgRNA for nanoluc knockdown
pBH504	LacI(YQR)_pCFXA_O1CP_AmyC3sgRNA_p1_dCas9	NBU1	LacI-controlled sgRNA for Bt Amylopectin susC knockdown
pBH505	RbsR(YQR)_pCFXA_O1CP_AmyC3sgRNA_p1_dCas9	NBU1	RbsR-controlled sgRNA for Bt Amylopectin susC knockdown

pBH506	LacI(YQR)_pCFXA_O1CP_InuC4sgRNA_p1_dCas9	NBU1	LacI-controlled sgRNA for Bo Inulin susC knockdown
pBH507	RbsR(YQR)_pCFXA_O1CP_InuC4sgRNA_p1_dCas9	NBU1	RbsR-controlled sgRNA for Bo Inulin susC knockdown
pBH508	LacI(YQR)_pCFXA_O1CP_InuC6sgRNA_p1_dCas9	NBU1	LacI-controlled sgRNA for Bu Inulin susC knockdown
pBH509	RbsR(YQR)_pCFXA_O1CP_InuC6sgRNA_p1_dCas9	NBU1	RbsR-controlled sgRNA for Bu Inulin susC knockdown
pBH510	LacI(YQR)_RbsR(YQR)_pCFXA_O1CP_AmyC3sgRNA_p1_dCas9	NBU2	AND gate for Bt Amylopectin susC knockdown
pBH511	LacI(YQR)_RbsR(YQR)_pCFXA_O1CP_InuC6sgRNA_p1_dCas9	NBU2	AND gate for Bu Inulin susC knockdown
pBH512	IA(9)(YQR)_RA(1)(YQR)_pCFXA_O1CP_InuC4sgRNA_p1_dCas9	NBU1	NOR gate for Bo Inulin susC knockdown
pBH513	IA(9)(YQR)_pCFXA_O1CP_RbsR(HQN)_pCFXA_OttgCP_InuC4sgRNA_p1_dCas9	NBU1	OR gate for Bo Inulin susC knockdown

Supplementary Table 2: Genetic parts used in this work.

Name	Type	Sequence
LacI(YQR)	gene	gtgaaaccagtaacgttatacgatgtcgcagagtatgccggtgtctcttatcagaccgtttcccgcgtggtgaaccaggccagccacgtttctgcgaaaa<br>cgcgggaaaaagtggaagcggcgatggcggagctgaattacattcccaaccgcgtggcacaacaactggcgggcaaacagtcgttgctgattggc<br>gttgccacctccagtctggccctgcacgcgccgtcgcaaattgtcgcggcgattaaatctcgcgccgatcaactgggtgccagcgtggtggtgtcgat<br>ggtagaacgaagcggcgtcgaagcctgtaaaacggcggtgcacaatcttctcgcgcaacgcgtcagtgggctgatcattaactatccgctggatgac<br>caggatgccattgctgtggaagctgcctgcactaatgttccggcgttatttcttgatgtctctgaccagacacccatcaacagtattattttctcccatgaag<br>acggtacgcgactgggcgtggagcatctggtcgcattgggtcaccagcaaatcgcgctgttagcgggcccattaagttctgtctcggcgcgtctgcgt<br>ctggctggctggcataaatatctcactcgcaatcaaattcagccgatagcggaacgggaaggcgactggagtgccatgtccggttttcaacaaaccatg<br>caaatgctgaatgagggcatcgttcccactgcgatgctggttgccaacgatcagatggcgctgggcgcaatgcgcgccattaccgagtccgggctgc<br>gcgttggtgcggatatctcggtagtgggatacgacgataccgaagacagctcatgttatatcccgccgttaaccaccatcaaacaggattttcgcctgct<br>ggggcaaaccagcgtggaccgcttgctgcaactctctcagggccaggcggtgaagggcaatcagctgttgcccgtctcactggtgaaaagaaaaac<br>caccctggcgcccaatacgcaaaccgcctctccccgcgcgttggccgattcattaatgcagctggcacgacaggtttcccgactggaaagcgggcag<br>tga
RbsR(YQR)	gene	gtgaaaccagtaacgttatacgatgtcgcagagtatgccggtgtctcttatcagaccgtttcccgcgtggtgaaccaggccagccacgtttctgcgaaaa<br>cgcgggaaaaagtggaagcggcgatggcggagctcaattacattcccaaccgcgtggcacaacaactggcgggcaaagcgtcgcataccattggc<br>atgttgatcactgccagtaccaatcctttctattcagaactggtgcgtggcgttgaacgcagctgcttcgaacgcggttatagtctcgtcctttgcaataccg<br>aaggcgatgaacagcggatgaatcgcaatctggaaacgctgatgcaaaaacgcgttgatggcttgctgttactgtgcaccgaaacgcatcaaccttcg<br>cgtgaaatcatgcaacgttatccgacagtgcctactgtgatgatggactgggctccgttcgatggcgacagcgatcttattcaggataactcgttgctgg<br>gcggagacttagcaacgcaatatctgatcgataaaggtcatacccgtatcgcctgtattaccggcccgctggataaaactccggcgcgcctgcggttg<br>gaaggttatcgggcggcgatgaaacgtgcgggtctcaacattcctgatggctatgaagtcactggtgattttgaatttaacggcgggtttgacgctatgc<br>gccaactgctatcacatccgctgcgtcctcaggccgtctttaccggaaatgacgctatggctgttggcgtttaccaggcgttatatcaggcagagttaca<br>ggttccgcaggatatcgcggtgattggctatgacgatatcgaactggcaagctttatgacgccaccattaaccactatccaccaaccgaaagatgaact<br>gggggagctggcgattgatgtactcatccatcggataacccagccgacccttcagcaacaacgattacaacttactccgattctgatggaacgcggttc<br>ggcttagctggtgaaaagaaaaaccaccctggcgcccaatacgcaaaccgcctctccccgcgcgttggccgattcattaatgcagctggcacgacag<br>gtttcccgactggaaagcgggcagtga
IA(9)(YQR)	gene	gtgaaaccagtaacgttatacgatgtcgcagagtatgccggtgtctcttatcagaccgtttcccgcgtggtgaaccaggccagccacgtttctgcgaaaa<br>cgcgggaaaaagtggaagcggcgatggcggagctgaattacattcccaaccgcgtggcacaacaactggcgggcaaacagtcgttgctgattggc<br>gttgccacctccagtctggccctgcacgcgctgtcgcaaattgtcgcggcgattaaatctcgcgcctatcaactgggtgccagcgtgttcgtgtcgatgg<br>tagaacgaagcggcatcgaagcctgtaaaacggcggtgcacaatcttctcgcgcaacgcgtcagtgggctgatcattaactatccgctggataaccag<br>gatgccattgctgtggaagctgcctgcactaatgttccggcgttatttcttgatgtctctgaccagacacccatcaacagtattattttctcccatgaagacg<br>gtacgcgactgggcgtggagcatctggtcgcattgggtcaccagcaaatcgcgctgttagcgggcccattaagttctgtctcggcgcgtctgcgtctgg<br>ctggctggcataaatatctcactcgcaatcaaattcagccgatagcggaacgggaaggcgactggagtgccatgtccggttttcaacaaaccatgcaa<br>atgctgaatgagggcatcgttcccactgcgatgctggttgccaacgatcagatggcgctgggcgcaatgcgcgccattaccgagaccgggctgcgcg<br>ttggtgcggatatctcggtagtgggatacgacgataccgaagacagctcatgttatatcccgccgttaaccaccatcaaacaggattttcgcctgctggg<br>gcaaaccagcgtggaccgcttgctgcaactctctcagggccaggcggtgaagggcaatcagctgttgcccgtctcactggtgaaaagaaaaaccacc<br>ctggcgcccaatacgcaaaccgcctctccccgcgcgttggccgattcattaatgcagctggcacgacaggtttcccgactggaaagcgggcagtga
RA(1)(YQR)	gene	gtgaaaccagtaacgttatacgatgtcgcagagtatgccggtgtctcttatcagaccgtttcccgcgtggtgaaccaggccagccacgtttctgcgaaaa<br>cgcgggaaaaagtggaagcggcgatggcggagctcaattacattcccaaccgcgtggcacaacaactggcgggcaaagcgtcgcataccattggc<br>atgttgatcactgccagtaccaatcctttctattcagaactggtgcgtggcgttgaacgcagctgcttcgaacgcggttatagtctcgatctttgcaataccg<br>aaggcgatgaacagcggatgaatcgcaatctggaaacgctgatgcaaaaacgcgttgatggcttgctgttactgtgcaccgaaacgcatcaaccttcg<br>cgtgaaatcatgcaacgttatccgacagtgcctactgtgatgatggactgggctccgttcgatggcgacagcgatcttattcaggataactcgttgctgg<br>gcggagacttagcaacgcaatatctgatcgataaaggtcatacccgtatcgcctgtattaccggcccgctggataaaactccggcgcgcctgcggttg<br>gaaggttatcgggcggcgatgaaacgtgcgggtctcaacattcctgatggctatgaagtcactggtgattttgaatttaacggcgggtttgacgctatgc<br>gccaactgctatcacatccgctgcgtcctcaggccgtctttaccggaaatgacgctatggctgttggcgtttaccaggcgttatatcaggcagagttaca<br>ggttccgcaggatatcgcggtgattggctatgacgatatcgaactggcaagctttatgacgccaccattaaccactatccaccaaccgaaagatgaact<br>gggggagctggcgattgatgtactcatccatcggataacccagccgacccttcagcaacaacgattacaacttactccgattctgatggaacgcggttc<br>ggcttagctggtgaaaagaaaaaccaccctggcgcccaatacgcaaaccgcctctccccgcgcgttggccgattcattaatgcagctggcacgacag<br>gtttcccgactggaaagcgggcagtga
NanoLuc	gene	atggtttttactctggaagattttgttggcgattggcgtcagaccgcgggttataatttggatcaagtcctggaacagggtggcgtaagctctctgttccag<br>aacctgggtgtgagcgtgacgccgattcagcgcatcgttctgtccggcgagaacggtctgaaaattgatattcatgtgatcatcccgtacgaaggcctg<br>agcggtgaccaaatgggtcaaatcgagaaaatctttaaagtcgtctacccagttgacgatcaccacttcaaggttatcttgcattacggtacgctggtgatt<br>gatggtgtgaccccgaatatgattgactatttcggccgtccgtatgaaggcattgccgtttttgacggtaaaaagatcaccgtcaccggtaccctgtggaa<br>tggcaataagattattgacgagcgtctgattaacccggacggcagcctgctgttccgcgtgaccatcaacggtgtcacgggttggcgtctgtgcgagcg<br>catcctggcataa

dCas9	gene	atggataagaaatactcaataggcttagctatcggcacaaatagcgtcggatgggcggtgatcactgatgaatataaggttccgtctaaaaagttcaagg<br>ttctgggaaatacagaccgccacagtatcaaaaaaaatcttataggggctcttttatttgacagtggagagacagcggaagcgactcgtctcaaacgga<br>cagctcgtagaaggtatacacgtcggaagaatcgtatttgttatctacaggagatttttcaaatgagatggcgaaagtagatgatagtttctttcatcgactt<br>gaagagtcttttttggtggaagaagacaagaagcatgaacgtcatcctatttttggaaatatagtagatgaagttgcttatcatgagaaatatccaactatct<br>atcatctgcgaaaaaaattggtagattctactgataaagcggatttgcgcttaatctatttggccttagcgcatatgattaagtttcgtggtcatttttttgattga<br>gggagatttaaatcctgataatagtgatgtggacaaactatttatccagttggtacaaacctacaatcaattatttgaagaaaaccctattaacgcaagtgg<br>agtagatgctaaagcgattctttctgcacgattgagtaaatcaagacgattagaaaatctcattgctcagctccccggtgagaagaaaaatggcttatttgg<br>gaatctcattgctttgtcattgggtttgacccctaattttaaatcaaattttgatttggcagaagatgctaaattacagctttcaaaagatacttacgatgatgatt<br>tagataatttattggcgcaaattggagatcaatatgctgatttgtttttggcagctaagaatttatcagatgctattttactttcagatatcctaagagtaaatact<br>gaaataactaaggctcccctatcagcttcaatgattaaacgctacgatgaacatcatcaagacttgactcttttaaaagctttagttcgacaacaacttccag<br>aaaagtataaagaaatcttttttgatcaatcaaaaaacggatatgcaggttatattgatgggggagctagccaagaagaattttataaatttatcaaaccaat<br>tttagaaaaaatggatggtactgaggaattattggtgaaactaaatcgtgaagatttgctgcgcaagcaacggacctttgacaacggctctattccccatc<br>aaattcacttgggtgagctgcatgctattttgagaagacaagaagacttttatccatttttaaaagacaatcgtgagaagattgaaaaaatcttgacttttcga<br>attccttattatgttggtccattggcgcgtggcaatagtcgttttgcatggatgactcggaagtctgaagaaacaattaccccatggaattttgaagaagttg<br>tcgataaaggtgcttcagctcaatcatttattgaacgcatgacaaactttgataaaaatcttccaaatgaaaaagtactaccaaaacatagtttgctttatgag<br>tattttacggtttataacgaattgacaaaggtcaaatatgttactgaaggaatgcgaaaaccagcatttctttcaggtgaacagaagaaagccattgttgatt<br>tactcttcaaaacaaatcgaaaagtaaccgttaagcaattaaaagaagattatttcaaaaaaatagaatgttttgatagtgttgaaatttcaggagttgaaga<br>tagatttaatgcttcattaggtacctaccatgatttgctaaaaattattaaagataaagatttttttggataatgaagaaaatgaagatatcttagaggatattgttt<br>taacattgaccttatttgaagatagggagatgattgaggaaagacttaaaacatatgctcacctctttgatgataaggtgatgaaacagcttaaacgtcgcc<br>gttatactggttggggacgtttgtctcgaaaattgattaatggtattagggataagcaatctggcaaaacaatattagatttttttgaaatcagatggttttgcca<br>atcgcaattttatgcagctgatccatgatgatagtttgacatttaaagaagacattcaaaaagcacaagtgtctggacaaggcgatagtttacatgaacata<br>ttgcaaatttagctggtagccctgctattaaaaaaggtattttacagactgtaaaagttgttgatgaattggtcaaagtaatggggcggcataagccagaaa<br>atatcgttattgaaatggcacgtgaaaatcagacaactcaaaagggccagaaaaattcgcgagagcgtatgaaacgaatcgaagaaggtatcaaaga<br>attaggaagtcagattcttaaagagcatcctgttgaaaatactcaattgcaaaatgaaaagctctatctctattatctccaaaatggaagagacatgtatgtg<br>gaccaagaattagatattaatcgtttaagtgattatgatgtcgatgccattgttccacaaagtttccttaaagacgattcaatagacaataaggtcttaacgcg<br>ttctgataaaaatcgtggtaaatcggataacgttccaagtgaagaagtagtcaaaaagatgaaaaactattggagacaacttctaaacgccaagttaatca<br>ctcaacgtaagtttgataatttaacgaaagctgaacgtggtggtttgagtgaacttgataaagctggttttatcaaacgccaattggttgaaactcgccaaat<br>cactaagcatgtggcacaaattttggatagtcgcatgaatactaaatacgatgaaaatgataaacttattcgagaggttaaagtgattaccttaaaatctaaa<br>ttagtttctgacttccgaaaagatttccaattctataaagtacgtgagattaacaattaccatcatgcccatgatgcgtatctaaatgccgtcgttggaactgc<br>tttgattaagaaatatccaaaacttgaatcggagtttgtctatggtgattataaagtttatgatgttcgtaaaatgattgctaagtctgagcaagaaataggca<br>aagcaaccgcaaaatatttcttttactctaatatcatgaacttcttcaaaacagaaattacacttgcaaatggagagattcgcaaacgccctctaatcgaaac<br>taatggggaaactggagaaattgtctgggataaagggcgagattttgccacagtgcgcaaagtattgtccatgccccaagtcaatattgtcaagaaaac<br>agaagtacagacaggcgaattctccaaggagtcaattttaccaaaaagaaattcggacaagcttattgctcgtaaaaaagactgggatccaaaaaaata<br>tggtggttttgatagtccaacggtagcttattcagtcctagtggttgctaaggtggaaaaagggaaatcgaagaagttaaaatccgttaaagagttactag<br>ggatcacaattatggaaagaagttcctttgaaaaaaatccgattgactttttagaagctaaaggatataaggaagttaaaaaagacttaatcattaaactac<br>ctaaatatagtctttttgagttagaaaacggtcgtaaacggatgctggctagtgccggagaattacaaaaaggaaatgagctggctctgccaagcaaata<br>tgtgaatttttatatttagctagtcattatgaaaagttgaagggtagtccagaagataacgaacaaaaacaattgtttgtggagcagcataagcattatttag<br>atgagattattgagcaaatcagtgaattttctaagcgtgttattttagcagatgccaatttagataaagttcttagtgcatataacaaacatagagacaaacca<br>atacgtgaacaagcagaaaatattattcatttatttacgttgacgaatcttggagctcccgctgcttttaaatattttgatacaacaattgatcgtaaacgatat<br>acgtctacaaaagaagttttagatgccactcttatccatcaatccatcactggtctttatgaaacacgcattgatttgagtcagctaggaggtgactga
P_{cfxA}	promoter	ttacaaagaaaattcgacaaactgttatttttctatctatttatttgggtgggaaactttagttatgtacctttgtcggc
P_1	promoter + RBS	gataaagtttggaagataaagctaaaagttcttatctttgcagtccgaaataaagacatataaaagaaaagacacc
P_{BT1311}	promoter + RBS	tgatctggaagaagcaatgaaagctgctgttaagtctccgaatcaggtattgttcctgacaggtgtattcccatccggtaaacgcggatactttgcagttga<br>tctgactcaggaataaattataaattaaggtaagaagattgtaggataagctaatgaaatagaaaaaggatgccgtcacacaacttgtcggcattctttttttg<br>ttttattagttgaaaatatagtgaaaaagttgcctaaatatgtatgttaacaaattatttgtcgtaactttgcactccaaatctgtttttaacatatggcacta
P_{cfxa}(O1)	promoter	tacaaagaaaattcgacaaactgttatttttctatctatttatttgaattgtgagcggataacaattacctttgtcggcaattgtgagcggataacaatt
P_{cfxa}(Otta)	promoter	tacaaagaaaattcgacaaactgttatttttctatctatttatttgaattttaagcgcttaaaatttacctttgtcggcaattttaagcgcttaaaatt
P_{cfxa}(Ogac)	promoter	tacaaagaaaattcgacaaactgttatttttctatctatttatttgaattgacagcgctgtcaatttacctttgtcggcaattgacagcgctgtcaatt
P_{cfxa}(Ottg)	promoter	tacaaagaaaattcgacaaactgttatttttctatctatttatttgaattttgagcgctcaaaatttacctttgtcggcaattttgagcgctcaaaatt
P_{cfxa}(Oagg)	promoter	tacaaagaaaattcgacaaactgttatttttctatctatttatttgaattaggagcgctcctaatttacctttgtcggcaattaggagcgctcctaatt
P_{cfxa}(Osym)	promoter	tacaaagaaaattcgacaaactgttatttttctatctatttatttgaattgtgagcgctcacaatttacctttgtcggcaattgtgagcgctcacaatt
nano4	sgRNA	gacagaacgatgcgctgaatgttttagagctagaaatagcaagttaaaataaggctagtccgttatcaacttgaaaaagtggcaccgagtcggtgcttttt<br>t
BOinu4	sgRNA	cctgacatcacattaccagtgttttagagctagaaatagcaagttaaaataaggctagtccgttatcaacttgaaaaagtggcaccgagtcggtgcttttttt
BUinu6	sgRNA	gtcaactcaatgcgtaaagtgttttagagctagaaatagcaagttaaaataaggctagtccgttatcaacttgaaaaagtggcaccgagtcggtgcttttttt
BTamy3	sgRNA	acaacattggcaccgataacgttttagagctagaaatagcaagttaaaataaggctagtccgttatcaacttgaaaaagtggcaccgagtcggtgcttttttt

HH ribozyme	5' ribozyme	nnnnnnctgatgagtccgtgaggacgaaacgagtaagctcgtc
HDV ribozyme	3' ribozyme	ggccggcatggtcccagcctcctcgctggcgccggctgggcaacatgcttcggcatggcgaatgggac
nanoTerm	terminator	gcactctaatcgttatcggagtgcttttagattactaatcaaattgcttcta
L3S2P55	terminator	ctcggtaccaaagacgaaCaataagacgctgaaaagcgtcttttttcgttttggtcc
L3S2P21	terminator	ctcggtaccaaattccagaaaagaggcctcccgaaaggggggccttttttcgttttggtcc
TAN DBD	partial gene	gtgaaaccagtaacgttatacgatgtcgcagagtatgccggtgtctctaccgcgaccgtttccaac
GKR DBD	partial gene	gtgaaaccagtaacgttatacgatgtcgcagagtatgccggtgtctctggaaagaccgtttcccgc
HQN DBD	partial gene	gtgaaaccagtaacgttatacgatgtcgcagagtatgccggtgtctctcatcagaccgtttccaat
KSL DBD	partial gene	gtgaaaccagtaacgttatacgatgtcgcagagtatgccggtgtctctaaaagcaccgtttccctg

a. For the HH ribozyme, the first 6 bp are the reverse complement of the 6 bp directly downstream of the ribozyme.
b. The DBD sequences include the first 22 residues of the gene, ending at the "R" of the "YQR" motif.

Supplemental References

1. Rondon, R.E., Groseclose, T.M., Short, A.E. & Wilson, C.J. Transcriptional programming using engineered systems of transcription factors and genetic architectures. *Nat Commun* **10**, 4784 (2019).
2. Groseclose, T.M., Rondon, R.E., Herde, Z.D., Aldrete, C.A. & Wilson, C.J. Engineered systems of inducible anti-repressors for the next generation of biological programming. *Nat Commun* **11**, 4440 (2020).
3. Nielsen, A.A. *et al.* Genetic circuit design automation. *Science* **352**, aac7341 (2016).
4. Taketani, M. *et al.* Genetic circuit design automation for the gut resident species Bacteroides thetaiotaomicron. *Nat Biotechnol* **38**, 962-969 (2020).
5. Tamsir, A., Tabor, J.J. & Voigt, C.A. Robust multicellular computing using genetically encoded NOR gates and chemical 'wires'. *Nature* **469**, 212-5 (2011).
6. Groseclose, T.M., Hersey, A.N., Huang, B.D., Realff, M.J. & Wilson, C.J. Biological signal processing filters via engineering allosteric transcription factors. *Proc Natl Acad Sci U S A* **118**(2021).
