# Supplementary material for: Transcriptional programming in a Bacteroides consortium
Source: Nat Commun. 2022 Jul 6;13:3901. doi: 10.1038/s41467-022-31614-8 (PMC9259675; doi:10.1038/s41467-022-31614-8)
Supplement: Supplementary file 2 — Reporting Summary [file 41467_2022_31614_MOESM2_ESM.pdf]

nature portfolio

Corresponding author(s): Corey J. Wilson

Last updated by author(s): Jun 24, 2022

Reporting Summary

Nature Portfolio wishes to improve the reproducibility of the work that we publish. This form provides structure for consistency and transparency in reporting. For further information on Nature Portfolio policies, see our Editorial Policies and the Editorial Policy Checklist.

Statistics

For all statistical analyses, confirm that the following items are present in the figure legend, table legend, main text, or Methods section.

n/a	Confirmed	
☐	☒	The exact sample size (*n*) for each experimental group/condition, given as a discrete number and unit of measurement
☐	☒	A statement on whether measurements were taken from distinct samples or whether the same sample was measured repeatedly
☐	☒	The statistical test(s) used AND whether they are one- or two-sided<br>*Only common tests should be described solely by name; describe more complex techniques in the Methods section.*
☒	☐	A description of all covariates tested
☒	☐	A description of any assumptions or corrections, such as tests of normality and adjustment for multiple comparisons
☐	☒	A full description of the statistical parameters including central tendency (e.g. means) or other basic estimates (e.g. regression coefficient) AND variation (e.g. standard deviation) or associated estimates of uncertainty (e.g. confidence intervals)
☐	☒	For null hypothesis testing, the test statistic (e.g. *F*, *t*, *r*) with confidence intervals, effect sizes, degrees of freedom and *P* value noted<br>*Give P values as exact values whenever suitable.*
☒	☐	For Bayesian analysis, information on the choice of priors and Markov chain Monte Carlo settings
☒	☐	For hierarchical and complex designs, identification of the appropriate level for tests and full reporting of outcomes
☒	☐	Estimates of effect sizes (e.g. Cohen's *d*, Pearson's *r*), indicating how they were calculated

Our web collection on statistics for biologists contains articles on many of the points above.

Software and code

Policy information about availability of computer code

Data collection	Molecular Devices SoftMax Pro (version 7.0.3)
Data analysis	Microsoft Excel (16.0), ApE Plasmid Editor (version 2.0.51), Graphpad Prism 9.3.1

For manuscripts utilizing custom algorithms or software that are central to the research but not yet described in published literature, software must be made available to editors and reviewers. We strongly encourage code deposition in a community repository (e.g. GitHub). See the Nature Portfolio guidelines for submitting code & software for further information.

Data

Policy information about availability of data

All manuscripts must include a data availability statement. This statement should provide the following information, where applicable:

- Accession codes, unique identifiers, or web links for publicly available datasets
- A description of any restrictions on data availability
- For clinical datasets or third party data, please ensure that the statement adheres to our policy

The authors declare that all data supporting the findings of this study are available within the paper and its supplementary information. The analyzed data and source data will be made available in Supplementary Data Files and Source Data. Any other information can be made available from the corresponding author upon reasonable request. The sequences of the following plasmids are provided in GenBank and in the source data files with respective accession numbers: pBH001-pBH002 (ON060706-ON060707), pBH101-pBH120 (ON060708-ON060727), pBH201-pBH212 (ON060728-ON060739), pBH301-pBH306 (ON060740-ON060745), pBH501-pBH513 (ON060746-ON060758).

Field-specific reporting

Please select the one below that is the best fit for your research. If you are not sure, read the appropriate sections before making your selection.

☒ Life sciences ☐ Behavioural & social sciences ☐ Ecological, evolutionary & environmental sciences

For a reference copy of the document with all sections, see nature.com/documents/nr-reporting-summary-flat.pdf

Life sciences study design

All studies must disclose on these points even when the disclosure is negative.

Sample size	No statistical methods were used for sample size calculation. A sample size of n=6 or n=12 was chosen for luminescence assays based on previous works (Rondon et al., 2019), (Groseclose et al., 2020). For luminescence assays, n=6 biological replicates were performed per day, with certain assays repeated on a second day to provide an internal consistency check. Co-culture assays were performed with n=3 samples due to their enhanced technical difficulty. A sample size of 3 was chosen based on similar synthetic biology works (Taketani et al., 2020; Mimee et al., 2015).
Data exclusions	No data has been excluded from analyses. All data collected is shown on plots and is included in the Source Data file.
Replication	The following experiments were repeated on 2 separate days with n=6 biological replicates on each day: B. theta OR, NAND, XNOR, XOR luciferase logic gates; B. ovatus AND, NOR luciferase logic gates; B. uniformis AND, NOR, OR, NAND luciferase logic gates. This was done to confirm the reproducibility of the assay and logic gates. All experiments were faithfully reproduced within 1 s.d. of the original. The co-culture experiment in Fig. 6 was successfully replicated on a second day and this data is provided in the Source Data File. Data for all other experiments were derived from independent biological replicates taken on the same day.
Randomization	Randomization was not performed in this study as it was not relevant. Cultures were always started from single colonies derived from known strains streaked onto BHI agar plates.
Blinding	Blinding was not relevant to this study since knowledge of genotype and plasmid sequence was required for experiments to be performed under the correct conditions.

Reporting for specific materials, systems and methods

We require information from authors about some types of materials, experimental systems and methods used in many studies. Here, indicate whether each material, system or method listed is relevant to your study. If you are not sure if a list item applies to your research, read the appropriate section before selecting a response.

Materials & experimental systems

n/a	Involved in the study
☒	☐ Antibodies
☒	☐ Eukaryotic cell lines
☒	☐ Palaeontology and archaeology
☒	☐ Animals and other organisms
☒	☐ Human research participants
☒	☐ Clinical data
☒	☐ Dual use research of concern

Methods

n/a	Involved in the study
☒	☐ ChIP-seq
☒	☐ Flow cytometry
☒	☐ MRI-based neuroimaging
