# Supplementary material for: Transcriptional programming in a Bacteroides consortium
Source: Nat Commun. 2022 Jul 6;13:3901. doi: 10.1038/s41467-022-31614-8 (PMC9259675; doi:10.1038/s41467-022-31614-8)
Supplement: Supplementary file 3 — Source Data Files [file 41467_2022_31614_MOESM3_ESM.zip › SOURCE DATA/Plasmid Sequences Uploaded to Genbank.docx]

>pBH001

gtttaacggttgtggacaacaagccagggatgtaacgcactgagaagcccttagagcctctcaaagcaattttgagtgacacaggaacacttaacggctgacatgggaattcccctccaccgcggtggCCCTTGAGACGGCATCTTCCATCtacactttatgcttccggctcgtatgttgtgtggaattgtgagcggataacaatttcacacaggaaacagctatgaccatgattacgccaagcttgcatgcctgcaggtcgactctagaggatccccgggtaccgagctcgaattcactggccgtcgttttacaacgtcgtgactgggaaaaccctggcgttacccaacttaatcgccttgcagcacatccccctttcgccagctggcgtaatagcgaagaggcccgcaccgatcgcccttcccaacagttgcgcagcctgaatggcgaatggcgcctgatgcggtattttctccttacgcatctgtgcggtatttcacaccgcatatggtgcactctcagtacaatctgctctgatgccgcatagGATCTGATGCATCGTCTCACCGAtacttgtgcctgttctatttccgaaccgaccgcttgtatgaatccatcaaaattcgttttctctatgttggattccttgttgctcatattgtgatgataatttctacaaatatagtcattggtaactatctatgaaactgtttgatacttttatagttgattaaacttgttcatggcatttgccttaatatcatccgctatgtcaatgtagggtttcatagctttgtagtcgctgtgtcccgtccatttcatgaccacctgtgccgggattccgagagccagcgcattgcagatgaatgtccttcttcctgcatgggtactgagcaaagcgtatttgggtgtgacttcatcaatacgttcatttcccttgtagtaggtttcccgtacaggctcgttgatttctgccagttcgcccagctctttcaggtaatcgttcatcttctggttgctgatgacgggcagagccatgtaattctcgaaatggatgtccttgtatttgtccagtatggctttgctgtatttgttcagttcaatcgtcaggctgtcggcagtcttgactgtggttatttcgatgtggtcggacttcacatcgcttcttttcagattgcgaacatccgaataccgcaaactcgtaaagcagcagaacaggaaaacatcacgcacacgttccaggtattgcttatccttgggtatctggtagtctttcagcttgttcagttcatcccaagtcaggaagattacttttttcgaggtggttttcagtttcggtttgaacgtatcgtatgcaatgttctgatgatgtcctttcctgaagctccagcgcaggaaccatttgaggaatcccatttgcttgccgatggtgctgtttctcatatccttggtgtcacgcaggaagttgacgtattcgttcaatccaaactcgttgaaatagttgaacgttgcatcctccttgaactctttgaggtggttcctcactgctgcaaatttttcataggtggatgccgtccagttattctggttaccgcactcttttacaaactcatcgaacacctcccaaaagctgacaggggcttcttccggctgttcttcactggtgtctttcattctcatgttgaaagcttccttcaactgttgggtcgttggcatgacctcctgcacctcaaattccttgaaaatattctggatttcggcatagtatttcagcaagtccgtattgatttcggctgcactttgctttagcttgttggtacatccgttctttacccgctgcttatctacatcccatttggctacgtcaatccggtagcccgttgtaaactcgatacgttggctggcaaagatgacacgcatacggatgggtacgttctctacgattggcacaccgttctttttccggctctccaatgcaaaaatgatgttgcgcttgatattcataattgggtgcgtttgaaattctacacccaaatatacacccaattattgagatagcaaaagacatttagaaacatttacttttactctatattgtaatttacacttgattatcagtcgtttgcagtTttatgatattctgtgaaagtataagttcgagagcctgtctctccgcaaaaaacgctgaaaatcagcagattgcaaaacaaacaccctgttttacacccaagaatgtaaagtcggctgtttttgttttatttaagataatacaaccactacataataaaagagtagcgatattaaaagaatccgatgagaaaagactaatatttatctatccattcagtttgatttttcaggactttacatcgtcctgaaagtatttgttggtaccggtaccgaggacgcgtaaacatttacagttgcatgtggcctattgtttttagccgttaaatattttataactattaaatagcgatacaaattgttcgaaactaatattgtttatatcatatattctcgcatgttttaaagctttattaaattgattttttgtaaacagtttttcgtactctttgttaacccatttcattacaaaagtttcatatttttttctctctttaaatgccatttttgctggctttctttttaatacaattaatgtgctatccactttaggttttggatggaaataatacctaggaatttttgctaatatagaaatatctacctctgccattaacagcaatgctagtgatctgtttgtatctaataacattttagcaaaaccatattccactattaaataacttattgtggctgaactttcaaaaacaatttttcgaattatatttgtgcttatgttgtaaggtatgctgccaaatattttatatggattgtggctaggaaatgtaaatttcagtatatcatcatttactatttgatagttaggataatttaagagcttattacgagttacctcacataatttagaatcaatttctatcgccgttacaaaattacatctctttaccaatccagcagtaaaatgacctttccctgcacctatttcaaagatgttatctttttcatctaaacttatgcaattcattattttttctatgtgatattttgaagtaataaaattttgactatcttttatatttactttgttcattataacctctccttaatttattgcatctcttttcgaatatttatgttttttgagaaaagaacgtactcatggttcatcccgatatgcgtatcggtctgtatatcagcaactttctatgtgtttcaactacaatagtcatctattctcatctttctgagtccaccccctgcaaagcccctctttacgacataaaaattcggtcggaaaaggtatgcaaaagatgtttctctctttaagagaaactcttcgggatgcaaaaatatgaaaataactccaattcaccaaattatatagcgacttttttacaaaatgctaaaatttgttgatttccgtcaagcaattgttgagcaaaaatgtcttttacgataaaatgatacctcaatatcaactgtttagcaaaacgatatttctcttaaagagagaaacacctttttgttcaccaatccccgacttttaatcccgcggccatgattgaaaaaggaagagtatgagtattcaacatttccgtgtcgcccttattcccttttttgcggcattttgccttcctgtttttgctcacccagaaacgctggtgaaagtaaaagatgctgaagatcagttgggtgcacgagtgggttacatcgaactggatctcaacagcggtaagatccttgagagttttcgccccgaagaacgttttccaatgatgagcacttttaaagttctgctatgtggcgcggtattatcccgtattgacgccgggcaagagcaactcggtcgccgcatacactattctcagaatgacttggttgagtactcaccagtcacagaaaagcatcttacggatggcatgacagtaagagaattatgcagtgctgccataaccatgagtgataacactgcggccaacttacttctgacaacgatcggaggaccgaaggagctaaccgcttttttgcacaacatgggggatcatgtaactcgccttgatcgttgggaaccggagctgaatgaagccataccaaacgacgagcgtgacaccacgatgcctgtagcaatggcaacaacgttgcgcaaactattaactggcgaactacttactctagcttcccggcaacaattaatagactggatggaggcggataaagttgcaggaccacttctgcgctcggcccttccggctggctggtttattgctgataaatctggagccggtgagcgtgggtctcgcggtatcattgcagcactggggccagatggtaagccctcccgtatcgtagttatctacacgacggggagtcaggcaactatggatgaacgaaatagacagatcgctgagataggtgcctcactgattaagcattggtaactgtcagaccaagtttactcataacgcgtcaattcgagggggatcaattccgtgataggtgggctgcccttcctggttggcttggtttcatcagccatccgcttgccctcatctgttacgccggcggtagccggccagcctcgcagagcaggattcccgttgagcaccgccaggtgcgaataagggacagtgaagaaggaacacccgctcgcgggtgggcctacttcacctatcctgcccggctgacgccgttggatacaccaaggaaagtctacacgaaccctttggcaaaatcctgtatatcgtgcgaaaaaggatggatataccgaaaaaatcgctataatgaccccgaagcagggttatgcagcggaaaacggaattgatccggccacgatgcgtccggcgtagaggatctgaagatcagcagttcaacctgttgatagtacgtactaagctctcatgtttcacgtactaagctctcatgtttaacgtactaagctctcatgtttaacgaactaaaccctcatggctaacgtactaagctctcatggctaacgtactaagctctcatgtttcacgtactaagctctcatgtttgaacaataaaattaatataaatcagcaacttaaatagcctctaaggttttaagttttataagaaaaaaaagaatatataaggcttttaaagcttttaag

>pBH002

gatgtaacgcactgagaagcccttagagcctctcaaagcaattttgagtgacacaggaacacttaacggctgacatgggaattcccctccaccgcggtgtattaatgcggctgcCCCTTGAGACGGCATCTTCCATCtacactttatgcttccggctcgtatgttgtgtggaattgtgagcggataacaatttcacacaggaaacagctatgaccatgattacgccaagcttgcatgcctgcaggtcgactctagaggatccccgggtaccgagctcgaattcactggccgtcgttttacaacgtcgtgactgggaaaaccctggcgttacccaacttaatcgccttgcagcacatccccctttcgccagctggcgtaatagcgaagaggcccgcaccgatcgcccttcccaacagttgcgcagcctgaatggcgaatggcgcctgatgcggtattttctccttacgcatctgtgcggtatttcacaccgcatatggtgcactctcagtacaatctgctctgatgccgcatagGATCTGATGCATCGTCTCACCGAtaattgcctatcttccagtgatggaacagcatttgtgcattggctgcaacaatcagccttacttgtgcctgttctatttccgaaccgaccgcttgtatgaatGcatcaaaattcgttttctctaCgttggattccttgttgctcatattgtgatgataatttctacaaatatagtcattggtaactatctatgaaactgtttgatacttttatcagtctaatagttttacaaggtctttcttcatttcttcgtcaatatccctgtatcgtctgaaagctttgcttccctccttgtgtcccgacagtgcggaaacaaggttcgggtctttcacttttttatagatattgccgataaacgtacgtcttgccagatggctgcttgccacttcataaataggtcgtttgatttcgttgtgcgtcaacgggtctaagattgttacgatgcggtcaactccagctaatttgaatatcttttttatggcatcattgtacttttgctcggatatgaacggcaacagttttccctcatattctttgtagcgttcaaggatttctttcgctttgtcgttaagtggaacacgtaccgtaaccggattcccctctttggttttcttgggaatatattctatggcttcattgaccacatttagtttggtcattcggtacaggtcgctcaccctgcatcctatcagtgtctgaaatatgaatatatccctctgtattgccagttgtggggtggcagaaaggtctgcattaaaaatcctgtccctttcttcgagtgttatataataaggtgtaccatatgtacactcctctatcggaaacttgtcgaaaggtctgtttgtggtgcgtttgttatcgaagcaccacaggaagaatgtgcgtattcttgaaaaacagtctatcagcgtgtttttgcttctgggctgtggtgtcctcttttcgggaatggcttcataaatgctcgggtaaagttcataatactggtattcgttctgaaagaaatcccacatatcccgaagcgtgtcaggtgttaccaaatccacatcaaggataaagcccttttgtcctctctttgtagcccttacatatagttcataacgcagtaaggctcttttgacaacccggaaatttttctttcgtacttccgacaaagggtgcttgtttagaaattcatcgaatagttctccaatggtaggcttgataaccacttcctccggcaaaaaatatttttcaggatggtaaaatttatcaagtgttgtttttagccattctttgtctattgcttccttttcttgttgatataccttctcgatataggttttcaactggcgtatctcttcatttatatgggtacgcatttcttcattgcagacagctttcgtttttacacattcgtctttatcatcccatagattgggattgatggctaactgagtaggagcaacggagtctaactgccttccgtttctaaaacggacatagattgtagccatggattctgtatcatatcgtttggctgcttttttaatgataaaggttactttcatagactttcaggttgaattttactctgctgcaaatataaatattttccccagcattttccccacatctgctaaatattttgcaattcgattaaacttggattaaaatttaataggattataacatattgaaatacagtgtaattgtggcgtttttccgcatttttctttttacccacattttccccactttaattatttcaaatgcggggtctgggtacaagaaagaaagctaagtatttgatagttcaatacttagctttttcttttgcttgaattttccccacattttccccacacgtgcaaaaaatatagcagtaagtcattatttcttttggttgaacgtagagagtagcgatattaaaagaatccgatgagaaaagactaatatttatctatccattcagtttgatttttcaggactttacatcgtcctgaaagtatttgttggtaccggtaccgaggacgcgtaaacatttacaGTTGCATGTGGCCTATTGTTTaggacgcgttatctccttaacgtacgttttcgttccattggccctcaaaccccgttatatacattcatgtccatttatgtaaaaaatcctgctgaccttgtttatgtcttgtcagtcaccatttgcaaaaccatatttgaccctcaaagaggctgaatttgataagcaacttgctacatactcataataaggagctaaatagaacacgaatgggaaatactcaaatgccaaactaaagaagatattggccaaaataaacgctataccgagagagaaacttgatttttcaacttcctaaccaacagtgttgttcaaacatttctacttatttgtacttaccagttgaacctacgtttccctaataaaatgtctatggtaaaaagttaaaaaatcctcctacttttgttagatatatttttttgtgtaattttgtaatcgttatgcggcagtaataatatacatattaatacgagttaggaatcctgtagttctcatatgctacgaggaggtattaaaaggtgcgtttcgacaatgcatctattgtagtatattattgcttaatccaaatgaatattataaatttaggaattcttgctcacattgatgcaggaaaaacttccgtaaccgagaatctgctgtttgccagtggagcaacggaaaagtgcggctgtgtggataatggtgacaccataacggactctatggatatagagaaacgtagaggaattactgttcgggcttctacgacatctattatctggaatggtgtgaaatgcaatatcattgacactccgggacacatggattttattgcggaagtggagcggacattcaaaatgcttgatggagcagtcctcatcttatccgcaaaggaaggcatacaagcgcagacaaagttgctgttcaatactttacagaagctgcaaatcccgacaattatatttatcaataagattgaccgagccggtgtgaatttggagcgtttgtatctggatataaaagcaaatctgtctcaagatgtcctgtttatgcaaaatgttgtcgatggatcggtttatccggtttgctcccaaacatatataaaggaagaatacaaagaatttgtatgcaaccatgacgacaatatattagaacgatatttggcggatagcgaaatttcaccggctgattattggaatacgataatcgctcttgtggcaaaagccaaagtctatccggtgctacatggatcagcaatgttcaatatcggtatcaatgagttgttggacgccatcacttcttttatacttcctccggcatcggtctcaaacagactttcatcttatctttataagatagagcatgaccccaaaggacataaaagaagttttctaaaaataattgacggaagtctgagacttcgagacgttgtaagaatcaacgattcggaaaaattcatcaagattaaaaatctaaaaactatcaatcagggcagagagataaatgttgatgaagtgggcgccaatgatatcgcgattgtagaggatatggatgattttcgaatcggaaattatttaggtgctgaaccttgtttgattcaaggattatcgcatcagcatcccgctctcaaatcctccgtccggccagacaggcccgaagagagaagcaaggtgatatccgctctgaatacattgtggattgaagacccgtctttgtccttttccataaactcatatagtgatgaattggaaatctcgttatatggtttaacccaaaaggaaatcatacagacattgctggaagaacgattttccgtaaaggtccattttgatgagatcaagactatatacaaagaacgacctgtaaaaaaggtcaataagattattcagatcgaagtgccgcccaacccttattgggccacaatagggctgactcttgaacccttaccgttagggacagggttgcaaatcgaaagtgacatctcctatggttatctgaaccattcttttcaaaatgccgtttttgaagggattcgtatgtcttgccaatccgggttacatggatgggaagtgactgatctgaaagtaacttttactcaagccgagtattatagcccggtaagtacacctgctgatttcagacagctgaccccttatgtcttcaggctggccttgcaacagtcaggtgtggacattctcgaaccgatgctctattttgagttgcagataccccaagcggcaagttccaaagctattacagatttgcaaaaaatgatgtctgagattgaagacatcagttgcaataatgagtggtgtcatattaaagggaaagttccattaaatacaagtaaagactatgcatcagaagtaagttcatacactaagggcttaggcatttttatggttaagccatgcgggtatcaaataacaaaaggcggttattctgataatatccgcatgaacgaaaaagataaacttttattcatgttccaaaaatcaatgtcatcaaaataatggtataacctctccttaatttattgcatctcttttcgaatatttatgttttttgagaaaagaacgtactcatggttcatcccgatatgcgtatcggtctgtatatcagcaactttctatgtgtttcaactacaatagtcatctattctcatctttctgagtccaccccctgcaaagcccctctttacgacataaaaattcggtcggaaaaggtatgcaaaagatgtttctctctttaagagaaactcttcgggatgcaaaaatatgaaaataactccaattcaccaaattatatagcgacttttttacaaaatgctaaaatttgttgatttccgtcaagcaattgttgagcaaaaatgtcttttacgataaaatgatacctcaatatcaactgtttagcaaaacgatatttctcttaaagagagaaacacctttttgttcaccaatccccgacttttaatcccgcggccatgattgaaaaaggaagagtatgagtattcaacatttccgtgtcgcccttattcccttttttgcggcattttgccttcctgtttttgctcacccagaaacgctggtgaaagtaaaagatgctgaagatcagttgggtgcacgagtgggttacatcgaactggatctcaacagcggtaagatccttgagagttttcgccccgaagaacgttttccaatgatgagcacttttaaagttctgctatgtggcgcggtattatcccgtattgacgccgggcaagagcaactcggtcgccgcatacactattctcagaatgacttggttgagtactcaccagtcacagaaaagcatcttacggatggcatgacagtaagagaattatgcagtgctgccataaccatgagtgataacactgcggccaacttacttctgacaacgatcggaggaccgaaggagctaaccgcttttttgcacaacatgggggatcatgtaactcgccttgatcgttgggaaccggagctgaatgaagccataccaaacgacgagcgtgacaccacgatgcctgtagcaatggcaacaacgttgcgcaaactattaactggcgaactacttactctagcttcccggcaacaattaatagactggatggaggcggataaagttgcaggaccacttctgcgctcggcccttccggctggctggtttattgctgataaatctggagccggtgagcgtgggtcacgcggtatcattgcagcactggggccagatggtaagccctcccgtatcgtagttatctacacgacggggagtcaggcaactatggatgaacgaaatagacagatcgctgagataggtgcctcactgattaagcattggtaactgtcagaccaagtttactcataacgcgtcaattcgagggggatcaattccgtgataggtgggctgcccttcctggttggcttggtttcatcagccatccgcttgccctcatctgttacgccggcggtagccggccagcctcgcagagcaggattcccgttgagcaccgccaggtgcgaataagggacagtgaagaaggaacacccgctcgcgggtgggcctacttcacctatcctgcccggctgacgccgttggatacaccaaggaaagtctacacgaaccctttggcaaaatcctgtatatcgtgcgaaaaaggatggatataccgaaaaaatcgctataatgaccccgaagcagggttatgcagcggaaaacggaattgatccggccacgatgcgtccggcgtagaggatctgaagatcagcagttcaacctgttgatagtacgtactaagctctcatgtttcacgtactaagctctcatgtttaacgtactaagctctcatgtttaacgaactaaaccctcatggctaacgtactaagctctcatggctaacgtactaagctctcatgtttcacgtactaagctctcatgtttgaacaataaaattaatataaatcagcaacttaaatagcctctaaggttttaagttttataagaaaaaaaagaatatataaggcttttaaagcttttaaggtttaacggttgtggacaacaagccagg

>pBH101

gtttaacggttgtggacaacaagccagggatgtaacgcactgagaagcccttagagcctctcaaagcaattttgagtgacacaggaacacttaacggctgacatgggaattcccctccaccgcggtggtacaaagaaaattcgacaaactgttatttttctatctatttatttgAATTGTGAGCGGATAACAATTacctttgtcggcAATTGTGAGCGGATAACAATTaaataaagatattctcgtcaaacaaatataaataatataaacatggtttttactctggaagattttgttggcgattggcgtcagaccgcgggttataatttggatcaagtcctggaacagggtggcgtaagctctctgttccagaacctgggtgtgagcgtgacgccgattcagcgcatcgttctgtccggcgagaacggtctgaaaattgatattcatgtgatcatcccgtacgaaggcctgagcggtgaccaaatgggtcaaatcgagaaaatctttaaagtcgtctacccagttgacgatcaccacttcaaggttatcttgcattacggtacgctggtgattgatggtgtgaccccgaatatgattgactatttcggccgtccgtatgaaggcattgccgtttttgacggtaaaaagatcaccgtcaccggtaccctgtggaatggcaataagattattgacgagcgtctgattaacccggacggcagcctgctgttccgcgtgaccatcaacggtgtcacgggttggcgtctgtgcgagcgcatcctggcataatgaactgcacttgctttgataattaatgataaacaatctaaaagcactctaatcgttatcggagtgcttttagattactaatcaaattgcttctactaattgcctatcttccagtgatggaacagcatttgtgcattggctgcaacaatcagccttgatctggaagaagcaatgaaagctgctgttaagtctccgaatcaggtattgttcctgacaggtgtattcccatccggtaaacgcggatactttgcagttgatctgactcaggaataaattataaattaaggtaagaagattgtaggataagctaatgaaatagaaaaaggatgccgtcacacaacttgtcggcattcttttttgttttattagttgaaaatatagtgaaaaagttgcctaaatatgtatgttaacaaattatttgtcgtaactttgcactccaaatctgtttttaacatatggcactagtgAAACCAGTAACGTTATACGATGTCGCAGAGTATGCCGGTGTCTCTTATCAGACCGTTTCCCGCGTGGTGAACCAGGCCAGCCACGTTTCTGCGAAAACGCGGGAAAAAGTGGAAGCGGCGATGGCGGAGCTGAATTACATTCCCAACCGCGTGGCACAACAACTGGCGGGCAAACAGTCGTTGCTGATTGGCGTTGCCACCTCCAGTCTGGCCCTGCACGCGCCGTCGCAAATTGTCGCGGCGATTAAATCTCGCGCCGATCAACTGGGTGCCAGCGTGGTGGTGTCGATGGTAGAACGAAGCGGCGTCGAAGCCTGTAAAACGGCGGTGCACAATCTTCTCGCGCAACGCGTCAGTGGGCTGATCATTAACTATCCGCTGGATGACCAGGATGCCATTGCTGTGGAAGCTGCCTGCACTAATGTTCCGGCGTTATTTCTTGATGTCTCTGACCAGACACCCATCAACAGTATTATTTTCTCCCATGAAGACGGTACGCGACTGGGCGTGGAGCATCTGGTCGCATTGGGTCACCAGCAAATCGCGCTGTTAGCGGGCCCATTAAGTTCTGTCTCGGCGCGTCTGCGTCTGGCTGGCTGGCATAAATATCTCACTCGCAATCAAATTCAGCCGATAGCGGAACGGGAAGGCGACTGGAGTGCCATGTCCGGTTTTCAACAAACCATGCAAATGCTGAATGAGGGCATCGTTCCCACTGCGATGCTGGTTGCCAACGATCAGATGGCGCTGGGCGCAATGCGCGCCATTACCGAGTCCGGGCTGCGCGTTGGTGCGGATATCTCGGTAGTGGGATACGACGATACCGAAGACAGCTCATGTTATATCCCGCCGTTAACCACCATCAAACAGGATTTTCGCCTGCTGGGGCAAACCAGCGTGGACCGCTTGCTGCAACTCTCTCAGGGCCAGGCGGTGAAGGGCAATCAGCTGTTGCCCGTCTCACTGGTGAAAAGAAAAACCACCCTGGCGCCCAATACGCAAACCGCCTCTCCCCGCGCGTTGGCCGATTCATTAATGCAGCTGGCACGACAGGTTTCCCGACTGGAAAGCGGGCAGtgagctttcctcggtaccaaattccagaaaagaggcctcccgaaaggggggccttttttcgttttggtcctacttgtgcctgttctatttccgaaccgaccgcttgtatgaatccatcaaaattcgttttctctatgttggattccttgttgctcatattgtgatgataatttctacaaatatagtcattggtaactatctatgaaactgtttgatacttttatagttgattaaacttgttcatggcatttgccttaatatcatccgctatgtcaatgtagggtttcatagctttgtagtcgctgtgtcccgtccatttcatgaccacctgtgccgggattccgagagccagcgcattgcagatgaatgtccttcttcctgcatgggtactgagcaaagcgtatttgggtgtgacttcatcaatacgttcatttcccttgtagtaggtttcccgtacaggctcgttgatttctgccagttcgcccagctctttcaggtaatcgttcatcttctggttgctgatgacgggcagagccatgtaattctcgaaatggatgtccttgtatttgtccagtatggctttgctgtatttgttcagttcaatcgtcaggctgtcggcagtcttgactgtggttatttcgatgtggtcggacttcacatcgcttcttttcagattgcgaacatccgaataccgcaaactcgtaaagcagcagaacaggaaaacatcacgcacacgttccaggtattgcttatccttgggtatctggtagtctttcagcttgttcagttcatcccaagtcaggaagattacttttttcgaggtggttttcagtttcggtttgaacgtatcgtatgcaatgttctgatgatgtcctttcttgaagctccagcgcaggaaccatttgaggaatcccatttgcttgccgatggtgctgtttctcatatccttggtgtcacgcaggaagttgacgtattcgttcaatccaaactcgttgaaatagttgaacgttgcatcctccttgaactctttgaggtggttcctcactgctgcaaatttttcataggtggatgccgtccagttattctggttaccgcactcttttacaaactcatcgaacacctcccaaaagctgacaggggcttcttccggctgttcttcgctggtgtctttcattctcatgttgaaagcttccttcaactgttgggtcgttggcatgacctcctgcacctcaaattccttgaaaatattctggatttcggcatagtatttcagcaagtccgtattgatttcggctgcactttgctttagcttgttggtacatccgctctttacccgctgcttatctgcatcccatttggctacgtcaatccggtagcccgttgtaaactcgatgcgttggctggcaaagatgacacgcatacggatgggtacgttctctacgattggcacaccgttctttttccggctctccaatgcaaaaatgatgttgcgcttgatattcataattgggtgcgtttgaaattctacacccaaatatacacccaattattgagatagcaaaagacatttagaaacatttacttttactctatattgtaatttacacttgattatcagtcgtttgcagtcttatgatattctgtgaaagtataagttcgagagcctgtctctccgcaaaaaacgctgaaaatcagcagattgcaaaacaaacaccctgttttacacccaagaatgtaaagtcggctgtttttgttttatttaagataatacaaccactacataataaaagagtagcgatattaaaagaatccgatgagaaaagactaatatttatctatccattcagtttgatttttcaggactttacatcgtcctgaaagtatttgttggtaccggtaccgaggacgcgtaaacatttacagttgcatgtggcctattgtttttagccgttaaatattttataactattaaatagcgatacaaattgttcgaaactaatattgtttatatcatatattctcgcatgttttaaagctttattaaattgattttttgtaaacagtttttcgtactctttgttaacccatttcattacaaaagtttcatatttttttctctctttaaatgccatttttgctggctttctttttaatacaattaatgtgctatccactttaggttttggatggaaataatacctaggaatttttgctaatatagaaatatctacctctgccattaacagcaatgctagtgatctgtttgtatctaataacattttagcaaaaccatattccactattaaataacttattgtggctgaactttcaaaaacaatttttcgaattatatttgtgcttatgttgtaaggtatgctgccaaatattttatatggattgtggctaggaaatgtaaatttcagtatatcatcatttactatttgatagttaggataatttaagagcttattacgagttacctcacataatttagaatcaatttctatcgccgttacaaaattacatctctttaccaatccagcagtaaaatgacctttccctgcacctatttcaaagatgttatctttttcatctaaacttatgcaattcattattttttctatgtgatattttgaagtaataaaattttgactatcttttatatttactttgttcattataacctctccttaatttattgcatctcttttcgaatatttatgttttttgagaaaagaacgtactcatggttcatcccgatatgcgtatcggtctgtatatcagcaactttctatgtgtttcaactacaatagtcatctattctcatctttctgagtccaccccctgcaaagcccctctttacgacataaaaattcggtcggaaaaggtatgcaaaagatgtttctctctttaagagaaactcttcgggatgcaaaaatatgaaaataactccaattcaccaaattatatagcgacttttttacaaaatgctaaaatttgttgatttccgtcaagcaattgttgagcaaaaatgtcttttacgataaaatgatacctcaatatcaactgtttagcaaaacgatatttctcttaaagagagaaacacctttttgttcaccaatccccgacttttaatcccgcggccatgattgaaaaaggaagagtatgagtattcaacatttccgtgtcgcccttattcccttttttgcggcattttgccttcctgtttttgctcacccagaaacgctggtgaaagtaaaagatgctgaagatcagttgggtgcacgagtgggttacatcgaactggatctcaacagcggtaagatccttgagagttttcgccccgaagaacgttttccaatgatgagcacttttaaagttctgctatgtggcgcggtattatcccgtattgacgccgggcaagagcaactcggtcgccgcatacactattctcagaatgacttggttgagtactcaccagtcacagaaaagcatcttacggatggcatgacagtaagagaattatgcagtgctgccataaccatgagtgataacactgcggccaacttacttctgacaacgatcggaggaccgaaggagctaaccgcttttttgcacaacatgggggatcatgtaactcgccttgatcgttgggaaccggagctgaatgaagccataccaaacgacgagcgtgacaccacgatgcctgtagcaatggcaacaacgttgcgcaaactattaactggcgaactacttactctagcttcccggcaacaattaatagactggatggaggcggataaagttgcaggaccacttctgcgctcggcccttccggctggctggtttattgctgataaatctggagccggtgagcgtgggtctcgcggtatcattgcagcactggggccagatggtaagccctcccgtatcgtagttatctacacgacggggagtcaggcaactatggatgaacgaaatagacagatcgctgagataggtgcctcactgattaagcattggtaactgtcagaccaagtttactcataacgcgtcaattcgagggggatcaattccgtgataggtgggctgcccttcctggttggcttggtttcatcagccatccgcttgccctcatctgttacgccggcggtagccggccagcctcgcagagcaggattcccgttgagcaccgccaggtgcgaataagggacagtgaagaaggaacacccgctcgcgggtgggcctacttcacctatcctgcccggctgacgccgttggatacaccaaggaaagtctacacgaaccctttggcaaaatcctgtatatcgtgcgaaaaaggatggatataccgaaaaaatcgctataatgaccccgaagcagggttatgcagcggaaaacggaattgatccggccacgatgcgtccggcgtagaggatctgaagatcagcagttcaacctgttgatagtacgtactaagctctcatgtttcacgtactaagctctcatgtttaacgtactaagctctcatgtttaacgaactaaaccctcatggctaacgtactaagctctcatggctaacgtactaagctctcatgtttcacgtactaagctctcatgtttgaacaataaaattaatataaatcagcaacttaaatagcctctaaggttttaagttttataagaaaaaaaagaatatataaggcttttaaagcttttaag

>pBH102

gtttaacggttgtggacaacaagccagggatgtaacgcactgagaagcccttagagcctctcaaagcaattttgagtgacacaggaacacttaacggctgacatgggaattcccctccaccgcggtggtacaaagaaaattcgacaaactgttatttttctatctatttatttgaattTTAAGCGCTTAAaatttacctttgtcggcaattTTAAGCGCTTAAaattaaataaagatattctcgtcaaacaaatataaataatataaacatggtttttactctggaagattttgttggcgattggcgtcagaccgcgggttataatttggatcaagtcctggaacagggtggcgtaagctctctgttccagaacctgggtgtgagcgtgacgccgattcagcgcatcgttctgtccggcgagaacggtctgaaaattgatattcatgtgatcatcccgtacgaaggcctgagcggtgaccaaatgggtcaaatcgagaaaatctttaaagtcgtctacccagttgacgatcaccacttcaaggttatcttgcattacggtacgctggtgattgatggtgtgaccccgaatatgattgactatttcggccgtccgtatgaaggcattgccgtttttgacggtaaaaagatcaccgtcaccggtaccctgtggaatggcaataagattattgacgagcgtctgattaacccggacggcagcctgctgttccgcgtgaccatcaacggtgtcacgggttggcgtctgtgcgagcgcatcctggcataatgaactgcacttgctttgataattaatgataaacaatctaaaagcactctaatcgttatcggagtgcttttagattactaatcaaattgcttctactaattgcctatcttccagtgatggaacagcatttgtgcattggctgcaacaatcagccttgatctggaagaagcaatgaaagctgctgttaagtctccgaatcaggtattgttcctgacaggtgtattcccatccggtaaacgcggatactttgcagttgatctgactcaggaataaattataaattaaggtaagaagattgtaggataagctaatgaaatagaaaaaggatgccgtcacacaacttgtcggcattcttttttgttttattagttgaaaatatagtgaaaaagttgcctaaatatgtatgttaacaaattatttgtcgtaactttgcactccaaatctgtttttaacatatggcactagtgAAACCAGTAACGTTATACGATGTCGCAGAGTATGCCGGTGTCTCTACCGCGACCGTTTCCAACGTGGTGAACCAGGCCAGCCACGTTTCTGCGAAAACGCGGGAAAAAGTGGAAGCGGCGATGGCGGAGCTGAATTACATTCCCAACCGCGTGGCACAACAACTGGCGGGCAAACAGTCGTTGCTGATTGGCGTTGCCACCTCCAGTCTGGCCCTGCACGCGCCGTCGCAAATTGTCGCGGCGATTAAATCTCGCGCCGATCAACTGGGTGCCAGCGTGGTGGTGTCGATGGTAGAACGAAGCGGCGTCGAAGCCTGTAAAACGGCGGTGCACAATCTTCTCGCGCAACGCGTCAGTGGGCTGATCATTAACTATCCGCTGGATGACCAGGATGCCATTGCTGTGGAAGCTGCCTGCACTAATGTTCCGGCGTTATTTCTTGATGTCTCTGACCAGACACCCATCAACAGTATTATTTTCTCCCATGAAGACGGTACGCGACTGGGCGTGGAGCATCTGGTCGCATTGGGTCACCAGCAAATCGCGCTGTTAGCGGGCCCATTAAGTTCTGTCTCGGCGCGTCTGCGTCTGGCTGGCTGGCATAAATATCTCACTCGCAATCAAATTCAGCCGATAGCGGAACGGGAAGGCGACTGGAGTGCCATGTCCGGTTTTCAACAAACCATGCAAATGCTGAATGAGGGCATCGTTCCCACTGCGATGCTGGTTGCCAACGATCAGATGGCGCTGGGCGCAATGCGCGCCATTACCGAGTCCGGGCTGCGCGTTGGTGCGGATATCTCGGTAGTGGGATACGACGATACCGAAGACAGCTCATGTTATATCCCGCCGTTAACCACCATCAAACAGGATTTTCGCCTGCTGGGGCAAACCAGCGTGGACCGCTTGCTGCAACTCTCTCAGGGCCAGGCGGTGAAGGGCAATCAGCTGTTGCCCGTCTCACTGGTGAAAAGAAAAACCACCCTGGCGCCCAATACGCAAACCGCCTCTCCCCGCGCGTTGGCCGATTCATTAATGCAGCTGGCACGACAGGTTTCCCGACTGGAAAGCGGGCAGtgagctttcctcggtaccaaattccagaaaagaggcctcccgaaaggggggccttttttcgttttggtcctacttgtgcctgttctatttccgaaccgaccgcttgtatgaatccatcaaaattcgttttctctatgttggattccttgttgctcatattgtgatgataatttctacaaatatagtcattggtaactatctatgaaactgtttgatacttttatagttgattaaacttgttcatggcatttgccttaatatcatccgctatgtcaatgtagggtttcatagctttgtagtcgctgtgtcccgtccatttcatgaccacctgtgccgggattccgagagccagcgcattgcagatgaatgtccttcttcctgcatgggtactgagcaaagcgtatttgggtgtgacttcatcaatacgttcatttcccttgtagtaggtttcccgtacaggctcgttgatttctgccagttcgcccagctctttcaggtaatcgttcatcttctggttgctgatgacgggcagagccatgtaattctcgaaatggatgtccttgtatttgtccagtatggctttgctgtatttgttcagttcaatcgtcaggctgtcggcagtcttgactgtggttatttcgatgtggtcggacttcacatcgcttcttttcagattgcgaacatccgaataccgcaaactcgtaaagcagcagaacaggaaaacatcacgcacacgttccaggtattgcttatccttgggtatctggtagtctttcagcttgttcagttcatcccaagtcaggaagattacttttttcgaggtggttttcagtttcggtttgaacgtatcgtatgcaatgttctgatgatgtcctttcttgaagctccagcgcaggaaccatttgaggaatcccatttgcttgccgatggtgctgtttctcatatccttggtgtcacgcaggaagttgacgtattcgttcaatccaaactcgttgaaatagttgaacgttgcatcctccttgaactctttgaggtggttcctcactgctgcaaatttttcataggtggatgccgtccagttattctggttaccgcactcttttacaaactcatcgaacacctcccaaaagctgacaggggcttcttccggctgttcttcgctggtgtctttcattctcatgttgaaagcttccttcaactgttgggtcgttggcatgacctcctgcacctcaaattccttgaaaatattctggatttcggcatagtatttcagcaagtccgtattgatttcggctgcactttgctttagcttgttggtacatccgctctttacccgctgcttatctgcatcccatttggctacgtcaatccggtagcccgttgtaaactcgatgcgttggctggcaaagatgacacgcatacggatgggtacgttctctacgattggcacaccgttctttttccggctctccaatgcaaaaatgatgttgcgcttgatattcataattgggtgcgtttgaaattctacacccaaatatacacccaattattgagatagcaaaagacatttagaaacatttacttttactctatattgtaatttacacttgattatcagtcgtttgcagtcttatgatattctgtgaaagtataagttcgagagcctgtctctccgcaaaaaacgctgaaaatcagcagattgcaaaacaaacaccctgttttacacccaagaatgtaaagtcggctgtttttgttttatttaagataatacaaccactacataataaaagagtagcgatattaaaagaatccgatgagaaaagactaatatttatctatccattcagtttgatttttcaggactttacatcgtcctgaaagtatttgttggtaccggtaccgaggacgcgtaaacatttacagttgcatgtggcctattgtttttagccgttaaatattttataactattaaatagcgatacaaattgttcgaaactaatattgtttatatcatatattctcgcatgttttaaagctttattaaattgattttttgtaaacagtttttcgtactctttgttaacccatttcattacaaaagtttcatatttttttctctctttaaatgccatttttgctggctttctttttaatacaattaatgtgctatccactttaggttttggatggaaataatacctaggaatttttgctaatatagaaatatctacctctgccattaacagcaatgctagtgatctgtttgtatctaataacattttagcaaaaccatattccactattaaataacttattgtggctgaactttcaaaaacaatttttcgaattatatttgtgcttatgttgtaaggtatgctgccaaatattttatatggattgtggctaggaaatgtaaatttcagtatatcatcatttactatttgatagttaggataatttaagagcttattacgagttacctcacataatttagaatcaatttctatcgccgttacaaaattacatctctttaccaatccagcagtaaaatgacctttccctgcacctatttcaaagatgttatctttttcatctaaacttatgcaattcattattttttctatgtgatattttgaagtaataaaattttgactatcttttatatttactttgttcattataacctctccttaatttattgcatctcttttcgaatatttatgttttttgagaaaagaacgtactcatggttcatcccgatatgcgtatcggtctgtatatcagcaactttctatgtgtttcaactacaatagtcatctattctcatctttctgagtccaccccctgcaaagcccctctttacgacataaaaattcggtcggaaaaggtatgcaaaagatgtttctctctttaagagaaactcttcgggatgcaaaaatatgaaaataactccaattcaccaaattatatagcgacttttttacaaaatgctaaaatttgttgatttccgtcaagcaattgttgagcaaaaatgtcttttacgataaaatgatacctcaatatcaactgtttagcaaaacgatatttctcttaaagagagaaacacctttttgttcaccaatccccgacttttaatcccgcggccatgattgaaaaaggaagagtatgagtattcaacatttccgtgtcgcccttattcccttttttgcggcattttgccttcctgtttttgctcacccagaaacgctggtgaaagtaaaagatgctgaagatcagttgggtgcacgagtgggttacatcgaactggatctcaacagcggtaagatccttgagagttttcgccccgaagaacgttttccaatgatgagcacttttaaagttctgctatgtggcgcggtattatcccgtattgacgccgggcaagagcaactcggtcgccgcatacactattctcagaatgacttggttgagtactcaccagtcacagaaaagcatcttacggatggcatgacagtaagagaattatgcagtgctgccataaccatgagtgataacactgcggccaacttacttctgacaacgatcggaggaccgaaggagctaaccgcttttttgcacaacatgggggatcatgtaactcgccttgatcgttgggaaccggagctgaatgaagccataccaaacgacgagcgtgacaccacgatgcctgtagcaatggcaacaacgttgcgcaaactattaactggcgaactacttactctagcttcccggcaacaattaatagactggatggaggcggataaagttgcaggaccacttctgcgctcggcccttccggctggctggtttattgctgataaatctggagccggtgagcgtgggtctcgcggtatcattgcagcactggggccagatggtaagccctcccgtatcgtagttatctacacgacggggagtcaggcaactatggatgaacgaaatagacagatcgctgagataggtgcctcactgattaagcattggtaactgtcagaccaagtttactcataacgcgtcaattcgagggggatcaattccgtgataggtgggctgcccttcctggttggcttggtttcatcagccatccgcttgccctcatctgttacgccggcggtagccggccagcctcgcagagcaggattcccgttgagcaccgccaggtgcgaataagggacagtgaagaaggaacacccgctcgcgggtgggcctacttcacctatcctgcccggctgacgccgttggatacaccaaggaaagtctacacgaaccctttggcaaaatcctgtatatcgtgcgaaaaaggatggatataccgaaaaaatcgctataatgaccccgaagcagggttatgcagcggaaaacggaattgatccggccacgatgcgtccggcgtagaggatctgaagatcagcagttcaacctgttgatagtacgtactaagctctcatgtttcacgtactaagctctcatgtttaacgtactaagctctcatgtttaacgaactaaaccctcatggctaacgtactaagctctcatggctaacgtactaagctctcatgtttcacgtactaagctctcatgtttgaacaataaaattaatataaatcagcaacttaaatagcctctaaggttttaagttttataagaaaaaaaagaatatataaggcttttaaagcttttaag

>pBH103

gtttaacggttgtggacaacaagccagggatgtaacgcactgagaagcccttagagcctctcaaagcaattttgagtgacacaggaacacttaacggctgacatgggaattcccctccaccgcggtggtacaaagaaaattcgacaaactgttatttttctatctatttatttgAATTaggAGCGCTcctAATTtacctttgtcggcAATTaggAGCGCTcctAATTaaataaagatattctcgtcaaacaaatataaataatataaacatggtttttactctggaagattttgttggcgattggcgtcagaccgcgggttataatttggatcaagtcctggaacagggtggcgtaagctctctgttccagaacctgggtgtgagcgtgacgccgattcagcgcatcgttctgtccggcgagaacggtctgaaaattgatattcatgtgatcatcccgtacgaaggcctgagcggtgaccaaatgggtcaaatcgagaaaatctttaaagtcgtctacccagttgacgatcaccacttcaaggttatcttgcattacggtacgctggtgattgatggtgtgaccccgaatatgattgactatttcggccgtccgtatgaaggcattgccgtttttgacggtaaaaagatcaccgtcaccggtaccctgtggaatggcaataagattattgacgagcgtctgattaacccggacggcagcctgctgttccgcgtgaccatcaacggtgtcacgggttggcgtctgtgcgagcgcatcctggcataatgaactgcacttgctttgataattaatgataaacaatctaaaagcactctaatcgttatcggagtgcttttagattactaatcaaattgcttctactaattgcctatcttccagtgatggaacagcatttgtgcattggctgcaacaatcagccttgatctggaagaagcaatgaaagctgctgttaagtctccgaatcaggtattgttcctgacaggtgtattcccatccggtaaacgcggatactttgcagttgatctgactcaggaataaattataaattaaggtaagaagattgtaggataagctaatgaaatagaaaaaggatgccgtcacacaacttgtcggcattcttttttgttttattagttgaaaatatagtgaaaaagttgcctaaatatgtatgttaacaaattatttgtcgtaactttgcactccaaatctgtttttaacatatggcactagtgAAACCAGTAACGTTATACGATGTCGCAGAGTATGCCGGTGTCTCTAAAAGCACCGTTTCCCTGGTGGTGAACCAGGCCAGCCACGTTTCTGCGAAAACGCGGGAAAAAGTGGAAGCGGCGATGGCGGAGCTGAATTACATTCCCAACCGCGTGGCACAACAACTGGCGGGCAAACAGTCGTTGCTGATTGGCGTTGCCACCTCCAGTCTGGCCCTGCACGCGCCGTCGCAAATTGTCGCGGCGATTAAATCTCGCGCCGATCAACTGGGTGCCAGCGTGGTGGTGTCGATGGTAGAACGAAGCGGCGTCGAAGCCTGTAAAACGGCGGTGCACAATCTTCTCGCGCAACGCGTCAGTGGGCTGATCATTAACTATCCGCTGGATGACCAGGATGCCATTGCTGTGGAAGCTGCCTGCACTAATGTTCCGGCGTTATTTCTTGATGTCTCTGACCAGACACCCATCAACAGTATTATTTTCTCCCATGAAGACGGTACGCGACTGGGCGTGGAGCATCTGGTCGCATTGGGTCACCAGCAAATCGCGCTGTTAGCGGGCCCATTAAGTTCTGTCTCGGCGCGTCTGCGTCTGGCTGGCTGGCATAAATATCTCACTCGCAATCAAATTCAGCCGATAGCGGAACGGGAAGGCGACTGGAGTGCCATGTCCGGTTTTCAACAAACCATGCAAATGCTGAATGAGGGCATCGTTCCCACTGCGATGCTGGTTGCCAACGATCAGATGGCGCTGGGCGCAATGCGCGCCATTACCGAGTCCGGGCTGCGCGTTGGTGCGGATATCTCGGTAGTGGGATACGACGATACCGAAGACAGCTCATGTTATATCCCGCCGTTAACCACCATCAAACAGGATTTTCGCCTGCTGGGGCAAACCAGCGTGGACCGCTTGCTGCAACTCTCTCAGGGCCAGGCGGTGAAGGGCAATCAGCTGTTGCCCGTCTCACTGGTGAAAAGAAAAACCACCCTGGCGCCCAATACGCAAACCGCCTCTCCCCGCGCGTTGGCCGATTCATTAATGCAGCTGGCACGACAGGTTTCCCGACTGGAAAGCGGGCAGtgagctttcctcggtaccaaattccagaaaagaggcctcccgaaaggggggccttttttcgttttggtcctacttgtgcctgttctatttccgaaccgaccgcttgtatgaatccatcaaaattcgttttctctatgttggattccttgttgctcatattgtgatgataatttctacaaatatagtcattggtaactatctatgaaactgtttgatacttttatagttgattaaacttgttcatggcatttgccttaatatcatccgctatgtcaatgtagggtttcatagctttgtagtcgctgtgtcccgtccatttcatgaccacctgtgccgggattccgagagccagcgcattgcagatgaatgtccttcttcctgcatgggtactgagcaaagcgtatttgggtgtgacttcatcaatacgttcatttcccttgtagtaggtttcccgtacaggctcgttgatttctgccagttcgcccagctctttcaggtaatcgttcatcttctggttgctgatgacgggcagagccatgtaattctcgaaatggatgtccttgtatttgtccagtatggctttgctgtatttgttcagttcaatcgtcaggctgtcggcagtcttgactgtggttatttcgatgtggtcggacttcacatcgcttcttttcagattgcgaacatccgaataccgcaaactcgtaaagcagcagaacaggaaaacatcacgcacacgttccaggtattgcttatccttgggtatctggtagtctttcagcttgttcagttcatcccaagtcaggaagattacttttttcgaggtggttttcagtttcggtttgaacgtatcgtatgcaatgttctgatgatgtcctttcttgaagctccagcgcaggaaccatttgaggaatcccatttgcttgccgatggtgctgtttctcatatccttggtgtcacgcaggaagttgacgtattcgttcaatccaaactcgttgaaatagttgaacgttgcatcctccttgaactctttgaggtggttcctcactgctgcaaatttttcataggtggatgccgtccagttattctggttaccgcactcttttacaaactcatcgaacacctcccaaaagctgacaggggcttcttccggctgttcttcgctggtgtctttcattctcatgttgaaagcttccttcaactgttgggtcgttggcatgacctcctgcacctcaaattccttgaaaatattctggatttcggcatagtatttcagcaagtccgtattgatttcggctgcactttgctttagcttgttggtacatccgctctttacccgctgcttatctgcatcccatttggctacgtcaatccggtagcccgttgtaaactcgatgcgttggctggcaaagatgacacgcatacggatgggtacgttctctacgattggcacaccgttctttttccggctctccaatgcaaaaatgatgttgcgcttgatattcataattgggtgcgtttgaaattctacacccaaatatacacccaattattgagatagcaaaagacatttagaaacatttacttttactctatattgtaatttacacttgattatcagtcgtttgcagtcttatgatattctgtgaaagtataagttcgagagcctgtctctccgcaaaaaacgctgaaaatcagcagattgcaaaacaaacaccctgttttacacccaagaatgtaaagtcggctgtttttgttttatttaagataatacaaccactacataataaaagagtagcgatattaaaagaatccgatgagaaaagactaatatttatctatccattcagtttgatttttcaggactttacatcgtcctgaaagtatttgttggtaccggtaccgaggacgcgtaaacatttacagttgcatgtggcctattgtttttagccgttaaatattttataactattaaatagcgatacaaattgttcgaaactaatattgtttatatcatatattctcgcatgttttaaagctttattaaattgattttttgtaaacagtttttcgtactctttgttaacccatttcattacaaaagtttcatatttttttctctctttaaatgccatttttgctggctttctttttaatacaattaatgtgctatccactttaggttttggatggaaataatacctaggaatttttgctaatatagaaatatctacctctgccattaacagcaatgctagtgatctgtttgtatctaataacattttagcaaaaccatattccactattaaataacttattgtggctgaactttcaaaaacaatttttcgaattatatttgtgcttatgttgtaaggtatgctgccaaatattttatatggattgtggctaggaaatgtaaatttcagtatatcatcatttactatttgatagttaggataatttaagagcttattacgagttacctcacataatttagaatcaatttctatcgccgttacaaaattacatctctttaccaatccagcagtaaaatgacctttccctgcacctatttcaaagatgttatctttttcatctaaacttatgcaattcattattttttctatgtgatattttgaagtaataaaattttgactatcttttatatttactttgttcattataacctctccttaatttattgcatctcttttcgaatatttatgttttttgagaaaagaacgtactcatggttcatcccgatatgcgtatcggtctgtatatcagcaactttctatgtgtttcaactacaatagtcatctattctcatctttctgagtccaccccctgcaaagcccctctttacgacataaaaattcggtcggaaaaggtatgcaaaagatgtttctctctttaagagaaactcttcgggatgcaaaaatatgaaaataactccaattcaccaaattatatagcgacttttttacaaaatgctaaaatttgttgatttccgtcaagcaattgttgagcaaaaatgtcttttacgataaaatgatacctcaatatcaactgtttagcaaaacgatatttctcttaaagagagaaacacctttttgttcaccaatccccgacttttaatcccgcggccatgattgaaaaaggaagagtatgagtattcaacatttccgtgtcgcccttattcccttttttgcggcattttgccttcctgtttttgctcacccagaaacgctggtgaaagtaaaagatgctgaagatcagttgggtgcacgagtgggttacatcgaactggatctcaacagcggtaagatccttgagagttttcgccccgaagaacgttttccaatgatgagcacttttaaagttctgctatgtggcgcggtattatcccgtattgacgccgggcaagagcaactcggtcgccgcatacactattctcagaatgacttggttgagtactcaccagtcacagaaaagcatcttacggatggcatgacagtaagagaattatgcagtgctgccataaccatgagtgataacactgcggccaacttacttctgacaacgatcggaggaccgaaggagctaaccgcttttttgcacaacatgggggatcatgtaactcgccttgatcgttgggaaccggagctgaatgaagccataccaaacgacgagcgtgacaccacgatgcctgtagcaatggcaacaacgttgcgcaaactattaactggcgaactacttactctagcttcccggcaacaattaatagactggatggaggcggataaagttgcaggaccacttctgcgctcggcccttccggctggctggtttattgctgataaatctggagccggtgagcgtgggtctcgcggtatcattgcagcactggggccagatggtaagccctcccgtatcgtagttatctacacgacggggagtcaggcaactatggatgaacgaaatagacagatcgctgagataggtgcctcactgattaagcattggtaactgtcagaccaagtttactcataacgcgtcaattcgagggggatcaattccgtgataggtgggctgcccttcctggttggcttggtttcatcagccatccgcttgccctcatctgttacgccggcggtagccggccagcctcgcagagcaggattcccgttgagcaccgccaggtgcgaataagggacagtgaagaaggaacacccgctcgcgggtgggcctacttcacctatcctgcccggctgacgccgttggatacaccaaggaaagtctacacgaaccctttggcaaaatcctgtatatcgtgcgaaaaaggatggatataccgaaaaaatcgctataatgaccccgaagcagggttatgcagcggaaaacggaattgatccggccacgatgcgtccggcgtagaggatctgaagatcagcagttcaacctgttgatagtacgtactaagctctcatgtttcacgtactaagctctcatgtttaacgtactaagctctcatgtttaacgaactaaaccctcatggctaacgtactaagctctcatggctaacgtactaagctctcatgtttcacgtactaagctctcatgtttgaacaataaaattaatataaatcagcaacttaaatagcctctaaggttttaagttttataagaaaaaaaagaatatataaggcttttaaagcttttaag

>pBH104

gtttaacggttgtggacaacaagccagggatgtaacgcactgagaagcccttagagcctctcaaagcaattttgagtgacacaggaacacttaacggctgacatgggaattcccctccaccgcggtggtacaaagaaaattcgacaaactgttatttttctatctatttatttgAATTttgAGCGCTcaaAATTtacctttgtcggcAATTttgAGCGCTcaaAATTaaataaagatattctcgtcaaacaaatataaataatataaacatggtttttactctggaagattttgttggcgattggcgtcagaccgcgggttataatttggatcaagtcctggaacagggtggcgtaagctctctgttccagaacctgggtgtgagcgtgacgccgattcagcgcatcgttctgtccggcgagaacggtctgaaaattgatattcatgtgatcatcccgtacgaaggcctgagcggtgaccaaatgggtcaaatcgagaaaatctttaaagtcgtctacccagttgacgatcaccacttcaaggttatcttgcattacggtacgctggtgattgatggtgtgaccccgaatatgattgactatttcggccgtccgtatgaaggcattgccgtttttgacggtaaaaagatcaccgtcaccggtaccctgtggaatggcaataagattattgacgagcgtctgattaacccggacggcagcctgctgttccgcgtgaccatcaacggtgtcacgggttggcgtctgtgcgagcgcatcctggcataatgaactgcacttgctttgataattaatgataaacaatctaaaagcactctaatcgttatcggagtgcttttagattactaatcaaattgcttctactaattgcctatcttccagtgatggaacagcatttgtgcattggctgcaacaatcagccttgatctggaagaagcaatgaaagctgctgttaagtctccgaatcaggtattgttcctgacaggtgtattcccatccggtaaacgcggatactttgcagttgatctgactcaggaataaattataaattaaggtaagaagattgtaggataagctaatgaaatagaaaaaggatgccgtcacacaacttgtcggcattcttttttgttttattagttgaaaatatagtgaaaaagttgcctaaatatgtatgttaacaaattatttgtcgtaactttgcactccaaatctgtttttaacatatggcactagtgAAACCAGTAACGTTATACGATGTCGCAGAGTATGCCGGTGTCTCTcatcagACCGTTTCCaatGTGGTGAACCAGGCCAGCCACGTTTCTGCGAAAACGCGGGAAAAAGTGGAAGCGGCGATGGCGGAGCTGAATTACATTCCCAACCGCGTGGCACAACAACTGGCGGGCAAACAGTCGTTGCTGATTGGCGTTGCCACCTCCAGTCTGGCCCTGCACGCGCCGTCGCAAATTGTCGCGGCGATTAAATCTCGCGCCGATCAACTGGGTGCCAGCGTGGTGGTGTCGATGGTAGAACGAAGCGGCGTCGAAGCCTGTAAAACGGCGGTGCACAATCTTCTCGCGCAACGCGTCAGTGGGCTGATCATTAACTATCCGCTGGATGACCAGGATGCCATTGCTGTGGAAGCTGCCTGCACTAATGTTCCGGCGTTATTTCTTGATGTCTCTGACCAGACACCCATCAACAGTATTATTTTCTCCCATGAAGACGGTACGCGACTGGGCGTGGAGCATCTGGTCGCATTGGGTCACCAGCAAATCGCGCTGTTAGCGGGCCCATTAAGTTCTGTCTCGGCGCGTCTGCGTCTGGCTGGCTGGCATAAATATCTCACTCGCAATCAAATTCAGCCGATAGCGGAACGGGAAGGCGACTGGAGTGCCATGTCCGGTTTTCAACAAACCATGCAAATGCTGAATGAGGGCATCGTTCCCACTGCGATGCTGGTTGCCAACGATCAGATGGCGCTGGGCGCAATGCGCGCCATTACCGAGTCCGGGCTGCGCGTTGGTGCGGATATCTCGGTAGTGGGATACGACGATACCGAAGACAGCTCATGTTATATCCCGCCGTTAACCACCATCAAACAGGATTTTCGCCTGCTGGGGCAAACCAGCGTGGACCGCTTGCTGCAACTCTCTCAGGGCCAGGCGGTGAAGGGCAATCAGCTGTTGCCCGTCTCACTGGTGAAAAGAAAAACCACCCTGGCGCCCAATACGCAAACCGCCTCTCCCCGCGCGTTGGCCGATTCATTAATGCAGCTGGCACGACAGGTTTCCCGACTGGAAAGCGGGCAGtgagctttcctcggtaccaaattccagaaaagaggcctcccgaaaggggggccttttttcgttttggtcctacttgtgcctgttctatttccgaaccgaccgcttgtatgaatccatcaaaattcgttttctctatgttggattccttgttgctcatattgtgatgataatttctacaaatatagtcattggtaactatctatgaaactgtttgatacttttatagttgattaaacttgttcatggcatttgccttaatatcatccgctatgtcaatgtagggtttcatagctttgtagtcgctgtgtcccgtccatttcatgaccacctgtgccgggattccgagagccagcgcattgcagatgaatgtccttcttcctgcatgggtactgagcaaagcgtatttgggtgtgacttcatcaatacgttcatttcccttgtagtaggtttcccgtacaggctcgttgatttctgccagttcgcccagctctttcaggtaatcgttcatcttctggttgctgatgacgggcagagccatgtaattctcgaaatggatgtccttgtatttgtccagtatggctttgctgtatttgttcagttcaatcgtcaggctgtcggcagtcttgactgtggttatttcgatgtggtcggacttcacatcgcttcttttcagattgcgaacatccgaataccgcaaactcgtaaagcagcagaacaggaaaacatcacgcacacgttccaggtattgcttatccttgggtatctggtagtctttcagcttgttcagttcatcccaagtcaggaagattacttttttcgaggtggttttcagtttcggtttgaacgtatcgtatgcaatgttctgatgatgtcctttcttgaagctccagcgcaggaaccatttgaggaatcccatttgcttgccgatggtgctgtttctcatatccttggtgtcacgcaggaagttgacgtattcgttcaatccaaactcgttgaaatagttgaacgttgcatcctccttgaactctttgaggtggttcctcactgctgcaaatttttcataggtggatgccgtccagttattctggttaccgcactcttttacaaactcatcgaacacctcccaaaagctgacaggggcttcttccggctgttcttcgctggtgtctttcattctcatgttgaaagcttccttcaactgttgggtcgttggcatgacctcctgcacctcaaattccttgaaaatattctggatttcggcatagtatttcagcaagtccgtattgatttcggctgcactttgctttagcttgttggtacatccgctctttacccgctgcttatctgcatcccatttggctacgtcaatccggtagcccgttgtaaactcgatgcgttggctggcaaagatgacacgcatacggatgggtacgttctctacgattggcacaccgttctttttccggctctccaatgcaaaaatgatgttgcgcttgatattcataattgggtgcgtttgaaattctacacccaaatatacacccaattattgagatagcaaaagacatttagaaacatttacttttactctatattgtaatttacacttgattatcagtcgtttgcagtcttatgatattctgtgaaagtataagttcgagagcctgtctctccgcaaaaaacgctgaaaatcagcagattgcaaaacaaacaccctgttttacacccaagaatgtaaagtcggctgtttttgttttatttaagataatacaaccactacataataaaagagtagcgatattaaaagaatccgatgagaaaagactaatatttatctatccattcagtttgatttttcaggactttacatcgtcctgaaagtatttgttggtaccggtaccgaggacgcgtaaacatttacagttgcatgtggcctattgtttttagccgttaaatattttataactattaaatagcgatacaaattgttcgaaactaatattgtttatatcatatattctcgcatgttttaaagctttattaaattgattttttgtaaacagtttttcgtactctttgttaacccatttcattacaaaagtttcatatttttttctctctttaaatgccatttttgctggctttctttttaatacaattaatgtgctatccactttaggttttggatggaaataatacctaggaatttttgctaatatagaaatatctacctctgccattaacagcaatgctagtgatctgtttgtatctaataacattttagcaaaaccatattccactattaaataacttattgtggctgaactttcaaaaacaatttttcgaattatatttgtgcttatgttgtaaggtatgctgccaaatattttatatggattgtggctaggaaatgtaaatttcagtatatcatcatttactatttgatagttaggataatttaagagcttattacgagttacctcacataatttagaatcaatttctatcgccgttacaaaattacatctctttaccaatccagcagtaaaatgacctttccctgcacctatttcaaagatgttatctttttcatctaaacttatgcaattcattattttttctatgtgatattttgaagtaataaaattttgactatcttttatatttactttgttcattataacctctccttaatttattgcatctcttttcgaatatttatgttttttgagaaaagaacgtactcatggttcatcccgatatgcgtatcggtctgtatatcagcaactttctatgtgtttcaactacaatagtcatctattctcatctttctgagtccaccccctgcaaagcccctctttacgacataaaaattcggtcggaaaaggtatgcaaaagatgtttctctctttaagagaaactcttcgggatgcaaaaatatgaaaataactccaattcaccaaattatatagcgacttttttacaaaatgctaaaatttgttgatttccgtcaagcaattgttgagcaaaaatgtcttttacgataaaatgatacctcaatatcaactgtttagcaaaacgatatttctcttaaagagagaaacacctttttgttcaccaatccccgacttttaatcccgcggccatgattgaaaaaggaagagtatgagtattcaacatttccgtgtcgcccttattcccttttttgcggcattttgccttcctgtttttgctcacccagaaacgctggtgaaagtaaaagatgctgaagatcagttgggtgcacgagtgggttacatcgaactggatctcaacagcggtaagatccttgagagttttcgccccgaagaacgttttccaatgatgagcacttttaaagttctgctatgtggcgcggtattatcccgtattgacgccgggcaagagcaactcggtcgccgcatacactattctcagaatgacttggttgagtactcaccagtcacagaaaagcatcttacggatggcatgacagtaagagaattatgcagtgctgccataaccatgagtgataacactgcggccaacttacttctgacaacgatcggaggaccgaaggagctaaccgcttttttgcacaacatgggggatcatgtaactcgccttgatcgttgggaaccggagctgaatgaagccataccaaacgacgagcgtgacaccacgatgcctgtagcaatggcaacaacgttgcgcaaactattaactggcgaactacttactctagcttcccggcaacaattaatagactggatggaggcggataaagttgcaggaccacttctgcgctcggcccttccggctggctggtttattgctgataaatctggagccggtgagcgtgggtctcgcggtatcattgcagcactggggccagatggtaagccctcccgtatcgtagttatctacacgacggggagtcaggcaactatggatgaacgaaatagacagatcgctgagataggtgcctcactgattaagcattggtaactgtcagaccaagtttactcataacgcgtcaattcgagggggatcaattccgtgataggtgggctgcccttcctggttggcttggtttcatcagccatccgcttgccctcatctgttacgccggcggtagccggccagcctcgcagagcaggattcccgttgagcaccgccaggtgcgaataagggacagtgaagaaggaacacccgctcgcgggtgggcctacttcacctatcctgcccggctgacgccgttggatacaccaaggaaagtctacacgaaccctttggcaaaatcctgtatatcgtgcgaaaaaggatggatataccgaaaaaatcgctataatgaccccgaagcagggttatgcagcggaaaacggaattgatccggccacgatgcgtccggcgtagaggatctgaagatcagcagttcaacctgttgatagtacgtactaagctctcatgtttcacgtactaagctctcatgtttaacgtactaagctctcatgtttaacgaactaaaccctcatggctaacgtactaagctctcatggctaacgtactaagctctcatgtttcacgtactaagctctcatgtttgaacaataaaattaatataaatcagcaacttaaatagcctctaaggttttaagttttataagaaaaaaaagaatatataaggcttttaaagcttttaag

>pBH105

gtttaacggttgtggacaacaagccagggatgtaacgcactgagaagcccttagagcctctcaaagcaattttgagtgacacaggaacacttaacggctgacatgggaattcccctccaccgcggtggtacaaagaaaattcgacaaactgttatttttctatctatttatttgAATTgacAGCGCTgtcAATTtacctttgtcggcAATTgacAGCGCTgtcAATTaaataaagatattctcgtcaaacaaatataaataatataaacatggtttttactctggaagattttgttggcgattggcgtcagaccgcgggttataatttggatcaagtcctggaacagggtggcgtaagctctctgttccagaacctgggtgtgagcgtgacgccgattcagcgcatcgttctgtccggcgagaacggtctgaaaattgatattcatgtgatcatcccgtacgaaggcctgagcggtgaccaaatgggtcaaatcgagaaaatctttaaagtcgtctacccagttgacgatcaccacttcaaggttatcttgcattacggtacgctggtgattgatggtgtgaccccgaatatgattgactatttcggccgtccgtatgaaggcattgccgtttttgacggtaaaaagatcaccgtcaccggtaccctgtggaatggcaataagattattgacgagcgtctgattaacccggacggcagcctgctgttccgcgtgaccatcaacggtgtcacgggttggcgtctgtgcgagcgcatcctggcataatgaactgcacttgctttgataattaatgataaacaatctaaaagcactctaatcgttatcggagtgcttttagattactaatcaaattgcttctactaattgcctatcttccagtgatggaacagcatttgtgcattggctgcaacaatcagccttgatctggaagaagcaatgaaagctgctgttaagtctccgaatcaggtattgttcctgacaggtgtattcccatccggtaaacgcggatactttgcagttgatctgactcaggaataaattataaattaaggtaagaagattgtaggataagctaatgaaatagaaaaaggatgccgtcacacaacttgtcggcattcttttttgttttattagttgaaaatatagtgaaaaagttgcctaaatatgtatgttaacaaattatttgtcgtaactttgcactccaaatctgtttttaacatatggcactagtgAAACCAGTAACGTTATACGATGTCGCAGAGTATGCCGGTGTCTCTggaaagACCGTTTCCcgcGTGGTGAACCAGGCCAGCCACGTTTCTGCGAAAACGCGGGAAAAAGTGGAAGCGGCGATGGCGGAGCTGAATTACATTCCCAACCGCGTGGCACAACAACTGGCGGGCAAACAGTCGTTGCTGATTGGCGTTGCCACCTCCAGTCTGGCCCTGCACGCGCCGTCGCAAATTGTCGCGGCGATTAAATCTCGCGCCGATCAACTGGGTGCCAGCGTGGTGGTGTCGATGGTAGAACGAAGCGGCGTCGAAGCCTGTAAAACGGCGGTGCACAATCTTCTCGCGCAACGCGTCAGTGGGCTGATCATTAACTATCCGCTGGATGACCAGGATGCCATTGCTGTGGAAGCTGCCTGCACTAATGTTCCGGCGTTATTTCTTGATGTCTCTGACCAGACACCCATCAACAGTATTATTTTCTCCCATGAAGACGGTACGCGACTGGGCGTGGAGCATCTGGTCGCATTGGGTCACCAGCAAATCGCGCTGTTAGCGGGCCCATTAAGTTCTGTCTCGGCGCGTCTGCGTCTGGCTGGCTGGCATAAATATCTCACTCGCAATCAAATTCAGCCGATAGCGGAACGGGAAGGCGACTGGAGTGCCATGTCCGGTTTTCAACAAACCATGCAAATGCTGAATGAGGGCATCGTTCCCACTGCGATGCTGGTTGCCAACGATCAGATGGCGCTGGGCGCAATGCGCGCCATTACCGAGTCCGGGCTGCGCGTTGGTGCGGATATCTCGGTAGTGGGATACGACGATACCGAAGACAGCTCATGTTATATCCCGCCGTTAACCACCATCAAACAGGATTTTCGCCTGCTGGGGCAAACCAGCGTGGACCGCTTGCTGCAACTCTCTCAGGGCCAGGCGGTGAAGGGCAATCAGCTGTTGCCCGTCTCACTGGTGAAAAGAAAAACCACCCTGGCGCCCAATACGCAAACCGCCTCTCCCCGCGCGTTGGCCGATTCATTAATGCAGCTGGCACGACAGGTTTCCCGACTGGAAAGCGGGCAGtgagctttcctcggtaccaaattccagaaaagaggcctcccgaaaggggggccttttttcgttttggtcctacttgtgcctgttctatttccgaaccgaccgcttgtatgaatccatcaaaattcgttttctctatgttggattccttgttgctcatattgtgatgataatttctacaaatatagtcattggtaactatctatgaaactgtttgatacttttatagttgattaaacttgttcatggcatttgccttaatatcatccgctatgtcaatgtagggtttcatagctttgtagtcgctgtgtcccgtccatttcatgaccacctgtgccgggattccgagagccagcgcattgcagatgaatgtccttcttcctgcatgggtactgagcaaagcgtatttgggtgtgacttcatcaatacgttcatttcccttgtagtaggtttcccgtacaggctcgttgatttctgccagttcgcccagctctttcaggtaatcgttcatcttctggttgctgatgacgggcagagccatgtaattctcgaaatggatgtccttgtatttgtccagtatggctttgctgtatttgttcagttcaatcgtcaggctgtcggcagtcttgactgtggttatttcgatgtggtcggacttcacatcgcttcttttcagattgcgaacatccgaataccgcaaactcgtaaagcagcagaacaggaaaacatcacgcacacgttccaggtattgcttatccttgggtatctggtagtctttcagcttgttcagttcatcccaagtcaggaagattacttttttcgaggtggttttcagtttcggtttgaacgtatcgtatgcaatgttctgatgatgtcctttcttgaagctccagcgcaggaaccatttgaggaatcccatttgcttgccgatggtgctgtttctcatatccttggtgtcacgcaggaagttgacgtattcgttcaatccaaactcgttgaaatagttgaacgttgcatcctccttgaactctttgaggtggttcctcactgctgcaaatttttcataggtggatgccgtccagttattctggttaccgcactcttttacaaactcatcgaacacctcccaaaagctgacaggggcttcttccggctgttcttcgctggtgtctttcattctcatgttgaaagcttccttcaactgttgggtcgttggcatgacctcctgcacctcaaattccttgaaaatattctggatttcggcatagtatttcagcaagtccgtattgatttcggctgcactttgctttagcttgttggtacatccgctctttacccgctgcttatctgcatcccatttggctacgtcaatccggtagcccgttgtaaactcgatgcgttggctggcaaagatgacacgcatacggatgggtacgttctctacgattggcacaccgttctttttccggctctccaatgcaaaaatgatgttgcgcttgatattcataattgggtgcgtttgaaattctacacccaaatatacacccaattattgagatagcaaaagacatttagaaacatttacttttactctatattgtaatttacacttgattatcagtcgtttgcagtcttatgatattctgtgaaagtataagttcgagagcctgtctctccgcaaaaaacgctgaaaatcagcagattgcaaaacaaacaccctgttttacacccaagaatgtaaagtcggctgtttttgttttatttaagataatacaaccactacataataaaagagtagcgatattaaaagaatccgatgagaaaagactaatatttatctatccattcagtttgatttttcaggactttacatcgtcctgaaagtatttgttggtaccggtaccgaggacgcgtaaacatttacagttgcatgtggcctattgtttttagccgttaaatattttataactattaaatagcgatacaaattgttcgaaactaatattgtttatatcatatattctcgcatgttttaaagctttattaaattgattttttgtaaacagtttttcgtactctttgttaacccatttcattacaaaagtttcatatttttttctctctttaaatgccatttttgctggctttctttttaatacaattaatgtgctatccactttaggttttggatggaaataatacctaggaatttttgctaatatagaaatatctacctctgccattaacagcaatgctagtgatctgtttgtatctaataacattttagcaaaaccatattccactattaaataacttattgtggctgaactttcaaaaacaatttttcgaattatatttgtgcttatgttgtaaggtatgctgccaaatattttatatggattgtggctaggaaatgtaaatttcagtatatcatcatttactatttgatagttaggataatttaagagcttattacgagttacctcacataatttagaatcaatttctatcgccgttacaaaattacatctctttaccaatccagcagtaaaatgacctttccctgcacctatttcaaagatgttatctttttcatctaaacttatgcaattcattattttttctatgtgatattttgaagtaataaaattttgactatcttttatatttactttgttcattataacctctccttaatttattgcatctcttttcgaatatttatgttttttgagaaaagaacgtactcatggttcatcccgatatgcgtatcggtctgtatatcagcaactttctatgtgtttcaactacaatagtcatctattctcatctttctgagtccaccccctgcaaagcccctctttacgacataaaaattcggtcggaaaaggtatgcaaaagatgtttctctctttaagagaaactcttcgggatgcaaaaatatgaaaataactccaattcaccaaattatatagcgacttttttacaaaatgctaaaatttgttgatttccgtcaagcaattgttgagcaaaaatgtcttttacgataaaatgatacctcaatatcaactgtttagcaaaacgatatttctcttaaagagagaaacacctttttgttcaccaatccccgacttttaatcccgcggccatgattgaaaaaggaagagtatgagtattcaacatttccgtgtcgcccttattcccttttttgcggcattttgccttcctgtttttgctcacccagaaacgctggtgaaagtaaaagatgctgaagatcagttgggtgcacgagtgggttacatcgaactggatctcaacagcggtaagatccttgagagttttcgccccgaagaacgttttccaatgatgagcacttttaaagttctgctatgtggcgcggtattatcccgtattgacgccgggcaagagcaactcggtcgccgcatacactattctcagaatgacttggttgagtactcaccagtcacagaaaagcatcttacggatggcatgacagtaagagaattatgcagtgctgccataaccatgagtgataacactgcggccaacttacttctgacaacgatcggaggaccgaaggagctaaccgcttttttgcacaacatgggggatcatgtaactcgccttgatcgttgggaaccggagctgaatgaagccataccaaacgacgagcgtgacaccacgatgcctgtagcaatggcaacaacgttgcgcaaactattaactggcgaactacttactctagcttcccggcaacaattaatagactggatggaggcggataaagttgcaggaccacttctgcgctcggcccttccggctggctggtttattgctgataaatctggagccggtgagcgtgggtctcgcggtatcattgcagcactggggccagatggtaagccctcccgtatcgtagttatctacacgacggggagtcaggcaactatggatgaacgaaatagacagatcgctgagataggtgcctcactgattaagcattggtaactgtcagaccaagtttactcataacgcgtcaattcgagggggatcaattccgtgataggtgggctgcccttcctggttggcttggtttcatcagccatccgcttgccctcatctgttacgccggcggtagccggccagcctcgcagagcaggattcccgttgagcaccgccaggtgcgaataagggacagtgaagaaggaacacccgctcgcgggtgggcctacttcacctatcctgcccggctgacgccgttggatacaccaaggaaagtctacacgaaccctttggcaaaatcctgtatatcgtgcgaaaaaggatggatataccgaaaaaatcgctataatgaccccgaagcagggttatgcagcggaaaacggaattgatccggccacgatgcgtccggcgtagaggatctgaagatcagcagttcaacctgttgatagtacgtactaagctctcatgtttcacgtactaagctctcatgtttaacgtactaagctctcatgtttaacgaactaaaccctcatggctaacgtactaagctctcatggctaacgtactaagctctcatgtttcacgtactaagctctcatgtttgaacaataaaattaatataaatcagcaacttaaatagcctctaaggttttaagttttataagaaaaaaaagaatatataaggcttttaaagcttttaag

>pBH106

gtttaacggttgtggacaacaagccagggatgtaacgcactgagaagcccttagagcctctcaaagcaattttgagtgacacaggaacacttaacggctgacatgggaattcccctccaccgcggtggtacaaagaaaattcgacaaactgttatttttctatctatttatttgAATTGTGAGCGGATAACAATTacctttgtcggcAATTGTGAGCGGATAACAATTaaataaagatattctcgtcaaacaaatataaataatataaacatggtttttactctggaagattttgttggcgattggcgtcagaccgcgggttataatttggatcaagtcctggaacagggtggcgtaagctctctgttccagaacctgggtgtgagcgtgacgccgattcagcgcatcgttctgtccggcgagaacggtctgaaaattgatattcatgtgatcatcccgtacgaaggcctgagcggtgaccaaatgggtcaaatcgagaaaatctttaaagtcgtctacccagttgacgatcaccacttcaaggttatcttgcattacggtacgctggtgattgatggtgtgaccccgaatatgattgactatttcggccgtccgtatgaaggcattgccgtttttgacggtaaaaagatcaccgtcaccggtaccctgtggaatggcaataagattattgacgagcgtctgattaacccggacggcagcctgctgttccgcgtgaccatcaacggtgtcacgggttggcgtctgtgcgagcgcatcctggcataatgaactgcacttgctttgataattaatgataaacaatctaaaagcactctaatcgttatcggagtgcttttagattactaatcaaattgcttctactaattgcctatcttccagtgatggaacagcatttgtgcattggctgcaacaatcagccttgatctggaagaagcaatgaaagctgctgttaagtctccgaatcaggtattgttcctgacaggtgtattcccatccggtaaacgcggatactttgcagttgatctgactcaggaataaattataaattaaggtaagaagattgtaggataagctaatgaaatagaaaaaggatgccgtcacacaacttgtcggcattcttttttgttttattagttgaaaatatagtgaaaaagttgcctaaatatgtatgttaacaaattatttgtcgtaactttgcactccaaatctgtttttaacatatggcactagtgAAACCAGTAACGTTATACGATGTCGCAGAGTATGCCGGTGTCTCTTATCAGACCGTTTCCCGCGTGGTGAACCAGGCCAGCCACGTTTCTGCGAAAACGCGGGAAAAAGTGGAAGCGGCGATGGCGGAGCTCAATTACATTCCCAACCGCGTGGCACAACAACTGGCGGGCAAAGCGTCGCATACCATTGGCATGTTGATCACTGCCAGTACCAATCCTTTCTATTCAGAACTGGTGCGTGGCGTTGAACGCAGCTGCTTCGAACGCGGTTATAGTCTCGTCCTTTGCAATACCGAAGGCGATGAACAGCGGATGAATCGCAATCTGGAAACGCTGATGCAAAAACGCGTTGATGGCTTGCTGTTACTGTGCACCGAAACGCATCAACCTTCGCGTGAAATCATGCAACGTTATCCGACAGTGCCTACTGTGATGATGGACTGGGCTCCGTTCGATGGCGACAGCGATCTTATTCAGGATAACTCGTTGCTGGGCGGAGACTTAGCAACGCAATATCTGATCGATAAAGGTCATACCCGTATCGCCTGTATTACCGGCCCGCTGGATAAAACTCCGGCGCGCCTGCGGTTGGAAGGTTATCGGGCGGCGATGAAACGTGCGGGTCTCAACATTCCTGATGGCTATGAAGTCACTGGTGATTTTGAATTTAACGGCGGGTTTGACGCTATGCGCCAACTGCTATCACATCCGCTGCGTCCTCAGGCCGTCTTTACCGGAAATGACGCTATGGCTGTTGGCGTTTACCAGGCGTTATATCAGGCAGAGTTACAGGTTCCGCAGGATATCGCGGTGATTGGCTATGACGATATCGAACTGGCAAGCTTTATGACGCCACCATTAACCACTATCCACCAACCGAAAGATGAACTGGGGGAGCTGGCGATTGATGTACTCATCCATCGGATAACCCAGCCGACCCTTCAGCAACAACGATTACAACTTACTCCGATTCTGATGGAACGCGGTTCGGCTTAGCTGGTGAAAAGAAAAACCACCCTGGCGCCCAATACGCAAACCGCCTCTCCCCGCGCGTTGGCCGATTCATTAATGCAGCTGGCACGACAGGTTTCCCGACTGGAAAGCGGGCAGTGAgctttcctcggtaccaaattccagaaaagaggcctcccgaaaggggggccttttttcgttttggtcctacttgtgcctgttctatttccgaaccgaccgcttgtatgaatccatcaaaattcgttttctctatgttggattccttgttgctcatattgtgatgataatttctacaaatatagtcattggtaactatctatgaaactgtttgatacttttatagttgattaaacttgttcatggcatttgccttaatatcatccgctatgtcaatgtagggtttcatagctttgtagtcgctgtgtcccgtccatttcatgaccacctgtgccgggattccgagagccagcgcattgcagatgaatgtccttcttcctgcatgggtactgagcaaagcgtatttgggtgtgacttcatcaatacgttcatttcccttgtagtaggtttcccgtacaggctcgttgatttctgccagttcgcccagctctttcaggtaatcgttcatcttctggttgctgatgacgggcagagccatgtaattctcgaaatggatgtccttgtatttgtccagtatggctttgctgtatttgttcagttcaatcgtcaggctgtcggcagtcttgactgtggttatttcgatgtggtcggacttcacatcgcttcttttcagattgcgaacatccgaataccgcaaactcgtaaagcagcagaacaggaaaacatcacgcacacgttccaggtattgcttatccttgggtatctggtagtctttcagcttgttcagttcatcccaagtcaggaagattacttttttcgaggtggttttcagtttcggtttgaacgtatcgtatgcaatgttctgatgatgtcctttcttgaagctccagcgcaggaaccatttgaggaatcccatttgcttgccgatggtgctgtttctcatatccttggtgtcacgcaggaagttgacgtattcgttcaatccaaactcgttgaaatagttgaacgttgcatcctccttgaactctttgaggtggttcctcactgctgcaaatttttcataggtggatgccgtccagttattctggttaccgcactcttttacaaactcatcgaacacctcccaaaagctgacaggggcttcttccggctgttcttcgctggtgtctttcattctcatgttgaaagcttccttcaactgttgggtcgttggcatgacctcctgcacctcaaattccttgaaaatattctggatttcggcatagtatttcagcaagtccgtattgatttcggctgcactttgctttagcttgttggtacatccgctctttacccgctgcttatctgcatcccatttggctacgtcaatccggtagcccgttgtaaactcgatgcgttggctggcaaagatgacacgcatacggatgggtacgttctctacgattggcacaccgttctttttccggctctccaatgcaaaaatgatgttgcgcttgatattcataattgggtgcgtttgaaattctacacccaaatatacacccaattattgagatagcaaaagacatttagaaacatttacttttactctatattgtaatttacacttgattatcagtcgtttgcagtcttatgatattctgtgaaagtataagttcgagagcctgtctctccgcaaaaaacgctgaaaatcagcagattgcaaaacaaacaccctgttttacacccaagaatgtaaagtcggctgtttttgttttatttaagataatacaaccactacataataaaagagtagcgatattaaaagaatccgatgagaaaagactaatatttatctatccattcagtttgatttttcaggactttacatcgtcctgaaagtatttgttggtaccggtaccgaggacgcgtaaacatttacagttgcatgtggcctattgtttttagccgttaaatattttataactattaaatagcgatacaaattgttcgaaactaatattgtttatatcatatattctcgcatgttttaaagctttattaaattgattttttgtaaacagtttttcgtactctttgttaacccatttcattacaaaagtttcatatttttttctctctttaaatgccatttttgctggctttctttttaatacaattaatgtgctatccactttaggttttggatggaaataatacctaggaatttttgctaatatagaaatatctacctctgccattaacagcaatgctagtgatctgtttgtatctaataacattttagcaaaaccatattccactattaaataacttattgtggctgaactttcaaaaacaatttttcgaattatatttgtgcttatgttgtaaggtatgctgccaaatattttatatggattgtggctaggaaatgtaaatttcagtatatcatcatttactatttgatagttaggataatttaagagcttattacgagttacctcacataatttagaatcaatttctatcgccgttacaaaattacatctctttaccaatccagcagtaaaatgacctttccctgcacctatttcaaagatgttatctttttcatctaaacttatgcaattcattattttttctatgtgatattttgaagtaataaaattttgactatcttttatatttactttgttcattataacctctccttaatttattgcatctcttttcgaatatttatgttttttgagaaaagaacgtactcatggttcatcccgatatgcgtatcggtctgtatatcagcaactttctatgtgtttcaactacaatagtcatctattctcatctttctgagtccaccccctgcaaagcccctctttacgacataaaaattcggtcggaaaaggtatgcaaaagatgtttctctctttaagagaaactcttcgggatgcaaaaatatgaaaataactccaattcaccaaattatatagcgacttttttacaaaatgctaaaatttgttgatttccgtcaagcaattgttgagcaaaaatgtcttttacgataaaatgatacctcaatatcaactgtttagcaaaacgatatttctcttaaagagagaaacacctttttgttcaccaatccccgacttttaatcccgcggccatgattgaaaaaggaagagtatgagtattcaacatttccgtgtcgcccttattcccttttttgcggcattttgccttcctgtttttgctcacccagaaacgctggtgaaagtaaaagatgctgaagatcagttgggtgcacgagtgggttacatcgaactggatctcaacagcggtaagatccttgagagttttcgccccgaagaacgttttccaatgatgagcacttttaaagttctgctatgtggcgcggtattatcccgtattgacgccgggcaagagcaactcggtcgccgcatacactattctcagaatgacttggttgagtactcaccagtcacagaaaagcatcttacggatggcatgacagtaagagaattatgcagtgctgccataaccatgagtgataacactgcggccaacttacttctgacaacgatcggaggaccgaaggagctaaccgcttttttgcacaacatgggggatcatgtaactcgccttgatcgttgggaaccggagctgaatgaagccataccaaacgacgagcgtgacaccacgatgcctgtagcaatggcaacaacgttgcgcaaactattaactggcgaactacttactctagcttcccggcaacaattaatagactggatggaggcggataaagttgcaggaccacttctgcgctcggcccttccggctggctggtttattgctgataaatctggagccggtgagcgtgggtctcgcggtatcattgcagcactggggccagatggtaagccctcccgtatcgtagttatctacacgacggggagtcaggcaactatggatgaacgaaatagacagatcgctgagataggtgcctcactgattaagcattggtaactgtcagaccaagtttactcataacgcgtcaattcgagggggatcaattccgtgataggtgggctgcccttcctggttggcttggtttcatcagccatccgcttgccctcatctgttacgccggcggtagccggccagcctcgcagagcaggattcccgttgagcaccgccaggtgcgaataagggacagtgaagaaggaacacccgctcgcgggtgggcctacttcacctatcctgcccggctgacgccgttggatacaccaaggaaagtctacacgaaccctttggcaaaatcctgtatatcgtgcgaaaaaggatggatataccgaaaaaatcgctataatgaccccgaagcagggttatgcagcggaaaacggaattgatccggccacgatgcgtccggcgtagaggatctgaagatcagcagttcaacctgttgatagtacgtactaagctctcatgtttcacgtactaagctctcatgtttaacgtactaagctctcatgtttaacgaactaaaccctcatggctaacgtactaagctctcatggctaacgtactaagctctcatgtttcacgtactaagctctcatgtttgaacaataaaattaatataaatcagcaacttaaatagcctctaaggttttaagttttataagaaaaaaaagaatatataaggcttttaaagcttttaag

>pBH107

gtttaacggttgtggacaacaagccagggatgtaacgcactgagaagcccttagagcctctcaaagcaattttgagtgacacaggaacacttaacggctgacatgggaattcccctccaccgcggtggtacaaagaaaattcgacaaactgttatttttctatctatttatttgaattTTAAGCGCTTAAaatttacctttgtcggcaattTTAAGCGCTTAAaattaaataaagatattctcgtcaaacaaatataaataatataaacatggtttttactctggaagattttgttggcgattggcgtcagaccgcgggttataatttggatcaagtcctggaacagggtggcgtaagctctctgttccagaacctgggtgtgagcgtgacgccgattcagcgcatcgttctgtccggcgagaacggtctgaaaattgatattcatgtgatcatcccgtacgaaggcctgagcggtgaccaaatgggtcaaatcgagaaaatctttaaagtcgtctacccagttgacgatcaccacttcaaggttatcttgcattacggtacgctggtgattgatggtgtgaccccgaatatgattgactatttcggccgtccgtatgaaggcattgccgtttttgacggtaaaaagatcaccgtcaccggtaccctgtggaatggcaataagattattgacgagcgtctgattaacccggacggcagcctgctgttccgcgtgaccatcaacggtgtcacgggttggcgtctgtgcgagcgcatcctggcataatgaactgcacttgctttgataattaatgataaacaatctaaaagcactctaatcgttatcggagtgcttttagattactaatcaaattgcttctactaattgcctatcttccagtgatggaacagcatttgtgcattggctgcaacaatcagccttgatctggaagaagcaatgaaagctgctgttaagtctccgaatcaggtattgttcctgacaggtgtattcccatccggtaaacgcggatactttgcagttgatctgactcaggaataaattataaattaaggtaagaagattgtaggataagctaatgaaatagaaaaaggatgccgtcacacaacttgtcggcattcttttttgttttattagttgaaaatatagtgaaaaagttgcctaaatatgtatgttaacaaattatttgtcgtaactttgcactccaaatctgtttttaacatatggcactagtgAAACCAGTAACGTTATACGATGTCGCAGAGTATGCCGGTGTCTCTACCGCGACCGTTTCCAACGTGGTGAACCAGGCCAGCCACGTTTCTGCGAAAACGCGGGAAAAAGTGGAAGCGGCGATGGCGGAGCTCAATTACATTCCCAACCGCGTGGCACAACAACTGGCGGGCAAAGCGTCGCATACCATTGGCATGTTGATCACTGCCAGTACCAATCCTTTCTATTCAGAACTGGTGCGTGGCGTTGAACGCAGCTGCTTCGAACGCGGTTATAGTCTCGTCCTTTGCAATACCGAAGGCGATGAACAGCGGATGAATCGCAATCTGGAAACGCTGATGCAAAAACGCGTTGATGGCTTGCTGTTACTGTGCACCGAAACGCATCAACCTTCGCGTGAAATCATGCAACGTTATCCGACAGTGCCTACTGTGATGATGGACTGGGCTCCGTTCGATGGCGACAGCGATCTTATTCAGGATAACTCGTTGCTGGGCGGAGACTTAGCAACGCAATATCTGATCGATAAAGGTCATACCCGTATCGCCTGTATTACCGGCCCGCTGGATAAAACTCCGGCGCGCCTGCGGTTGGAAGGTTATCGGGCGGCGATGAAACGTGCGGGTCTCAACATTCCTGATGGCTATGAAGTCACTGGTGATTTTGAATTTAACGGCGGGTTTGACGCTATGCGCCAACTGCTATCACATCCGCTGCGTCCTCAGGCCGTCTTTACCGGAAATGACGCTATGGCTGTTGGCGTTTACCAGGCGTTATATCAGGCAGAGTTACAGGTTCCGCAGGATATCGCGGTGATTGGCTATGACGATATCGAACTGGCAAGCTTTATGACGCCACCATTAACCACTATCCACCAACCGAAAGATGAACTGGGGGAGCTGGCGATTGATGTACTCATCCATCGGATAACCCAGCCGACCCTTCAGCAACAACGATTACAACTTACTCCGATTCTGATGGAACGCGGTTCGGCTTAGCTGGTGAAAAGAAAAACCACCCTGGCGCCCAATACGCAAACCGCCTCTCCCCGCGCGTTGGCCGATTCATTAATGCAGCTGGCACGACAGGTTTCCCGACTGGAAAGCGGGCAGTGAgctttcctcggtaccaaattccagaaaagaggcctcccgaaaggggggccttttttcgttttggtcctacttgtgcctgttctatttccgaaccgaccgcttgtatgaatccatcaaaattcgttttctctatgttggattccttgttgctcatattgtgatgataatttctacaaatatagtcattggtaactatctatgaaactgtttgatacttttatagttgattaaacttgttcatggcatttgccttaatatcatccgctatgtcaatgtagggtttcatagctttgtagtcgctgtgtcccgtccatttcatgaccacctgtgccgggattccgagagccagcgcattgcagatgaatgtccttcttcctgcatgggtactgagcaaagcgtatttgggtgtgacttcatcaatacgttcatttcccttgtagtaggtttcccgtacaggctcgttgatttctgccagttcgcccagctctttcaggtaatcgttcatcttctggttgctgatgacgggcagagccatgtaattctcgaaatggatgtccttgtatttgtccagtatggctttgctgtatttgttcagttcaatcgtcaggctgtcggcagtcttgactgtggttatttcgatgtggtcggacttcacatcgcttcttttcagattgcgaacatccgaataccgcaaactcgtaaagcagcagaacaggaaaacatcacgcacacgttccaggtattgcttatccttgggtatctggtagtctttcagcttgttcagttcatcccaagtcaggaagattacttttttcgaggtggttttcagtttcggtttgaacgtatcgtatgcaatgttctgatgatgtcctttcttgaagctccagcgcaggaaccatttgaggaatcccatttgcttgccgatggtgctgtttctcatatccttggtgtcacgcaggaagttgacgtattcgttcaatccaaactcgttgaaatagttgaacgttgcatcctccttgaactctttgaggtggttcctcactgctgcaaatttttcataggtggatgccgtccagttattctggttaccgcactcttttacaaactcatcgaacacctcccaaaagctgacaggggcttcttccggctgttcttcgctggtgtctttcattctcatgttgaaagcttccttcaactgttgggtcgttggcatgacctcctgcacctcaaattccttgaaaatattctggatttcggcatagtatttcagcaagtccgtattgatttcggctgcactttgctttagcttgttggtacatccgctctttacccgctgcttatctgcatcccatttggctacgtcaatccggtagcccgttgtaaactcgatgcgttggctggcaaagatgacacgcatacggatgggtacgttctctacgattggcacaccgttctttttccggctctccaatgcaaaaatgatgttgcgcttgatattcataattgggtgcgtttgaaattctacacccaaatatacacccaattattgagatagcaaaagacatttagaaacatttacttttactctatattgtaatttacacttgattatcagtcgtttgcagtcttatgatattctgtgaaagtataagttcgagagcctgtctctccgcaaaaaacgctgaaaatcagcagattgcaaaacaaacaccctgttttacacccaagaatgtaaagtcggctgtttttgttttatttaagataatacaaccactacataataaaagagtagcgatattaaaagaatccgatgagaaaagactaatatttatctatccattcagtttgatttttcaggactttacatcgtcctgaaagtatttgttggtaccggtaccgaggacgcgtaaacatttacagttgcatgtggcctattgtttttagccgttaaatattttataactattaaatagcgatacaaattgttcgaaactaatattgtttatatcatatattctcgcatgttttaaagctttattaaattgattttttgtaaacagtttttcgtactctttgttaacccatttcattacaaaagtttcatatttttttctctctttaaatgccatttttgctggctttctttttaatacaattaatgtgctatccactttaggttttggatggaaataatacctaggaatttttgctaatatagaaatatctacctctgccattaacagcaatgctagtgatctgtttgtatctaataacattttagcaaaaccatattccactattaaataacttattgtggctgaactttcaaaaacaatttttcgaattatatttgtgcttatgttgtaaggtatgctgccaaatattttatatggattgtggctaggaaatgtaaatttcagtatatcatcatttactatttgatagttaggataatttaagagcttattacgagttacctcacataatttagaatcaatttctatcgccgttacaaaattacatctctttaccaatccagcagtaaaatgacctttccctgcacctatttcaaagatgttatctttttcatctaaacttatgcaattcattattttttctatgtgatattttgaagtaataaaattttgactatcttttatatttactttgttcattataacctctccttaatttattgcatctcttttcgaatatttatgttttttgagaaaagaacgtactcatggttcatcccgatatgcgtatcggtctgtatatcagcaactttctatgtgtttcaactacaatagtcatctattctcatctttctgagtccaccccctgcaaagcccctctttacgacataaaaattcggtcggaaaaggtatgcaaaagatgtttctctctttaagagaaactcttcgggatgcaaaaatatgaaaataactccaattcaccaaattatatagcgacttttttacaaaatgctaaaatttgttgatttccgtcaagcaattgttgagcaaaaatgtcttttacgataaaatgatacctcaatatcaactgtttagcaaaacgatatttctcttaaagagagaaacacctttttgttcaccaatccccgacttttaatcccgcggccatgattgaaaaaggaagagtatgagtattcaacatttccgtgtcgcccttattcccttttttgcggcattttgccttcctgtttttgctcacccagaaacgctggtgaaagtaaaagatgctgaagatcagttgggtgcacgagtgggttacatcgaactggatctcaacagcggtaagatccttgagagttttcgccccgaagaacgttttccaatgatgagcacttttaaagttctgctatgtggcgcggtattatcccgtattgacgccgggcaagagcaactcggtcgccgcatacactattctcagaatgacttggttgagtactcaccagtcacagaaaagcatcttacggatggcatgacagtaagagaattatgcagtgctgccataaccatgagtgataacactgcggccaacttacttctgacaacgatcggaggaccgaaggagctaaccgcttttttgcacaacatgggggatcatgtaactcgccttgatcgttgggaaccggagctgaatgaagccataccaaacgacgagcgtgacaccacgatgcctgtagcaatggcaacaacgttgcgcaaactattaactggcgaactacttactctagcttcccggcaacaattaatagactggatggaggcggataaagttgcaggaccacttctgcgctcggcccttccggctggctggtttattgctgataaatctggagccggtgagcgtgggtctcgcggtatcattgcagcactggggccagatggtaagccctcccgtatcgtagttatctacacgacggggagtcaggcaactatggatgaacgaaatagacagatcgctgagataggtgcctcactgattaagcattggtaactgtcagaccaagtttactcataacgcgtcaattcgagggggatcaattccgtgataggtgggctgcccttcctggttggcttggtttcatcagccatccgcttgccctcatctgttacgccggcggtagccggccagcctcgcagagcaggattcccgttgagcaccgccaggtgcgaataagggacagtgaagaaggaacacccgctcgcgggtgggcctacttcacctatcctgcccggctgacgccgttggatacaccaaggaaagtctacacgaaccctttggcaaaatcctgtatatcgtgcgaaaaaggatggatataccgaaaaaatcgctataatgaccccgaagcagggttatgcagcggaaaacggaattgatccggccacgatgcgtccggcgtagaggatctgaagatcagcagttcaacctgttgatagtacgtactaagctctcatgtttcacgtactaagctctcatgtttaacgtactaagctctcatgtttaacgaactaaaccctcatggctaacgtactaagctctcatggctaacgtactaagctctcatgtttcacgtactaagctctcatgtttgaacaataaaattaatataaatcagcaacttaaatagcctctaaggttttaagttttataagaaaaaaaagaatatataaggcttttaaagcttttaag

>pBH108

gtttaacggttgtggacaacaagccagggatgtaacgcactgagaagcccttagagcctctcaaagcaattttgagtgacacaggaacacttaacggctgacatgggaattcccctccaccgcggtggtacaaagaaaattcgacaaactgttatttttctatctatttatttgAATTaggAGCGCTcctAATTtacctttgtcggcAATTaggAGCGCTcctAATTaaataaagatattctcgtcaaacaaatataaataatataaacatggtttttactctggaagattttgttggcgattggcgtcagaccgcgggttataatttggatcaagtcctggaacagggtggcgtaagctctctgttccagaacctgggtgtgagcgtgacgccgattcagcgcatcgttctgtccggcgagaacggtctgaaaattgatattcatgtgatcatcccgtacgaaggcctgagcggtgaccaaatgggtcaaatcgagaaaatctttaaagtcgtctacccagttgacgatcaccacttcaaggttatcttgcattacggtacgctggtgattgatggtgtgaccccgaatatgattgactatttcggccgtccgtatgaaggcattgccgtttttgacggtaaaaagatcaccgtcaccggtaccctgtggaatggcaataagattattgacgagcgtctgattaacccggacggcagcctgctgttccgcgtgaccatcaacggtgtcacgggttggcgtctgtgcgagcgcatcctggcataatgaactgcacttgctttgataattaatgataaacaatctaaaagcactctaatcgttatcggagtgcttttagattactaatcaaattgcttctactaattgcctatcttccagtgatggaacagcatttgtgcattggctgcaacaatcagccttgatctggaagaagcaatgaaagctgctgttaagtctccgaatcaggtattgttcctgacaggtgtattcccatccggtaaacgcggatactttgcagttgatctgactcaggaataaattataaattaaggtaagaagattgtaggataagctaatgaaatagaaaaaggatgccgtcacacaacttgtcggcattcttttttgttttattagttgaaaatatagtgaaaaagttgcctaaatatgtatgttaacaaattatttgtcgtaactttgcactccaaatctgtttttaacatatggcactagtgAAACCAGTAACGTTATACGATGTCGCAGAGTATGCCGGTGTCTCTAAAAGCACCGTTTCCCTGGTGGTGAACCAGGCCAGCCACGTTTCTGCGAAAACGCGGGAAAAAGTGGAAGCGGCGATGGCGGAGCTCAATTACATTCCCAACCGCGTGGCACAACAACTGGCGGGCAAAGCGTCGCATACCATTGGCATGTTGATCACTGCCAGTACCAATCCTTTCTATTCAGAACTGGTGCGTGGCGTTGAACGCAGCTGCTTCGAACGCGGTTATAGTCTCGTCCTTTGCAATACCGAAGGCGATGAACAGCGGATGAATCGCAATCTGGAAACGCTGATGCAAAAACGCGTTGATGGCTTGCTGTTACTGTGCACCGAAACGCATCAACCTTCGCGTGAAATCATGCAACGTTATCCGACAGTGCCTACTGTGATGATGGACTGGGCTCCGTTCGATGGCGACAGCGATCTTATTCAGGATAACTCGTTGCTGGGCGGAGACTTAGCAACGCAATATCTGATCGATAAAGGTCATACCCGTATCGCCTGTATTACCGGCCCGCTGGATAAAACTCCGGCGCGCCTGCGGTTGGAAGGTTATCGGGCGGCGATGAAACGTGCGGGTCTCAACATTCCTGATGGCTATGAAGTCACTGGTGATTTTGAATTTAACGGCGGGTTTGACGCTATGCGCCAACTGCTATCACATCCGCTGCGTCCTCAGGCCGTCTTTACCGGAAATGACGCTATGGCTGTTGGCGTTTACCAGGCGTTATATCAGGCAGAGTTACAGGTTCCGCAGGATATCGCGGTGATTGGCTATGACGATATCGAACTGGCAAGCTTTATGACGCCACCATTAACCACTATCCACCAACCGAAAGATGAACTGGGGGAGCTGGCGATTGATGTACTCATCCATCGGATAACCCAGCCGACCCTTCAGCAACAACGATTACAACTTACTCCGATTCTGATGGAACGCGGTTCGGCTTAGCTGGTGAAAAGAAAAACCACCCTGGCGCCCAATACGCAAACCGCCTCTCCCCGCGCGTTGGCCGATTCATTAATGCAGCTGGCACGACAGGTTTCCCGACTGGAAAGCGGGCAGTGAgctttcctcggtaccaaattccagaaaagaggcctcccgaaaggggggccttttttcgttttggtcctacttgtgcctgttctatttccgaaccgaccgcttgtatgaatccatcaaaattcgttttctctatgttggattccttgttgctcatattgtgatgataatttctacaaatatagtcattggtaactatctatgaaactgtttgatacttttatagttgattaaacttgttcatggcatttgccttaatatcatccgctatgtcaatgtagggtttcatagctttgtagtcgctgtgtcccgtccatttcatgaccacctgtgccgggattccgagagccagcgcattgcagatgaatgtccttcttcctgcatgggtactgagcaaagcgtatttgggtgtgacttcatcaatacgttcatttcccttgtagtaggtttcccgtacaggctcgttgatttctgccagttcgcccagctctttcaggtaatcgttcatcttctggttgctgatgacgggcagagccatgtaattctcgaaatggatgtccttgtatttgtccagtatggctttgctgtatttgttcagttcaatcgtcaggctgtcggcagtcttgactgtggttatttcgatgtggtcggacttcacatcgcttcttttcagattgcgaacatccgaataccgcaaactcgtaaagcagcagaacaggaaaacatcacgcacacgttccaggtattgcttatccttgggtatctggtagtctttcagcttgttcagttcatcccaagtcaggaagattacttttttcgaggtggttttcagtttcggtttgaacgtatcgtatgcaatgttctgatgatgtcctttcttgaagctccagcgcaggaaccatttgaggaatcccatttgcttgccgatggtgctgtttctcatatccttggtgtcacgcaggaagttgacgtattcgttcaatccaaactcgttgaaatagttgaacgttgcatcctccttgaactctttgaggtggttcctcactgctgcaaatttttcataggtggatgccgtccagttattctggttaccgcactcttttacaaactcatcgaacacctcccaaaagctgacaggggcttcttccggctgttcttcgctggtgtctttcattctcatgttgaaagcttccttcaactgttgggtcgttggcatgacctcctgcacctcaaattccttgaaaatattctggatttcggcatagtatttcagcaagtccgtattgatttcggctgcactttgctttagcttgttggtacatccgctctttacccgctgcttatctgcatcccatttggctacgtcaatccggtagcccgttgtaaactcgatgcgttggctggcaaagatgacacgcatacggatgggtacgttctctacgattggcacaccgttctttttccggctctccaatgcaaaaatgatgttgcgcttgatattcataattgggtgcgtttgaaattctacacccaaatatacacccaattattgagatagcaaaagacatttagaaacatttacttttactctatattgtaatttacacttgattatcagtcgtttgcagtcttatgatattctgtgaaagtataagttcgagagcctgtctctccgcaaaaaacgctgaaaatcagcagattgcaaaacaaacaccctgttttacacccaagaatgtaaagtcggctgtttttgttttatttaagataatacaaccactacataataaaagagtagcgatattaaaagaatccgatgagaaaagactaatatttatctatccattcagtttgatttttcaggactttacatcgtcctgaaagtatttgttggtaccggtaccgaggacgcgtaaacatttacagttgcatgtggcctattgtttttagccgttaaatattttataactattaaatagcgatacaaattgttcgaaactaatattgtttatatcatatattctcgcatgttttaaagctttattaaattgattttttgtaaacagtttttcgtactctttgttaacccatttcattacaaaagtttcatatttttttctctctttaaatgccatttttgctggctttctttttaatacaattaatgtgctatccactttaggttttggatggaaataatacctaggaatttttgctaatatagaaatatctacctctgccattaacagcaatgctagtgatctgtttgtatctaataacattttagcaaaaccatattccactattaaataacttattgtggctgaactttcaaaaacaatttttcgaattatatttgtgcttatgttgtaaggtatgctgccaaatattttatatggattgtggctaggaaatgtaaatttcagtatatcatcatttactatttgatagttaggataatttaagagcttattacgagttacctcacataatttagaatcaatttctatcgccgttacaaaattacatctctttaccaatccagcagtaaaatgacctttccctgcacctatttcaaagatgttatctttttcatctaaacttatgcaattcattattttttctatgtgatattttgaagtaataaaattttgactatcttttatatttactttgttcattataacctctccttaatttattgcatctcttttcgaatatttatgttttttgagaaaagaacgtactcatggttcatcccgatatgcgtatcggtctgtatatcagcaactttctatgtgtttcaactacaatagtcatctattctcatctttctgagtccaccccctgcaaagcccctctttacgacataaaaattcggtcggaaaaggtatgcaaaagatgtttctctctttaagagaaactcttcgggatgcaaaaatatgaaaataactccaattcaccaaattatatagcgacttttttacaaaatgctaaaatttgttgatttccgtcaagcaattgttgagcaaaaatgtcttttacgataaaatgatacctcaatatcaactgtttagcaaaacgatatttctcttaaagagagaaacacctttttgttcaccaatccccgacttttaatcccgcggccatgattgaaaaaggaagagtatgagtattcaacatttccgtgtcgcccttattcccttttttgcggcattttgccttcctgtttttgctcacccagaaacgctggtgaaagtaaaagatgctgaagatcagttgggtgcacgagtgggttacatcgaactggatctcaacagcggtaagatccttgagagttttcgccccgaagaacgttttccaatgatgagcacttttaaagttctgctatgtggcgcggtattatcccgtattgacgccgggcaagagcaactcggtcgccgcatacactattctcagaatgacttggttgagtactcaccagtcacagaaaagcatcttacggatggcatgacagtaagagaattatgcagtgctgccataaccatgagtgataacactgcggccaacttacttctgacaacgatcggaggaccgaaggagctaaccgcttttttgcacaacatgggggatcatgtaactcgccttgatcgttgggaaccggagctgaatgaagccataccaaacgacgagcgtgacaccacgatgcctgtagcaatggcaacaacgttgcgcaaactattaactggcgaactacttactctagcttcccggcaacaattaatagactggatggaggcggataaagttgcaggaccacttctgcgctcggcccttccggctggctggtttattgctgataaatctggagccggtgagcgtgggtctcgcggtatcattgcagcactggggccagatggtaagccctcccgtatcgtagttatctacacgacggggagtcaggcaactatggatgaacgaaatagacagatcgctgagataggtgcctcactgattaagcattggtaactgtcagaccaagtttactcataacgcgtcaattcgagggggatcaattccgtgataggtgggctgcccttcctggttggcttggtttcatcagccatccgcttgccctcatctgttacgccggcggtagccggccagcctcgcagagcaggattcccgttgagcaccgccaggtgcgaataagggacagtgaagaaggaacacccgctcgcgggtgggcctacttcacctatcctgcccggctgacgccgttggatacaccaaggaaagtctacacgaaccctttggcaaaatcctgtatatcgtgcgaaaaaggatggatataccgaaaaaatcgctataatgaccccgaagcagggttatgcagcggaaaacggaattgatccggccacgatgcgtccggcgtagaggatctgaagatcagcagttcaacctgttgatagtacgtactaagctctcatgtttcacgtactaagctctcatgtttaacgtactaagctctcatgtttaacgaactaaaccctcatggctaacgtactaagctctcatggctaacgtactaagctctcatgtttcacgtactaagctctcatgtttgaacaataaaattaatataaatcagcaacttaaatagcctctaaggttttaagttttataagaaaaaaaagaatatataaggcttttaaagcttttaag

>pBH109

gtttaacggttgtggacaacaagccagggatgtaacgcactgagaagcccttagagcctctcaaagcaattttgagtgacacaggaacacttaacggctgacatgggaattcccctccaccgcggtggtacaaagaaaattcgacaaactgttatttttctatctatttatttgAATTttgAGCGCTcaaAATTtacctttgtcggcAATTttgAGCGCTcaaAATTaaataaagatattctcgtcaaacaaatataaataatataaacatggtttttactctggaagattttgttggcgattggcgtcagaccgcgggttataatttggatcaagtcctggaacagggtggcgtaagctctctgttccagaacctgggtgtgagcgtgacgccgattcagcgcatcgttctgtccggcgagaacggtctgaaaattgatattcatgtgatcatcccgtacgaaggcctgagcggtgaccaaatgggtcaaatcgagaaaatctttaaagtcgtctacccagttgacgatcaccacttcaaggttatcttgcattacggtacgctggtgattgatggtgtgaccccgaatatgattgactatttcggccgtccgtatgaaggcattgccgtttttgacggtaaaaagatcaccgtcaccggtaccctgtggaatggcaataagattattgacgagcgtctgattaacccggacggcagcctgctgttccgcgtgaccatcaacggtgtcacgggttggcgtctgtgcgagcgcatcctggcataatgaactgcacttgctttgataattaatgataaacaatctaaaagcactctaatcgttatcggagtgcttttagattactaatcaaattgcttctactaattgcctatcttccagtgatggaacagcatttgtgcattggctgcaacaatcagccttgatctggaagaagcaatgaaagctgctgttaagtctccgaatcaggtattgttcctgacaggtgtattcccatccggtaaacgcggatactttgcagttgatctgactcaggaataaattataaattaaggtaagaagattgtaggataagctaatgaaatagaaaaaggatgccgtcacacaacttgtcggcattcttttttgttttattagttgaaaatatagtgaaaaagttgcctaaatatgtatgttaacaaattatttgtcgtaactttgcactccaaatctgtttttaacatatggcactagtgAAACCAGTAACGTTATACGATGTCGCAGAGTATGCCGGTGTCTCTcatcagACCGTTTCCaatGTGGTGAACCAGGCCAGCCACGTTTCTGCGAAAACGCGGGAAAAAGTGGAAGCGGCGATGGCGGAGCTCAATTACATTCCCAACCGCGTGGCACAACAACTGGCGGGCAAAGCGTCGCATACCATTGGCATGTTGATCACTGCCAGTACCAATCCTTTCTATTCAGAACTGGTGCGTGGCGTTGAACGCAGCTGCTTCGAACGCGGTTATAGTCTCGTCCTTTGCAATACCGAAGGCGATGAACAGCGGATGAATCGCAATCTGGAAACGCTGATGCAAAAACGCGTTGATGGCTTGCTGTTACTGTGCACCGAAACGCATCAACCTTCGCGTGAAATCATGCAACGTTATCCGACAGTGCCTACTGTGATGATGGACTGGGCTCCGTTCGATGGCGACAGCGATCTTATTCAGGATAACTCGTTGCTGGGCGGAGACTTAGCAACGCAATATCTGATCGATAAAGGTCATACCCGTATCGCCTGTATTACCGGCCCGCTGGATAAAACTCCGGCGCGCCTGCGGTTGGAAGGTTATCGGGCGGCGATGAAACGTGCGGGTCTCAACATTCCTGATGGCTATGAAGTCACTGGTGATTTTGAATTTAACGGCGGGTTTGACGCTATGCGCCAACTGCTATCACATCCGCTGCGTCCTCAGGCCGTCTTTACCGGAAATGACGCTATGGCTGTTGGCGTTTACCAGGCGTTATATCAGGCAGAGTTACAGGTTCCGCAGGATATCGCGGTGATTGGCTATGACGATATCGAACTGGCAAGCTTTATGACGCCACCATTAACCACTATCCACCAACCGAAAGATGAACTGGGGGAGCTGGCGATTGATGTACTCATCCATCGGATAACCCAGCCGACCCTTCAGCAACAACGATTACAACTTACTCCGATTCTGATGGAACGCGGTTCGGCTTAGCTGGTGAAAAGAAAAACCACCCTGGCGCCCAATACGCAAACCGCCTCTCCCCGCGCGTTGGCCGATTCATTAATGCAGCTGGCACGACAGGTTTCCCGACTGGAAAGCGGGCAGTGAgctttcctcggtaccaaattccagaaaagaggcctcccgaaaggggggccttttttcgttttggtcctacttgtgcctgttctatttccgaaccgaccgcttgtatgaatccatcaaaattcgttttctctatgttggattccttgttgctcatattgtgatgataatttctacaaatatagtcattggtaactatctatgaaactgtttgatacttttatagttgattaaacttgttcatggcatttgccttaatatcatccgctatgtcaatgtagggtttcatagctttgtagtcgctgtgtcccgtccatttcatgaccacctgtgccgggattccgagagccagcgcattgcagatgaatgtccttcttcctgcatgggtactgagcaaagcgtatttgggtgtgacttcatcaatacgttcatttcccttgtagtaggtttcccgtacaggctcgttgatttctgccagttcgcccagctctttcaggtaatcgttcatcttctggttgctgatgacgggcagagccatgtaattctcgaaatggatgtccttgtatttgtccagtatggctttgctgtatttgttcagttcaatcgtcaggctgtcggcagtcttgactgtggttatttcgatgtggtcggacttcacatcgcttcttttcagattgcgaacatccgaataccgcaaactcgtaaagcagcagaacaggaaaacatcacgcacacgttccaggtattgcttatccttgggtatctggtagtctttcagcttgttcagttcatcccaagtcaggaagattacttttttcgaggtggttttcagtttcggtttgaacgtatcgtatgcaatgttctgatgatgtcctttcttgaagctccagcgcaggaaccatttgaggaatcccatttgcttgccgatggtgctgtttctcatatccttggtgtcacgcaggaagttgacgtattcgttcaatccaaactcgttgaaatagttgaacgttgcatcctccttgaactctttgaggtggttcctcactgctgcaaatttttcataggtggatgccgtccagttattctggttaccgcactcttttacaaactcatcgaacacctcccaaaagctgacaggggcttcttccggctgttcttcgctggtgtctttcattctcatgttgaaagcttccttcaactgttgggtcgttggcatgacctcctgcacctcaaattccttgaaaatattctggatttcggcatagtatttcagcaagtccgtattgatttcggctgcactttgctttagcttgttggtacatccgctctttacccgctgcttatctgcatcccatttggctacgtcaatccggtagcccgttgtaaactcgatgcgttggctggcaaagatgacacgcatacggatgggtacgttctctacgattggcacaccgttctttttccggctctccaatgcaaaaatgatgttgcgcttgatattcataattgggtgcgtttgaaattctacacccaaatatacacccaattattgagatagcaaaagacatttagaaacatttacttttactctatattgtaatttacacttgattatcagtcgtttgcagtcttatgatattctgtgaaagtataagttcgagagcctgtctctccgcaaaaaacgctgaaaatcagcagattgcaaaacaaacaccctgttttacacccaagaatgtaaagtcggctgtttttgttttatttaagataatacaaccactacataataaaagagtagcgatattaaaagaatccgatgagaaaagactaatatttatctatccattcagtttgatttttcaggactttacatcgtcctgaaagtatttgttggtaccggtaccgaggacgcgtaaacatttacagttgcatgtggcctattgtttttagccgttaaatattttataactattaaatagcgatacaaattgttcgaaactaatattgtttatatcatatattctcgcatgttttaaagctttattaaattgattttttgtaaacagtttttcgtactctttgttaacccatttcattacaaaagtttcatatttttttctctctttaaatgccatttttgctggctttctttttaatacaattaatgtgctatccactttaggttttggatggaaataatacctaggaatttttgctaatatagaaatatctacctctgccattaacagcaatgctagtgatctgtttgtatctaataacattttagcaaaaccatattccactattaaataacttattgtggctgaactttcaaaaacaatttttcgaattatatttgtgcttatgttgtaaggtatgctgccaaatattttatatggattgtggctaggaaatgtaaatttcagtatatcatcatttactatttgatagttaggataatttaagagcttattacgagttacctcacataatttagaatcaatttctatcgccgttacaaaattacatctctttaccaatccagcagtaaaatgacctttccctgcacctatttcaaagatgttatctttttcatctaaacttatgcaattcattattttttctatgtgatattttgaagtaataaaattttgactatcttttatatttactttgttcattataacctctccttaatttattgcatctcttttcgaatatttatgttttttgagaaaagaacgtactcatggttcatcccgatatgcgtatcggtctgtatatcagcaactttctatgtgtttcaactacaatagtcatctattctcatctttctgagtccaccccctgcaaagcccctctttacgacataaaaattcggtcggaaaaggtatgcaaaagatgtttctctctttaagagaaactcttcgggatgcaaaaatatgaaaataactccaattcaccaaattatatagcgacttttttacaaaatgctaaaatttgttgatttccgtcaagcaattgttgagcaaaaatgtcttttacgataaaatgatacctcaatatcaactgtttagcaaaacgatatttctcttaaagagagaaacacctttttgttcaccaatccccgacttttaatcccgcggccatgattgaaaaaggaagagtatgagtattcaacatttccgtgtcgcccttattcccttttttgcggcattttgccttcctgtttttgctcacccagaaacgctggtgaaagtaaaagatgctgaagatcagttgggtgcacgagtgggttacatcgaactggatctcaacagcggtaagatccttgagagttttcgccccgaagaacgttttccaatgatgagcacttttaaagttctgctatgtggcgcggtattatcccgtattgacgccgggcaagagcaactcggtcgccgcatacactattctcagaatgacttggttgagtactcaccagtcacagaaaagcatcttacggatggcatgacagtaagagaattatgcagtgctgccataaccatgagtgataacactgcggccaacttacttctgacaacgatcggaggaccgaaggagctaaccgcttttttgcacaacatgggggatcatgtaactcgccttgatcgttgggaaccggagctgaatgaagccataccaaacgacgagcgtgacaccacgatgcctgtagcaatggcaacaacgttgcgcaaactattaactggcgaactacttactctagcttcccggcaacaattaatagactggatggaggcggataaagttgcaggaccacttctgcgctcggcccttccggctggctggtttattgctgataaatctggagccggtgagcgtgggtctcgcggtatcattgcagcactggggccagatggtaagccctcccgtatcgtagttatctacacgacggggagtcaggcaactatggatgaacgaaatagacagatcgctgagataggtgcctcactgattaagcattggtaactgtcagaccaagtttactcataacgcgtcaattcgagggggatcaattccgtgataggtgggctgcccttcctggttggcttggtttcatcagccatccgcttgccctcatctgttacgccggcggtagccggccagcctcgcagagcaggattcccgttgagcaccgccaggtgcgaataagggacagtgaagaaggaacacccgctcgcgggtgggcctacttcacctatcctgcccggctgacgccgttggatacaccaaggaaagtctacacgaaccctttggcaaaatcctgtatatcgtgcgaaaaaggatggatataccgaaaaaatcgctataatgaccccgaagcagggttatgcagcggaaaacggaattgatccggccacgatgcgtccggcgtagaggatctgaagatcagcagttcaacctgttgatagtacgtactaagctctcatgtttcacgtactaagctctcatgtttaacgtactaagctctcatgtttaacgaactaaaccctcatggctaacgtactaagctctcatggctaacgtactaagctctcatgtttcacgtactaagctctcatgtttgaacaataaaattaatataaatcagcaacttaaatagcctctaaggttttaagttttataagaaaaaaaagaatatataaggcttttaaagcttttaag

>pBH110

gtttaacggttgtggacaacaagccagggatgtaacgcactgagaagcccttagagcctctcaaagcaattttgagtgacacaggaacacttaacggctgacatgggaattcccctccaccgcggtggtacaaagaaaattcgacaaactgttatttttctatctatttatttgAATTgacAGCGCTgtcAATTtacctttgtcggcAATTgacAGCGCTgtcAATTaaataaagatattctcgtcaaacaaatataaataatataaacatggtttttactctggaagattttgttggcgattggcgtcagaccgcgggttataatttggatcaagtcctggaacagggtggcgtaagctctctgttccagaacctgggtgtgagcgtgacgccgattcagcgcatcgttctgtccggcgagaacggtctgaaaattgatattcatgtgatcatcccgtacgaaggcctgagcggtgaccaaatgggtcaaatcgagaaaatctttaaagtcgtctacccagttgacgatcaccacttcaaggttatcttgcattacggtacgctggtgattgatggtgtgaccccgaatatgattgactatttcggccgtccgtatgaaggcattgccgtttttgacggtaaaaagatcaccgtcaccggtaccctgtggaatggcaataagattattgacgagcgtctgattaacccggacggcagcctgctgttccgcgtgaccatcaacggtgtcacgggttggcgtctgtgcgagcgcatcctggcataatgaactgcacttgctttgataattaatgataaacaatctaaaagcactctaatcgttatcggagtgcttttagattactaatcaaattgcttctactaattgcctatcttccagtgatggaacagcatttgtgcattggctgcaacaatcagccttgatctggaagaagcaatgaaagctgctgttaagtctccgaatcaggtattgttcctgacaggtgtattcccatccggtaaacgcggatactttgcagttgatctgactcaggaataaattataaattaaggtaagaagattgtaggataagctaatgaaatagaaaaaggatgccgtcacacaacttgtcggcattcttttttgttttattagttgaaaatatagtgaaaaagttgcctaaatatgtatgttaacaaattatttgtcgtaactttgcactccaaatctgtttttaacatatggcactagtgAAACCAGTAACGTTATACGATGTCGCAGAGTATGCCGGTGTCTCTggaaagACCGTTTCCcgcGTGGTGAACCAGGCCAGCCACGTTTCTGCGAAAACGCGGGAAAAAGTGGAAGCGGCGATGGCGGAGCTCAATTACATTCCCAACCGCGTGGCACAACAACTGGCGGGCAAAGCGTCGCATACCATTGGCATGTTGATCACTGCCAGTACCAATCCTTTCTATTCAGAACTGGTGCGTGGCGTTGAACGCAGCTGCTTCGAACGCGGTTATAGTCTCGTCCTTTGCAATACCGAAGGCGATGAACAGCGGATGAATCGCAATCTGGAAACGCTGATGCAAAAACGCGTTGATGGCTTGCTGTTACTGTGCACCGAAACGCATCAACCTTCGCGTGAAATCATGCAACGTTATCCGACAGTGCCTACTGTGATGATGGACTGGGCTCCGTTCGATGGCGACAGCGATCTTATTCAGGATAACTCGTTGCTGGGCGGAGACTTAGCAACGCAATATCTGATCGATAAAGGTCATACCCGTATCGCCTGTATTACCGGCCCGCTGGATAAAACTCCGGCGCGCCTGCGGTTGGAAGGTTATCGGGCGGCGATGAAACGTGCGGGTCTCAACATTCCTGATGGCTATGAAGTCACTGGTGATTTTGAATTTAACGGCGGGTTTGACGCTATGCGCCAACTGCTATCACATCCGCTGCGTCCTCAGGCCGTCTTTACCGGAAATGACGCTATGGCTGTTGGCGTTTACCAGGCGTTATATCAGGCAGAGTTACAGGTTCCGCAGGATATCGCGGTGATTGGCTATGACGATATCGAACTGGCAAGCTTTATGACGCCACCATTAACCACTATCCACCAACCGAAAGATGAACTGGGGGAGCTGGCGATTGATGTACTCATCCATCGGATAACCCAGCCGACCCTTCAGCAACAACGATTACAACTTACTCCGATTCTGATGGAACGCGGTTCGGCTTAGCTGGTGAAAAGAAAAACCACCCTGGCGCCCAATACGCAAACCGCCTCTCCCCGCGCGTTGGCCGATTCATTAATGCAGCTGGCACGACAGGTTTCCCGACTGGAAAGCGGGCAGTGAgctttcctcggtaccaaattccagaaaagaggcctcccgaaaggggggccttttttcgttttggtcctacttgtgcctgttctatttccgaaccgaccgcttgtatgaatccatcaaaattcgttttctctatgttggattccttgttgctcatattgtgatgataatttctacaaatatagtcattggtaactatctatgaaactgtttgatacttttatagttgattaaacttgttcatggcatttgccttaatatcatccgctatgtcaatgtagggtttcatagctttgtagtcgctgtgtcccgtccatttcatgaccacctgtgccgggattccgagagccagcgcattgcagatgaatgtccttcttcctgcatgggtactgagcaaagcgtatttgggtgtgacttcatcaatacgttcatttcccttgtagtaggtttcccgtacaggctcgttgatttctgccagttcgcccagctctttcaggtaatcgttcatcttctggttgctgatgacgggcagagccatgtaattctcgaaatggatgtccttgtatttgtccagtatggctttgctgtatttgttcagttcaatcgtcaggctgtcggcagtcttgactgtggttatttcgatgtggtcggacttcacatcgcttcttttcagattgcgaacatccgaataccgcaaactcgtaaagcagcagaacaggaaaacatcacgcacacgttccaggtattgcttatccttgggtatctggtagtctttcagcttgttcagttcatcccaagtcaggaagattacttttttcgaggtggttttcagtttcggtttgaacgtatcgtatgcaatgttctgatgatgtcctttcttgaagctccagcgcaggaaccatttgaggaatcccatttgcttgccgatggtgctgtttctcatatccttggtgtcacgcaggaagttgacgtattcgttcaatccaaactcgttgaaatagttgaacgttgcatcctccttgaactctttgaggtggttcctcactgctgcaaatttttcataggtggatgccgtccagttattctggttaccgcactcttttacaaactcatcgaacacctcccaaaagctgacaggggcttcttccggctgttcttcgctggtgtctttcattctcatgttgaaagcttccttcaactgttgggtcgttggcatgacctcctgcacctcaaattccttgaaaatattctggatttcggcatagtatttcagcaagtccgtattgatttcggctgcactttgctttagcttgttggtacatccgctctttacccgctgcttatctgcatcccatttggctacgtcaatccggtagcccgttgtaaactcgatgcgttggctggcaaagatgacacgcatacggatgggtacgttctctacgattggcacaccgttctttttccggctctccaatgcaaaaatgatgttgcgcttgatattcataattgggtgcgtttgaaattctacacccaaatatacacccaattattgagatagcaaaagacatttagaaacatttacttttactctatattgtaatttacacttgattatcagtcgtttgcagtcttatgatattctgtgaaagtataagttcgagagcctgtctctccgcaaaaaacgctgaaaatcagcagattgcaaaacaaacaccctgttttacacccaagaatgtaaagtcggctgtttttgttttatttaagataatacaaccactacataataaaagagtagcgatattaaaagaatccgatgagaaaagactaatatttatctatccattcagtttgatttttcaggactttacatcgtcctgaaagtatttgttggtaccggtaccgaggacgcgtaaacatttacagttgcatgtggcctattgtttttagccgttaaatattttataactattaaatagcgatacaaattgttcgaaactaatattgtttatatcatatattctcgcatgttttaaagctttattaaattgattttttgtaaacagtttttcgtactctttgttaacccatttcattacaaaagtttcatatttttttctctctttaaatgccatttttgctggctttctttttaatacaattaatgtgctatccactttaggttttggatggaaataatacctaggaatttttgctaatatagaaatatctacctctgccattaacagcaatgctagtgatctgtttgtatctaataacattttagcaaaaccatattccactattaaataacttattgtggctgaactttcaaaaacaatttttcgaattatatttgtgcttatgttgtaaggtatgctgccaaatattttatatggattgtggctaggaaatgtaaatttcagtatatcatcatttactatttgatagttaggataatttaagagcttattacgagttacctcacataatttagaatcaatttctatcgccgttacaaaattacatctctttaccaatccagcagtaaaatgacctttccctgcacctatttcaaagatgttatctttttcatctaaacttatgcaattcattattttttctatgtgatattttgaagtaataaaattttgactatcttttatatttactttgttcattataacctctccttaatttattgcatctcttttcgaatatttatgttttttgagaaaagaacgtactcatggttcatcccgatatgcgtatcggtctgtatatcagcaactttctatgtgtttcaactacaatagtcatctattctcatctttctgagtccaccccctgcaaagcccctctttacgacataaaaattcggtcggaaaaggtatgcaaaagatgtttctctctttaagagaaactcttcgggatgcaaaaatatgaaaataactccaattcaccaaattatatagcgacttttttacaaaatgctaaaatttgttgatttccgtcaagcaattgttgagcaaaaatgtcttttacgataaaatgatacctcaatatcaactgtttagcaaaacgatatttctcttaaagagagaaacacctttttgttcaccaatccccgacttttaatcccgcggccatgattgaaaaaggaagagtatgagtattcaacatttccgtgtcgcccttattcccttttttgcggcattttgccttcctgtttttgctcacccagaaacgctggtgaaagtaaaagatgctgaagatcagttgggtgcacgagtgggttacatcgaactggatctcaacagcggtaagatccttgagagttttcgccccgaagaacgttttccaatgatgagcacttttaaagttctgctatgtggcgcggtattatcccgtattgacgccgggcaagagcaactcggtcgccgcatacactattctcagaatgacttggttgagtactcaccagtcacagaaaagcatcttacggatggcatgacagtaagagaattatgcagtgctgccataaccatgagtgataacactgcggccaacttacttctgacaacgatcggaggaccgaaggagctaaccgcttttttgcacaacatgggggatcatgtaactcgccttgatcgttgggaaccggagctgaatgaagccataccaaacgacgagcgtgacaccacgatgcctgtagcaatggcaacaacgttgcgcaaactattaactggcgaactacttactctagcttcccggcaacaattaatagactggatggaggcggataaagttgcaggaccacttctgcgctcggcccttccggctggctggtttattgctgataaatctggagccggtgagcgtgggtctcgcggtatcattgcagcactggggccagatggtaagccctcccgtatcgtagttatctacacgacggggagtcaggcaactatggatgaacgaaatagacagatcgctgagataggtgcctcactgattaagcattggtaactgtcagaccaagtttactcataacgcgtcaattcgagggggatcaattccgtgataggtgggctgcccttcctggttggcttggtttcatcagccatccgcttgccctcatctgttacgccggcggtagccggccagcctcgcagagcaggattcccgttgagcaccgccaggtgcgaataagggacagtgaagaaggaacacccgctcgcgggtgggcctacttcacctatcctgcccggctgacgccgttggatacaccaaggaaagtctacacgaaccctttggcaaaatcctgtatatcgtgcgaaaaaggatggatataccgaaaaaatcgctataatgaccccgaagcagggttatgcagcggaaaacggaattgatccggccacgatgcgtccggcgtagaggatctgaagatcagcagttcaacctgttgatagtacgtactaagctctcatgtttcacgtactaagctctcatgtttaacgtactaagctctcatgtttaacgaactaaaccctcatggctaacgtactaagctctcatggctaacgtactaagctctcatgtttcacgtactaagctctcatgtttgaacaataaaattaatataaatcagcaacttaaatagcctctaaggttttaagttttataagaaaaaaaagaatatataaggcttttaaagcttttaag

>pBH111

gtttaacggttgtggacaacaagccagggatgtaacgcactgagaagcccttagagcctctcaaagcaattttgagtgacacaggaacacttaacggctgacatgggaattcccctccaccgcggtggtacaaagaaaattcgacaaactgttatttttctatctatttatttgAATTGTGAGCGGATAACAATTacctttgtcggcAATTGTGAGCGGATAACAATTaaataaagatattctcgtcaaacaaatataaataatataaacatggtttttactctggaagattttgttggcgattggcgtcagaccgcgggttataatttggatcaagtcctggaacagggtggcgtaagctctctgttccagaacctgggtgtgagcgtgacgccgattcagcgcatcgttctgtccggcgagaacggtctgaaaattgatattcatgtgatcatcccgtacgaaggcctgagcggtgaccaaatgggtcaaatcgagaaaatctttaaagtcgtctacccagttgacgatcaccacttcaaggttatcttgcattacggtacgctggtgattgatggtgtgaccccgaatatgattgactatttcggccgtccgtatgaaggcattgccgtttttgacggtaaaaagatcaccgtcaccggtaccctgtggaatggcaataagattattgacgagcgtctgattaacccggacggcagcctgctgttccgcgtgaccatcaacggtgtcacgggttggcgtctgtgcgagcgcatcctggcataatgaactgcacttgctttgataattaatgataaacaatctaaaagcactctaatcgttatcggagtgcttttagattactaatcaaattgcttctactaattgcctatcttccagtgatggaacagcatttgtgcattggctgcaacaatcagccttgatctggaagaagcaatgaaagctgctgttaagtctccgaatcaggtattgttcctgacaggtgtattcccatccggtaaacgcggatactttgcagttgatctgactcaggaataaattataaattaaggtaagaagattgtaggataagctaatgaaatagaaaaaggatgccgtcacacaacttgtcggcattcttttttgttttattagttgaaaatatagtgaaaaagttgcctaaatatgtatgttaacaaattatttgtcgtaactttgcactccaaatctgtttttaacatatggcactagtgAAACCAGTAACGTTATACGATGTCGCAGAGTATGCCGGTGTCTCTTATCAGACCGTTTCCCGCGTGGTGAACCAGGCCAGCCACGTTTCTGCGAAAACGCGGGAAAAAGTGGAAGCGGCGATGGCGGAGCTGAATTACATTCCCAACCGCGTGGCACAACAACTGGCGGGCAAACAGTCGTTGCTGATTGGCGTTGCCACCTCCAGTCTGGCCCTGCACGCGCTGTCGCAAATTGTCGCGGCGATTAAATCTCGCGCCTATCAACTGGGTGCCAGCGTGTTCGTGTCGATGGTAGAACGAAGCGGCATCGAAGCCTGTAAAACGGCGGTGCACAATCTTCTCGCGCAACGCGTCAGTGGGCTGATCATTAACTATCCGCTGGATAACCAGGATGCCATTGCTGTGGAAGCTGCCTGCACTAATGTTCCGGCGTTATTTCTTGATGTCTCTGACCAGACACCCATCAACAGTATTATTTTCTCCCATGAAGACGGTACGCGACTGGGCGTGGAGCATCTGGTCGCATTGGGTCACCAGCAAATCGCGCTGTTAGCGGGCCCATTAAGTTCTGTCTCGGCGCGTCTGCGTCTGGCTGGCTGGCATAAATATCTCACTCGCAATCAAATTCAGCCGATAGCGGAACGGGAAGGCGACTGGAGTGCCATGTCCGGTTTTCAACAAACCATGCAAATGCTGAATGAGGGCATCGTTCCCACTGCGATGCTGGTTGCCAACGATCAGATGGCGCTGGGCGCAATGCGCGCCATTACCGAGACCGGGCTGCGCGTTGGTGCGGATATCTCGGTAGTGGGATACGACGATACCGAAGACAGCTCATGTTATATCCCGCCGTTAACCACCATCAAACAGGATTTTCGCCTGCTGGGGCAAACCAGCGTGGACCGCTTGCTGCAACTCTCTCAGGGCCAGGCGGTGAAGGGCAATCAGCTGTTGCCCGTCTCACTGGTGAAAAGAAAAACCACCCTGGCGCCCAATACGCAAACCGCCTCTCCCCGCGCGTTGGCCGATTCATTAATGCAGCTGGCACGACAGGTTTCCCGACTGGAAAGCGGGCAGtgagctttcctcggtaccaaattccagaaaagaggcctcccgaaaggggggccttttttcgttttggtcctacttgtgcctgttctatttccgaaccgaccgcttgtatgaatccatcaaaattcgttttctctatgttggattccttgttgctcatattgtgatgataatttctacaaatatagtcattggtaactatctatgaaactgtttgatacttttatagttgattaaacttgttcatggcatttgccttaatatcatccgctatgtcaatgtagggtttcatagctttgtagtcgctgtgtcccgtccatttcatgaccacctgtgccgggattccgagagccagcgcattgcagatgaatgtccttcttcctgcatgggtactgagcaaagcgtatttgggtgtgacttcatcaatacgttcatttcccttgtagtaggtttcccgtacaggctcgttgatttctgccagttcgcccagctctttcaggtaatcgttcatcttctggttgctgatgacgggcagagccatgtaattctcgaaatggatgtccttgtatttgtccagtatggctttgctgtatttgttcagttcaatcgtcaggctgtcggcagtcttgactgtggttatttcgatgtggtcggacttcacatcgcttcttttcagattgcgaacatccgaataccgcaaactcgtaaagcagcagaacaggaaaacatcacgcacacgttccaggtattgcttatccttgggtatctggtagtctttcagcttgttcagttcatcccaagtcaggaagattacttttttcgaggtggttttcagtttcggtttgaacgtatcgtatgcaatgttctgatgatgtcctttcttgaagctccagcgcaggaaccatttgaggaatcccatttgcttgccgatggtgctgtttctcatatccttggtgtcacgcaggaagttgacgtattcgttcaatccaaactcgttgaaatagttgaacgttgcatcctccttgaactctttgaggtggttcctcactgctgcaaatttttcataggtggatgccgtccagttattctggttaccgcactcttttacaaactcatcgaacacctcccaaaagctgacaggggcttcttccggctgttcttcgctggtgtctttcattctcatgttgaaagcttccttcaactgttgggtcgttggcatgacctcctgcacctcaaattccttgaaaatattctggatttcggcatagtatttcagcaagtccgtattgatttcggctgcactttgctttagcttgttggtacatccgctctttacccgctgcttatctgcatcccatttggctacgtcaatccggtagcccgttgtaaactcgatgcgttggctggcaaagatgacacgcatacggatgggtacgttctctacgattggcacaccgttctttttccggctctccaatgcaaaaatgatgttgcgcttgatattcataattgggtgcgtttgaaattctacacccaaatatacacccaattattgagatagcaaaagacatttagaaacatttacttttactctatattgtaatttacacttgattatcagtcgtttgcagtcttatgatattctgtgaaagtataagttcgagagcctgtctctccgcaaaaaacgctgaaaatcagcagattgcaaaacaaacaccctgttttacacccaagaatgtaaagtcggctgtttttgttttatttaagataatacaaccactacataataaaagagtagcgatattaaaagaatccgatgagaaaagactaatatttatctatccattcagtttgatttttcaggactttacatcgtcctgaaagtatttgttggtaccggtaccgaggacgcgtaaacatttacagttgcatgtggcctattgtttttagccgttaaatattttataactattaaatagcgatacaaattgttcgaaactaatattgtttatatcatatattctcgcatgttttaaagctttattaaattgattttttgtaaacagtttttcgtactctttgttaacccatttcattacaaaagtttcatatttttttctctctttaaatgccatttttgctggctttctttttaatacaattaatgtgctatccactttaggttttggatggaaataatacctaggaatttttgctaatatagaaatatctacctctgccattaacagcaatgctagtgatctgtttgtatctaataacattttagcaaaaccatattccactattaaataacttattgtggctgaactttcaaaaacaatttttcgaattatatttgtgcttatgttgtaaggtatgctgccaaatattttatatggattgtggctaggaaatgtaaatttcagtatatcatcatttactatttgatagttaggataatttaagagcttattacgagttacctcacataatttagaatcaatttctatcgccgttacaaaattacatctctttaccaatccagcagtaaaatgacctttccctgcacctatttcaaagatgttatctttttcatctaaacttatgcaattcattattttttctatgtgatattttgaagtaataaaattttgactatcttttatatttactttgttcattataacctctccttaatttattgcatctcttttcgaatatttatgttttttgagaaaagaacgtactcatggttcatcccgatatgcgtatcggtctgtatatcagcaactttctatgtgtttcaactacaatagtcatctattctcatctttctgagtccaccccctgcaaagcccctctttacgacataaaaattcggtcggaaaaggtatgcaaaagatgtttctctctttaagagaaactcttcgggatgcaaaaatatgaaaataactccaattcaccaaattatatagcgacttttttacaaaatgctaaaatttgttgatttccgtcaagcaattgttgagcaaaaatgtcttttacgataaaatgatacctcaatatcaactgtttagcaaaacgatatttctcttaaagagagaaacacctttttgttcaccaatccccgacttttaatcccgcggccatgattgaaaaaggaagagtatgagtattcaacatttccgtgtcgcccttattcccttttttgcggcattttgccttcctgtttttgctcacccagaaacgctggtgaaagtaaaagatgctgaagatcagttgggtgcacgagtgggttacatcgaactggatctcaacagcggtaagatccttgagagttttcgccccgaagaacgttttccaatgatgagcacttttaaagttctgctatgtggcgcggtattatcccgtattgacgccgggcaagagcaactcggtcgccgcatacactattctcagaatgacttggttgagtactcaccagtcacagaaaagcatcttacggatggcatgacagtaagagaattatgcagtgctgccataaccatgagtgataacactgcggccaacttacttctgacaacgatcggaggaccgaaggagctaaccgcttttttgcacaacatgggggatcatgtaactcgccttgatcgttgggaaccggagctgaatgaagccataccaaacgacgagcgtgacaccacgatgcctgtagcaatggcaacaacgttgcgcaaactattaactggcgaactacttactctagcttcccggcaacaattaatagactggatggaggcggataaagttgcaggaccacttctgcgctcggcccttccggctggctggtttattgctgataaatctggagccggtgagcgtgggtctcgcggtatcattgcagcactggggccagatggtaagccctcccgtatcgtagttatctacacgacggggagtcaggcaactatggatgaacgaaatagacagatcgctgagataggtgcctcactgattaagcattggtaactgtcagaccaagtttactcataacgcgtcaattcgagggggatcaattccgtgataggtgggctgcccttcctggttggcttggtttcatcagccatccgcttgccctcatctgttacgccggcggtagccggccagcctcgcagagcaggattcccgttgagcaccgccaggtgcgaataagggacagtgaagaaggaacacccgctcgcgggtgggcctacttcacctatcctgcccggctgacgccgttggatacaccaaggaaagtctacacgaaccctttggcaaaatcctgtatatcgtgcgaaaaaggatggatataccgaaaaaatcgctataatgaccccgaagcagggttatgcagcggaaaacggaattgatccggccacgatgcgtccggcgtagaggatctgaagatcagcagttcaacctgttgatagtacgtactaagctctcatgtttcacgtactaagctctcatgtttaacgtactaagctctcatgtttaacgaactaaaccctcatggctaacgtactaagctctcatggctaacgtactaagctctcatgtttcacgtactaagctctcatgtttgaacaataaaattaatataaatcagcaacttaaatagcctctaaggttttaagttttataagaaaaaaaagaatatataaggcttttaaagcttttaag

>pBH112

gtttaacggttgtggacaacaagccagggatgtaacgcactgagaagcccttagagcctctcaaagcaattttgagtgacacaggaacacttaacggctgacatgggaattcccctccaccgcggtggtacaaagaaaattcgacaaactgttatttttctatctatttatttgaattTTAAGCGCTTAAaatttacctttgtcggcaattTTAAGCGCTTAAaattaaataaagatattctcgtcaaacaaatataaataatataaacatggtttttactctggaagattttgttggcgattggcgtcagaccgcgggttataatttggatcaagtcctggaacagggtggcgtaagctctctgttccagaacctgggtgtgagcgtgacgccgattcagcgcatcgttctgtccggcgagaacggtctgaaaattgatattcatgtgatcatcccgtacgaaggcctgagcggtgaccaaatgggtcaaatcgagaaaatctttaaagtcgtctacccagttgacgatcaccacttcaaggttatcttgcattacggtacgctggtgattgatggtgtgaccccgaatatgattgactatttcggccgtccgtatgaaggcattgccgtttttgacggtaaaaagatcaccgtcaccggtaccctgtggaatggcaataagattattgacgagcgtctgattaacccggacggcagcctgctgttccgcgtgaccatcaacggtgtcacgggttggcgtctgtgcgagcgcatcctggcataatgaactgcacttgctttgataattaatgataaacaatctaaaagcactctaatcgttatcggagtgcttttagattactaatcaaattgcttctactaattgcctatcttccagtgatggaacagcatttgtgcattggctgcaacaatcagccttgatctggaagaagcaatgaaagctgctgttaagtctccgaatcaggtattgttcctgacaggtgtattcccatccggtaaacgcggatactttgcagttgatctgactcaggaataaattataaattaaggtaagaagattgtaggataagctaatgaaatagaaaaaggatgccgtcacacaacttgtcggcattcttttttgttttattagttgaaaatatagtgaaaaagttgcctaaatatgtatgttaacaaattatttgtcgtaactttgcactccaaatctgtttttaacatatggcactagtgAAACCAGTAACGTTATACGATGTCGCAGAGTATGCCGGTGTCTCTACCGCGACCGTTTCCAACGTGGTGAACCAGGCCAGCCACGTTTCTGCGAAAACGCGGGAAAAAGTGGAAGCGGCGATGGCGGAGCTGAATTACATTCCCAACCGCGTGGCACAACAACTGGCGGGCAAACAGTCGTTGCTGATTGGCGTTGCCACCTCCAGTCTGGCCCTGCACGCGCTGTCGCAAATTGTCGCGGCGATTAAATCTCGCGCCTATCAACTGGGTGCCAGCGTGTTCGTGTCGATGGTAGAACGAAGCGGCATCGAAGCCTGTAAAACGGCGGTGCACAATCTTCTCGCGCAACGCGTCAGTGGGCTGATCATTAACTATCCGCTGGATAACCAGGATGCCATTGCTGTGGAAGCTGCCTGCACTAATGTTCCGGCGTTATTTCTTGATGTCTCTGACCAGACACCCATCAACAGTATTATTTTCTCCCATGAAGACGGTACGCGACTGGGCGTGGAGCATCTGGTCGCATTGGGTCACCAGCAAATCGCGCTGTTAGCGGGCCCATTAAGTTCTGTCTCGGCGCGTCTGCGTCTGGCTGGCTGGCATAAATATCTCACTCGCAATCAAATTCAGCCGATAGCGGAACGGGAAGGCGACTGGAGTGCCATGTCCGGTTTTCAACAAACCATGCAAATGCTGAATGAGGGCATCGTTCCCACTGCGATGCTGGTTGCCAACGATCAGATGGCGCTGGGCGCAATGCGCGCCATTACCGAGACCGGGCTGCGCGTTGGTGCGGATATCTCGGTAGTGGGATACGACGATACCGAAGACAGCTCATGTTATATCCCGCCGTTAACCACCATCAAACAGGATTTTCGCCTGCTGGGGCAAACCAGCGTGGACCGCTTGCTGCAACTCTCTCAGGGCCAGGCGGTGAAGGGCAATCAGCTGTTGCCCGTCTCACTGGTGAAAAGAAAAACCACCCTGGCGCCCAATACGCAAACCGCCTCTCCCCGCGCGTTGGCCGATTCATTAATGCAGCTGGCACGACAGGTTTCCCGACTGGAAAGCGGGCAGtgagctttcctcggtaccaaattccagaaaagaggcctcccgaaaggggggccttttttcgttttggtcctacttgtgcctgttctatttccgaaccgaccgcttgtatgaatccatcaaaattcgttttctctatgttggattccttgttgctcatattgtgatgataatttctacaaatatagtcattggtaactatctatgaaactgtttgatacttttatagttgattaaacttgttcatggcatttgccttaatatcatccgctatgtcaatgtagggtttcatagctttgtagtcgctgtgtcccgtccatttcatgaccacctgtgccgggattccgagagccagcgcattgcagatgaatgtccttcttcctgcatgggtactgagcaaagcgtatttgggtgtgacttcatcaatacgttcatttcccttgtagtaggtttcccgtacaggctcgttgatttctgccagttcgcccagctctttcaggtaatcgttcatcttctggttgctgatgacgggcagagccatgtaattctcgaaatggatgtccttgtatttgtccagtatggctttgctgtatttgttcagttcaatcgtcaggctgtcggcagtcttgactgtggttatttcgatgtggtcggacttcacatcgcttcttttcagattgcgaacatccgaataccgcaaactcgtaaagcagcagaacaggaaaacatcacgcacacgttccaggtattgcttatccttgggtatctggtagtctttcagcttgttcagttcatcccaagtcaggaagattacttttttcgaggtggttttcagtttcggtttgaacgtatcgtatgcaatgttctgatgatgtcctttcttgaagctccagcgcaggaaccatttgaggaatcccatttgcttgccgatggtgctgtttctcatatccttggtgtcacgcaggaagttgacgtattcgttcaatccaaactcgttgaaatagttgaacgttgcatcctccttgaactctttgaggtggttcctcactgctgcaaatttttcataggtggatgccgtccagttattctggttaccgcactcttttacaaactcatcgaacacctcccaaaagctgacaggggcttcttccggctgttcttcgctggtgtctttcattctcatgttgaaagcttccttcaactgttgggtcgttggcatgacctcctgcacctcaaattccttgaaaatattctggatttcggcatagtatttcagcaagtccgtattgatttcggctgcactttgctttagcttgttggtacatccgctctttacccgctgcttatctgcatcccatttggctacgtcaatccggtagcccgttgtaaactcgatgcgttggctggcaaagatgacacgcatacggatgggtacgttctctacgattggcacaccgttctttttccggctctccaatgcaaaaatgatgttgcgcttgatattcataattgggtgcgtttgaaattctacacccaaatatacacccaattattgagatagcaaaagacatttagaaacatttacttttactctatattgtaatttacacttgattatcagtcgtttgcagtcttatgatattctgtgaaagtataagttcgagagcctgtctctccgcaaaaaacgctgaaaatcagcagattgcaaaacaaacaccctgttttacacccaagaatgtaaagtcggctgtttttgttttatttaagataatacaaccactacataataaaagagtagcgatattaaaagaatccgatgagaaaagactaatatttatctatccattcagtttgatttttcaggactttacatcgtcctgaaagtatttgttggtaccggtaccgaggacgcgtaaacatttacagttgcatgtggcctattgtttttagccgttaaatattttataactattaaatagcgatacaaattgttcgaaactaatattgtttatatcatatattctcgcatgttttaaagctttattaaattgattttttgtaaacagtttttcgtactctttgttaacccatttcattacaaaagtttcatatttttttctctctttaaatgccatttttgctggctttctttttaatacaattaatgtgctatccactttaggttttggatggaaataatacctaggaatttttgctaatatagaaatatctacctctgccattaacagcaatgctagtgatctgtttgtatctaataacattttagcaaaaccatattccactattaaataacttattgtggctgaactttcaaaaacaatttttcgaattatatttgtgcttatgttgtaaggtatgctgccaaatattttatatggattgtggctaggaaatgtaaatttcagtatatcatcatttactatttgatagttaggataatttaagagcttattacgagttacctcacataatttagaatcaatttctatcgccgttacaaaattacatctctttaccaatccagcagtaaaatgacctttccctgcacctatttcaaagatgttatctttttcatctaaacttatgcaattcattattttttctatgtgatattttgaagtaataaaattttgactatcttttatatttactttgttcattataacctctccttaatttattgcatctcttttcgaatatttatgttttttgagaaaagaacgtactcatggttcatcccgatatgcgtatcggtctgtatatcagcaactttctatgtgtttcaactacaatagtcatctattctcatctttctgagtccaccccctgcaaagcccctctttacgacataaaaattcggtcggaaaaggtatgcaaaagatgtttctctctttaagagaaactcttcgggatgcaaaaatatgaaaataactccaattcaccaaattatatagcgacttttttacaaaatgctaaaatttgttgatttccgtcaagcaattgttgagcaaaaatgtcttttacgataaaatgatacctcaatatcaactgtttagcaaaacgatatttctcttaaagagagaaacacctttttgttcaccaatccccgacttttaatcccgcggccatgattgaaaaaggaagagtatgagtattcaacatttccgtgtcgcccttattcccttttttgcggcattttgccttcctgtttttgctcacccagaaacgctggtgaaagtaaaagatgctgaagatcagttgggtgcacgagtgggttacatcgaactggatctcaacagcggtaagatccttgagagttttcgccccgaagaacgttttccaatgatgagcacttttaaagttctgctatgtggcgcggtattatcccgtattgacgccgggcaagagcaactcggtcgccgcatacactattctcagaatgacttggttgagtactcaccagtcacagaaaagcatcttacggatggcatgacagtaagagaattatgcagtgctgccataaccatgagtgataacactgcggccaacttacttctgacaacgatcggaggaccgaaggagctaaccgcttttttgcacaacatgggggatcatgtaactcgccttgatcgttgggaaccggagctgaatgaagccataccaaacgacgagcgtgacaccacgatgcctgtagcaatggcaacaacgttgcgcaaactattaactggcgaactacttactctagcttcccggcaacaattaatagactggatggaggcggataaagttgcaggaccacttctgcgctcggcccttccggctggctggtttattgctgataaatctggagccggtgagcgtgggtctcgcggtatcattgcagcactggggccagatggtaagccctcccgtatcgtagttatctacacgacggggagtcaggcaactatggatgaacgaaatagacagatcgctgagataggtgcctcactgattaagcattggtaactgtcagaccaagtttactcataacgcgtcaattcgagggggatcaattccgtgataggtgggctgcccttcctggttggcttggtttcatcagccatccgcttgccctcatctgttacgccggcggtagccggccagcctcgcagagcaggattcccgttgagcaccgccaggtgcgaataagggacagtgaagaaggaacacccgctcgcgggtgggcctacttcacctatcctgcccggctgacgccgttggatacaccaaggaaagtctacacgaaccctttggcaaaatcctgtatatcgtgcgaaaaaggatggatataccgaaaaaatcgctataatgaccccgaagcagggttatgcagcggaaaacggaattgatccggccacgatgcgtccggcgtagaggatctgaagatcagcagttcaacctgttgatagtacgtactaagctctcatgtttcacgtactaagctctcatgtttaacgtactaagctctcatgtttaacgaactaaaccctcatggctaacgtactaagctctcatggctaacgtactaagctctcatgtttcacgtactaagctctcatgtttgaacaataaaattaatataaatcagcaacttaaatagcctctaaggttttaagttttataagaaaaaaaagaatatataaggcttttaaagcttttaag

>pBH113

gtttaacggttgtggacaacaagccagggatgtaacgcactgagaagcccttagagcctctcaaagcaattttgagtgacacaggaacacttaacggctgacatgggaattcccctccaccgcggtggtacaaagaaaattcgacaaactgttatttttctatctatttatttgAATTaggAGCGCTcctAATTtacctttgtcggcAATTaggAGCGCTcctAATTaaataaagatattctcgtcaaacaaatataaataatataaacatggtttttactctggaagattttgttggcgattggcgtcagaccgcgggttataatttggatcaagtcctggaacagggtggcgtaagctctctgttccagaacctgggtgtgagcgtgacgccgattcagcgcatcgttctgtccggcgagaacggtctgaaaattgatattcatgtgatcatcccgtacgaaggcctgagcggtgaccaaatgggtcaaatcgagaaaatctttaaagtcgtctacccagttgacgatcaccacttcaaggttatcttgcattacggtacgctggtgattgatggtgtgaccccgaatatgattgactatttcggccgtccgtatgaaggcattgccgtttttgacggtaaaaagatcaccgtcaccggtaccctgtggaatggcaataagattattgacgagcgtctgattaacccggacggcagcctgctgttccgcgtgaccatcaacggtgtcacgggttggcgtctgtgcgagcgcatcctggcataatgaactgcacttgctttgataattaatgataaacaatctaaaagcactctaatcgttatcggagtgcttttagattactaatcaaattgcttctactaattgcctatcttccagtgatggaacagcatttgtgcattggctgcaacaatcagccttgatctggaagaagcaatgaaagctgctgttaagtctccgaatcaggtattgttcctgacaggtgtattcccatccggtaaacgcggatactttgcagttgatctgactcaggaataaattataaattaaggtaagaagattgtaggataagctaatgaaatagaaaaaggatgccgtcacacaacttgtcggcattcttttttgttttattagttgaaaatatagtgaaaaagttgcctaaatatgtatgttaacaaattatttgtcgtaactttgcactccaaatctgtttttaacatatggcactagtgAAACCAGTAACGTTATACGATGTCGCAGAGTATGCCGGTGTCTCTAAAAGCACCGTTTCCCTGGTGGTGAACCAGGCCAGCCACGTTTCTGCGAAAACGCGGGAAAAAGTGGAAGCGGCGATGGCGGAGCTGAATTACATTCCCAACCGCGTGGCACAACAACTGGCGGGCAAACAGTCGTTGCTGATTGGCGTTGCCACCTCCAGTCTGGCCCTGCACGCGCTGTCGCAAATTGTCGCGGCGATTAAATCTCGCGCCTATCAACTGGGTGCCAGCGTGTTCGTGTCGATGGTAGAACGAAGCGGCATCGAAGCCTGTAAAACGGCGGTGCACAATCTTCTCGCGCAACGCGTCAGTGGGCTGATCATTAACTATCCGCTGGATAACCAGGATGCCATTGCTGTGGAAGCTGCCTGCACTAATGTTCCGGCGTTATTTCTTGATGTCTCTGACCAGACACCCATCAACAGTATTATTTTCTCCCATGAAGACGGTACGCGACTGGGCGTGGAGCATCTGGTCGCATTGGGTCACCAGCAAATCGCGCTGTTAGCGGGCCCATTAAGTTCTGTCTCGGCGCGTCTGCGTCTGGCTGGCTGGCATAAATATCTCACTCGCAATCAAATTCAGCCGATAGCGGAACGGGAAGGCGACTGGAGTGCCATGTCCGGTTTTCAACAAACCATGCAAATGCTGAATGAGGGCATCGTTCCCACTGCGATGCTGGTTGCCAACGATCAGATGGCGCTGGGCGCAATGCGCGCCATTACCGAGACCGGGCTGCGCGTTGGTGCGGATATCTCGGTAGTGGGATACGACGATACCGAAGACAGCTCATGTTATATCCCGCCGTTAACCACCATCAAACAGGATTTTCGCCTGCTGGGGCAAACCAGCGTGGACCGCTTGCTGCAACTCTCTCAGGGCCAGGCGGTGAAGGGCAATCAGCTGTTGCCCGTCTCACTGGTGAAAAGAAAAACCACCCTGGCGCCCAATACGCAAACCGCCTCTCCCCGCGCGTTGGCCGATTCATTAATGCAGCTGGCACGACAGGTTTCCCGACTGGAAAGCGGGCAGtgagctttcctcggtaccaaattccagaaaagaggcctcccgaaaggggggccttttttcgttttggtcctacttgtgcctgttctatttccgaaccgaccgcttgtatgaatccatcaaaattcgttttctctatgttggattccttgttgctcatattgtgatgataatttctacaaatatagtcattggtaactatctatgaaactgtttgatacttttatagttgattaaacttgttcatggcatttgccttaatatcatccgctatgtcaatgtagggtttcatagctttgtagtcgctgtgtcccgtccatttcatgaccacctgtgccgggattccgagagccagcgcattgcagatgaatgtccttcttcctgcatgggtactgagcaaagcgtatttgggtgtgacttcatcaatacgttcatttcccttgtagtaggtttcccgtacaggctcgttgatttctgccagttcgcccagctctttcaggtaatcgttcatcttctggttgctgatgacgggcagagccatgtaattctcgaaatggatgtccttgtatttgtccagtatggctttgctgtatttgttcagttcaatcgtcaggctgtcggcagtcttgactgtggttatttcgatgtggtcggacttcacatcgcttcttttcagattgcgaacatccgaataccgcaaactcgtaaagcagcagaacaggaaaacatcacgcacacgttccaggtattgcttatccttgggtatctggtagtctttcagcttgttcagttcatcccaagtcaggaagattacttttttcgaggtggttttcagtttcggtttgaacgtatcgtatgcaatgttctgatgatgtcctttcttgaagctccagcgcaggaaccatttgaggaatcccatttgcttgccgatggtgctgtttctcatatccttggtgtcacgcaggaagttgacgtattcgttcaatccaaactcgttgaaatagttgaacgttgcatcctccttgaactctttgaggtggttcctcactgctgcaaatttttcataggtggatgccgtccagttattctggttaccgcactcttttacaaactcatcgaacacctcccaaaagctgacaggggcttcttccggctgttcttcgctggtgtctttcattctcatgttgaaagcttccttcaactgttgggtcgttggcatgacctcctgcacctcaaattccttgaaaatattctggatttcggcatagtatttcagcaagtccgtattgatttcggctgcactttgctttagcttgttggtacatccgctctttacccgctgcttatctgcatcccatttggctacgtcaatccggtagcccgttgtaaactcgatgcgttggctggcaaagatgacacgcatacggatgggtacgttctctacgattggcacaccgttctttttccggctctccaatgcaaaaatgatgttgcgcttgatattcataattgggtgcgtttgaaattctacacccaaatatacacccaattattgagatagcaaaagacatttagaaacatttacttttactctatattgtaatttacacttgattatcagtcgtttgcagtcttatgatattctgtgaaagtataagttcgagagcctgtctctccgcaaaaaacgctgaaaatcagcagattgcaaaacaaacaccctgttttacacccaagaatgtaaagtcggctgtttttgttttatttaagataatacaaccactacataataaaagagtagcgatattaaaagaatccgatgagaaaagactaatatttatctatccattcagtttgatttttcaggactttacatcgtcctgaaagtatttgttggtaccggtaccgaggacgcgtaaacatttacagttgcatgtggcctattgtttttagccgttaaatattttataactattaaatagcgatacaaattgttcgaaactaatattgtttatatcatatattctcgcatgttttaaagctttattaaattgattttttgtaaacagtttttcgtactctttgttaacccatttcattacaaaagtttcatatttttttctctctttaaatgccatttttgctggctttctttttaatacaattaatgtgctatccactttaggttttggatggaaataatacctaggaatttttgctaatatagaaatatctacctctgccattaacagcaatgctagtgatctgtttgtatctaataacattttagcaaaaccatattccactattaaataacttattgtggctgaactttcaaaaacaatttttcgaattatatttgtgcttatgttgtaaggtatgctgccaaatattttatatggattgtggctaggaaatgtaaatttcagtatatcatcatttactatttgatagttaggataatttaagagcttattacgagttacctcacataatttagaatcaatttctatcgccgttacaaaattacatctctttaccaatccagcagtaaaatgacctttccctgcacctatttcaaagatgttatctttttcatctaaacttatgcaattcattattttttctatgtgatattttgaagtaataaaattttgactatcttttatatttactttgttcattataacctctccttaatttattgcatctcttttcgaatatttatgttttttgagaaaagaacgtactcatggttcatcccgatatgcgtatcggtctgtatatcagcaactttctatgtgtttcaactacaatagtcatctattctcatctttctgagtccaccccctgcaaagcccctctttacgacataaaaattcggtcggaaaaggtatgcaaaagatgtttctctctttaagagaaactcttcgggatgcaaaaatatgaaaataactccaattcaccaaattatatagcgacttttttacaaaatgctaaaatttgttgatttccgtcaagcaattgttgagcaaaaatgtcttttacgataaaatgatacctcaatatcaactgtttagcaaaacgatatttctcttaaagagagaaacacctttttgttcaccaatccccgacttttaatcccgcggccatgattgaaaaaggaagagtatgagtattcaacatttccgtgtcgcccttattcccttttttgcggcattttgccttcctgtttttgctcacccagaaacgctggtgaaagtaaaagatgctgaagatcagttgggtgcacgagtgggttacatcgaactggatctcaacagcggtaagatccttgagagttttcgccccgaagaacgttttccaatgatgagcacttttaaagttctgctatgtggcgcggtattatcccgtattgacgccgggcaagagcaactcggtcgccgcatacactattctcagaatgacttggttgagtactcaccagtcacagaaaagcatcttacggatggcatgacagtaagagaattatgcagtgctgccataaccatgagtgataacactgcggccaacttacttctgacaacgatcggaggaccgaaggagctaaccgcttttttgcacaacatgggggatcatgtaactcgccttgatcgttgggaaccggagctgaatgaagccataccaaacgacgagcgtgacaccacgatgcctgtagcaatggcaacaacgttgcgcaaactattaactggcgaactacttactctagcttcccggcaacaattaatagactggatggaggcggataaagttgcaggaccacttctgcgctcggcccttccggctggctggtttattgctgataaatctggagccggtgagcgtgggtctcgcggtatcattgcagcactggggccagatggtaagccctcccgtatcgtagttatctacacgacggggagtcaggcaactatggatgaacgaaatagacagatcgctgagataggtgcctcactgattaagcattggtaactgtcagaccaagtttactcataacgcgtcaattcgagggggatcaattccgtgataggtgggctgcccttcctggttggcttggtttcatcagccatccgcttgccctcatctgttacgccggcggtagccggccagcctcgcagagcaggattcccgttgagcaccgccaggtgcgaataagggacagtgaagaaggaacacccgctcgcgggtgggcctacttcacctatcctgcccggctgacgccgttggatacaccaaggaaagtctacacgaaccctttggcaaaatcctgtatatcgtgcgaaaaaggatggatataccgaaaaaatcgctataatgaccccgaagcagggttatgcagcggaaaacggaattgatccggccacgatgcgtccggcgtagaggatctgaagatcagcagttcaacctgttgatagtacgtactaagctctcatgtttcacgtactaagctctcatgtttaacgtactaagctctcatgtttaacgaactaaaccctcatggctaacgtactaagctctcatggctaacgtactaagctctcatgtttcacgtactaagctctcatgtttgaacaataaaattaatataaatcagcaacttaaatagcctctaaggttttaagttttataagaaaaaaaagaatatataaggcttttaaagcttttaag

>pBH114

gtttaacggttgtggacaacaagccagggatgtaacgcactgagaagcccttagagcctctcaaagcaattttgagtgacacaggaacacttaacggctgacatgggaattcccctccaccgcggtggtacaaagaaaattcgacaaactgttatttttctatctatttatttgAATTttgAGCGCTcaaAATTtacctttgtcggcAATTttgAGCGCTcaaAATTaaataaagatattctcgtcaaacaaatataaataatataaacatggtttttactctggaagattttgttggcgattggcgtcagaccgcgggttataatttggatcaagtcctggaacagggtggcgtaagctctctgttccagaacctgggtgtgagcgtgacgccgattcagcgcatcgttctgtccggcgagaacggtctgaaaattgatattcatgtgatcatcccgtacgaaggcctgagcggtgaccaaatgggtcaaatcgagaaaatctttaaagtcgtctacccagttgacgatcaccacttcaaggttatcttgcattacggtacgctggtgattgatggtgtgaccccgaatatgattgactatttcggccgtccgtatgaaggcattgccgtttttgacggtaaaaagatcaccgtcaccggtaccctgtggaatggcaataagattattgacgagcgtctgattaacccggacggcagcctgctgttccgcgtgaccatcaacggtgtcacgggttggcgtctgtgcgagcgcatcctggcataatgaactgcacttgctttgataattaatgataaacaatctaaaagcactctaatcgttatcggagtgcttttagattactaatcaaattgcttctactaattgcctatcttccagtgatggaacagcatttgtgcattggctgcaacaatcagccttgatctggaagaagcaatgaaagctgctgttaagtctccgaatcaggtattgttcctgacaggtgtattcccatccggtaaacgcggatactttgcagttgatctgactcaggaataaattataaattaaggtaagaagattgtaggataagctaatgaaatagaaaaaggatgccgtcacacaacttgtcggcattcttttttgttttattagttgaaaatatagtgaaaaagttgcctaaatatgtatgttaacaaattatttgtcgtaactttgcactccaaatctgtttttaacatatggcactagtgAAACCAGTAACGTTATACGATGTCGCAGAGTATGCCGGTGTCTCTcatcagACCGTTTCCaatGTGGTGAACCAGGCCAGCCACGTTTCTGCGAAAACGCGGGAAAAAGTGGAAGCGGCGATGGCGGAGCTGAATTACATTCCCAACCGCGTGGCACAACAACTGGCGGGCAAACAGTCGTTGCTGATTGGCGTTGCCACCTCCAGTCTGGCCCTGCACGCGCTGTCGCAAATTGTCGCGGCGATTAAATCTCGCGCCTATCAACTGGGTGCCAGCGTGTTCGTGTCGATGGTAGAACGAAGCGGCATCGAAGCCTGTAAAACGGCGGTGCACAATCTTCTCGCGCAACGCGTCAGTGGGCTGATCATTAACTATCCGCTGGATAACCAGGATGCCATTGCTGTGGAAGCTGCCTGCACTAATGTTCCGGCGTTATTTCTTGATGTCTCTGACCAGACACCCATCAACAGTATTATTTTCTCCCATGAAGACGGTACGCGACTGGGCGTGGAGCATCTGGTCGCATTGGGTCACCAGCAAATCGCGCTGTTAGCGGGCCCATTAAGTTCTGTCTCGGCGCGTCTGCGTCTGGCTGGCTGGCATAAATATCTCACTCGCAATCAAATTCAGCCGATAGCGGAACGGGAAGGCGACTGGAGTGCCATGTCCGGTTTTCAACAAACCATGCAAATGCTGAATGAGGGCATCGTTCCCACTGCGATGCTGGTTGCCAACGATCAGATGGCGCTGGGCGCAATGCGCGCCATTACCGAGACCGGGCTGCGCGTTGGTGCGGATATCTCGGTAGTGGGATACGACGATACCGAAGACAGCTCATGTTATATCCCGCCGTTAACCACCATCAAACAGGATTTTCGCCTGCTGGGGCAAACCAGCGTGGACCGCTTGCTGCAACTCTCTCAGGGCCAGGCGGTGAAGGGCAATCAGCTGTTGCCCGTCTCACTGGTGAAAAGAAAAACCACCCTGGCGCCCAATACGCAAACCGCCTCTCCCCGCGCGTTGGCCGATTCATTAATGCAGCTGGCACGACAGGTTTCCCGACTGGAAAGCGGGCAGtgagctttcctcggtaccaaattccagaaaagaggcctcccgaaaggggggccttttttcgttttggtcctacttgtgcctgttctatttccgaaccgaccgcttgtatgaatccatcaaaattcgttttctctatgttggattccttgttgctcatattgtgatgataatttctacaaatatagtcattggtaactatctatgaaactgtttgatacttttatagttgattaaacttgttcatggcatttgccttaatatcatccgctatgtcaatgtagggtttcatagctttgtagtcgctgtgtcccgtccatttcatgaccacctgtgccgggattccgagagccagcgcattgcagatgaatgtccttcttcctgcatgggtactgagcaaagcgtatttgggtgtgacttcatcaatacgttcatttcccttgtagtaggtttcccgtacaggctcgttgatttctgccagttcgcccagctctttcaggtaatcgttcatcttctggttgctgatgacgggcagagccatgtaattctcgaaatggatgtccttgtatttgtccagtatggctttgctgtatttgttcagttcaatcgtcaggctgtcggcagtcttgactgtggttatttcgatgtggtcggacttcacatcgcttcttttcagattgcgaacatccgaataccgcaaactcgtaaagcagcagaacaggaaaacatcacgcacacgttccaggtattgcttatccttgggtatctggtagtctttcagcttgttcagttcatcccaagtcaggaagattacttttttcgaggtggttttcagtttcggtttgaacgtatcgtatgcaatgttctgatgatgtcctttcttgaagctccagcgcaggaaccatttgaggaatcccatttgcttgccgatggtgctgtttctcatatccttggtgtcacgcaggaagttgacgtattcgttcaatccaaactcgttgaaatagttgaacgttgcatcctccttgaactctttgaggtggttcctcactgctgcaaatttttcataggtggatgccgtccagttattctggttaccgcactcttttacaaactcatcgaacacctcccaaaagctgacaggggcttcttccggctgttcttcgctggtgtctttcattctcatgttgaaagcttccttcaactgttgggtcgttggcatgacctcctgcacctcaaattccttgaaaatattctggatttcggcatagtatttcagcaagtccgtattgatttcggctgcactttgctttagcttgttggtacatccgctctttacccgctgcttatctgcatcccatttggctacgtcaatccggtagcccgttgtaaactcgatgcgttggctggcaaagatgacacgcatacggatgggtacgttctctacgattggcacaccgttctttttccggctctccaatgcaaaaatgatgttgcgcttgatattcataattgggtgcgtttgaaattctacacccaaatatacacccaattattgagatagcaaaagacatttagaaacatttacttttactctatattgtaatttacacttgattatcagtcgtttgcagtcttatgatattctgtgaaagtataagttcgagagcctgtctctccgcaaaaaacgctgaaaatcagcagattgcaaaacaaacaccctgttttacacccaagaatgtaaagtcggctgtttttgttttatttaagataatacaaccactacataataaaagagtagcgatattaaaagaatccgatgagaaaagactaatatttatctatccattcagtttgatttttcaggactttacatcgtcctgaaagtatttgttggtaccggtaccgaggacgcgtaaacatttacagttgcatgtggcctattgtttttagccgttaaatattttataactattaaatagcgatacaaattgttcgaaactaatattgtttatatcatatattctcgcatgttttaaagctttattaaattgattttttgtaaacagtttttcgtactctttgttaacccatttcattacaaaagtttcatatttttttctctctttaaatgccatttttgctggctttctttttaatacaattaatgtgctatccactttaggttttggatggaaataatacctaggaatttttgctaatatagaaatatctacctctgccattaacagcaatgctagtgatctgtttgtatctaataacattttagcaaaaccatattccactattaaataacttattgtggctgaactttcaaaaacaatttttcgaattatatttgtgcttatgttgtaaggtatgctgccaaatattttatatggattgtggctaggaaatgtaaatttcagtatatcatcatttactatttgatagttaggataatttaagagcttattacgagttacctcacataatttagaatcaatttctatcgccgttacaaaattacatctctttaccaatccagcagtaaaatgacctttccctgcacctatttcaaagatgttatctttttcatctaaacttatgcaattcattattttttctatgtgatattttgaagtaataaaattttgactatcttttatatttactttgttcattataacctctccttaatttattgcatctcttttcgaatatttatgttttttgagaaaagaacgtactcatggttcatcccgatatgcgtatcggtctgtatatcagcaactttctatgtgtttcaactacaatagtcatctattctcatctttctgagtccaccccctgcaaagcccctctttacgacataaaaattcggtcggaaaaggtatgcaaaagatgtttctctctttaagagaaactcttcgggatgcaaaaatatgaaaataactccaattcaccaaattatatagcgacttttttacaaaatgctaaaatttgttgatttccgtcaagcaattgttgagcaaaaatgtcttttacgataaaatgatacctcaatatcaactgtttagcaaaacgatatttctcttaaagagagaaacacctttttgttcaccaatccccgacttttaatcccgcggccatgattgaaaaaggaagagtatgagtattcaacatttccgtgtcgcccttattcccttttttgcggcattttgccttcctgtttttgctcacccagaaacgctggtgaaagtaaaagatgctgaagatcagttgggtgcacgagtgggttacatcgaactggatctcaacagcggtaagatccttgagagttttcgccccgaagaacgttttccaatgatgagcacttttaaagttctgctatgtggcgcggtattatcccgtattgacgccgggcaagagcaactcggtcgccgcatacactattctcagaatgacttggttgagtactcaccagtcacagaaaagcatcttacggatggcatgacagtaagagaattatgcagtgctgccataaccatgagtgataacactgcggccaacttacttctgacaacgatcggaggaccgaaggagctaaccgcttttttgcacaacatgggggatcatgtaactcgccttgatcgttgggaaccggagctgaatgaagccataccaaacgacgagcgtgacaccacgatgcctgtagcaatggcaacaacgttgcgcaaactattaactggcgaactacttactctagcttcccggcaacaattaatagactggatggaggcggataaagttgcaggaccacttctgcgctcggcccttccggctggctggtttattgctgataaatctggagccggtgagcgtgggtctcgcggtatcattgcagcactggggccagatggtaagccctcccgtatcgtagttatctacacgacggggagtcaggcaactatggatgaacgaaatagacagatcgctgagataggtgcctcactgattaagcattggtaactgtcagaccaagtttactcataacgcgtcaattcgagggggatcaattccgtgataggtgggctgcccttcctggttggcttggtttcatcagccatccgcttgccctcatctgttacgccggcggtagccggccagcctcgcagagcaggattcccgttgagcaccgccaggtgcgaataagggacagtgaagaaggaacacccgctcgcgggtgggcctacttcacctatcctgcccggctgacgccgttggatacaccaaggaaagtctacacgaaccctttggcaaaatcctgtatatcgtgcgaaaaaggatggatataccgaaaaaatcgctataatgaccccgaagcagggttatgcagcggaaaacggaattgatccggccacgatgcgtccggcgtagaggatctgaagatcagcagttcaacctgttgatagtacgtactaagctctcatgtttcacgtactaagctctcatgtttaacgtactaagctctcatgtttaacgaactaaaccctcatggctaacgtactaagctctcatggctaacgtactaagctctcatgtttcacgtactaagctctcatgtttgaacaataaaattaatataaatcagcaacttaaatagcctctaaggttttaagttttataagaaaaaaaagaatatataaggcttttaaagcttttaag

>pBH115

gtttaacggttgtggacaacaagccagggatgtaacgcactgagaagcccttagagcctctcaaagcaattttgagtgacacaggaacacttaacggctgacatgggaattcccctccaccgcggtggtacaaagaaaattcgacaaactgttatttttctatctatttatttgAATTgacAGCGCTgtcAATTtacctttgtcggcAATTgacAGCGCTgtcAATTaaataaagatattctcgtcaaacaaatataaataatataaacatggtttttactctggaagattttgttggcgattggcgtcagaccgcgggttataatttggatcaagtcctggaacagggtggcgtaagctctctgttccagaacctgggtgtgagcgtgacgccgattcagcgcatcgttctgtccggcgagaacggtctgaaaattgatattcatgtgatcatcccgtacgaaggcctgagcggtgaccaaatgggtcaaatcgagaaaatctttaaagtcgtctacccagttgacgatcaccacttcaaggttatcttgcattacggtacgctggtgattgatggtgtgaccccgaatatgattgactatttcggccgtccgtatgaaggcattgccgtttttgacggtaaaaagatcaccgtcaccggtaccctgtggaatggcaataagattattgacgagcgtctgattaacccggacggcagcctgctgttccgcgtgaccatcaacggtgtcacgggttggcgtctgtgcgagcgcatcctggcataatgaactgcacttgctttgataattaatgataaacaatctaaaagcactctaatcgttatcggagtgcttttagattactaatcaaattgcttctactaattgcctatcttccagtgatggaacagcatttgtgcattggctgcaacaatcagccttgatctggaagaagcaatgaaagctgctgttaagtctccgaatcaggtattgttcctgacaggtgtattcccatccggtaaacgcggatactttgcagttgatctgactcaggaataaattataaattaaggtaagaagattgtaggataagctaatgaaatagaaaaaggatgccgtcacacaacttgtcggcattcttttttgttttattagttgaaaatatagtgaaaaagttgcctaaatatgtatgttaacaaattatttgtcgtaactttgcactccaaatctgtttttaacatatggcactagtgAAACCAGTAACGTTATACGATGTCGCAGAGTATGCCGGTGTCTCTggaaagACCGTTTCCcgcGTGGTGAACCAGGCCAGCCACGTTTCTGCGAAAACGCGGGAAAAAGTGGAAGCGGCGATGGCGGAGCTGAATTACATTCCCAACCGCGTGGCACAACAACTGGCGGGCAAACAGTCGTTGCTGATTGGCGTTGCCACCTCCAGTCTGGCCCTGCACGCGCTGTCGCAAATTGTCGCGGCGATTAAATCTCGCGCCTATCAACTGGGTGCCAGCGTGTTCGTGTCGATGGTAGAACGAAGCGGCATCGAAGCCTGTAAAACGGCGGTGCACAATCTTCTCGCGCAACGCGTCAGTGGGCTGATCATTAACTATCCGCTGGATAACCAGGATGCCATTGCTGTGGAAGCTGCCTGCACTAATGTTCCGGCGTTATTTCTTGATGTCTCTGACCAGACACCCATCAACAGTATTATTTTCTCCCATGAAGACGGTACGCGACTGGGCGTGGAGCATCTGGTCGCATTGGGTCACCAGCAAATCGCGCTGTTAGCGGGCCCATTAAGTTCTGTCTCGGCGCGTCTGCGTCTGGCTGGCTGGCATAAATATCTCACTCGCAATCAAATTCAGCCGATAGCGGAACGGGAAGGCGACTGGAGTGCCATGTCCGGTTTTCAACAAACCATGCAAATGCTGAATGAGGGCATCGTTCCCACTGCGATGCTGGTTGCCAACGATCAGATGGCGCTGGGCGCAATGCGCGCCATTACCGAGACCGGGCTGCGCGTTGGTGCGGATATCTCGGTAGTGGGATACGACGATACCGAAGACAGCTCATGTTATATCCCGCCGTTAACCACCATCAAACAGGATTTTCGCCTGCTGGGGCAAACCAGCGTGGACCGCTTGCTGCAACTCTCTCAGGGCCAGGCGGTGAAGGGCAATCAGCTGTTGCCCGTCTCACTGGTGAAAAGAAAAACCACCCTGGCGCCCAATACGCAAACCGCCTCTCCCCGCGCGTTGGCCGATTCATTAATGCAGCTGGCACGACAGGTTTCCCGACTGGAAAGCGGGCAGtgagctttcctcggtaccaaattccagaaaagaggcctcccgaaaggggggccttttttcgttttggtcctacttgtgcctgttctatttccgaaccgaccgcttgtatgaatccatcaaaattcgttttctctatgttggattccttgttgctcatattgtgatgataatttctacaaatatagtcattggtaactatctatgaaactgtttgatacttttatagttgattaaacttgttcatggcatttgccttaatatcatccgctatgtcaatgtagggtttcatagctttgtagtcgctgtgtcccgtccatttcatgaccacctgtgccgggattccgagagccagcgcattgcagatgaatgtccttcttcctgcatgggtactgagcaaagcgtatttgggtgtgacttcatcaatacgttcatttcccttgtagtaggtttcccgtacaggctcgttgatttctgccagttcgcccagctctttcaggtaatcgttcatcttctggttgctgatgacgggcagagccatgtaattctcgaaatggatgtccttgtatttgtccagtatggctttgctgtatttgttcagttcaatcgtcaggctgtcggcagtcttgactgtggttatttcgatgtggtcggacttcacatcgcttcttttcagattgcgaacatccgaataccgcaaactcgtaaagcagcagaacaggaaaacatcacgcacacgttccaggtattgcttatccttgggtatctggtagtctttcagcttgttcagttcatcccaagtcaggaagattacttttttcgaggtggttttcagtttcggtttgaacgtatcgtatgcaatgttctgatgatgtcctttcttgaagctccagcgcaggaaccatttgaggaatcccatttgcttgccgatggtgctgtttctcatatccttggtgtcacgcaggaagttgacgtattcgttcaatccaaactcgttgaaatagttgaacgttgcatcctccttgaactctttgaggtggttcctcactgctgcaaatttttcataggtggatgccgtccagttattctggttaccgcactcttttacaaactcatcgaacacctcccaaaagctgacaggggcttcttccggctgttcttcgctggtgtctttcattctcatgttgaaagcttccttcaactgttgggtcgttggcatgacctcctgcacctcaaattccttgaaaatattctggatttcggcatagtatttcagcaagtccgtattgatttcggctgcactttgctttagcttgttggtacatccgctctttacccgctgcttatctgcatcccatttggctacgtcaatccggtagcccgttgtaaactcgatgcgttggctggcaaagatgacacgcatacggatgggtacgttctctacgattggcacaccgttctttttccggctctccaatgcaaaaatgatgttgcgcttgatattcataattgggtgcgtttgaaattctacacccaaatatacacccaattattgagatagcaaaagacatttagaaacatttacttttactctatattgtaatttacacttgattatcagtcgtttgcagtcttatgatattctgtgaaagtataagttcgagagcctgtctctccgcaaaaaacgctgaaaatcagcagattgcaaaacaaacaccctgttttacacccaagaatgtaaagtcggctgtttttgttttatttaagataatacaaccactacataataaaagagtagcgatattaaaagaatccgatgagaaaagactaatatttatctatccattcagtttgatttttcaggactttacatcgtcctgaaagtatttgttggtaccggtaccgaggacgcgtaaacatttacagttgcatgtggcctattgtttttagccgttaaatattttataactattaaatagcgatacaaattgttcgaaactaatattgtttatatcatatattctcgcatgttttaaagctttattaaattgattttttgtaaacagtttttcgtactctttgttaacccatttcattacaaaagtttcatatttttttctctctttaaatgccatttttgctggctttctttttaatacaattaatgtgctatccactttaggttttggatggaaataatacctaggaatttttgctaatatagaaatatctacctctgccattaacagcaatgctagtgatctgtttgtatctaataacattttagcaaaaccatattccactattaaataacttattgtggctgaactttcaaaaacaatttttcgaattatatttgtgcttatgttgtaaggtatgctgccaaatattttatatggattgtggctaggaaatgtaaatttcagtatatcatcatttactatttgatagttaggataatttaagagcttattacgagttacctcacataatttagaatcaatttctatcgccgttacaaaattacatctctttaccaatccagcagtaaaatgacctttccctgcacctatttcaaagatgttatctttttcatctaaacttatgcaattcattattttttctatgtgatattttgaagtaataaaattttgactatcttttatatttactttgttcattataacctctccttaatttattgcatctcttttcgaatatttatgttttttgagaaaagaacgtactcatggttcatcccgatatgcgtatcggtctgtatatcagcaactttctatgtgtttcaactacaatagtcatctattctcatctttctgagtccaccccctgcaaagcccctctttacgacataaaaattcggtcggaaaaggtatgcaaaagatgtttctctctttaagagaaactcttcgggatgcaaaaatatgaaaataactccaattcaccaaattatatagcgacttttttacaaaatgctaaaatttgttgatttccgtcaagcaattgttgagcaaaaatgtcttttacgataaaatgatacctcaatatcaactgtttagcaaaacgatatttctcttaaagagagaaacacctttttgttcaccaatccccgacttttaatcccgcggccatgattgaaaaaggaagagtatgagtattcaacatttccgtgtcgcccttattcccttttttgcggcattttgccttcctgtttttgctcacccagaaacgctggtgaaagtaaaagatgctgaagatcagttgggtgcacgagtgggttacatcgaactggatctcaacagcggtaagatccttgagagttttcgccccgaagaacgttttccaatgatgagcacttttaaagttctgctatgtggcgcggtattatcccgtattgacgccgggcaagagcaactcggtcgccgcatacactattctcagaatgacttggttgagtactcaccagtcacagaaaagcatcttacggatggcatgacagtaagagaattatgcagtgctgccataaccatgagtgataacactgcggccaacttacttctgacaacgatcggaggaccgaaggagctaaccgcttttttgcacaacatgggggatcatgtaactcgccttgatcgttgggaaccggagctgaatgaagccataccaaacgacgagcgtgacaccacgatgcctgtagcaatggcaacaacgttgcgcaaactattaactggcgaactacttactctagcttcccggcaacaattaatagactggatggaggcggataaagttgcaggaccacttctgcgctcggcccttccggctggctggtttattgctgataaatctggagccggtgagcgtgggtctcgcggtatcattgcagcactggggccagatggtaagccctcccgtatcgtagttatctacacgacggggagtcaggcaactatggatgaacgaaatagacagatcgctgagataggtgcctcactgattaagcattggtaactgtcagaccaagtttactcataacgcgtcaattcgagggggatcaattccgtgataggtgggctgcccttcctggttggcttggtttcatcagccatccgcttgccctcatctgttacgccggcggtagccggccagcctcgcagagcaggattcccgttgagcaccgccaggtgcgaataagggacagtgaagaaggaacacccgctcgcgggtgggcctacttcacctatcctgcccggctgacgccgttggatacaccaaggaaagtctacacgaaccctttggcaaaatcctgtatatcgtgcgaaaaaggatggatataccgaaaaaatcgctataatgaccccgaagcagggttatgcagcggaaaacggaattgatccggccacgatgcgtccggcgtagaggatctgaagatcagcagttcaacctgttgatagtacgtactaagctctcatgtttcacgtactaagctctcatgtttaacgtactaagctctcatgtttaacgaactaaaccctcatggctaacgtactaagctctcatggctaacgtactaagctctcatgtttcacgtactaagctctcatgtttgaacaataaaattaatataaatcagcaacttaaatagcctctaaggttttaagttttataagaaaaaaaagaatatataaggcttttaaagcttttaag

>pBH116

gtttaacggttgtggacaacaagccagggatgtaacgcactgagaagcccttagagcctctcaaagcaattttgagtgacacaggaacacttaacggctgacatgggaattcccctccaccgcggtggtacaaagaaaattcgacaaactgttatttttctatctatttatttgAATTGTGAGCGGATAACAATTacctttgtcggcAATTGTGAGCGGATAACAATTaaataaagatattctcgtcaaacaaatataaataatataaacatggtttttactctggaagattttgttggcgattggcgtcagaccgcgggttataatttggatcaagtcctggaacagggtggcgtaagctctctgttccagaacctgggtgtgagcgtgacgccgattcagcgcatcgttctgtccggcgagaacggtctgaaaattgatattcatgtgatcatcccgtacgaaggcctgagcggtgaccaaatgggtcaaatcgagaaaatctttaaagtcgtctacccagttgacgatcaccacttcaaggttatcttgcattacggtacgctggtgattgatggtgtgaccccgaatatgattgactatttcggccgtccgtatgaaggcattgccgtttttgacggtaaaaagatcaccgtcaccggtaccctgtggaatggcaataagattattgacgagcgtctgattaacccggacggcagcctgctgttccgcgtgaccatcaacggtgtcacgggttggcgtctgtgcgagcgcatcctggcataatgaactgcacttgctttgataattaatgataaacaatctaaaagcactctaatcgttatcggagtgcttttagattactaatcaaattgcttctactaattgcctatcttccagtgatggaacagcatttgtgcattggctgcaacaatcagccttgatctggaagaagcaatgaaagctgctgttaagtctccgaatcaggtattgttcctgacaggtgtattcccatccggtaaacgcggatactttgcagttgatctgactcaggaataaattataaattaaggtaagaagattgtaggataagctaatgaaatagaaaaaggatgccgtcacacaacttgtcggcattcttttttgttttattagttgaaaatatagtgaaaaagttgcctaaatatgtatgttaacaaattatttgtcgtaactttgcactccaaatctgtttttaacatatggcactagtgAAACCAGTAACGTTATACGATGTCGCAGAGTATGCCGGTGTCTCTTATCAGACCGTTTCCCGCGTGGTGAACCAGGCCAGCCACGTTTCTGCGAAAACGCGGGAAAAAGTGGAAGCGGCGATGGCGGAGCTCAATTACATTCCCAACCGCGTGGCACAACAACTGGCGGGCAAAGCGTCGCATACCATTGGCATGTTGATCACTGCCAGTACCAATCCTTTCTATTCAGAACTGGTGCGTGGCGTTGAACGCAGCTGCTTCGAACGCGGTTATAGTCTCGatCTTTGCAATACCGAAGGCGATGAACAGCGGATGAATCGCAATCTGGAAACGCTGATGCAAAAACGCGTTGATGGCTTGCTGTTACTGTGCACCGAAACGCATCAACCTTCGCGTGAAATCATGCAACGTTATCCGACAGTGCCTACTGTGATGATGGACTGGGCTCCGTTCGATGGCGACAGCGATCTTATTCAGGATAACTCGTTGCTGGGCGGAGACTTAGCAACGCAATATCTGATCGATAAAGGTCATACCCGTATCGCCTGTATTACCGGCCCGCTGGATAAAACTCCGGCGCGCCTGCGGTTGGAAGGTTATCGGGCGGCGATGAAACGTGCGGGTCTCAACATTCCTGATGGCTATGAAGTCACTGGTGATTTTGAATTTAACGGCGGGTTTGACGCTATGCGCCAACTGCTATCACATCCGCTGCGTCCTCAGGCCGTCTTTACCGGAAATGACGCTATGGCTGTTGGCGTTTACCAGGCGTTATATCAGGCAGAGTTACAGGTTCCGCAGGATATCGCGGTGATTGGCTATGACGATATCGAACTGGCAAGCTTTATGACGCCACCATTAACCACTATCCACCAACCGAAAGATGAACTGGGGGAGCTGGCGATTGATGTACTCATCCATCGGATAACCCAGCCGACCCTTCAGCAACAACGATTACAACTTACTCCGATTCTGATGGAACGCGGTTCGGCTTAGCTGGTGAAAAGAAAAACCACCCTGGCGCCCAATACGCAAACCGCCTCTCCCCGCGCGTTGGCCGATTCATTAATGCAGCTGGCACGACAGGTTTCCCGACTGGAAAGCGGGCAGTGAgctttcctcggtaccaaattccagaaaagaggcctcccgaaaggggggccttttttcgttttggtcctacttgtgcctgttctatttccgaaccgaccgcttgtatgaatccatcaaaattcgttttctctatgttggattccttgttgctcatattgtgatgataatttctacaaatatagtcattggtaactatctatgaaactgtttgatacttttatagttgattaaacttgttcatggcatttgccttaatatcatccgctatgtcaatgtagggtttcatagctttgtagtcgctgtgtcccgtccatttcatgaccacctgtgccgggattccgagagccagcgcattgcagatgaatgtccttcttcctgcatgggtactgagcaaagcgtatttgggtgtgacttcatcaatacgttcatttcccttgtagtaggtttcccgtacaggctcgttgatttctgccagttcgcccagctctttcaggtaatcgttcatcttctggttgctgatgacgggcagagccatgtaattctcgaaatggatgtccttgtatttgtccagtatggctttgctgtatttgttcagttcaatcgtcaggctgtcggcagtcttgactgtggttatttcgatgtggtcggacttcacatcgcttcttttcagattgcgaacatccgaataccgcaaactcgtaaagcagcagaacaggaaaacatcacgcacacgttccaggtattgcttatccttgggtatctggtagtctttcagcttgttcagttcatcccaagtcaggaagattacttttttcgaggtggttttcagtttcggtttgaacgtatcgtatgcaatgttctgatgatgtcctttcttgaagctccagcgcaggaaccatttgaggaatcccatttgcttgccgatggtgctgtttctcatatccttggtgtcacgcaggaagttgacgtattcgttcaatccaaactcgttgaaatagttgaacgttgcatcctccttgaactctttgaggtggttcctcactgctgcaaatttttcataggtggatgccgtccagttattctggttaccgcactcttttacaaactcatcgaacacctcccaaaagctgacaggggcttcttccggctgttcttcgctggtgtctttcattctcatgttgaaagcttccttcaactgttgggtcgttggcatgacctcctgcacctcaaattccttgaaaatattctggatttcggcatagtatttcagcaagtccgtattgatttcggctgcactttgctttagcttgttggtacatccgctctttacccgctgcttatctgcatcccatttggctacgtcaatccggtagcccgttgtaaactcgatgcgttggctggcaaagatgacacgcatacggatgggtacgttctctacgattggcacaccgttctttttccggctctccaatgcaaaaatgatgttgcgcttgatattcataattgggtgcgtttgaaattctacacccaaatatacacccaattattgagatagcaaaagacatttagaaacatttacttttactctatattgtaatttacacttgattatcagtcgtttgcagtcttatgatattctgtgaaagtataagttcgagagcctgtctctccgcaaaaaacgctgaaaatcagcagattgcaaaacaaacaccctgttttacacccaagaatgtaaagtcggctgtttttgttttatttaagataatacaaccactacataataaaagagtagcgatattaaaagaatccgatgagaaaagactaatatttatctatccattcagtttgatttttcaggactttacatcgtcctgaaagtatttgttggtaccggtaccgaggacgcgtaaacatttacagttgcatgtggcctattgtttttagccgttaaatattttataactattaaatagcgatacaaattgttcgaaactaatattgtttatatcatatattctcgcatgttttaaagctttattaaattgattttttgtaaacagtttttcgtactctttgttaacccatttcattacaaaagtttcatatttttttctctctttaaatgccatttttgctggctttctttttaatacaattaatgtgctatccactttaggttttggatggaaataatacctaggaatttttgctaatatagaaatatctacctctgccattaacagcaatgctagtgatctgtttgtatctaataacattttagcaaaaccatattccactattaaataacttattgtggctgaactttcaaaaacaatttttcgaattatatttgtgcttatgttgtaaggtatgctgccaaatattttatatggattgtggctaggaaatgtaaatttcagtatatcatcatttactatttgatagttaggataatttaagagcttattacgagttacctcacataatttagaatcaatttctatcgccgttacaaaattacatctctttaccaatccagcagtaaaatgacctttccctgcacctatttcaaagatgttatctttttcatctaaacttatgcaattcattattttttctatgtgatattttgaagtaataaaattttgactatcttttatatttactttgttcattataacctctccttaatttattgcatctcttttcgaatatttatgttttttgagaaaagaacgtactcatggttcatcccgatatgcgtatcggtctgtatatcagcaactttctatgtgtttcaactacaatagtcatctattctcatctttctgagtccaccccctgcaaagcccctctttacgacataaaaattcggtcggaaaaggtatgcaaaagatgtttctctctttaagagaaactcttcgggatgcaaaaatatgaaaataactccaattcaccaaattatatagcgacttttttacaaaatgctaaaatttgttgatttccgtcaagcaattgttgagcaaaaatgtcttttacgataaaatgatacctcaatatcaactgtttagcaaaacgatatttctcttaaagagagaaacacctttttgttcaccaatccccgacttttaatcccgcggccatgattgaaaaaggaagagtatgagtattcaacatttccgtgtcgcccttattcccttttttgcggcattttgccttcctgtttttgctcacccagaaacgctggtgaaagtaaaagatgctgaagatcagttgggtgcacgagtgggttacatcgaactggatctcaacagcggtaagatccttgagagttttcgccccgaagaacgttttccaatgatgagcacttttaaagttctgctatgtggcgcggtattatcccgtattgacgccgggcaagagcaactcggtcgccgcatacactattctcagaatgacttggttgagtactcaccagtcacagaaaagcatcttacggatggcatgacagtaagagaattatgcagtgctgccataaccatgagtgataacactgcggccaacttacttctgacaacgatcggaggaccgaaggagctaaccgcttttttgcacaacatgggggatcatgtaactcgccttgatcgttgggaaccggagctgaatgaagccataccaaacgacgagcgtgacaccacgatgcctgtagcaatggcaacaacgttgcgcaaactattaactggcgaactacttactctagcttcccggcaacaattaatagactggatggaggcggataaagttgcaggaccacttctgcgctcggcccttccggctggctggtttattgctgataaatctggagccggtgagcgtgggtctcgcggtatcattgcagcactggggccagatggtaagccctcccgtatcgtagttatctacacgacggggagtcaggcaactatggatgaacgaaatagacagatcgctgagataggtgcctcactgattaagcattggtaactgtcagaccaagtttactcataacgcgtcaattcgagggggatcaattccgtgataggtgggctgcccttcctggttggcttggtttcatcagccatccgcttgccctcatctgttacgccggcggtagccggccagcctcgcagagcaggattcccgttgagcaccgccaggtgcgaataagggacagtgaagaaggaacacccgctcgcgggtgggcctacttcacctatcctgcccggctgacgccgttggatacaccaaggaaagtctacacgaaccctttggcaaaatcctgtatatcgtgcgaaaaaggatggatataccgaaaaaatcgctataatgaccccgaagcagggttatgcagcggaaaacggaattgatccggccacgatgcgtccggcgtagaggatctgaagatcagcagttcaacctgttgatagtacgtactaagctctcatgtttcacgtactaagctctcatgtttaacgtactaagctctcatgtttaacgaactaaaccctcatggctaacgtactaagctctcatggctaacgtactaagctctcatgtttcacgtactaagctctcatgtttgaacaataaaattaatataaatcagcaacttaaatagcctctaaggttttaagttttataagaaaaaaaagaatatataaggcttttaaagcttttaag

>pBH117

gtttaacggttgtggacaacaagccagggatgtaacgcactgagaagcccttagagcctctcaaagcaattttgagtgacacaggaacacttaacggctgacatgggaattcccctccaccgcggtggtacaaagaaaattcgacaaactgttatttttctatctatttatttgaattTTAAGCGCTTAAaatttacctttgtcggcaattTTAAGCGCTTAAaattaaataaagatattctcgtcaaacaaatataaataatataaacatggtttttactctggaagattttgttggcgattggcgtcagaccgcgggttataatttggatcaagtcctggaacagggtggcgtaagctctctgttccagaacctgggtgtgagcgtgacgccgattcagcgcatcgttctgtccggcgagaacggtctgaaaattgatattcatgtgatcatcccgtacgaaggcctgagcggtgaccaaatgggtcaaatcgagaaaatctttaaagtcgtctacccagttgacgatcaccacttcaaggttatcttgcattacggtacgctggtgattgatggtgtgaccccgaatatgattgactatttcggccgtccgtatgaaggcattgccgtttttgacggtaaaaagatcaccgtcaccggtaccctgtggaatggcaataagattattgacgagcgtctgattaacccggacggcagcctgctgttccgcgtgaccatcaacggtgtcacgggttggcgtctgtgcgagcgcatcctggcataatgaactgcacttgctttgataattaatgataaacaatctaaaagcactctaatcgttatcggagtgcttttagattactaatcaaattgcttctactaattgcctatcttccagtgatggaacagcatttgtgcattggctgcaacaatcagccttgatctggaagaagcaatgaaagctgctgttaagtctccgaatcaggtattgttcctgacaggtgtattcccatccggtaaacgcggatactttgcagttgatctgactcaggaataaattataaattaaggtaagaagattgtaggataagctaatgaaatagaaaaaggatgccgtcacacaacttgtcggcattcttttttgttttattagttgaaaatatagtgaaaaagttgcctaaatatgtatgttaacaaattatttgtcgtaactttgcactccaaatctgtttttaacatatggcactagtgAAACCAGTAACGTTATACGATGTCGCAGAGTATGCCGGTGTCTCTACCGCGACCGTTTCCAACGTGGTGAACCAGGCCAGCCACGTTTCTGCGAAAACGCGGGAAAAAGTGGAAGCGGCGATGGCGGAGCTCAATTACATTCCCAACCGCGTGGCACAACAACTGGCGGGCAAAGCGTCGCATACCATTGGCATGTTGATCACTGCCAGTACCAATCCTTTCTATTCAGAACTGGTGCGTGGCGTTGAACGCAGCTGCTTCGAACGCGGTTATAGTCTCGatCTTTGCAATACCGAAGGCGATGAACAGCGGATGAATCGCAATCTGGAAACGCTGATGCAAAAACGCGTTGATGGCTTGCTGTTACTGTGCACCGAAACGCATCAACCTTCGCGTGAAATCATGCAACGTTATCCGACAGTGCCTACTGTGATGATGGACTGGGCTCCGTTCGATGGCGACAGCGATCTTATTCAGGATAACTCGTTGCTGGGCGGAGACTTAGCAACGCAATATCTGATCGATAAAGGTCATACCCGTATCGCCTGTATTACCGGCCCGCTGGATAAAACTCCGGCGCGCCTGCGGTTGGAAGGTTATCGGGCGGCGATGAAACGTGCGGGTCTCAACATTCCTGATGGCTATGAAGTCACTGGTGATTTTGAATTTAACGGCGGGTTTGACGCTATGCGCCAACTGCTATCACATCCGCTGCGTCCTCAGGCCGTCTTTACCGGAAATGACGCTATGGCTGTTGGCGTTTACCAGGCGTTATATCAGGCAGAGTTACAGGTTCCGCAGGATATCGCGGTGATTGGCTATGACGATATCGAACTGGCAAGCTTTATGACGCCACCATTAACCACTATCCACCAACCGAAAGATGAACTGGGGGAGCTGGCGATTGATGTACTCATCCATCGGATAACCCAGCCGACCCTTCAGCAACAACGATTACAACTTACTCCGATTCTGATGGAACGCGGTTCGGCTTAGCTGGTGAAAAGAAAAACCACCCTGGCGCCCAATACGCAAACCGCCTCTCCCCGCGCGTTGGCCGATTCATTAATGCAGCTGGCACGACAGGTTTCCCGACTGGAAAGCGGGCAGTGAgctttcctcggtaccaaattccagaaaagaggcctcccgaaaggggggccttttttcgttttggtcctacttgtgcctgttctatttccgaaccgaccgcttgtatgaatccatcaaaattcgttttctctatgttggattccttgttgctcatattgtgatgataatttctacaaatatagtcattggtaactatctatgaaactgtttgatacttttatagttgattaaacttgttcatggcatttgccttaatatcatccgctatgtcaatgtagggtttcatagctttgtagtcgctgtgtcccgtccatttcatgaccacctgtgccgggattccgagagccagcgcattgcagatgaatgtccttcttcctgcatgggtactgagcaaagcgtatttgggtgtgacttcatcaatacgttcatttcccttgtagtaggtttcccgtacaggctcgttgatttctgccagttcgcccagctctttcaggtaatcgttcatcttctggttgctgatgacgggcagagccatgtaattctcgaaatggatgtccttgtatttgtccagtatggctttgctgtatttgttcagttcaatcgtcaggctgtcggcagtcttgactgtggttatttcgatgtggtcggacttcacatcgcttcttttcagattgcgaacatccgaataccgcaaactcgtaaagcagcagaacaggaaaacatcacgcacacgttccaggtattgcttatccttgggtatctggtagtctttcagcttgttcagttcatcccaagtcaggaagattacttttttcgaggtggttttcagtttcggtttgaacgtatcgtatgcaatgttctgatgatgtcctttcttgaagctccagcgcaggaaccatttgaggaatcccatttgcttgccgatggtgctgtttctcatatccttggtgtcacgcaggaagttgacgtattcgttcaatccaaactcgttgaaatagttgaacgttgcatcctccttgaactctttgaggtggttcctcactgctgcaaatttttcataggtggatgccgtccagttattctggttaccgcactcttttacaaactcatcgaacacctcccaaaagctgacaggggcttcttccggctgttcttcgctggtgtctttcattctcatgttgaaagcttccttcaactgttgggtcgttggcatgacctcctgcacctcaaattccttgaaaatattctggatttcggcatagtatttcagcaagtccgtattgatttcggctgcactttgctttagcttgttggtacatccgctctttacccgctgcttatctgcatcccatttggctacgtcaatccggtagcccgttgtaaactcgatgcgttggctggcaaagatgacacgcatacggatgggtacgttctctacgattggcacaccgttctttttccggctctccaatgcaaaaatgatgttgcgcttgatattcataattgggtgcgtttgaaattctacacccaaatatacacccaattattgagatagcaaaagacatttagaaacatttacttttactctatattgtaatttacacttgattatcagtcgtttgcagtcttatgatattctgtgaaagtataagttcgagagcctgtctctccgcaaaaaacgctgaaaatcagcagattgcaaaacaaacaccctgttttacacccaagaatgtaaagtcggctgtttttgttttatttaagataatacaaccactacataataaaagagtagcgatattaaaagaatccgatgagaaaagactaatatttatctatccattcagtttgatttttcaggactttacatcgtcctgaaagtatttgttggtaccggtaccgaggacgcgtaaacatttacagttgcatgtggcctattgtttttagccgttaaatattttataactattaaatagcgatacaaattgttcgaaactaatattgtttatatcatatattctcgcatgttttaaagctttattaaattgattttttgtaaacagtttttcgtactctttgttaacccatttcattacaaaagtttcatatttttttctctctttaaatgccatttttgctggctttctttttaatacaattaatgtgctatccactttaggttttggatggaaataatacctaggaatttttgctaatatagaaatatctacctctgccattaacagcaatgctagtgatctgtttgtatctaataacattttagcaaaaccatattccactattaaataacttattgtggctgaactttcaaaaacaatttttcgaattatatttgtgcttatgttgtaaggtatgctgccaaatattttatatggattgtggctaggaaatgtaaatttcagtatatcatcatttactatttgatagttaggataatttaagagcttattacgagttacctcacataatttagaatcaatttctatcgccgttacaaaattacatctctttaccaatccagcagtaaaatgacctttccctgcacctatttcaaagatgttatctttttcatctaaacttatgcaattcattattttttctatgtgatattttgaagtaataaaattttgactatcttttatatttactttgttcattataacctctccttaatttattgcatctcttttcgaatatttatgttttttgagaaaagaacgtactcatggttcatcccgatatgcgtatcggtctgtatatcagcaactttctatgtgtttcaactacaatagtcatctattctcatctttctgagtccaccccctgcaaagcccctctttacgacataaaaattcggtcggaaaaggtatgcaaaagatgtttctctctttaagagaaactcttcgggatgcaaaaatatgaaaataactccaattcaccaaattatatagcgacttttttacaaaatgctaaaatttgttgatttccgtcaagcaattgttgagcaaaaatgtcttttacgataaaatgatacctcaatatcaactgtttagcaaaacgatatttctcttaaagagagaaacacctttttgttcaccaatccccgacttttaatcccgcggccatgattgaaaaaggaagagtatgagtattcaacatttccgtgtcgcccttattcccttttttgcggcattttgccttcctgtttttgctcacccagaaacgctggtgaaagtaaaagatgctgaagatcagttgggtgcacgagtgggttacatcgaactggatctcaacagcggtaagatccttgagagttttcgccccgaagaacgttttccaatgatgagcacttttaaagttctgctatgtggcgcggtattatcccgtattgacgccgggcaagagcaactcggtcgccgcatacactattctcagaatgacttggttgagtactcaccagtcacagaaaagcatcttacggatggcatgacagtaagagaattatgcagtgctgccataaccatgagtgataacactgcggccaacttacttctgacaacgatcggaggaccgaaggagctaaccgcttttttgcacaacatgggggatcatgtaactcgccttgatcgttgggaaccggagctgaatgaagccataccaaacgacgagcgtgacaccacgatgcctgtagcaatggcaacaacgttgcgcaaactattaactggcgaactacttactctagcttcccggcaacaattaatagactggatggaggcggataaagttgcaggaccacttctgcgctcggcccttccggctggctggtttattgctgataaatctggagccggtgagcgtgggtctcgcggtatcattgcagcactggggccagatggtaagccctcccgtatcgtagttatctacacgacggggagtcaggcaactatggatgaacgaaatagacagatcgctgagataggtgcctcactgattaagcattggtaactgtcagaccaagtttactcataacgcgtcaattcgagggggatcaattccgtgataggtgggctgcccttcctggttggcttggtttcatcagccatccgcttgccctcatctgttacgccggcggtagccggccagcctcgcagagcaggattcccgttgagcaccgccaggtgcgaataagggacagtgaagaaggaacacccgctcgcgggtgggcctacttcacctatcctgcccggctgacgccgttggatacaccaaggaaagtctacacgaaccctttggcaaaatcctgtatatcgtgcgaaaaaggatggatataccgaaaaaatcgctataatgaccccgaagcagggttatgcagcggaaaacggaattgatccggccacgatgcgtccggcgtagaggatctgaagatcagcagttcaacctgttgatagtacgtactaagctctcatgtttcacgtactaagctctcatgtttaacgtactaagctctcatgtttaacgaactaaaccctcatggctaacgtactaagctctcatggctaacgtactaagctctcatgtttcacgtactaagctctcatgtttgaacaataaaattaatataaatcagcaacttaaatagcctctaaggttttaagttttataagaaaaaaaagaatatataaggcttttaaagcttttaag

>pBH118

gtttaacggttgtggacaacaagccagggatgtaacgcactgagaagcccttagagcctctcaaagcaattttgagtgacacaggaacacttaacggctgacatgggaattcccctccaccgcggtggtacaaagaaaattcgacaaactgttatttttctatctatttatttgAATTaggAGCGCTcctAATTtacctttgtcggcAATTaggAGCGCTcctAATTaaataaagatattctcgtcaaacaaatataaataatataaacatggtttttactctggaagattttgttggcgattggcgtcagaccgcgggttataatttggatcaagtcctggaacagggtggcgtaagctctctgttccagaacctgggtgtgagcgtgacgccgattcagcgcatcgttctgtccggcgagaacggtctgaaaattgatattcatgtgatcatcccgtacgaaggcctgagcggtgaccaaatgggtcaaatcgagaaaatctttaaagtcgtctacccagttgacgatcaccacttcaaggttatcttgcattacggtacgctggtgattgatggtgtgaccccgaatatgattgactatttcggccgtccgtatgaaggcattgccgtttttgacggtaaaaagatcaccgtcaccggtaccctgtggaatggcaataagattattgacgagcgtctgattaacccggacggcagcctgctgttccgcgtgaccatcaacggtgtcacgggttggcgtctgtgcgagcgcatcctggcataatgaactgcacttgctttgataattaatgataaacaatctaaaagcactctaatcgttatcggagtgcttttagattactaatcaaattgcttctactaattgcctatcttccagtgatggaacagcatttgtgcattggctgcaacaatcagccttgatctggaagaagcaatgaaagctgctgttaagtctccgaatcaggtattgttcctgacaggtgtattcccatccggtaaacgcggatactttgcagttgatctgactcaggaataaattataaattaaggtaagaagattgtaggataagctaatgaaatagaaaaaggatgccgtcacacaacttgtcggcattcttttttgttttattagttgaaaatatagtgaaaaagttgcctaaatatgtatgttaacaaattatttgtcgtaactttgcactccaaatctgtttttaacatatggcactagtgAAACCAGTAACGTTATACGATGTCGCAGAGTATGCCGGTGTCTCTAAAAGCACCGTTTCCCTGGTGGTGAACCAGGCCAGCCACGTTTCTGCGAAAACGCGGGAAAAAGTGGAAGCGGCGATGGCGGAGCTCAATTACATTCCCAACCGCGTGGCACAACAACTGGCGGGCAAAGCGTCGCATACCATTGGCATGTTGATCACTGCCAGTACCAATCCTTTCTATTCAGAACTGGTGCGTGGCGTTGAACGCAGCTGCTTCGAACGCGGTTATAGTCTCGatCTTTGCAATACCGAAGGCGATGAACAGCGGATGAATCGCAATCTGGAAACGCTGATGCAAAAACGCGTTGATGGCTTGCTGTTACTGTGCACCGAAACGCATCAACCTTCGCGTGAAATCATGCAACGTTATCCGACAGTGCCTACTGTGATGATGGACTGGGCTCCGTTCGATGGCGACAGCGATCTTATTCAGGATAACTCGTTGCTGGGCGGAGACTTAGCAACGCAATATCTGATCGATAAAGGTCATACCCGTATCGCCTGTATTACCGGCCCGCTGGATAAAACTCCGGCGCGCCTGCGGTTGGAAGGTTATCGGGCGGCGATGAAACGTGCGGGTCTCAACATTCCTGATGGCTATGAAGTCACTGGTGATTTTGAATTTAACGGCGGGTTTGACGCTATGCGCCAACTGCTATCACATCCGCTGCGTCCTCAGGCCGTCTTTACCGGAAATGACGCTATGGCTGTTGGCGTTTACCAGGCGTTATATCAGGCAGAGTTACAGGTTCCGCAGGATATCGCGGTGATTGGCTATGACGATATCGAACTGGCAAGCTTTATGACGCCACCATTAACCACTATCCACCAACCGAAAGATGAACTGGGGGAGCTGGCGATTGATGTACTCATCCATCGGATAACCCAGCCGACCCTTCAGCAACAACGATTACAACTTACTCCGATTCTGATGGAACGCGGTTCGGCTTAGCTGGTGAAAAGAAAAACCACCCTGGCGCCCAATACGCAAACCGCCTCTCCCCGCGCGTTGGCCGATTCATTAATGCAGCTGGCACGACAGGTTTCCCGACTGGAAAGCGGGCAGTGAgctttcctcggtaccaaattccagaaaagaggcctcccgaaaggggggccttttttcgttttggtcctacttgtgcctgttctatttccgaaccgaccgcttgtatgaatccatcaaaattcgttttctctatgttggattccttgttgctcatattgtgatgataatttctacaaatatagtcattggtaactatctatgaaactgtttgatacttttatagttgattaaacttgttcatggcatttgccttaatatcatccgctatgtcaatgtagggtttcatagctttgtagtcgctgtgtcccgtccatttcatgaccacctgtgccgggattccgagagccagcgcattgcagatgaatgtccttcttcctgcatgggtactgagcaaagcgtatttgggtgtgacttcatcaatacgttcatttcccttgtagtaggtttcccgtacaggctcgttgatttctgccagttcgcccagctctttcaggtaatcgttcatcttctggttgctgatgacgggcagagccatgtaattctcgaaatggatgtccttgtatttgtccagtatggctttgctgtatttgttcagttcaatcgtcaggctgtcggcagtcttgactgtggttatttcgatgtggtcggacttcacatcgcttcttttcagattgcgaacatccgaataccgcaaactcgtaaagcagcagaacaggaaaacatcacgcacacgttccaggtattgcttatccttgggtatctggtagtctttcagcttgttcagttcatcccaagtcaggaagattacttttttcgaggtggttttcagtttcggtttgaacgtatcgtatgcaatgttctgatgatgtcctttcttgaagctccagcgcaggaaccatttgaggaatcccatttgcttgccgatggtgctgtttctcatatccttggtgtcacgcaggaagttgacgtattcgttcaatccaaactcgttgaaatagttgaacgttgcatcctccttgaactctttgaggtggttcctcactgctgcaaatttttcataggtggatgccgtccagttattctggttaccgcactcttttacaaactcatcgaacacctcccaaaagctgacaggggcttcttccggctgttcttcgctggtgtctttcattctcatgttgaaagcttccttcaactgttgggtcgttggcatgacctcctgcacctcaaattccttgaaaatattctggatttcggcatagtatttcagcaagtccgtattgatttcggctgcactttgctttagcttgttggtacatccgctctttacccgctgcttatctgcatcccatttggctacgtcaatccggtagcccgttgtaaactcgatgcgttggctggcaaagatgacacgcatacggatgggtacgttctctacgattggcacaccgttctttttccggctctccaatgcaaaaatgatgttgcgcttgatattcataattgggtgcgtttgaaattctacacccaaatatacacccaattattgagatagcaaaagacatttagaaacatttacttttactctatattgtaatttacacttgattatcagtcgtttgcagtcttatgatattctgtgaaagtataagttcgagagcctgtctctccgcaaaaaacgctgaaaatcagcagattgcaaaacaaacaccctgttttacacccaagaatgtaaagtcggctgtttttgttttatttaagataatacaaccactacataataaaagagtagcgatattaaaagaatccgatgagaaaagactaatatttatctatccattcagtttgatttttcaggactttacatcgtcctgaaagtatttgttggtaccggtaccgaggacgcgtaaacatttacagttgcatgtggcctattgtttttagccgttaaatattttataactattaaatagcgatacaaattgttcgaaactaatattgtttatatcatatattctcgcatgttttaaagctttattaaattgattttttgtaaacagtttttcgtactctttgttaacccatttcattacaaaagtttcatatttttttctctctttaaatgccatttttgctggctttctttttaatacaattaatgtgctatccactttaggttttggatggaaataatacctaggaatttttgctaatatagaaatatctacctctgccattaacagcaatgctagtgatctgtttgtatctaataacattttagcaaaaccatattccactattaaataacttattgtggctgaactttcaaaaacaatttttcgaattatatttgtgcttatgttgtaaggtatgctgccaaatattttatatggattgtggctaggaaatgtaaatttcagtatatcatcatttactatttgatagttaggataatttaagagcttattacgagttacctcacataatttagaatcaatttctatcgccgttacaaaattacatctctttaccaatccagcagtaaaatgacctttccctgcacctatttcaaagatgttatctttttcatctaaacttatgcaattcattattttttctatgtgatattttgaagtaataaaattttgactatcttttatatttactttgttcattataacctctccttaatttattgcatctcttttcgaatatttatgttttttgagaaaagaacgtactcatggttcatcccgatatgcgtatcggtctgtatatcagcaactttctatgtgtttcaactacaatagtcatctattctcatctttctgagtccaccccctgcaaagcccctctttacgacataaaaattcggtcggaaaaggtatgcaaaagatgtttctctctttaagagaaactcttcgggatgcaaaaatatgaaaataactccaattcaccaaattatatagcgacttttttacaaaatgctaaaatttgttgatttccgtcaagcaattgttgagcaaaaatgtcttttacgataaaatgatacctcaatatcaactgtttagcaaaacgatatttctcttaaagagagaaacacctttttgttcaccaatccccgacttttaatcccgcggccatgattgaaaaaggaagagtatgagtattcaacatttccgtgtcgcccttattcccttttttgcggcattttgccttcctgtttttgctcacccagaaacgctggtgaaagtaaaagatgctgaagatcagttgggtgcacgagtgggttacatcgaactggatctcaacagcggtaagatccttgagagttttcgccccgaagaacgttttccaatgatgagcacttttaaagttctgctatgtggcgcggtattatcccgtattgacgccgggcaagagcaactcggtcgccgcatacactattctcagaatgacttggttgagtactcaccagtcacagaaaagcatcttacggatggcatgacagtaagagaattatgcagtgctgccataaccatgagtgataacactgcggccaacttacttctgacaacgatcggaggaccgaaggagctaaccgcttttttgcacaacatgggggatcatgtaactcgccttgatcgttgggaaccggagctgaatgaagccataccaaacgacgagcgtgacaccacgatgcctgtagcaatggcaacaacgttgcgcaaactattaactggcgaactacttactctagcttcccggcaacaattaatagactggatggaggcggataaagttgcaggaccacttctgcgctcggcccttccggctggctggtttattgctgataaatctggagccggtgagcgtgggtctcgcggtatcattgcagcactggggccagatggtaagccctcccgtatcgtagttatctacacgacggggagtcaggcaactatggatgaacgaaatagacagatcgctgagataggtgcctcactgattaagcattggtaactgtcagaccaagtttactcataacgcgtcaattcgagggggatcaattccgtgataggtgggctgcccttcctggttggcttggtttcatcagccatccgcttgccctcatctgttacgccggcggtagccggccagcctcgcagagcaggattcccgttgagcaccgccaggtgcgaataagggacagtgaagaaggaacacccgctcgcgggtgggcctacttcacctatcctgcccggctgacgccgttggatacaccaaggaaagtctacacgaaccctttggcaaaatcctgtatatcgtgcgaaaaaggatggatataccgaaaaaatcgctataatgaccccgaagcagggttatgcagcggaaaacggaattgatccggccacgatgcgtccggcgtagaggatctgaagatcagcagttcaacctgttgatagtacgtactaagctctcatgtttcacgtactaagctctcatgtttaacgtactaagctctcatgtttaacgaactaaaccctcatggctaacgtactaagctctcatggctaacgtactaagctctcatgtttcacgtactaagctctcatgtttgaacaataaaattaatataaatcagcaacttaaatagcctctaaggttttaagttttataagaaaaaaaagaatatataaggcttttaaagcttttaag

>pBH119

gtttaacggttgtggacaacaagccagggatgtaacgcactgagaagcccttagagcctctcaaagcaattttgagtgacacaggaacacttaacggctgacatgggaattcccctccaccgcggtggtacaaagaaaattcgacaaactgttatttttctatctatttatttgAATTttgAGCGCTcaaAATTtacctttgtcggcAATTttgAGCGCTcaaAATTaaataaagatattctcgtcaaacaaatataaataatataaacatggtttttactctggaagattttgttggcgattggcgtcagaccgcgggttataatttggatcaagtcctggaacagggtggcgtaagctctctgttccagaacctgggtgtgagcgtgacgccgattcagcgcatcgttctgtccggcgagaacggtctgaaaattgatattcatgtgatcatcccgtacgaaggcctgagcggtgaccaaatgggtcaaatcgagaaaatctttaaagtcgtctacccagttgacgatcaccacttcaaggttatcttgcattacggtacgctggtgattgatggtgtgaccccgaatatgattgactatttcggccgtccgtatgaaggcattgccgtttttgacggtaaaaagatcaccgtcaccggtaccctgtggaatggcaataagattattgacgagcgtctgattaacccggacggcagcctgctgttccgcgtgaccatcaacggtgtcacgggttggcgtctgtgcgagcgcatcctggcataatgaactgcacttgctttgataattaatgataaacaatctaaaagcactctaatcgttatcggagtgcttttagattactaatcaaattgcttctactaattgcctatcttccagtgatggaacagcatttgtgcattggctgcaacaatcagccttgatctggaagaagcaatgaaagctgctgttaagtctccgaatcaggtattgttcctgacaggtgtattcccatccggtaaacgcggatactttgcagttgatctgactcaggaataaattataaattaaggtaagaagattgtaggataagctaatgaaatagaaaaaggatgccgtcacacaacttgtcggcattcttttttgttttattagttgaaaatatagtgaaaaagttgcctaaatatgtatgttaacaaattatttgtcgtaactttgcactccaaatctgtttttaacatatggcactagtgAAACCAGTAACGTTATACGATGTCGCAGAGTATGCCGGTGTCTCTcatcagACCGTTTCCaatGTGGTGAACCAGGCCAGCCACGTTTCTGCGAAAACGCGGGAAAAAGTGGAAGCGGCGATGGCGGAGCTCAATTACATTCCCAACCGCGTGGCACAACAACTGGCGGGCAAAGCGTCGCATACCATTGGCATGTTGATCACTGCCAGTACCAATCCTTTCTATTCAGAACTGGTGCGTGGCGTTGAACGCAGCTGCTTCGAACGCGGTTATAGTCTCGatCTTTGCAATACCGAAGGCGATGAACAGCGGATGAATCGCAATCTGGAAACGCTGATGCAAAAACGCGTTGATGGCTTGCTGTTACTGTGCACCGAAACGCATCAACCTTCGCGTGAAATCATGCAACGTTATCCGACAGTGCCTACTGTGATGATGGACTGGGCTCCGTTCGATGGCGACAGCGATCTTATTCAGGATAACTCGTTGCTGGGCGGAGACTTAGCAACGCAATATCTGATCGATAAAGGTCATACCCGTATCGCCTGTATTACCGGCCCGCTGGATAAAACTCCGGCGCGCCTGCGGTTGGAAGGTTATCGGGCGGCGATGAAACGTGCGGGTCTCAACATTCCTGATGGCTATGAAGTCACTGGTGATTTTGAATTTAACGGCGGGTTTGACGCTATGCGCCAACTGCTATCACATCCGCTGCGTCCTCAGGCCGTCTTTACCGGAAATGACGCTATGGCTGTTGGCGTTTACCAGGCGTTATATCAGGCAGAGTTACAGGTTCCGCAGGATATCGCGGTGATTGGCTATGACGATATCGAACTGGCAAGCTTTATGACGCCACCATTAACCACTATCCACCAACCGAAAGATGAACTGGGGGAGCTGGCGATTGATGTACTCATCCATCGGATAACCCAGCCGACCCTTCAGCAACAACGATTACAACTTACTCCGATTCTGATGGAACGCGGTTCGGCTTAGCTGGTGAAAAGAAAAACCACCCTGGCGCCCAATACGCAAACCGCCTCTCCCCGCGCGTTGGCCGATTCATTAATGCAGCTGGCACGACAGGTTTCCCGACTGGAAAGCGGGCAGTGAgctttcctcggtaccaaattccagaaaagaggcctcccgaaaggggggccttttttcgttttggtcctacttgtgcctgttctatttccgaaccgaccgcttgtatgaatccatcaaaattcgttttctctatgttggattccttgttgctcatattgtgatgataatttctacaaatatagtcattggtaactatctatgaaactgtttgatacttttatagttgattaaacttgttcatggcatttgccttaatatcatccgctatgtcaatgtagggtttcatagctttgtagtcgctgtgtcccgtccatttcatgaccacctgtgccgggattccgagagccagcgcattgcagatgaatgtccttcttcctgcatgggtactgagcaaagcgtatttgggtgtgacttcatcaatacgttcatttcccttgtagtaggtttcccgtacaggctcgttgatttctgccagttcgcccagctctttcaggtaatcgttcatcttctggttgctgatgacgggcagagccatgtaattctcgaaatggatgtccttgtatttgtccagtatggctttgctgtatttgttcagttcaatcgtcaggctgtcggcagtcttgactgtggttatttcgatgtggtcggacttcacatcgcttcttttcagattgcgaacatccgaataccgcaaactcgtaaagcagcagaacaggaaaacatcacgcacacgttccaggtattgcttatccttgggtatctggtagtctttcagcttgttcagttcatcccaagtcaggaagattacttttttcgaggtggttttcagtttcggtttgaacgtatcgtatgcaatgttctgatgatgtcctttcttgaagctccagcgcaggaaccatttgaggaatcccatttgcttgccgatggtgctgtttctcatatccttggtgtcacgcaggaagttgacgtattcgttcaatccaaactcgttgaaatagttgaacgttgcatcctccttgaactctttgaggtggttcctcactgctgcaaatttttcataggtggatgccgtccagttattctggttaccgcactcttttacaaactcatcgaacacctcccaaaagctgacaggggcttcttccggctgttcttcgctggtgtctttcattctcatgttgaaagcttccttcaactgttgggtcgttggcatgacctcctgcacctcaaattccttgaaaatattctggatttcggcatagtatttcagcaagtccgtattgatttcggctgcactttgctttagcttgttggtacatccgctctttacccgctgcttatctgcatcccatttggctacgtcaatccggtagcccgttgtaaactcgatgcgttggctggcaaagatgacacgcatacggatgggtacgttctctacgattggcacaccgttctttttccggctctccaatgcaaaaatgatgttgcgcttgatattcataattgggtgcgtttgaaattctacacccaaatatacacccaattattgagatagcaaaagacatttagaaacatttacttttactctatattgtaatttacacttgattatcagtcgtttgcagtcttatgatattctgtgaaagtataagttcgagagcctgtctctccgcaaaaaacgctgaaaatcagcagattgcaaaacaaacaccctgttttacacccaagaatgtaaagtcggctgtttttgttttatttaagataatacaaccactacataataaaagagtagcgatattaaaagaatccgatgagaaaagactaatatttatctatccattcagtttgatttttcaggactttacatcgtcctgaaagtatttgttggtaccggtaccgaggacgcgtaaacatttacagttgcatgtggcctattgtttttagccgttaaatattttataactattaaatagcgatacaaattgttcgaaactaatattgtttatatcatatattctcgcatgttttaaagctttattaaattgattttttgtaaacagtttttcgtactctttgttaacccatttcattacaaaagtttcatatttttttctctctttaaatgccatttttgctggctttctttttaatacaattaatgtgctatccactttaggttttggatggaaataatacctaggaatttttgctaatatagaaatatctacctctgccattaacagcaatgctagtgatctgtttgtatctaataacattttagcaaaaccatattccactattaaataacttattgtggctgaactttcaaaaacaatttttcgaattatatttgtgcttatgttgtaaggtatgctgccaaatattttatatggattgtggctaggaaatgtaaatttcagtatatcatcatttactatttgatagttaggataatttaagagcttattacgagttacctcacataatttagaatcaatttctatcgccgttacaaaattacatctctttaccaatccagcagtaaaatgacctttccctgcacctatttcaaagatgttatctttttcatctaaacttatgcaattcattattttttctatgtgatattttgaagtaataaaattttgactatcttttatatttactttgttcattataacctctccttaatttattgcatctcttttcgaatatttatgttttttgagaaaagaacgtactcatggttcatcccgatatgcgtatcggtctgtatatcagcaactttctatgtgtttcaactacaatagtcatctattctcatctttctgagtccaccccctgcaaagcccctctttacgacataaaaattcggtcggaaaaggtatgcaaaagatgtttctctctttaagagaaactcttcgggatgcaaaaatatgaaaataactccaattcaccaaattatatagcgacttttttacaaaatgctaaaatttgttgatttccgtcaagcaattgttgagcaaaaatgtcttttacgataaaatgatacctcaatatcaactgtttagcaaaacgatatttctcttaaagagagaaacacctttttgttcaccaatccccgacttttaatcccgcggccatgattgaaaaaggaagagtatgagtattcaacatttccgtgtcgcccttattcccttttttgcggcattttgccttcctgtttttgctcacccagaaacgctggtgaaagtaaaagatgctgaagatcagttgggtgcacgagtgggttacatcgaactggatctcaacagcggtaagatccttgagagttttcgccccgaagaacgttttccaatgatgagcacttttaaagttctgctatgtggcgcggtattatcccgtattgacgccgggcaagagcaactcggtcgccgcatacactattctcagaatgacttggttgagtactcaccagtcacagaaaagcatcttacggatggcatgacagtaagagaattatgcagtgctgccataaccatgagtgataacactgcggccaacttacttctgacaacgatcggaggaccgaaggagctaaccgcttttttgcacaacatgggggatcatgtaactcgccttgatcgttgggaaccggagctgaatgaagccataccaaacgacgagcgtgacaccacgatgcctgtagcaatggcaacaacgttgcgcaaactattaactggcgaactacttactctagcttcccggcaacaattaatagactggatggaggcggataaagttgcaggaccacttctgcgctcggcccttccggctggctggtttattgctgataaatctggagccggtgagcgtgggtctcgcggtatcattgcagcactggggccagatggtaagccctcccgtatcgtagttatctacacgacggggagtcaggcaactatggatgaacgaaatagacagatcgctgagataggtgcctcactgattaagcattggtaactgtcagaccaagtttactcataacgcgtcaattcgagggggatcaattccgtgataggtgggctgcccttcctggttggcttggtttcatcagccatccgcttgccctcatctgttacgccggcggtagccggccagcctcgcagagcaggattcccgttgagcaccgccaggtgcgaataagggacagtgaagaaggaacacccgctcgcgggtgggcctacttcacctatcctgcccggctgacgccgttggatacaccaaggaaagtctacacgaaccctttggcaaaatcctgtatatcgtgcgaaaaaggatggatataccgaaaaaatcgctataatgaccccgaagcagggttatgcagcggaaaacggaattgatccggccacgatgcgtccggcgtagaggatctgaagatcagcagttcaacctgttgatagtacgtactaagctctcatgtttcacgtactaagctctcatgtttaacgtactaagctctcatgtttaacgaactaaaccctcatggctaacgtactaagctctcatggctaacgtactaagctctcatgtttcacgtactaagctctcatgtttgaacaataaaattaatataaatcagcaacttaaatagcctctaaggttttaagttttataagaaaaaaaagaatatataaggcttttaaagcttttaag

>pBH120

gtttaacggttgtggacaacaagccagggatgtaacgcactgagaagcccttagagcctctcaaagcaattttgagtgacacaggaacacttaacggctgacatgggaattcccctccaccgcggtggtacaaagaaaattcgacaaactgttatttttctatctatttatttgAATTgacAGCGCTgtcAATTtacctttgtcggcAATTgacAGCGCTgtcAATTaaataaagatattctcgtcaaacaaatataaataatataaacatggtttttactctggaagattttgttggcgattggcgtcagaccgcgggttataatttggatcaagtcctggaacagggtggcgtaagctctctgttccagaacctgggtgtgagcgtgacgccgattcagcgcatcgttctgtccggcgagaacggtctgaaaattgatattcatgtgatcatcccgtacgaaggcctgagcggtgaccaaatgggtcaaatcgagaaaatctttaaagtcgtctacccagttgacgatcaccacttcaaggttatcttgcattacggtacgctggtgattgatggtgtgaccccgaatatgattgactatttcggccgtccgtatgaaggcattgccgtttttgacggtaaaaagatcaccgtcaccggtaccctgtggaatggcaataagattattgacgagcgtctgattaacccggacggcagcctgctgttccgcgtgaccatcaacggtgtcacgggttggcgtctgtgcgagcgcatcctggcataatgaactgcacttgctttgataattaatgataaacaatctaaaagcactctaatcgttatcggagtgcttttagattactaatcaaattgcttctactaattgcctatcttccagtgatggaacagcatttgtgcattggctgcaacaatcagccttgatctggaagaagcaatgaaagctgctgttaagtctccgaatcaggtattgttcctgacaggtgtattcccatccggtaaacgcggatactttgcagttgatctgactcaggaataaattataaattaaggtaagaagattgtaggataagctaatgaaatagaaaaaggatgccgtcacacaacttgtcggcattcttttttgttttattagttgaaaatatagtgaaaaagttgcctaaatatgtatgttaacaaattatttgtcgtaactttgcactccaaatctgtttttaacatatggcactagtgAAACCAGTAACGTTATACGATGTCGCAGAGTATGCCGGTGTCTCTggaaagACCGTTTCCcgcGTGGTGAACCAGGCCAGCCACGTTTCTGCGAAAACGCGGGAAAAAGTGGAAGCGGCGATGGCGGAGCTCAATTACATTCCCAACCGCGTGGCACAACAACTGGCGGGCAAAGCGTCGCATACCATTGGCATGTTGATCACTGCCAGTACCAATCCTTTCTATTCAGAACTGGTGCGTGGCGTTGAACGCAGCTGCTTCGAACGCGGTTATAGTCTCGatCTTTGCAATACCGAAGGCGATGAACAGCGGATGAATCGCAATCTGGAAACGCTGATGCAAAAACGCGTTGATGGCTTGCTGTTACTGTGCACCGAAACGCATCAACCTTCGCGTGAAATCATGCAACGTTATCCGACAGTGCCTACTGTGATGATGGACTGGGCTCCGTTCGATGGCGACAGCGATCTTATTCAGGATAACTCGTTGCTGGGCGGAGACTTAGCAACGCAATATCTGATCGATAAAGGTCATACCCGTATCGCCTGTATTACCGGCCCGCTGGATAAAACTCCGGCGCGCCTGCGGTTGGAAGGTTATCGGGCGGCGATGAAACGTGCGGGTCTCAACATTCCTGATGGCTATGAAGTCACTGGTGATTTTGAATTTAACGGCGGGTTTGACGCTATGCGCCAACTGCTATCACATCCGCTGCGTCCTCAGGCCGTCTTTACCGGAAATGACGCTATGGCTGTTGGCGTTTACCAGGCGTTATATCAGGCAGAGTTACAGGTTCCGCAGGATATCGCGGTGATTGGCTATGACGATATCGAACTGGCAAGCTTTATGACGCCACCATTAACCACTATCCACCAACCGAAAGATGAACTGGGGGAGCTGGCGATTGATGTACTCATCCATCGGATAACCCAGCCGACCCTTCAGCAACAACGATTACAACTTACTCCGATTCTGATGGAACGCGGTTCGGCTTAGCTGGTGAAAAGAAAAACCACCCTGGCGCCCAATACGCAAACCGCCTCTCCCCGCGCGTTGGCCGATTCATTAATGCAGCTGGCACGACAGGTTTCCCGACTGGAAAGCGGGCAGTGAgctttcctcggtaccaaattccagaaaagaggcctcccgaaaggggggccttttttcgttttggtcctacttgtgcctgttctatttccgaaccgaccgcttgtatgaatccatcaaaattcgttttctctatgttggattccttgttgctcatattgtgatgataatttctacaaatatagtcattggtaactatctatgaaactgtttgatacttttatagttgattaaacttgttcatggcatttgccttaatatcatccgctatgtcaatgtagggtttcatagctttgtagtcgctgtgtcccgtccatttcatgaccacctgtgccgggattccgagagccagcgcattgcagatgaatgtccttcttcctgcatgggtactgagcaaagcgtatttgggtgtgacttcatcaatacgttcatttcccttgtagtaggtttcccgtacaggctcgttgatttctgccagttcgcccagctctttcaggtaatcgttcatcttctggttgctgatgacgggcagagccatgtaattctcgaaatggatgtccttgtatttgtccagtatggctttgctgtatttgttcagttcaatcgtcaggctgtcggcagtcttgactgtggttatttcgatgtggtcggacttcacatcgcttcttttcagattgcgaacatccgaataccgcaaactcgtaaagcagcagaacaggaaaacatcacgcacacgttccaggtattgcttatccttgggtatctggtagtctttcagcttgttcagttcatcccaagtcaggaagattacttttttcgaggtggttttcagtttcggtttgaacgtatcgtatgcaatgttctgatgatgtcctttcttgaagctccagcgcaggaaccatttgaggaatcccatttgcttgccgatggtgctgtttctcatatccttggtgtcacgcaggaagttgacgtattcgttcaatccaaactcgttgaaatagttgaacgttgcatcctccttgaactctttgaggtggttcctcactgctgcaaatttttcataggtggatgccgtccagttattctggttaccgcactcttttacaaactcatcgaacacctcccaaaagctgacaggggcttcttccggctgttcttcgctggtgtctttcattctcatgttgaaagcttccttcaactgttgggtcgttggcatgacctcctgcacctcaaattccttgaaaatattctggatttcggcatagtatttcagcaagtccgtattgatttcggctgcactttgctttagcttgttggtacatccgctctttacccgctgcttatctgcatcccatttggctacgtcaatccggtagcccgttgtaaactcgatgcgttggctggcaaagatgacacgcatacggatgggtacgttctctacgattggcacaccgttctttttccggctctccaatgcaaaaatgatgttgcgcttgatattcataattgggtgcgtttgaaattctacacccaaatatacacccaattattgagatagcaaaagacatttagaaacatttacttttactctatattgtaatttacacttgattatcagtcgtttgcagtcttatgatattctgtgaaagtataagttcgagagcctgtctctccgcaaaaaacgctgaaaatcagcagattgcaaaacaaacaccctgttttacacccaagaatgtaaagtcggctgtttttgttttatttaagataatacaaccactacataataaaagagtagcgatattaaaagaatccgatgagaaaagactaatatttatctatccattcagtttgatttttcaggactttacatcgtcctgaaagtatttgttggtaccggtaccgaggacgcgtaaacatttacagttgcatgtggcctattgtttttagccgttaaatattttataactattaaatagcgatacaaattgttcgaaactaatattgtttatatcatatattctcgcatgttttaaagctttattaaattgattttttgtaaacagtttttcgtactctttgttaacccatttcattacaaaagtttcatatttttttctctctttaaatgccatttttgctggctttctttttaatacaattaatgtgctatccactttaggttttggatggaaataatacctaggaatttttgctaatatagaaatatctacctctgccattaacagcaatgctagtgatctgtttgtatctaataacattttagcaaaaccatattccactattaaataacttattgtggctgaactttcaaaaacaatttttcgaattatatttgtgcttatgttgtaaggtatgctgccaaatattttatatggattgtggctaggaaatgtaaatttcagtatatcatcatttactatttgatagttaggataatttaagagcttattacgagttacctcacataatttagaatcaatttctatcgccgttacaaaattacatctctttaccaatccagcagtaaaatgacctttccctgcacctatttcaaagatgttatctttttcatctaaacttatgcaattcattattttttctatgtgatattttgaagtaataaaattttgactatcttttatatttactttgttcattataacctctccttaatttattgcatctcttttcgaatatttatgttttttgagaaaagaacgtactcatggttcatcccgatatgcgtatcggtctgtatatcagcaactttctatgtgtttcaactacaatagtcatctattctcatctttctgagtccaccccctgcaaagcccctctttacgacataaaaattcggtcggaaaaggtatgcaaaagatgtttctctctttaagagaaactcttcgggatgcaaaaatatgaaaataactccaattcaccaaattatatagcgacttttttacaaaatgctaaaatttgttgatttccgtcaagcaattgttgagcaaaaatgtcttttacgataaaatgatacctcaatatcaactgtttagcaaaacgatatttctcttaaagagagaaacacctttttgttcaccaatccccgacttttaatcccgcggccatgattgaaaaaggaagagtatgagtattcaacatttccgtgtcgcccttattcccttttttgcggcattttgccttcctgtttttgctcacccagaaacgctggtgaaagtaaaagatgctgaagatcagttgggtgcacgagtgggttacatcgaactggatctcaacagcggtaagatccttgagagttttcgccccgaagaacgttttccaatgatgagcacttttaaagttctgctatgtggcgcggtattatcccgtattgacgccgggcaagagcaactcggtcgccgcatacactattctcagaatgacttggttgagtactcaccagtcacagaaaagcatcttacggatggcatgacagtaagagaattatgcagtgctgccataaccatgagtgataacactgcggccaacttacttctgacaacgatcggaggaccgaaggagctaaccgcttttttgcacaacatgggggatcatgtaactcgccttgatcgttgggaaccggagctgaatgaagccataccaaacgacgagcgtgacaccacgatgcctgtagcaatggcaacaacgttgcgcaaactattaactggcgaactacttactctagcttcccggcaacaattaatagactggatggaggcggataaagttgcaggaccacttctgcgctcggcccttccggctggctggtttattgctgataaatctggagccggtgagcgtgggtctcgcggtatcattgcagcactggggccagatggtaagccctcccgtatcgtagttatctacacgacggggagtcaggcaactatggatgaacgaaatagacagatcgctgagataggtgcctcactgattaagcattggtaactgtcagaccaagtttactcataacgcgtcaattcgagggggatcaattccgtgataggtgggctgcccttcctggttggcttggtttcatcagccatccgcttgccctcatctgttacgccggcggtagccggccagcctcgcagagcaggattcccgttgagcaccgccaggtgcgaataagggacagtgaagaaggaacacccgctcgcgggtgggcctacttcacctatcctgcccggctgacgccgttggatacaccaaggaaagtctacacgaaccctttggcaaaatcctgtatatcgtgcgaaaaaggatggatataccgaaaaaatcgctataatgaccccgaagcagggttatgcagcggaaaacggaattgatccggccacgatgcgtccggcgtagaggatctgaagatcagcagttcaacctgttgatagtacgtactaagctctcatgtttcacgtactaagctctcatgtttaacgtactaagctctcatgtttaacgaactaaaccctcatggctaacgtactaagctctcatggctaacgtactaagctctcatgtttcacgtactaagctctcatgtttgaacaataaaattaatataaatcagcaacttaaatagcctctaaggttttaagttttataagaaaaaaaagaatatataaggcttttaaagcttttaag

>pBH201

gtttaacggttgtggacaacaagccagggatgtaacgcactgagaagcccttagagcctctcaaagcaattttgagtgacacaggaacacttaacggctgacatgggaattcccctccaccgcggtggtacaaagaaaattcgacaaactgttatttttctatctatttatttgAATTGTGAGCGGATAACAATTacctttgtcggcAATTGTGAGCGGATAACAATTaaataaagatattctcgtcaaacaaatataaataatataaacatggtttttactctggaagattttgttggcgattggcgtcagaccgcgggttataatttggatcaagtcctggaacagggtggcgtaagctctctgttccagaacctgggtgtgagcgtgacgccgattcagcgcatcgttctgtccggcgagaacggtctgaaaattgatattcatgtgatcatcccgtacgaaggcctgagcggtgaccaaatgggtcaaatcgagaaaatctttaaagtcgtctacccagttgacgatcaccacttcaaggttatcttgcattacggtacgctggtgattgatggtgtgaccccgaatatgattgactatttcggccgtccgtatgaaggcattgccgtttttgacggtaaaaagatcaccgtcaccggtaccctgtggaatggcaataagattattgacgagcgtctgattaacccggacggcagcctgctgttccgcgtgaccatcaacggtgtcacgggttggcgtctgtgcgagcgcatcctggcataatgaactgcacttgctttgataattaatgataaacaatctaaaagcactctaatcgttatcggagtgcttttagattactaatcaaattgcttctactaattgcctatcttccagtgatggaacagcatttgtgcattggctgcaacaatcagccttgatctggaagaagcaatgaaagctgctgttaagtctccgaatcaggtattgttcctgacaggtgtattcccatccggtaaacgcggatactttgcagttgatctgactcaggaataaattataaattaaggtaagaagattgtaggataagctaatgaaatagaaaaaggatgccgtcacacaacttgtcggcattcttttttgttttattagttgaaaatatagtgaaaaagttgcctaaatatgtatgttaacaaattatttgtcgtaactttgcactccaaatctgtttttaacatatggcactagtgAAACCAGTAACGTTATACGATGTCGCAGAGTATGCCGGTGTCTCTTATCAGACCGTTTCCCGCGTGGTGAACCAGGCCAGCCACGTTTCTGCGAAAACGCGGGAAAAAGTGGAAGCGGCGATGGCGGAGCTGAATTACATTCCCAACCGCGTGGCACAACAACTGGCGGGCAAACAGTCGTTGCTGATTGGCGTTGCCACCTCCAGTCTGGCCCTGCACGCGCCGTCGCAAATTGTCGCGGCGATTAAATCTCGCGCCGATCAACTGGGTGCCAGCGTGGTGGTGTCGATGGTAGAACGAAGCGGCGTCGAAGCCTGTAAAACGGCGGTGCACAATCTTCTCGCGCAACGCGTCAGTGGGCTGATCATTAACTATCCGCTGGATGACCAGGATGCCATTGCTGTGGAAGCTGCCTGCACTAATGTTCCGGCGTTATTTCTTGATGTCTCTGACCAGACACCCATCAACAGTATTATTTTCTCCCATGAAGACGGTACGCGACTGGGCGTGGAGCATCTGGTCGCATTGGGTCACCAGCAAATCGCGCTGTTAGCGGGCCCATTAAGTTCTGTCTCGGCGCGTCTGCGTCTGGCTGGCTGGCATAAATATCTCACTCGCAATCAAATTCAGCCGATAGCGGAACGGGAAGGCGACTGGAGTGCCATGTCCGGTTTTCAACAAACCATGCAAATGCTGAATGAGGGCATCGTTCCCACTGCGATGCTGGTTGCCAACGATCAGATGGCGCTGGGCGCAATGCGCGCCATTACCGAGTCCGGGCTGCGCGTTGGTGCGGATATCTCGGTAGTGGGATACGACGATACCGAAGACAGCTCATGTTATATCCCGCCGTTAACCACCATCAAACAGGATTTTCGCCTGCTGGGGCAAACCAGCGTGGACCGCTTGCTGCAACTCTCTCAGGGCCAGGCGGTGAAGGGCAATCAGCTGTTGCCCGTCTCACTGGTGAAAAGAAAAACCACCCTGGCGCCCAATACGCAAACCGCCTCTCCCCGCGCGTTGGCCGATTCATTAATGCAGCTGGCACGACAGGTTTCCCGACTGGAAAGCGGGCAGtgagctttcctcggtaccaaattccagaaaagaggcctcccgaaaggggggccttttttcgttttggtcctacttgtgcctgttctatttccgaaccgaccgcttgtatgaatccatcaaaattcgttttctctatgttggattccttgtttgatctggaagaagcaatgaaagctgctgttaagtctccgaatcaggtattgttcctgacaggtgtattcccatccggtaaacgcggatactttgcagttgatctgactcaggaataaattataaattaaggtaagaagattgtaggataagctaatgaaatagaaaaaggatgccgtcacacaacttgtcggcattcttttttgttttattagttgaaaatatagtgaaaaagttgcctaaatatgtatgttaacaaattatttgtcgtaactttgcactccaaatctgtttttaacatatggcactagtgAAACCAGTAACGTTATACGATGTCGCAGAGTATGCCGGTGTCTCTTATCAGACCGTTTCCCGCGTGGTGAACCAGGCCAGCCACGTTTCTGCGAAAACGCGGGAAAAAGTGGAAGCGGCGATGGCGGAGCTCAATTACATTCCCAACCGCGTGGCACAACAACTGGCGGGCAAAGCGTCGCATACCATTGGCATGTTGATCACTGCCAGTACCAATCCTTTCTATTCAGAACTGGTGCGTGGCGTTGAACGCAGCTGCTTCGAACGCGGTTATAGTCTCGTCCTTTGCAATACCGAAGGCGATGAACAGCGGATGAATCGCAATCTGGAAACGCTGATGCAAAAACGCGTTGATGGCTTGCTGTTACTGTGCACCGAAACGCATCAACCTTCGCGTGAAATCATGCAACGTTATCCGACAGTGCCTACTGTGATGATGGACTGGGCTCCGTTCGATGGCGACAGCGATCTTATTCAGGATAACTCGTTGCTGGGCGGAGACTTAGCAACGCAATATCTGATCGATAAAGGTCATACCCGTATCGCCTGTATTACCGGCCCGCTGGATAAAACTCCGGCGCGCCTGCGGTTGGAAGGTTATCGGGCGGCGATGAAACGTGCGGGTCTCAACATTCCTGATGGCTATGAAGTCACTGGTGATTTTGAATTTAACGGCGGGTTTGACGCTATGCGCCAACTGCTATCACATCCGCTGCGTCCTCAGGCCGTCTTTACCGGAAATGACGCTATGGCTGTTGGCGTTTACCAGGCGTTATATCAGGCAGAGTTACAGGTTCCGCAGGATATCGCGGTGATTGGCTATGACGATATCGAACTGGCAAGCTTTATGACGCCACCATTAACCACTATCCACCAACCGAAAGATGAACTGGGGGAGCTGGCGATTGATGTACTCATCCATCGGATAACCCAGCCGACCCTTCAGCAACAACGATTACAACTTACTCCGATTCTGATGGAACGCGGTTCGGCTTAGCTGGTGAAAAGAAAAACCACCCTGGCGCCCAATACGCAAACCGCCTCTCCCCGCGCGTTGGCCGATTCATTAATGCAGCTGGCACGACAGGTTTCCCGACTGGAAAGCGGGCAGTGAgctttcctcggtaccaaattccagaaaagaggcctcccgaaaggggggccttttttcgttttggtccgctcatattgtgatgataatttctacaaatatagtcattggtaactatctatgaaactgtttgatacttttatagttgattaaacttgttcatggcatttgccttaatatcatccgctatgtcaatgtagggtttcatagctttgtagtcgctgtgtcccgtccatttcatgaccacctgtgccgggattccgagagccagcgcattgcagatgaatgtccttcttcctgcatgggtactgagcaaagcgtatttgggtgtgacttcatcaatacgttcatttcccttgtagtaggtttcccgtacaggctcgttgatttctgccagttcgcccagctctttcaggtaatcgttcatcttctggttgctgatgacgggcagagccatgtaattctcgaaatggatgtccttgtatttgtccagtatggctttgctgtatttgttcagttcaatcgtcaggctgtcggcagtcttgactgtggttatttcgatgtggtcggacttcacatcgcttcttttcagattgcgaacatccgaataccgcaaactcgtaaagcagcagaacaggaaaacatcacgcacacgttccaggtattgcttatccttgggtatctggtagtctttcagcttgttcagttcatcccaagtcaggaagattacttttttcgaggtggttttcagtttcggtttgaacgtatcgtatgcaatgttctgatgatgtcctttcttgaagctccagcgcaggaaccatttgaggaatcccatttgcttgccgatggtgctgtttctcatatccttggtgtcacgcaggaagttgacgtattcgttcaatccaaactcgttgaaatagttgaacgttgcatcctccttgaactctttgaggtggttcctcactgctgcaaatttttcataggtggatgccgtccagttattctggttaccgcactcttttacaaactcatcgaacacctcccaaaagctgacaggggcttcttccggctgttcttcgctggtgtctttcattctcatgttgaaagcttccttcaactgttgggtcgttggcatgacctcctgcacctcaaattccttgaaaatattctggatttcggcatagtatttcagcaagtccgtattgatttcggctgcactttgctttagcttgttggtacatccgctctttacccgctgcttatctgcatcccatttggctacgtcaatccggtagcccgttgtaaactcgatgcgttggctggcaaagatgacacgcatacggatgggtacgttctctacgattggcacaccgttctttttccggctctccaatgcaaaaatgatgttgcgcttgatattcataattgggtgcgtttgaaattctacacccaaatatacacccaattattgagatagcaaaagacatttagaaacatttacttttactctatattgtaatttacacttgattatcagtcgtttgcagtcttatgatattctgtgaaagtataagttcgagagcctgtctctccgcaaaaaacgctgaaaatcagcagattgcaaaacaaacaccctgttttacacccaagaatgtaaagtcggctgtttttgttttatttaagataatacaaccactacataataaaagagtagcgatattaaaagaatccgatgagaaaagactaatatttatctatccattcagtttgatttttcaggactttacatcgtcctgaaagtatttgttggtaccggtaccgaggacgcgtaaacatttacagttgcatgtggcctattgtttttagccgttaaatattttataactattaaatagcgatacaaattgttcgaaactaatattgtttatatcatatattctcgcatgttttaaagctttattaaattgattttttgtaaacagtttttcgtactctttgttaacccatttcattacaaaagtttcatatttttttctctctttaaatgccatttttgctggctttctttttaatacaattaatgtgctatccactttaggttttggatggaaataatacctaggaatttttgctaatatagaaatatctacctctgccattaacagcaatgctagtgatctgtttgtatctaataacattttagcaaaaccatattccactattaaataacttattgtggctgaactttcaaaaacaatttttcgaattatatttgtgcttatgttgtaaggtatgctgccaaatattttatatggattgtggctaggaaatgtaaatttcagtatatcatcatttactatttgatagttaggataatttaagagcttattacgagttacctcacataatttagaatcaatttctatcgccgttacaaaattacatctctttaccaatccagcagtaaaatgacctttccctgcacctatttcaaagatgttatctttttcatctaaacttatgcaattcattattttttctatgtgatattttgaagtaataaaattttgactatcttttatatttactttgttcattataacctctccttaatttattgcatctcttttcgaatatttatgttttttgagaaaagaacgtactcatggttcatcccgatatgcgtatcggtctgtatatcagcaactttctatgtgtttcaactacaatagtcatctattctcatctttctgagtccaccccctgcaaagcccctctttacgacataaaaattcggtcggaaaaggtatgcaaaagatgtttctctctttaagagaaactcttcgggatgcaaaaatatgaaaataactccaattcaccaaattatatagcgacttttttacaaaatgctaaaatttgttgatttccgtcaagcaattgttgagcaaaaatgtcttttacgataaaatgatacctcaatatcaactgtttagcaaaacgatatttctcttaaagagagaaacacctttttgttcaccaatccccgacttttaatcccgcggccatgattgaaaaaggaagagtatgagtattcaacatttccgtgtcgcccttattcccttttttgcggcattttgccttcctgtttttgctcacccagaaacgctggtgaaagtaaaagatgctgaagatcagttgggtgcacgagtgggttacatcgaactggatctcaacagcggtaagatccttgagagttttcgccccgaagaacgttttccaatgatgagcacttttaaagttctgctatgtggcgcggtattatcccgtattgacgccgggcaagagcaactcggtcgccgcatacactattctcagaatgacttggttgagtactcaccagtcacagaaaagcatcttacggatggcatgacagtaagagaattatgcagtgctgccataaccatgagtgataacactgcggccaacttacttctgacaacgatcggaggaccgaaggagctaaccgcttttttgcacaacatgggggatcatgtaactcgccttgatcgttgggaaccggagctgaatgaagccataccaaacgacgagcgtgacaccacgatgcctgtagcaatggcaacaacgttgcgcaaactattaactggcgaactacttactctagcttcccggcaacaattaatagactggatggaggcggataaagttgcaggaccacttctgcgctcggcccttccggctggctggtttattgctgataaatctggagccggtgagcgtgggtctcgcggtatcattgcagcactggggccagatggtaagccctcccgtatcgtagttatctacacgacggggagtcaggcaactatggatgaacgaaatagacagatcgctgagataggtgcctcactgattaagcattggtaactgtcagaccaagtttactcataacgcgtcaattcgagggggatcaattccgtgataggtgggctgcccttcctggttggcttggtttcatcagccatccgcttgccctcatctgttacgccggcggtagccggccagcctcgcagagcaggattcccgttgagcaccgccaggtgcgaataagggacagtgaagaaggaacacccgctcgcgggtgggcctacttcacctatcctgcccggctgacgccgttggatacaccaaggaaagtctacacgaaccctttggcaaaatcctgtatatcgtgcgaaaaaggatggatataccgaaaaaatcgctataatgaccccgaagcagggttatgcagcggaaaacggaattgatccggccacgatgcgtccggcgtagaggatctgaagatcagcagttcaacctgttgatagtacgtactaagctctcatgtttcacgtactaagctctcatgtttaacgtactaagctctcatgtttaacgaactaaaccctcatggctaacgtactaagctctcatggctaacgtactaagctctcatgtttcacgtactaagctctcatgtttgaacaataaaattaatataaatcagcaacttaaatagcctctaaggttttaagttttataagaaaaaaaagaatatataaggcttttaaagcttttaag

>pBH202

gtttaacggttgtggacaacaagccagggatgtaacgcactgagaagcccttagagcctctcaaagcaattttgagtgacacaggaacacttaacggctgacatgggaattcccctccaccgcggtggtacaaagaaaattcgacaaactgttatttttctatctatttatttgaattTTAAGCGCTTAAaatttacctttgtcggcaattTTAAGCGCTTAAaattaaataaagatattctcgtcaaacaaatataaataatataaacatggtttttactctggaagattttgttggcgattggcgtcagaccgcgggttataatttggatcaagtcctggaacagggtggcgtaagctctctgttccagaacctgggtgtgagcgtgacgccgattcagcgcatcgttctgtccggcgagaacggtctgaaaattgatattcatgtgatcatcccgtacgaaggcctgagcggtgaccaaatgggtcaaatcgagaaaatctttaaagtcgtctacccagttgacgatcaccacttcaaggttatcttgcattacggtacgctggtgattgatggtgtgaccccgaatatgattgactatttcggccgtccgtatgaaggcattgccgtttttgacggtaaaaagatcaccgtcaccggtaccctgtggaatggcaataagattattgacgagcgtctgattaacccggacggcagcctgctgttccgcgtgaccatcaacggtgtcacgggttggcgtctgtgcgagcgcatcctggcataatgaactgcacttgctttgataattaatgataaacaatctaaaagcactctaatcgttatcggagtgcttttagattactaatcaaattgcttctactaattgcctatcttccagtgatggaacagcatttgtgcattggctgcaacaatcagccttgatctggaagaagcaatgaaagctgctgttaagtctccgaatcaggtattgttcctgacaggtgtattcccatccggtaaacgcggatactttgcagttgatctgactcaggaataaattataaattaaggtaagaagattgtaggataagctaatgaaatagaaaaaggatgccgtcacacaacttgtcggcattcttttttgttttattagttgaaaatatagtgaaaaagttgcctaaatatgtatgttaacaaattatttgtcgtaactttgcactccaaatctgtttttaacatatggcactagtgAAACCAGTAACGTTATACGATGTCGCAGAGTATGCCGGTGTCTCTACCGCGACCGTTTCCAACGTGGTGAACCAGGCCAGCCACGTTTCTGCGAAAACGCGGGAAAAAGTGGAAGCGGCGATGGCGGAGCTGAATTACATTCCCAACCGCGTGGCACAACAACTGGCGGGCAAACAGTCGTTGCTGATTGGCGTTGCCACCTCCAGTCTGGCCCTGCACGCGCCGTCGCAAATTGTCGCGGCGATTAAATCTCGCGCCGATCAACTGGGTGCCAGCGTGGTGGTGTCGATGGTAGAACGAAGCGGCGTCGAAGCCTGTAAAACGGCGGTGCACAATCTTCTCGCGCAACGCGTCAGTGGGCTGATCATTAACTATCCGCTGGATGACCAGGATGCCATTGCTGTGGAAGCTGCCTGCACTAATGTTCCGGCGTTATTTCTTGATGTCTCTGACCAGACACCCATCAACAGTATTATTTTCTCCCATGAAGACGGTACGCGACTGGGCGTGGAGCATCTGGTCGCATTGGGTCACCAGCAAATCGCGCTGTTAGCGGGCCCATTAAGTTCTGTCTCGGCGCGTCTGCGTCTGGCTGGCTGGCATAAATATCTCACTCGCAATCAAATTCAGCCGATAGCGGAACGGGAAGGCGACTGGAGTGCCATGTCCGGTTTTCAACAAACCATGCAAATGCTGAATGAGGGCATCGTTCCCACTGCGATGCTGGTTGCCAACGATCAGATGGCGCTGGGCGCAATGCGCGCCATTACCGAGTCCGGGCTGCGCGTTGGTGCGGATATCTCGGTAGTGGGATACGACGATACCGAAGACAGCTCATGTTATATCCCGCCGTTAACCACCATCAAACAGGATTTTCGCCTGCTGGGGCAAACCAGCGTGGACCGCTTGCTGCAACTCTCTCAGGGCCAGGCGGTGAAGGGCAATCAGCTGTTGCCCGTCTCACTGGTGAAAAGAAAAACCACCCTGGCGCCCAATACGCAAACCGCCTCTCCCCGCGCGTTGGCCGATTCATTAATGCAGCTGGCACGACAGGTTTCCCGACTGGAAAGCGGGCAGtgagctttcctcggtaccaaattccagaaaagaggcctcccgaaaggggggccttttttcgttttggtcctacttgtgcctgttctatttccgaaccgaccgcttgtatgaatccatcaaaattcgttttctctatgttggattccttgtttgatctggaagaagcaatgaaagctgctgttaagtctccgaatcaggtattgttcctgacaggtgtattcccatccggtaaacgcggatactttgcagttgatctgactcaggaataaattataaattaaggtaagaagattgtaggataagctaatgaaatagaaaaaggatgccgtcacacaacttgtcggcattcttttttgttttattagttgaaaatatagtgaaaaagttgcctaaatatgtatgttaacaaattatttgtcgtaactttgcactccaaatctgtttttaacatatggcactagtgAAACCAGTAACGTTATACGATGTCGCAGAGTATGCCGGTGTCTCTACCGCGACCGTTTCCAACGTGGTGAACCAGGCCAGCCACGTTTCTGCGAAAACGCGGGAAAAAGTGGAAGCGGCGATGGCGGAGCTCAATTACATTCCCAACCGCGTGGCACAACAACTGGCGGGCAAAGCGTCGCATACCATTGGCATGTTGATCACTGCCAGTACCAATCCTTTCTATTCAGAACTGGTGCGTGGCGTTGAACGCAGCTGCTTCGAACGCGGTTATAGTCTCGTCCTTTGCAATACCGAAGGCGATGAACAGCGGATGAATCGCAATCTGGAAACGCTGATGCAAAAACGCGTTGATGGCTTGCTGTTACTGTGCACCGAAACGCATCAACCTTCGCGTGAAATCATGCAACGTTATCCGACAGTGCCTACTGTGATGATGGACTGGGCTCCGTTCGATGGCGACAGCGATCTTATTCAGGATAACTCGTTGCTGGGCGGAGACTTAGCAACGCAATATCTGATCGATAAAGGTCATACCCGTATCGCCTGTATTACCGGCCCGCTGGATAAAACTCCGGCGCGCCTGCGGTTGGAAGGTTATCGGGCGGCGATGAAACGTGCGGGTCTCAACATTCCTGATGGCTATGAAGTCACTGGTGATTTTGAATTTAACGGCGGGTTTGACGCTATGCGCCAACTGCTATCACATCCGCTGCGTCCTCAGGCCGTCTTTACCGGAAATGACGCTATGGCTGTTGGCGTTTACCAGGCGTTATATCAGGCAGAGTTACAGGTTCCGCAGGATATCGCGGTGATTGGCTATGACGATATCGAACTGGCAAGCTTTATGACGCCACCATTAACCACTATCCACCAACCGAAAGATGAACTGGGGGAGCTGGCGATTGATGTACTCATCCATCGGATAACCCAGCCGACCCTTCAGCAACAACGATTACAACTTACTCCGATTCTGATGGAACGCGGTTCGGCTTAGCTGGTGAAAAGAAAAACCACCCTGGCGCCCAATACGCAAACCGCCTCTCCCCGCGCGTTGGCCGATTCATTAATGCAGCTGGCACGACAGGTTTCCCGACTGGAAAGCGGGCAGTGAgctttcctcggtaccaaattccagaaaagaggcctcccgaaaggggggccttttttcgttttggtccgctcatattgtgatgataatttctacaaatatagtcattggtaactatctatgaaactgtttgatacttttatagttgattaaacttgttcatggcatttgccttaatatcatccgctatgtcaatgtagggtttcatagctttgtagtcgctgtgtcccgtccatttcatgaccacctgtgccgggattccgagagccagcgcattgcagatgaatgtccttcttcctgcatgggtactgagcaaagcgtatttgggtgtgacttcatcaatacgttcatttcccttgtagtaggtttcccgtacaggctcgttgatttctgccagttcgcccagctctttcaggtaatcgttcatcttctggttgctgatgacgggcagagccatgtaattctcgaaatggatgtccttgtatttgtccagtatggctttgctgtatttgttcagttcaatcgtcaggctgtcggcagtcttgactgtggttatttcgatgtggtcggacttcacatcgcttcttttcagattgcgaacatccgaataccgcaaactcgtaaagcagcagaacaggaaaacatcacgcacacgttccaggtattgcttatccttgggtatctggtagtctttcagcttgttcagttcatcccaagtcaggaagattacttttttcgaggtggttttcagtttcggtttgaacgtatcgtatgcaatgttctgatgatgtcctttcttgaagctccagcgcaggaaccatttgaggaatcccatttgcttgccgatggtgctgtttctcatatccttggtgtcacgcaggaagttgacgtattcgttcaatccaaactcgttgaaatagttgaacgttgcatcctccttgaactctttgaggtggttcctcactgctgcaaatttttcataggtggatgccgtccagttattctggttaccgcactcttttacaaactcatcgaacacctcccaaaagctgacaggggcttcttccggctgttcttcgctggtgtctttcattctcatgttgaaagcttccttcaactgttgggtcgttggcatgacctcctgcacctcaaattccttgaaaatattctggatttcggcatagtatttcagcaagtccgtattgatttcggctgcactttgctttagcttgttggtacatccgctctttacccgctgcttatctgcatcccatttggctacgtcaatccggtagcccgttgtaaactcgatgcgttggctggcaaagatgacacgcatacggatgggtacgttctctacgattggcacaccgttctttttccggctctccaatgcaaaaatgatgttgcgcttgatattcataattgggtgcgtttgaaattctacacccaaatatacacccaattattgagatagcaaaagacatttagaaacatttacttttactctatattgtaatttacacttgattatcagtcgtttgcagtcttatgatattctgtgaaagtataagttcgagagcctgtctctccgcaaaaaacgctgaaaatcagcagattgcaaaacaaacaccctgttttacacccaagaatgtaaagtcggctgtttttgttttatttaagataatacaaccactacataataaaagagtagcgatattaaaagaatccgatgagaaaagactaatatttatctatccattcagtttgatttttcaggactttacatcgtcctgaaagtatttgttggtaccggtaccgaggacgcgtaaacatttacagttgcatgtggcctattgtttttagccgttaaatattttataactattaaatagcgatacaaattgttcgaaactaatattgtttatatcatatattctcgcatgttttaaagctttattaaattgattttttgtaaacagtttttcgtactctttgttaacccatttcattacaaaagtttcatatttttttctctctttaaatgccatttttgctggctttctttttaatacaattaatgtgctatccactttaggttttggatggaaataatacctaggaatttttgctaatatagaaatatctacctctgccattaacagcaatgctagtgatctgtttgtatctaataacattttagcaaaaccatattccactattaaataacttattgtggctgaactttcaaaaacaatttttcgaattatatttgtgcttatgttgtaaggtatgctgccaaatattttatatggattgtggctaggaaatgtaaatttcagtatatcatcatttactatttgatagttaggataatttaagagcttattacgagttacctcacataatttagaatcaatttctatcgccgttacaaaattacatctctttaccaatccagcagtaaaatgacctttccctgcacctatttcaaagatgttatctttttcatctaaacttatgcaattcattattttttctatgtgatattttgaagtaataaaattttgactatcttttatatttactttgttcattataacctctccttaatttattgcatctcttttcgaatatttatgttttttgagaaaagaacgtactcatggttcatcccgatatgcgtatcggtctgtatatcagcaactttctatgtgtttcaactacaatagtcatctattctcatctttctgagtccaccccctgcaaagcccctctttacgacataaaaattcggtcggaaaaggtatgcaaaagatgtttctctctttaagagaaactcttcgggatgcaaaaatatgaaaataactccaattcaccaaattatatagcgacttttttacaaaatgctaaaatttgttgatttccgtcaagcaattgttgagcaaaaatgtcttttacgataaaatgatacctcaatatcaactgtttagcaaaacgatatttctcttaaagagagaaacacctttttgttcaccaatccccgacttttaatcccgcggccatgattgaaaaaggaagagtatgagtattcaacatttccgtgtcgcccttattcccttttttgcggcattttgccttcctgtttttgctcacccagaaacgctggtgaaagtaaaagatgctgaagatcagttgggtgcacgagtgggttacatcgaactggatctcaacagcggtaagatccttgagagttttcgccccgaagaacgttttccaatgatgagcacttttaaagttctgctatgtggcgcggtattatcccgtattgacgccgggcaagagcaactcggtcgccgcatacactattctcagaatgacttggttgagtactcaccagtcacagaaaagcatcttacggatggcatgacagtaagagaattatgcagtgctgccataaccatgagtgataacactgcggccaacttacttctgacaacgatcggaggaccgaaggagctaaccgcttttttgcacaacatgggggatcatgtaactcgccttgatcgttgggaaccggagctgaatgaagccataccaaacgacgagcgtgacaccacgatgcctgtagcaatggcaacaacgttgcgcaaactattaactggcgaactacttactctagcttcccggcaacaattaatagactggatggaggcggataaagttgcaggaccacttctgcgctcggcccttccggctggctggtttattgctgataaatctggagccggtgagcgtgggtctcgcggtatcattgcagcactggggccagatggtaagccctcccgtatcgtagttatctacacgacggggagtcaggcaactatggatgaacgaaatagacagatcgctgagataggtgcctcactgattaagcattggtaactgtcagaccaagtttactcataacgcgtcaattcgagggggatcaattccgtgataggtgggctgcccttcctggttggcttggtttcatcagccatccgcttgccctcatctgttacgccggcggtagccggccagcctcgcagagcaggattcccgttgagcaccgccaggtgcgaataagggacagtgaagaaggaacacccgctcgcgggtgggcctacttcacctatcctgcccggctgacgccgttggatacaccaaggaaagtctacacgaaccctttggcaaaatcctgtatatcgtgcgaaaaaggatggatataccgaaaaaatcgctataatgaccccgaagcagggttatgcagcggaaaacggaattgatccggccacgatgcgtccggcgtagaggatctgaagatcagcagttcaacctgttgatagtacgtactaagctctcatgtttcacgtactaagctctcatgtttaacgtactaagctctcatgtttaacgaactaaaccctcatggctaacgtactaagctctcatggctaacgtactaagctctcatgtttcacgtactaagctctcatgtttgaacaataaaattaatataaatcagcaacttaaatagcctctaaggttttaagttttataagaaaaaaaagaatatataaggcttttaaagcttttaag

>pBH203

gtttaacggttgtggacaacaagccagggatgtaacgcactgagaagcccttagagcctctcaaagcaattttgagtgacacaggaacacttaacggctgacatgggaattcccctccaccgcggtggtacaaagaaaattcgacaaactgttatttttctatctatttatttgAATTaggAGCGCTcctAATTtacctttgtcggcAATTaggAGCGCTcctAATTaaataaagatattctcgtcaaacaaatataaataatataaacatggtttttactctggaagattttgttggcgattggcgtcagaccgcgggttataatttggatcaagtcctggaacagggtggcgtaagctctctgttccagaacctgggtgtgagcgtgacgccgattcagcgcatcgttctgtccggcgagaacggtctgaaaattgatattcatgtgatcatcccgtacgaaggcctgagcggtgaccaaatgggtcaaatcgagaaaatctttaaagtcgtctacccagttgacgatcaccacttcaaggttatcttgcattacggtacgctggtgattgatggtgtgaccccgaatatgattgactatttcggccgtccgtatgaaggcattgccgtttttgacggtaaaaagatcaccgtcaccggtaccctgtggaatggcaataagattattgacgagcgtctgattaacccggacggcagcctgctgttccgcgtgaccatcaacggtgtcacgggttggcgtctgtgcgagcgcatcctggcataatgaactgcacttgctttgataattaatgataaacaatctaaaagcactctaatcgttatcggagtgcttttagattactaatcaaattgcttctactaattgcctatcttccagtgatggaacagcatttgtgcattggctgcaacaatcagccttgatctggaagaagcaatgaaagctgctgttaagtctccgaatcaggtattgttcctgacaggtgtattcccatccggtaaacgcggatactttgcagttgatctgactcaggaataaattataaattaaggtaagaagattgtaggataagctaatgaaatagaaaaaggatgccgtcacacaacttgtcggcattcttttttgttttattagttgaaaatatagtgaaaaagttgcctaaatatgtatgttaacaaattatttgtcgtaactttgcactccaaatctgtttttaacatatggcactagtgAAACCAGTAACGTTATACGATGTCGCAGAGTATGCCGGTGTCTCTAAAAGCACCGTTTCCCTGGTGGTGAACCAGGCCAGCCACGTTTCTGCGAAAACGCGGGAAAAAGTGGAAGCGGCGATGGCGGAGCTGAATTACATTCCCAACCGCGTGGCACAACAACTGGCGGGCAAACAGTCGTTGCTGATTGGCGTTGCCACCTCCAGTCTGGCCCTGCACGCGCCGTCGCAAATTGTCGCGGCGATTAAATCTCGCGCCGATCAACTGGGTGCCAGCGTGGTGGTGTCGATGGTAGAACGAAGCGGCGTCGAAGCCTGTAAAACGGCGGTGCACAATCTTCTCGCGCAACGCGTCAGTGGGCTGATCATTAACTATCCGCTGGATGACCAGGATGCCATTGCTGTGGAAGCTGCCTGCACTAATGTTCCGGCGTTATTTCTTGATGTCTCTGACCAGACACCCATCAACAGTATTATTTTCTCCCATGAAGACGGTACGCGACTGGGCGTGGAGCATCTGGTCGCATTGGGTCACCAGCAAATCGCGCTGTTAGCGGGCCCATTAAGTTCTGTCTCGGCGCGTCTGCGTCTGGCTGGCTGGCATAAATATCTCACTCGCAATCAAATTCAGCCGATAGCGGAACGGGAAGGCGACTGGAGTGCCATGTCCGGTTTTCAACAAACCATGCAAATGCTGAATGAGGGCATCGTTCCCACTGCGATGCTGGTTGCCAACGATCAGATGGCGCTGGGCGCAATGCGCGCCATTACCGAGTCCGGGCTGCGCGTTGGTGCGGATATCTCGGTAGTGGGATACGACGATACCGAAGACAGCTCATGTTATATCCCGCCGTTAACCACCATCAAACAGGATTTTCGCCTGCTGGGGCAAACCAGCGTGGACCGCTTGCTGCAACTCTCTCAGGGCCAGGCGGTGAAGGGCAATCAGCTGTTGCCCGTCTCACTGGTGAAAAGAAAAACCACCCTGGCGCCCAATACGCAAACCGCCTCTCCCCGCGCGTTGGCCGATTCATTAATGCAGCTGGCACGACAGGTTTCCCGACTGGAAAGCGGGCAGtgagctttcctcggtaccaaattccagaaaagaggcctcccgaaaggggggccttttttcgttttggtcctacttgtgcctgttctatttccgaaccgaccgcttgtatgaatccatcaaaattcgttttctctatgttggattccttgtttgatctggaagaagcaatgaaagctgctgttaagtctccgaatcaggtattgttcctgacaggtgtattcccatccggtaaacgcggatactttgcagttgatctgactcaggaataaattataaattaaggtaagaagattgtaggataagctaatgaaatagaaaaaggatgccgtcacacaacttgtcggcattcttttttgttttattagttgaaaatatagtgaaaaagttgcctaaatatgtatgttaacaaattatttgtcgtaactttgcactccaaatctgtttttaacatatggcactagtgAAACCAGTAACGTTATACGATGTCGCAGAGTATGCCGGTGTCTCTAAAAGCACCGTTTCCCTGGTGGTGAACCAGGCCAGCCACGTTTCTGCGAAAACGCGGGAAAAAGTGGAAGCGGCGATGGCGGAGCTCAATTACATTCCCAACCGCGTGGCACAACAACTGGCGGGCAAAGCGTCGCATACCATTGGCATGTTGATCACTGCCAGTACCAATCCTTTCTATTCAGAACTGGTGCGTGGCGTTGAACGCAGCTGCTTCGAACGCGGTTATAGTCTCGTCCTTTGCAATACCGAAGGCGATGAACAGCGGATGAATCGCAATCTGGAAACGCTGATGCAAAAACGCGTTGATGGCTTGCTGTTACTGTGCACCGAAACGCATCAACCTTCGCGTGAAATCATGCAACGTTATCCGACAGTGCCTACTGTGATGATGGACTGGGCTCCGTTCGATGGCGACAGCGATCTTATTCAGGATAACTCGTTGCTGGGCGGAGACTTAGCAACGCAATATCTGATCGATAAAGGTCATACCCGTATCGCCTGTATTACCGGCCCGCTGGATAAAACTCCGGCGCGCCTGCGGTTGGAAGGTTATCGGGCGGCGATGAAACGTGCGGGTCTCAACATTCCTGATGGCTATGAAGTCACTGGTGATTTTGAATTTAACGGCGGGTTTGACGCTATGCGCCAACTGCTATCACATCCGCTGCGTCCTCAGGCCGTCTTTACCGGAAATGACGCTATGGCTGTTGGCGTTTACCAGGCGTTATATCAGGCAGAGTTACAGGTTCCGCAGGATATCGCGGTGATTGGCTATGACGATATCGAACTGGCAAGCTTTATGACGCCACCATTAACCACTATCCACCAACCGAAAGATGAACTGGGGGAGCTGGCGATTGATGTACTCATCCATCGGATAACCCAGCCGACCCTTCAGCAACAACGATTACAACTTACTCCGATTCTGATGGAACGCGGTTCGGCTTAGCTGGTGAAAAGAAAAACCACCCTGGCGCCCAATACGCAAACCGCCTCTCCCCGCGCGTTGGCCGATTCATTAATGCAGCTGGCACGACAGGTTTCCCGACTGGAAAGCGGGCAGTGAgctttcctcggtaccaaattccagaaaagaggcctcccgaaaggggggccttttttcgttttggtccgctcatattgtgatgataatttctacaaatatagtcattggtaactatctatgaaactgtttgatacttttatagttgattaaacttgttcatggcatttgccttaatatcatccgctatgtcaatgtagggtttcatagctttgtagtcgctgtgtcccgtccatttcatgaccacctgtgccgggattccgagagccagcgcattgcagatgaatgtccttcttcctgcatgggtactgagcaaagcgtatttgggtgtgacttcatcaatacgttcatttcccttgtagtaggtttcccgtacaggctcgttgatttctgccagttcgcccagctctttcaggtaatcgttcatcttctggttgctgatgacgggcagagccatgtaattctcgaaatggatgtccttgtatttgtccagtatggctttgctgtatttgttcagttcaatcgtcaggctgtcggcagtcttgactgtggttatttcgatgtggtcggacttcacatcgcttcttttcagattgcgaacatccgaataccgcaaactcgtaaagcagcagaacaggaaaacatcacgcacacgttccaggtattgcttatccttgggtatctggtagtctttcagcttgttcagttcatcccaagtcaggaagattacttttttcgaggtggttttcagtttcggtttgaacgtatcgtatgcaatgttctgatgatgtcctttcttgaagctccagcgcaggaaccatttgaggaatcccatttgcttgccgatggtgctgtttctcatatccttggtgtcacgcaggaagttgacgtattcgttcaatccaaactcgttgaaatagttgaacgttgcatcctccttgaactctttgaggtggttcctcactgctgcaaatttttcataggtggatgccgtccagttattctggttaccgcactcttttacaaactcatcgaacacctcccaaaagctgacaggggcttcttccggctgttcttcgctggtgtctttcattctcatgttgaaagcttccttcaactgttgggtcgttggcatgacctcctgcacctcaaattccttgaaaatattctggatttcggcatagtatttcagcaagtccgtattgatttcggctgcactttgctttagcttgttggtacatccgctctttacccgctgcttatctgcatcccatttggctacgtcaatccggtagcccgttgtaaactcgatgcgttggctggcaaagatgacacgcatacggatgggtacgttctctacgattggcacaccgttctttttccggctctccaatgcaaaaatgatgttgcgcttgatattcataattgggtgcgtttgaaattctacacccaaatatacacccaattattgagatagcaaaagacatttagaaacatttacttttactctatattgtaatttacacttgattatcagtcgtttgcagtcttatgatattctgtgaaagtataagttcgagagcctgtctctccgcaaaaaacgctgaaaatcagcagattgcaaaacaaacaccctgttttacacccaagaatgtaaagtcggctgtttttgttttatttaagataatacaaccactacataataaaagagtagcgatattaaaagaatccgatgagaaaagactaatatttatctatccattcagtttgatttttcaggactttacatcgtcctgaaagtatttgttggtaccggtaccgaggacgcgtaaacatttacagttgcatgtggcctattgtttttagccgttaaatattttataactattaaatagcgatacaaattgttcgaaactaatattgtttatatcatatattctcgcatgttttaaagctttattaaattgattttttgtaaacagtttttcgtactctttgttaacccatttcattacaaaagtttcatatttttttctctctttaaatgccatttttgctggctttctttttaatacaattaatgtgctatccactttaggttttggatggaaataatacctaggaatttttgctaatatagaaatatctacctctgccattaacagcaatgctagtgatctgtttgtatctaataacattttagcaaaaccatattccactattaaataacttattgtggctgaactttcaaaaacaatttttcgaattatatttgtgcttatgttgtaaggtatgctgccaaatattttatatggattgtggctaggaaatgtaaatttcagtatatcatcatttactatttgatagttaggataatttaagagcttattacgagttacctcacataatttagaatcaatttctatcgccgttacaaaattacatctctttaccaatccagcagtaaaatgacctttccctgcacctatttcaaagatgttatctttttcatctaaacttatgcaattcattattttttctatgtgatattttgaagtaataaaattttgactatcttttatatttactttgttcattataacctctccttaatttattgcatctcttttcgaatatttatgttttttgagaaaagaacgtactcatggttcatcccgatatgcgtatcggtctgtatatcagcaactttctatgtgtttcaactacaatagtcatctattctcatctttctgagtccaccccctgcaaagcccctctttacgacataaaaattcggtcggaaaaggtatgcaaaagatgtttctctctttaagagaaactcttcgggatgcaaaaatatgaaaataactccaattcaccaaattatatagcgacttttttacaaaatgctaaaatttgttgatttccgtcaagcaattgttgagcaaaaatgtcttttacgataaaatgatacctcaatatcaactgtttagcaaaacgatatttctcttaaagagagaaacacctttttgttcaccaatccccgacttttaatcccgcggccatgattgaaaaaggaagagtatgagtattcaacatttccgtgtcgcccttattcccttttttgcggcattttgccttcctgtttttgctcacccagaaacgctggtgaaagtaaaagatgctgaagatcagttgggtgcacgagtgggttacatcgaactggatctcaacagcggtaagatccttgagagttttcgccccgaagaacgttttccaatgatgagcacttttaaagttctgctatgtggcgcggtattatcccgtattgacgccgggcaagagcaactcggtcgccgcatacactattctcagaatgacttggttgagtactcaccagtcacagaaaagcatcttacggatggcatgacagtaagagaattatgcagtgctgccataaccatgagtgataacactgcggccaacttacttctgacaacgatcggaggaccgaaggagctaaccgcttttttgcacaacatgggggatcatgtaactcgccttgatcgttgggaaccggagctgaatgaagccataccaaacgacgagcgtgacaccacgatgcctgtagcaatggcaacaacgttgcgcaaactattaactggcgaactacttactctagcttcccggcaacaattaatagactggatggaggcggataaagttgcaggaccacttctgcgctcggcccttccggctggctggtttattgctgataaatctggagccggtgagcgtgggtctcgcggtatcattgcagcactggggccagatggtaagccctcccgtatcgtagttatctacacgacggggagtcaggcaactatggatgaacgaaatagacagatcgctgagataggtgcctcactgattaagcattggtaactgtcagaccaagtttactcataacgcgtcaattcgagggggatcaattccgtgataggtgggctgcccttcctggttggcttggtttcatcagccatccgcttgccctcatctgttacgccggcggtagccggccagcctcgcagagcaggattcccgttgagcaccgccaggtgcgaataagggacagtgaagaaggaacacccgctcgcgggtgggcctacttcacctatcctgcccggctgacgccgttggatacaccaaggaaagtctacacgaaccctttggcaaaatcctgtatatcgtgcgaaaaaggatggatataccgaaaaaatcgctataatgaccccgaagcagggttatgcagcggaaaacggaattgatccggccacgatgcgtccggcgtagaggatctgaagatcagcagttcaacctgttgatagtacgtactaagctctcatgtttcacgtactaagctctcatgtttaacgtactaagctctcatgtttaacgaactaaaccctcatggctaacgtactaagctctcatggctaacgtactaagctctcatgtttcacgtactaagctctcatgtttgaacaataaaattaatataaatcagcaacttaaatagcctctaaggttttaagttttataagaaaaaaaagaatatataaggcttttaaagcttttaag

>pBH204

gtttaacggttgtggacaacaagccagggatgtaacgcactgagaagcccttagagcctctcaaagcaattttgagtgacacaggaacacttaacggctgacatgggaattcccctccaccgcggtggtacaaagaaaattcgacaaactgttatttttctatctatttatttgAATTGTGAGCGGATAACAATTacctttgtcggcAATTGTGAGCGGATAACAATTaaataaagatattctcgtcaaacaaatataaataatataaacatggtttttactctggaagattttgttggcgattggcgtcagaccgcgggttataatttggatcaagtcctggaacagggtggcgtaagctctctgttccagaacctgggtgtgagcgtgacgccgattcagcgcatcgttctgtccggcgagaacggtctgaaaattgatattcatgtgatcatcccgtacgaaggcctgagcggtgaccaaatgggtcaaatcgagaaaatctttaaagtcgtctacccagttgacgatcaccacttcaaggttatcttgcattacggtacgctggtgattgatggtgtgaccccgaatatgattgactatttcggccgtccgtatgaaggcattgccgtttttgacggtaaaaagatcaccgtcaccggtaccctgtggaatggcaataagattattgacgagcgtctgattaacccggacggcagcctgctgttccgcgtgaccatcaacggtgtcacgggttggcgtctgtgcgagcgcatcctggcataatgaactgcacttgctttgataattaatgataaacaatctaaaagcactctaatcgttatcggagtgcttttagattactaatcaaattgcttctactaattgcctatcttccagtgatggaacagcatttgtgcattggctgcaacaatcagccttgatctggaagaagcaatgaaagctgctgttaagtctccgaatcaggtattgttcctgacaggtgtattcccatccggtaaacgcggatactttgcagttgatctgactcaggaataaattataaattaaggtaagaagattgtaggataagctaatgaaatagaaaaaggatgccgtcacacaacttgtcggcattcttttttgttttattagttgaaaatatagtgaaaaagttgcctaaatatgtatgttaacaaattatttgtcgtaactttgcactccaaatctgtttttaacatatggcactagtgAAACCAGTAACGTTATACGATGTCGCAGAGTATGCCGGTGTCTCTTATCAGACCGTTTCCCGCGTGGTGAACCAGGCCAGCCACGTTTCTGCGAAAACGCGGGAAAAAGTGGAAGCGGCGATGGCGGAGCTGAATTACATTCCCAACCGCGTGGCACAACAACTGGCGGGCAAACAGTCGTTGCTGATTGGCGTTGCCACCTCCAGTCTGGCCCTGCACGCGCTGTCGCAAATTGTCGCGGCGATTAAATCTCGCGCCTATCAACTGGGTGCCAGCGTGTTCGTGTCGATGGTAGAACGAAGCGGCATCGAAGCCTGTAAAACGGCGGTGCACAATCTTCTCGCGCAACGCGTCAGTGGGCTGATCATTAACTATCCGCTGGATAACCAGGATGCCATTGCTGTGGAAGCTGCCTGCACTAATGTTCCGGCGTTATTTCTTGATGTCTCTGACCAGACACCCATCAACAGTATTATTTTCTCCCATGAAGACGGTACGCGACTGGGCGTGGAGCATCTGGTCGCATTGGGTCACCAGCAAATCGCGCTGTTAGCGGGCCCATTAAGTTCTGTCTCGGCGCGTCTGCGTCTGGCTGGCTGGCATAAATATCTCACTCGCAATCAAATTCAGCCGATAGCGGAACGGGAAGGCGACTGGAGTGCCATGTCCGGTTTTCAACAAACCATGCAAATGCTGAATGAGGGCATCGTTCCCACTGCGATGCTGGTTGCCAACGATCAGATGGCGCTGGGCGCAATGCGCGCCATTACCGAGACCGGGCTGCGCGTTGGTGCGGATATCTCGGTAGTGGGATACGACGATACCGAAGACAGCTCATGTTATATCCCGCCGTTAACCACCATCAAACAGGATTTTCGCCTGCTGGGGCAAACCAGCGTGGACCGCTTGCTGCAACTCTCTCAGGGCCAGGCGGTGAAGGGCAATCAGCTGTTGCCCGTCTCACTGGTGAAAAGAAAAACCACCCTGGCGCCCAATACGCAAACCGCCTCTCCCCGCGCGTTGGCCGATTCATTAATGCAGCTGGCACGACAGGTTTCCCGACTGGAAAGCGGGCAGtgagctttcctcggtaccaaattccagaaaagaggcctcccgaaaggggggccttttttcgttttggtcctacttgtgcctgttctatttccgaaccgaccgcttgtatgaatccatcaaaattcgttttctctatgttggattccttgtttgatctggaagaagcaatgaaagctgctgttaagtctccgaatcaggtattgttcctgacaggtgtattcccatccggtaaacgcggatactttgcagttgatctgactcaggaataaattataaattaaggtaagaagattgtaggataagctaatgaaatagaaaaaggatgccgtcacacaacttgtcggcattcttttttgttttattagttgaaaatatagtgaaaaagttgcctaaatatgtatgttaacaaattatttgtcgtaactttgcactccaaatctgtttttaacatatggcactagtgAAACCAGTAACGTTATACGATGTCGCAGAGTATGCCGGTGTCTCTTATCAGACCGTTTCCCGCGTGGTGAACCAGGCCAGCCACGTTTCTGCGAAAACGCGGGAAAAAGTGGAAGCGGCGATGGCGGAGCTCAATTACATTCCCAACCGCGTGGCACAACAACTGGCGGGCAAAGCGTCGCATACCATTGGCATGTTGATCACTGCCAGTACCAATCCTTTCTATTCAGAACTGGTGCGTGGCGTTGAACGCAGCTGCTTCGAACGCGGTTATAGTCTCGatCTTTGCAATACCGAAGGCGATGAACAGCGGATGAATCGCAATCTGGAAACGCTGATGCAAAAACGCGTTGATGGCTTGCTGTTACTGTGCACCGAAACGCATCAACCTTCGCGTGAAATCATGCAACGTTATCCGACAGTGCCTACTGTGATGATGGACTGGGCTCCGTTCGATGGCGACAGCGATCTTATTCAGGATAACTCGTTGCTGGGCGGAGACTTAGCAACGCAATATCTGATCGATAAAGGTCATACCCGTATCGCCTGTATTACCGGCCCGCTGGATAAAACTCCGGCGCGCCTGCGGTTGGAAGGTTATCGGGCGGCGATGAAACGTGCGGGTCTCAACATTCCTGATGGCTATGAAGTCACTGGTGATTTTGAATTTAACGGCGGGTTTGACGCTATGCGCCAACTGCTATCACATCCGCTGCGTCCTCAGGCCGTCTTTACCGGAAATGACGCTATGGCTGTTGGCGTTTACCAGGCGTTATATCAGGCAGAGTTACAGGTTCCGCAGGATATCGCGGTGATTGGCTATGACGATATCGAACTGGCAAGCTTTATGACGCCACCATTAACCACTATCCACCAACCGAAAGATGAACTGGGGGAGCTGGCGATTGATGTACTCATCCATCGGATAACCCAGCCGACCCTTCAGCAACAACGATTACAACTTACTCCGATTCTGATGGAACGCGGTTCGGCTTAGCTGGTGAAAAGAAAAACCACCCTGGCGCCCAATACGCAAACCGCCTCTCCCCGCGCGTTGGCCGATTCATTAATGCAGCTGGCACGACAGGTTTCCCGACTGGAAAGCGGGCAGTGAgctttcctcggtaccaaattccagaaaagaggcctcccgaaaggggggccttttttcgttttggtccgctcatattgtgatgataatttctacaaatatagtcattggtaactatctatgaaactgtttgatacttttatagttgattaaacttgttcatggcatttgccttaatatcatccgctatgtcaatgtagggtttcatagctttgtagtcgctgtgtcccgtccatttcatgaccacctgtgccgggattccgagagccagcgcattgcagatgaatgtccttcttcctgcatgggtactgagcaaagcgtatttgggtgtgacttcatcaatacgttcatttcccttgtagtaggtttcccgtacaggctcgttgatttctgccagttcgcccagctctttcaggtaatcgttcatcttctggttgctgatgacgggcagagccatgtaattctcgaaatggatgtccttgtatttgtccagtatggctttgctgtatttgttcagttcaatcgtcaggctgtcggcagtcttgactgtggttatttcgatgtggtcggacttcacatcgcttcttttcagattgcgaacatccgaataccgcaaactcgtaaagcagcagaacaggaaaacatcacgcacacgttccaggtattgcttatccttgggtatctggtagtctttcagcttgttcagttcatcccaagtcaggaagattacttttttcgaggtggttttcagtttcggtttgaacgtatcgtatgcaatgttctgatgatgtcctttcttgaagctccagcgcaggaaccatttgaggaatcccatttgcttgccgatggtgctgtttctcatatccttggtgtcacgcaggaagttgacgtattcgttcaatccaaactcgttgaaatagttgaacgttgcatcctccttgaactctttgaggtggttcctcactgctgcaaatttttcataggtggatgccgtccagttattctggttaccgcactcttttacaaactcatcgaacacctcccaaaagctgacaggggcttcttccggctgttcttcgctggtgtctttcattctcatgttgaaagcttccttcaactgttgggtcgttggcatgacctcctgcacctcaaattccttgaaaatattctggatttcggcatagtatttcagcaagtccgtattgatttcggctgcactttgctttagcttgttggtacatccgctctttacccgctgcttatctgcatcccatttggctacgtcaatccggtagcccgttgtaaactcgatgcgttggctggcaaagatgacacgcatacggatgggtacgttctctacgattggcacaccgttctttttccggctctccaatgcaaaaatgatgttgcgcttgatattcataattgggtgcgtttgaaattctacacccaaatatacacccaattattgagatagcaaaagacatttagaaacatttacttttactctatattgtaatttacacttgattatcagtcgtttgcagtcttatgatattctgtgaaagtataagttcgagagcctgtctctccgcaaaaaacgctgaaaatcagcagattgcaaaacaaacaccctgttttacacccaagaatgtaaagtcggctgtttttgttttatttaagataatacaaccactacataataaaagagtagcgatattaaaagaatccgatgagaaaagactaatatttatctatccattcagtttgatttttcaggactttacatcgtcctgaaagtatttgttggtaccggtaccgaggacgcgtaaacatttacagttgcatgtggcctattgtttttagccgttaaatattttataactattaaatagcgatacaaattgttcgaaactaatattgtttatatcatatattctcgcatgttttaaagctttattaaattgattttttgtaaacagtttttcgtactctttgttaacccatttcattacaaaagtttcatatttttttctctctttaaatgccatttttgctggctttctttttaatacaattaatgtgctatccactttaggttttggatggaaataatacctaggaatttttgctaatatagaaatatctacctctgccattaacagcaatgctagtgatctgtttgtatctaataacattttagcaaaaccatattccactattaaataacttattgtggctgaactttcaaaaacaatttttcgaattatatttgtgcttatgttgtaaggtatgctgccaaatattttatatggattgtggctaggaaatgtaaatttcagtatatcatcatttactatttgatagttaggataatttaagagcttattacgagttacctcacataatttagaatcaatttctatcgccgttacaaaattacatctctttaccaatccagcagtaaaatgacctttccctgcacctatttcaaagatgttatctttttcatctaaacttatgcaattcattattttttctatgtgatattttgaagtaataaaattttgactatcttttatatttactttgttcattataacctctccttaatttattgcatctcttttcgaatatttatgttttttgagaaaagaacgtactcatggttcatcccgatatgcgtatcggtctgtatatcagcaactttctatgtgtttcaactacaatagtcatctattctcatctttctgagtccaccccctgcaaagcccctctttacgacataaaaattcggtcggaaaaggtatgcaaaagatgtttctctctttaagagaaactcttcgggatgcaaaaatatgaaaataactccaattcaccaaattatatagcgacttttttacaaaatgctaaaatttgttgatttccgtcaagcaattgttgagcaaaaatgtcttttacgataaaatgatacctcaatatcaactgtttagcaaaacgatatttctcttaaagagagaaacacctttttgttcaccaatccccgacttttaatcccgcggccatgattgaaaaaggaagagtatgagtattcaacatttccgtgtcgcccttattcccttttttgcggcattttgccttcctgtttttgctcacccagaaacgctggtgaaagtaaaagatgctgaagatcagttgggtgcacgagtgggttacatcgaactggatctcaacagcggtaagatccttgagagttttcgccccgaagaacgttttccaatgatgagcacttttaaagttctgctatgtggcgcggtattatcccgtattgacgccgggcaagagcaactcggtcgccgcatacactattctcagaatgacttggttgagtactcaccagtcacagaaaagcatcttacggatggcatgacagtaagagaattatgcagtgctgccataaccatgagtgataacactgcggccaacttacttctgacaacgatcggaggaccgaaggagctaaccgcttttttgcacaacatgggggatcatgtaactcgccttgatcgttgggaaccggagctgaatgaagccataccaaacgacgagcgtgacaccacgatgcctgtagcaatggcaacaacgttgcgcaaactattaactggcgaactacttactctagcttcccggcaacaattaatagactggatggaggcggataaagttgcaggaccacttctgcgctcggcccttccggctggctggtttattgctgataaatctggagccggtgagcgtgggtctcgcggtatcattgcagcactggggccagatggtaagccctcccgtatcgtagttatctacacgacggggagtcaggcaactatggatgaacgaaatagacagatcgctgagataggtgcctcactgattaagcattggtaactgtcagaccaagtttactcataacgcgtcaattcgagggggatcaattccgtgataggtgggctgcccttcctggttggcttggtttcatcagccatccgcttgccctcatctgttacgccggcggtagccggccagcctcgcagagcaggattcccgttgagcaccgccaggtgcgaataagggacagtgaagaaggaacacccgctcgcgggtgggcctacttcacctatcctgcccggctgacgccgttggatacaccaaggaaagtctacacgaaccctttggcaaaatcctgtatatcgtgcgaaaaaggatggatataccgaaaaaatcgctataatgaccccgaagcagggttatgcagcggaaaacggaattgatccggccacgatgcgtccggcgtagaggatctgaagatcagcagttcaacctgttgatagtacgtactaagctctcatgtttcacgtactaagctctcatgtttaacgtactaagctctcatgtttaacgaactaaaccctcatggctaacgtactaagctctcatggctaacgtactaagctctcatgtttcacgtactaagctctcatgtttgaacaataaaattaatataaatcagcaacttaaatagcctctaaggttttaagttttataagaaaaaaaagaatatataaggcttttaaagcttttaag

>pBH205

gtttaacggttgtggacaacaagccagggatgtaacgcactgagaagcccttagagcctctcaaagcaattttgagtgacacaggaacacttaacggctgacatgggaattcccctccaccgcggtggtacaaagaaaattcgacaaactgttatttttctatctatttatttgaattTTAAGCGCTTAAaatttacctttgtcggcaattTTAAGCGCTTAAaattaaataaagatattctcgtcaaacaaatataaataatataaacatggtttttactctggaagattttgttggcgattggcgtcagaccgcgggttataatttggatcaagtcctggaacagggtggcgtaagctctctgttccagaacctgggtgtgagcgtgacgccgattcagcgcatcgttctgtccggcgagaacggtctgaaaattgatattcatgtgatcatcccgtacgaaggcctgagcggtgaccaaatgggtcaaatcgagaaaatctttaaagtcgtctacccagttgacgatcaccacttcaaggttatcttgcattacggtacgctggtgattgatggtgtgaccccgaatatgattgactatttcggccgtccgtatgaaggcattgccgtttttgacggtaaaaagatcaccgtcaccggtaccctgtggaatggcaataagattattgacgagcgtctgattaacccggacggcagcctgctgttccgcgtgaccatcaacggtgtcacgggttggcgtctgtgcgagcgcatcctggcataatgaactgcacttgctttgataattaatgataaacaatctaaaagcactctaatcgttatcggagtgcttttagattactaatcaaattgcttctactaattgcctatcttccagtgatggaacagcatttgtgcattggctgcaacaatcagccttgatctggaagaagcaatgaaagctgctgttaagtctccgaatcaggtattgttcctgacaggtgtattcccatccggtaaacgcggatactttgcagttgatctgactcaggaataaattataaattaaggtaagaagattgtaggataagctaatgaaatagaaaaaggatgccgtcacacaacttgtcggcattcttttttgttttattagttgaaaatatagtgaaaaagttgcctaaatatgtatgttaacaaattatttgtcgtaactttgcactccaaatctgtttttaacatatggcactagtgAAACCAGTAACGTTATACGATGTCGCAGAGTATGCCGGTGTCTCTACCGCGACCGTTTCCAACGTGGTGAACCAGGCCAGCCACGTTTCTGCGAAAACGCGGGAAAAAGTGGAAGCGGCGATGGCGGAGCTGAATTACATTCCCAACCGCGTGGCACAACAACTGGCGGGCAAACAGTCGTTGCTGATTGGCGTTGCCACCTCCAGTCTGGCCCTGCACGCGCTGTCGCAAATTGTCGCGGCGATTAAATCTCGCGCCTATCAACTGGGTGCCAGCGTGTTCGTGTCGATGGTAGAACGAAGCGGCATCGAAGCCTGTAAAACGGCGGTGCACAATCTTCTCGCGCAACGCGTCAGTGGGCTGATCATTAACTATCCGCTGGATAACCAGGATGCCATTGCTGTGGAAGCTGCCTGCACTAATGTTCCGGCGTTATTTCTTGATGTCTCTGACCAGACACCCATCAACAGTATTATTTTCTCCCATGAAGACGGTACGCGACTGGGCGTGGAGCATCTGGTCGCATTGGGTCACCAGCAAATCGCGCTGTTAGCGGGCCCATTAAGTTCTGTCTCGGCGCGTCTGCGTCTGGCTGGCTGGCATAAATATCTCACTCGCAATCAAATTCAGCCGATAGCGGAACGGGAAGGCGACTGGAGTGCCATGTCCGGTTTTCAACAAACCATGCAAATGCTGAATGAGGGCATCGTTCCCACTGCGATGCTGGTTGCCAACGATCAGATGGCGCTGGGCGCAATGCGCGCCATTACCGAGACCGGGCTGCGCGTTGGTGCGGATATCTCGGTAGTGGGATACGACGATACCGAAGACAGCTCATGTTATATCCCGCCGTTAACCACCATCAAACAGGATTTTCGCCTGCTGGGGCAAACCAGCGTGGACCGCTTGCTGCAACTCTCTCAGGGCCAGGCGGTGAAGGGCAATCAGCTGTTGCCCGTCTCACTGGTGAAAAGAAAAACCACCCTGGCGCCCAATACGCAAACCGCCTCTCCCCGCGCGTTGGCCGATTCATTAATGCAGCTGGCACGACAGGTTTCCCGACTGGAAAGCGGGCAGtgagctttcctcggtaccaaattccagaaaagaggcctcccgaaaggggggccttttttcgttttggtcctacttgtgcctgttctatttccgaaccgaccgcttgtatgaatccatcaaaattcgttttctctatgttggattccttgtttgatctggaagaagcaatgaaagctgctgttaagtctccgaatcaggtattgttcctgacaggtgtattcccatccggtaaacgcggatactttgcagttgatctgactcaggaataaattataaattaaggtaagaagattgtaggataagctaatgaaatagaaaaaggatgccgtcacacaacttgtcggcattcttttttgttttattagttgaaaatatagtgaaaaagttgcctaaatatgtatgttaacaaattatttgtcgtaactttgcactccaaatctgtttttaacatatggcactagtgAAACCAGTAACGTTATACGATGTCGCAGAGTATGCCGGTGTCTCTACCGCGACCGTTTCCAACGTGGTGAACCAGGCCAGCCACGTTTCTGCGAAAACGCGGGAAAAAGTGGAAGCGGCGATGGCGGAGCTCAATTACATTCCCAACCGCGTGGCACAACAACTGGCGGGCAAAGCGTCGCATACCATTGGCATGTTGATCACTGCCAGTACCAATCCTTTCTATTCAGAACTGGTGCGTGGCGTTGAACGCAGCTGCTTCGAACGCGGTTATAGTCTCGatCTTTGCAATACCGAAGGCGATGAACAGCGGATGAATCGCAATCTGGAAACGCTGATGCAAAAACGCGTTGATGGCTTGCTGTTACTGTGCACCGAAACGCATCAACCTTCGCGTGAAATCATGCAACGTTATCCGACAGTGCCTACTGTGATGATGGACTGGGCTCCGTTCGATGGCGACAGCGATCTTATTCAGGATAACTCGTTGCTGGGCGGAGACTTAGCAACGCAATATCTGATCGATAAAGGTCATACCCGTATCGCCTGTATTACCGGCCCGCTGGATAAAACTCCGGCGCGCCTGCGGTTGGAAGGTTATCGGGCGGCGATGAAACGTGCGGGTCTCAACATTCCTGATGGCTATGAAGTCACTGGTGATTTTGAATTTAACGGCGGGTTTGACGCTATGCGCCAACTGCTATCACATCCGCTGCGTCCTCAGGCCGTCTTTACCGGAAATGACGCTATGGCTGTTGGCGTTTACCAGGCGTTATATCAGGCAGAGTTACAGGTTCCGCAGGATATCGCGGTGATTGGCTATGACGATATCGAACTGGCAAGCTTTATGACGCCACCATTAACCACTATCCACCAACCGAAAGATGAACTGGGGGAGCTGGCGATTGATGTACTCATCCATCGGATAACCCAGCCGACCCTTCAGCAACAACGATTACAACTTACTCCGATTCTGATGGAACGCGGTTCGGCTTAGCTGGTGAAAAGAAAAACCACCCTGGCGCCCAATACGCAAACCGCCTCTCCCCGCGCGTTGGCCGATTCATTAATGCAGCTGGCACGACAGGTTTCCCGACTGGAAAGCGGGCAGTGAgctttcctcggtaccaaattccagaaaagaggcctcccgaaaggggggccttttttcgttttggtccgctcatattgtgatgataatttctacaaatatagtcattggtaactatctatgaaactgtttgatacttttatagttgattaaacttgttcatggcatttgccttaatatcatccgctatgtcaatgtagggtttcatagctttgtagtcgctgtgtcccgtccatttcatgaccacctgtgccgggattccgagagccagcgcattgcagatgaatgtccttcttcctgcatgggtactgagcaaagcgtatttgggtgtgacttcatcaatacgttcatttcccttgtagtaggtttcccgtacaggctcgttgatttctgccagttcgcccagctctttcaggtaatcgttcatcttctggttgctgatgacgggcagagccatgtaattctcgaaatggatgtccttgtatttgtccagtatggctttgctgtatttgttcagttcaatcgtcaggctgtcggcagtcttgactgtggttatttcgatgtggtcggacttcacatcgcttcttttcagattgcgaacatccgaataccgcaaactcgtaaagcagcagaacaggaaaacatcacgcacacgttccaggtattgcttatccttgggtatctggtagtctttcagcttgttcagttcatcccaagtcaggaagattacttttttcgaggtggttttcagtttcggtttgaacgtatcgtatgcaatgttctgatgatgtcctttcttgaagctccagcgcaggaaccatttgaggaatcccatttgcttgccgatggtgctgtttctcatatccttggtgtcacgcaggaagttgacgtattcgttcaatccaaactcgttgaaatagttgaacgttgcatcctccttgaactctttgaggtggttcctcactgctgcaaatttttcataggtggatgccgtccagttattctggttaccgcactcttttacaaactcatcgaacacctcccaaaagctgacaggggcttcttccggctgttcttcgctggtgtctttcattctcatgttgaaagcttccttcaactgttgggtcgttggcatgacctcctgcacctcaaattccttgaaaatattctggatttcggcatagtatttcagcaagtccgtattgatttcggctgcactttgctttagcttgttggtacatccgctctttacccgctgcttatctgcatcccatttggctacgtcaatccggtagcccgttgtaaactcgatgcgttggctggcaaagatgacacgcatacggatgggtacgttctctacgattggcacaccgttctttttccggctctccaatgcaaaaatgatgttgcgcttgatattcataattgggtgcgtttgaaattctacacccaaatatacacccaattattgagatagcaaaagacatttagaaacatttacttttactctatattgtaatttacacttgattatcagtcgtttgcagtcttatgatattctgtgaaagtataagttcgagagcctgtctctccgcaaaaaacgctgaaaatcagcagattgcaaaacaaacaccctgttttacacccaagaatgtaaagtcggctgtttttgttttatttaagataatacaaccactacataataaaagagtagcgatattaaaagaatccgatgagaaaagactaatatttatctatccattcagtttgatttttcaggactttacatcgtcctgaaagtatttgttggtaccggtaccgaggacgcgtaaacatttacagttgcatgtggcctattgtttttagccgttaaatattttataactattaaatagcgatacaaattgttcgaaactaatattgtttatatcatatattctcgcatgttttaaagctttattaaattgattttttgtaaacagtttttcgtactctttgttaacccatttcattacaaaagtttcatatttttttctctctttaaatgccatttttgctggctttctttttaatacaattaatgtgctatccactttaggttttggatggaaataatacctaggaatttttgctaatatagaaatatctacctctgccattaacagcaatgctagtgatctgtttgtatctaataacattttagcaaaaccatattccactattaaataacttattgtggctgaactttcaaaaacaatttttcgaattatatttgtgcttatgttgtaaggtatgctgccaaatattttatatggattgtggctaggaaatgtaaatttcagtatatcatcatttactatttgatagttaggataatttaagagcttattacgagttacctcacataatttagaatcaatttctatcgccgttacaaaattacatctctttaccaatccagcagtaaaatgacctttccctgcacctatttcaaagatgttatctttttcatctaaacttatgcaattcattattttttctatgtgatattttgaagtaataaaattttgactatcttttatatttactttgttcattataacctctccttaatttattgcatctcttttcgaatatttatgttttttgagaaaagaacgtactcatggttcatcccgatatgcgtatcggtctgtatatcagcaactttctatgtgtttcaactacaatagtcatctattctcatctttctgagtccaccccctgcaaagcccctctttacgacataaaaattcggtcggaaaaggtatgcaaaagatgtttctctctttaagagaaactcttcgggatgcaaaaatatgaaaataactccaattcaccaaattatatagcgacttttttacaaaatgctaaaatttgttgatttccgtcaagcaattgttgagcaaaaatgtcttttacgataaaatgatacctcaatatcaactgtttagcaaaacgatatttctcttaaagagagaaacacctttttgttcaccaatccccgacttttaatcccgcggccatgattgaaaaaggaagagtatgagtattcaacatttccgtgtcgcccttattcccttttttgcggcattttgccttcctgtttttgctcacccagaaacgctggtgaaagtaaaagatgctgaagatcagttgggtgcacgagtgggttacatcgaactggatctcaacagcggtaagatccttgagagttttcgccccgaagaacgttttccaatgatgagcacttttaaagttctgctatgtggcgcggtattatcccgtattgacgccgggcaagagcaactcggtcgccgcatacactattctcagaatgacttggttgagtactcaccagtcacagaaaagcatcttacggatggcatgacagtaagagaattatgcagtgctgccataaccatgagtgataacactgcggccaacttacttctgacaacgatcggaggaccgaaggagctaaccgcttttttgcacaacatgggggatcatgtaactcgccttgatcgttgggaaccggagctgaatgaagccataccaaacgacgagcgtgacaccacgatgcctgtagcaatggcaacaacgttgcgcaaactattaactggcgaactacttactctagcttcccggcaacaattaatagactggatggaggcggataaagttgcaggaccacttctgcgctcggcccttccggctggctggtttattgctgataaatctggagccggtgagcgtgggtctcgcggtatcattgcagcactggggccagatggtaagccctcccgtatcgtagttatctacacgacggggagtcaggcaactatggatgaacgaaatagacagatcgctgagataggtgcctcactgattaagcattggtaactgtcagaccaagtttactcataacgcgtcaattcgagggggatcaattccgtgataggtgggctgcccttcctggttggcttggtttcatcagccatccgcttgccctcatctgttacgccggcggtagccggccagcctcgcagagcaggattcccgttgagcaccgccaggtgcgaataagggacagtgaagaaggaacacccgctcgcgggtgggcctacttcacctatcctgcccggctgacgccgttggatacaccaaggaaagtctacacgaaccctttggcaaaatcctgtatatcgtgcgaaaaaggatggatataccgaaaaaatcgctataatgaccccgaagcagggttatgcagcggaaaacggaattgatccggccacgatgcgtccggcgtagaggatctgaagatcagcagttcaacctgttgatagtacgtactaagctctcatgtttcacgtactaagctctcatgtttaacgtactaagctctcatgtttaacgaactaaaccctcatggctaacgtactaagctctcatggctaacgtactaagctctcatgtttcacgtactaagctctcatgtttgaacaataaaattaatataaatcagcaacttaaatagcctctaaggttttaagttttataagaaaaaaaagaatatataaggcttttaaagcttttaag

>pBH206

gtttaacggttgtggacaacaagccagggatgtaacgcactgagaagcccttagagcctctcaaagcaattttgagtgacacaggaacacttaacggctgacatgggaattcccctccaccgcggtggtacaaagaaaattcgacaaactgttatttttctatctatttatttgAATTaggAGCGCTcctAATTtacctttgtcggcAATTaggAGCGCTcctAATTaaataaagatattctcgtcaaacaaatataaataatataaacatggtttttactctggaagattttgttggcgattggcgtcagaccgcgggttataatttggatcaagtcctggaacagggtggcgtaagctctctgttccagaacctgggtgtgagcgtgacgccgattcagcgcatcgttctgtccggcgagaacggtctgaaaattgatattcatgtgatcatcccgtacgaaggcctgagcggtgaccaaatgggtcaaatcgagaaaatctttaaagtcgtctacccagttgacgatcaccacttcaaggttatcttgcattacggtacgctggtgattgatggtgtgaccccgaatatgattgactatttcggccgtccgtatgaaggcattgccgtttttgacggtaaaaagatcaccgtcaccggtaccctgtggaatggcaataagattattgacgagcgtctgattaacccggacggcagcctgctgttccgcgtgaccatcaacggtgtcacgggttggcgtctgtgcgagcgcatcctggcataatgaactgcacttgctttgataattaatgataaacaatctaaaagcactctaatcgttatcggagtgcttttagattactaatcaaattgcttctactaattgcctatcttccagtgatggaacagcatttgtgcattggctgcaacaatcagccttgatctggaagaagcaatgaaagctgctgttaagtctccgaatcaggtattgttcctgacaggtgtattcccatccggtaaacgcggatactttgcagttgatctgactcaggaataaattataaattaaggtaagaagattgtaggataagctaatgaaatagaaaaaggatgccgtcacacaacttgtcggcattcttttttgttttattagttgaaaatatagtgaaaaagttgcctaaatatgtatgttaacaaattatttgtcgtaactttgcactccaaatctgtttttaacatatggcactagtgAAACCAGTAACGTTATACGATGTCGCAGAGTATGCCGGTGTCTCTAAAAGCACCGTTTCCCTGGTGGTGAACCAGGCCAGCCACGTTTCTGCGAAAACGCGGGAAAAAGTGGAAGCGGCGATGGCGGAGCTGAATTACATTCCCAACCGCGTGGCACAACAACTGGCGGGCAAACAGTCGTTGCTGATTGGCGTTGCCACCTCCAGTCTGGCCCTGCACGCGCTGTCGCAAATTGTCGCGGCGATTAAATCTCGCGCCTATCAACTGGGTGCCAGCGTGTTCGTGTCGATGGTAGAACGAAGCGGCATCGAAGCCTGTAAAACGGCGGTGCACAATCTTCTCGCGCAACGCGTCAGTGGGCTGATCATTAACTATCCGCTGGATAACCAGGATGCCATTGCTGTGGAAGCTGCCTGCACTAATGTTCCGGCGTTATTTCTTGATGTCTCTGACCAGACACCCATCAACAGTATTATTTTCTCCCATGAAGACGGTACGCGACTGGGCGTGGAGCATCTGGTCGCATTGGGTCACCAGCAAATCGCGCTGTTAGCGGGCCCATTAAGTTCTGTCTCGGCGCGTCTGCGTCTGGCTGGCTGGCATAAATATCTCACTCGCAATCAAATTCAGCCGATAGCGGAACGGGAAGGCGACTGGAGTGCCATGTCCGGTTTTCAACAAACCATGCAAATGCTGAATGAGGGCATCGTTCCCACTGCGATGCTGGTTGCCAACGATCAGATGGCGCTGGGCGCAATGCGCGCCATTACCGAGACCGGGCTGCGCGTTGGTGCGGATATCTCGGTAGTGGGATACGACGATACCGAAGACAGCTCATGTTATATCCCGCCGTTAACCACCATCAAACAGGATTTTCGCCTGCTGGGGCAAACCAGCGTGGACCGCTTGCTGCAACTCTCTCAGGGCCAGGCGGTGAAGGGCAATCAGCTGTTGCCCGTCTCACTGGTGAAAAGAAAAACCACCCTGGCGCCCAATACGCAAACCGCCTCTCCCCGCGCGTTGGCCGATTCATTAATGCAGCTGGCACGACAGGTTTCCCGACTGGAAAGCGGGCAGtgagctttcctcggtaccaaattccagaaaagaggcctcccgaaaggggggccttttttcgttttggtcctacttgtgcctgttctatttccgaaccgaccgcttgtatgaatccatcaaaattcgttttctctatgttggattccttgtttgatctggaagaagcaatgaaagctgctgttaagtctccgaatcaggtattgttcctgacaggtgtattcccatccggtaaacgcggatactttgcagttgatctgactcaggaataaattataaattaaggtaagaagattgtaggataagctaatgaaatagaaaaaggatgccgtcacacaacttgtcggcattcttttttgttttattagttgaaaatatagtgaaaaagttgcctaaatatgtatgttaacaaattatttgtcgtaactttgcactccaaatctgtttttaacatatggcactagtgAAACCAGTAACGTTATACGATGTCGCAGAGTATGCCGGTGTCTCTAAAAGCACCGTTTCCCTGGTGGTGAACCAGGCCAGCCACGTTTCTGCGAAAACGCGGGAAAAAGTGGAAGCGGCGATGGCGGAGCTCAATTACATTCCCAACCGCGTGGCACAACAACTGGCGGGCAAAGCGTCGCATACCATTGGCATGTTGATCACTGCCAGTACCAATCCTTTCTATTCAGAACTGGTGCGTGGCGTTGAACGCAGCTGCTTCGAACGCGGTTATAGTCTCGatCTTTGCAATACCGAAGGCGATGAACAGCGGATGAATCGCAATCTGGAAACGCTGATGCAAAAACGCGTTGATGGCTTGCTGTTACTGTGCACCGAAACGCATCAACCTTCGCGTGAAATCATGCAACGTTATCCGACAGTGCCTACTGTGATGATGGACTGGGCTCCGTTCGATGGCGACAGCGATCTTATTCAGGATAACTCGTTGCTGGGCGGAGACTTAGCAACGCAATATCTGATCGATAAAGGTCATACCCGTATCGCCTGTATTACCGGCCCGCTGGATAAAACTCCGGCGCGCCTGCGGTTGGAAGGTTATCGGGCGGCGATGAAACGTGCGGGTCTCAACATTCCTGATGGCTATGAAGTCACTGGTGATTTTGAATTTAACGGCGGGTTTGACGCTATGCGCCAACTGCTATCACATCCGCTGCGTCCTCAGGCCGTCTTTACCGGAAATGACGCTATGGCTGTTGGCGTTTACCAGGCGTTATATCAGGCAGAGTTACAGGTTCCGCAGGATATCGCGGTGATTGGCTATGACGATATCGAACTGGCAAGCTTTATGACGCCACCATTAACCACTATCCACCAACCGAAAGATGAACTGGGGGAGCTGGCGATTGATGTACTCATCCATCGGATAACCCAGCCGACCCTTCAGCAACAACGATTACAACTTACTCCGATTCTGATGGAACGCGGTTCGGCTTAGCTGGTGAAAAGAAAAACCACCCTGGCGCCCAATACGCAAACCGCCTCTCCCCGCGCGTTGGCCGATTCATTAATGCAGCTGGCACGACAGGTTTCCCGACTGGAAAGCGGGCAGTGAgctttcctcggtaccaaattccagaaaagaggcctcccgaaaggggggccttttttcgttttggtccgctcatattgtgatgataatttctacaaatatagtcattggtaactatctatgaaactgtttgatacttttatagttgattaaacttgttcatggcatttgccttaatatcatccgctatgtcaatgtagggtttcatagctttgtagtcgctgtgtcccgtccatttcatgaccacctgtgccgggattccgagagccagcgcattgcagatgaatgtccttcttcctgcatgggtactgagcaaagcgtatttgggtgtgacttcatcaatacgttcatttcccttgtagtaggtttcccgtacaggctcgttgatttctgccagttcgcccagctctttcaggtaatcgttcatcttctggttgctgatgacgggcagagccatgtaattctcgaaatggatgtccttgtatttgtccagtatggctttgctgtatttgttcagttcaatcgtcaggctgtcggcagtcttgactgtggttatttcgatgtggtcggacttcacatcgcttcttttcagattgcgaacatccgaataccgcaaactcgtaaagcagcagaacaggaaaacatcacgcacacgttccaggtattgcttatccttgggtatctggtagtctttcagcttgttcagttcatcccaagtcaggaagattacttttttcgaggtggttttcagtttcggtttgaacgtatcgtatgcaatgttctgatgatgtcctttcttgaagctccagcgcaggaaccatttgaggaatcccatttgcttgccgatggtgctgtttctcatatccttggtgtcacgcaggaagttgacgtattcgttcaatccaaactcgttgaaatagttgaacgttgcatcctccttgaactctttgaggtggttcctcactgctgcaaatttttcataggtggatgccgtccagttattctggttaccgcactcttttacaaactcatcgaacacctcccaaaagctgacaggggcttcttccggctgttcttcgctggtgtctttcattctcatgttgaaagcttccttcaactgttgggtcgttggcatgacctcctgcacctcaaattccttgaaaatattctggatttcggcatagtatttcagcaagtccgtattgatttcggctgcactttgctttagcttgttggtacatccgctctttacccgctgcttatctgcatcccatttggctacgtcaatccggtagcccgttgtaaactcgatgcgttggctggcaaagatgacacgcatacggatgggtacgttctctacgattggcacaccgttctttttccggctctccaatgcaaaaatgatgttgcgcttgatattcataattgggtgcgtttgaaattctacacccaaatatacacccaattattgagatagcaaaagacatttagaaacatttacttttactctatattgtaatttacacttgattatcagtcgtttgcagtcttatgatattctgtgaaagtataagttcgagagcctgtctctccgcaaaaaacgctgaaaatcagcagattgcaaaacaaacaccctgttttacacccaagaatgtaaagtcggctgtttttgttttatttaagataatacaaccactacataataaaagagtagcgatattaaaagaatccgatgagaaaagactaatatttatctatccattcagtttgatttttcaggactttacatcgtcctgaaagtatttgttggtaccggtaccgaggacgcgtaaacatttacagttgcatgtggcctattgtttttagccgttaaatattttataactattaaatagcgatacaaattgttcgaaactaatattgtttatatcatatattctcgcatgttttaaagctttattaaattgattttttgtaaacagtttttcgtactctttgttaacccatttcattacaaaagtttcatatttttttctctctttaaatgccatttttgctggctttctttttaatacaattaatgtgctatccactttaggttttggatggaaataatacctaggaatttttgctaatatagaaatatctacctctgccattaacagcaatgctagtgatctgtttgtatctaataacattttagcaaaaccatattccactattaaataacttattgtggctgaactttcaaaaacaatttttcgaattatatttgtgcttatgttgtaaggtatgctgccaaatattttatatggattgtggctaggaaatgtaaatttcagtatatcatcatttactatttgatagttaggataatttaagagcttattacgagttacctcacataatttagaatcaatttctatcgccgttacaaaattacatctctttaccaatccagcagtaaaatgacctttccctgcacctatttcaaagatgttatctttttcatctaaacttatgcaattcattattttttctatgtgatattttgaagtaataaaattttgactatcttttatatttactttgttcattataacctctccttaatttattgcatctcttttcgaatatttatgttttttgagaaaagaacgtactcatggttcatcccgatatgcgtatcggtctgtatatcagcaactttctatgtgtttcaactacaatagtcatctattctcatctttctgagtccaccccctgcaaagcccctctttacgacataaaaattcggtcggaaaaggtatgcaaaagatgtttctctctttaagagaaactcttcgggatgcaaaaatatgaaaataactccaattcaccaaattatatagcgacttttttacaaaatgctaaaatttgttgatttccgtcaagcaattgttgagcaaaaatgtcttttacgataaaatgatacctcaatatcaactgtttagcaaaacgatatttctcttaaagagagaaacacctttttgttcaccaatccccgacttttaatcccgcggccatgattgaaaaaggaagagtatgagtattcaacatttccgtgtcgcccttattcccttttttgcggcattttgccttcctgtttttgctcacccagaaacgctggtgaaagtaaaagatgctgaagatcagttgggtgcacgagtgggttacatcgaactggatctcaacagcggtaagatccttgagagttttcgccccgaagaacgttttccaatgatgagcacttttaaagttctgctatgtggcgcggtattatcccgtattgacgccgggcaagagcaactcggtcgccgcatacactattctcagaatgacttggttgagtactcaccagtcacagaaaagcatcttacggatggcatgacagtaagagaattatgcagtgctgccataaccatgagtgataacactgcggccaacttacttctgacaacgatcggaggaccgaaggagctaaccgcttttttgcacaacatgggggatcatgtaactcgccttgatcgttgggaaccggagctgaatgaagccataccaaacgacgagcgtgacaccacgatgcctgtagcaatggcaacaacgttgcgcaaactattaactggcgaactacttactctagcttcccggcaacaattaatagactggatggaggcggataaagttgcaggaccacttctgcgctcggcccttccggctggctggtttattgctgataaatctggagccggtgagcgtgggtctcgcggtatcattgcagcactggggccagatggtaagccctcccgtatcgtagttatctacacgacggggagtcaggcaactatggatgaacgaaatagacagatcgctgagataggtgcctcactgattaagcattggtaactgtcagaccaagtttactcataacgcgtcaattcgagggggatcaattccgtgataggtgggctgcccttcctggttggcttggtttcatcagccatccgcttgccctcatctgttacgccggcggtagccggccagcctcgcagagcaggattcccgttgagcaccgccaggtgcgaataagggacagtgaagaaggaacacccgctcgcgggtgggcctacttcacctatcctgcccggctgacgccgttggatacaccaaggaaagtctacacgaaccctttggcaaaatcctgtatatcgtgcgaaaaaggatggatataccgaaaaaatcgctataatgaccccgaagcagggttatgcagcggaaaacggaattgatccggccacgatgcgtccggcgtagaggatctgaagatcagcagttcaacctgttgatagtacgtactaagctctcatgtttcacgtactaagctctcatgtttaacgtactaagctctcatgtttaacgaactaaaccctcatggctaacgtactaagctctcatggctaacgtactaagctctcatgtttcacgtactaagctctcatgtttgaacaataaaattaatataaatcagcaacttaaatagcctctaaggttttaagttttataagaaaaaaaagaatatataaggcttttaaagcttttaag

>pBH207

gtttaacggttgtggacaacaagccagggatgtaacgcactgagaagcccttagagcctctcaaagcaattttgagtgacacaggaacacttaacggctgacatgggaattcccctccaccgcggtggCCCTgtacaaagaaaattcgacaaactgttatttttctatctatttatttgaattgtgagcggataacaattacctttgtcggcaattgtgagcggataacaattaaataaagatattctcgtcaaacaaatataaataatataaacatggtttttactctggaagattttgttggcgattggcgtcagaccgcgggttataatttggatcaagtcctggaacagggtggcgtaagctctctgttccagaacctgggtgtgagcgtgacgccgattcagcgcatcgttctgtccggcgagaacggtctgaaaattgatattcatgtgatcatcccgtacgaaggcctgagcggtgaccaaatgggtcaaatcgagaaaatctttaaagtcgtctacccagttgacgatcaccacttcaaggttatcttgcattacggtacgctggtgattgatggtgtgaccccgaatatgattgactatttcggccgtccgtatgaaggcattgccgtttttgacggtaaaaagatcaccgtcaccggtaccctgtggaatggcaataagattattgacgagcgtctgattaacccggacggcagcctgctgttccgcgtgaccatcaacggtgtcacgggttggcgtctgtgcgagcgcatcctggcataatgaactgcacttgctttgataattaatgataaacaatctaaaagcactctaatcgttatcggagtgcttttagattactaatcaaattgcttctactaattgcctatcttccagtgatggaacagcatttgAACGcattggctgcaacaatcagccttgatctggaagaagcaatgaaagctgctgttaagtctccgaatcaggtattgttcctgacaggtgtattcccatccggtaaacgcggatactttgcagttgatctgactcaggaataaattataaattaaggtaagaagattgtaggataagctaatgaaatagaaaaaggatgccgtcacacaacttgtcggcattcttttttgttttattagttgaaaatatagtgaaaaagttgcctaaatatgtatgttaacaaattatttgtcgtaactttgcactccaaatctgtttttaacatatggcactagtgAAACCAGTAACGTTATACGATGTCGCAGAGTATGCCGGTGTCTCTTATCAGACCGTTTCCCGCGTGGTGAACCAGGCCAGCCACGTTTCTGCGAAAACGCGGGAAAAAGTGGAAGCGGCGATGGCGGAGCTGAATTACATTCCCAACCGCGTGGCACAACAACTGGCGGGCAAACAGTCGTTGCTGATTGGCGTTGCCACCTCCAGTCTGGCCCTGCACGCGCCGTCGCAAATTGTCGCGGCGATTAAATCTCGCGCCGATCAACTGGGTGCCAGCGTGGTGGTGTCGATGGTAGAACGAAGCGGCGTCGAAGCCTGTAAAACGGCGGTGCACAATCTTCTCGCGCAACGCGTCAGTGGGCTGATCATTAACTATCCGCTGGATGACCAGGATGCCATTGCTGTGGAAGCTGCCTGCACTAATGTTCCGGCGTTATTTCTTGATGTCTCTGACCAGACACCCATCAACAGTATTATTTTCTCCCATGAAGACGGTACGCGACTGGGCGTGGAGCATCTGGTCGCATTGGGTCACCAGCAAATCGCGCTGTTAGCGGGCCCATTAAGTTCTGTCTCGGCGCGTCTGCGTCTGGCTGGCTGGCATAAATATCTCACTCGCAATCAAATTCAGCCGATAGCGGAACGGGAAGGCGACTGGAGTGCCATGTCCGGTTTTCAACAAACCATGCAAATGCTGAATGAGGGCATCGTTCCCACTGCGATGCTGGTTGCCAACGATCAGATGGCGCTGGGCGCAATGCGCGCCATTACCGAGTCCGGGCTGCGCGTTGGTGCGGATATCTCGGTAGTGGGATACGACGATACCGAAGACAGCTCATGTTATATCCCGCCGTTAACCACCATCAAACAGGATTTTCGCCTGCTGGGGCAAACCAGCGTGGACCGCTTGCTGCAACTCTCTCAGGGCCAGGCGGTGAAGGGCAATCAGCTGTTGCCCGTCTCACTGGTGAAAAGAAAAACCACCCTGGCGCCCAATACGCAAACCGCCTCTCCCCGCGCGTTGGCCGATTCATTAATGCAGCTGGCACGACAGGTTTCCCGACTGGAAAGCGGGCAGtgagtgcaacaatcagccttgatctggaagaagcaatgaaagctgctgttaagtctccgaatcaggtattgttcctgacaggtgtattcccatccggtaaacgcggatactttgcagttgatctgactcaggaataaattataaattaaggtaagaagattgtaggataagctaatgaaatagaaaaaggatgccgtcacacaacttgtcggcattcttttttgttttattagttgaaaatatagtgaaaaagttgcctaaatatgtatgttaacaaattatttgtcgtaactttgcactccaaatctgtttttaacatatggcactaGTGAAACCAGTAACGTTATACGATGTCGCAGAGTATGCCGGTGTCTCTTATCAGACCGTTTCCCGCGTGGTGAACCAGGCCAGCCACGTTTCTGCGAAAACGCGGGAAAAAGTGGAAGCGGCGATGGCGGAGCTCAATTACATTCCCAACCGCGTGGCACAACAACTGGCGGGCAAAGCGTCGCATACCATTGGCATGTTGATCACTGCCAGTACCAATCCTTTCTATTCAGAACTGGTGCGTGGCGTTGAACGCAGCTGCTTCGAACGCGGTTATAGTCTCGatCTTTGCAATACCGAAGGCGATGAACAGCGGATGAATCGCAATCTGGAAACGCTGATGCAAAAACGCGTTGATGGCTTGCTGTTACTGTGCACCGAAACGCATCAACCTTCGCGTGAAATCATGCAACGTTATCCGACAGTGCCTACTGTGATGATGGACTGGGCTCCGTTCGATGGCGACAGCGATCTTATTCAGGATAACTCGTTGCTGGGCGGAGACTTAGCAACGCAATATCTGATCGATAAAGGTCATACCCGTATCGCCTGTATTACCGGCCCGCTGGATAAAACTCCGGCGCGCCTGCGGTTGGAAGGTTATCGGGCGGCGATGAAACGTGCGGGTCTCAACATTCCTGATGGCTATGAAGTCACTGGTGATTTTGAATTTAACGGCGGGTTTGACGCTATGCGCCAACTGCTATCACATCCGCTGCGTCCTCAGGCCGTCTTTACCGGAAATGACGCTATGGCTGTTGGCGTTTACCAGGCGTTATATCAGGCAGAGTTACAGGTTCCGCAGGATATCGCGGTGATTGGCTATGACGATATCGAACTGGCAAGCTTTATGACGCCACCATTAACCACTATCCACCAACCGAAAGATGAACTGGGGGAGCTGGCGATTGATGTACTCATCCATCGGATAACCCAGCCGACCCTTCAGCAACAACGATTACAACTTACTCCGATTCTGATGGAACGCGGTTCGGCTTAGCTGGTGAAAAGAAAAACCACCCTGGCGCCCAATACGCAAACCGCCTCTCCCCGCGCGTTGGCCGATTCATTAATGCAGCTGGCACGACAGGTTTCCCGACTGGAAAGCGGGCAGTGAgctttcctcggtaccaaattccagaaaagaggcctcccgaaaggggggccttttttcgttttggtcctacttgtgcctgttctatttccATCCcggttcctggccttttgctggccttttgctcacatgttctttcctgcgttatcccctgattctgtggataaccgtattaccgcctttgagtgagctgatacCCGAtacttgtgcctgttctatttccgaaccgaccgcttgtatgaatccatcaaaattcgttttctctatgttggattccttgttgctcatattgtgatgataatttctacaaatatagtcattggtaactatctatgaaactgtttgatacttttatagttgattaaacttgttcatggcatttgccttaatatcatccgctatgtcaatgtagggtttcatagctttgtagtcgctgtgtcccgtccatttcatgaccacctgtgccgggattccgagagccagcgcattgcagatgaatgtccttcttcctgcatgggtactgagcaaagcgtatttgggtgtgacttcatcaatacgttcatttcccttgtagtaggtttcccgtacaggctcgttgatttctgccagttcgcccagctctttcaggtaatcgttcatcttctggttgctgatgacgggcagagccatgtaattctcgaaatggatgtccttgtatttgtccagtatggctttgctgtatttgttcagttcaatcgtcaggctgtcggcagtcttgactgtggttatttcgatgtggtcggacttcacatcgcttcttttcagattgcgaacatccgaataccgcaaactcgtaaagcagcagaacaggaaaacatcacgcacacgttccaggtattgcttatccttgggtatctggtagtctttcagcttgttcagttcatcccaagtcaggaagattacttttttcgaggtggttttcagtttcggtttgaacgtatcgtatgcaatgttctgatgatgtcctttcctgaagctccagcgcaggaaccatttgaggaatcccatttgcttgccgatggtgctgtttctcatatccttggtgtcacgcaggaagttgacgtattcgttcaatccaaactcgttgaaatagttgaacgttgcatcctccttgaactctttgaggtggttcctcactgctgcaaatttttcataggtggatgccgtccagttattctggttaccgcactcttttacaaactcatcgaacacctcccaaaagctgacaggggcttcttccggctgttcttcactggtgtctttcattctcatgttgaaagcttccttcaactgttgggtcgttggcatgacctcctgcacctcaaattccttgaaaatattctggatttcggcatagtatttcagcaagtccgtattgatttcggctgcactttgctttagcttgttggtacatccgttctttacccgctgcttatctacatcccatttggctacgtcaatccggtagcccgttgtaaactcgatacgttggctggcaaagatgacacgcatacggatgggtacgttctctacgattggcacaccgttctttttccggctctccaatgcaaaaatgatgttgcgcttgatattcataattgggtgcgtttgaaattctacacccaaatatacacccaattattgagatagcaaaagacatttagaaacatttacttttactctatattgtaatttacacttgattatcagtcgtttgcagtTttatgatattctgtgaaagtataagttcgagagcctgtctctccgcaaaaaacgctgaaaatcagcagattgcaaaacaaacaccctgttttacacccaagaatgtaaagtcggctgtttttgttttatttaagataatacaaccactacataataaaagagtagcgatattaaaagaatccgatgagaaaagactaatatttatctatccattcagtttgatttttcaggactttacatcgtcctgaaagtatttgttggtaccggtaccgaggacgcgtaaacatttacagttgcatgtggcctattgtttttagccgttaaatattttataactattaaatagcgatacaaattgttcgaaactaatattgtttatatcatatattctcgcatgttttaaagctttattaaattgattttttgtaaacagtttttcgtactctttgttaacccatttcattacaaaagtttcatatttttttctctctttaaatgccatttttgctggctttctttttaatacaattaatgtgctatccactttaggttttggatggaaataatacctaggaatttttgctaatatagaaatatctacctctgccattaacagcaatgctagtgatctgtttgtatctaataacattttagcaaaaccatattccactattaaataacttattgtggctgaactttcaaaaacaatttttcgaattatatttgtgcttatgttgtaaggtatgctgccaaatattttatatggattgtggctaggaaatgtaaatttcagtatatcatcatttactatttgatagttaggataatttaagagcttattacgagttacctcacataatttagaatcaatttctatcgccgttacaaaattacatctctttaccaatccagcagtaaaatgacctttccctgcacctatttcaaagatgttatctttttcatctaaacttatgcaattcattattttttctatgtgatattttgaagtaataaaattttgactatcttttatatttactttgttcattataacctctccttaatttattgcatctcttttcgaatatttatgttttttgagaaaagaacgtactcatggttcatcccgatatgcgtatcggtctgtatatcagcaactttctatgtgtttcaactacaatagtcatctattctcatctttctgagtccaccccctgcaaagcccctctttacgacataaaaattcggtcggaaaaggtatgcaaaagatgtttctctctttaagagaaactcttcgggatgcaaaaatatgaaaataactccaattcaccaaattatatagcgacttttttacaaaatgctaaaatttgttgatttccgtcaagcaattgttgagcaaaaatgtcttttacgataaaatgatacctcaatatcaactgtttagcaaaacgatatttctcttaaagagagaaacacctttttgttcaccaatccccgacttttaatcccgcggccatgattgaaaaaggaagagtatgagtattcaacatttccgtgtcgcccttattcccttttttgcggcattttgccttcctgtttttgctcacccagaaacgctggtgaaagtaaaagatgctgaagatcagttgggtgcacgagtgggttacatcgaactggatctcaacagcggtaagatccttgagagttttcgccccgaagaacgttttccaatgatgagcacttttaaagttctgctatgtggcgcggtattatcccgtattgacgccgggcaagagcaactcggtcgccgcatacactattctcagaatgacttggttgagtactcaccagtcacagaaaagcatcttacggatggcatgacagtaagagaattatgcagtgctgccataaccatgagtgataacactgcggccaacttacttctgacaacgatcggaggaccgaaggagctaaccgcttttttgcacaacatgggggatcatgtaactcgccttgatcgttgggaaccggagctgaatgaagccataccaaacgacgagcgtgacaccacgatgcctgtagcaatggcaacaacgttgcgcaaactattaactggcgaactacttactctagcttcccggcaacaattaatagactggatggaggcggataaagttgcaggaccacttctgcgctcggcccttccggctggctggtttattgctgataaatctggagccggtgagcgtgggtctcgcggtatcattgcagcactggggccagatggtaagccctcccgtatcgtagttatctacacgacggggagtcaggcaactatggatgaacgaaatagacagatcgctgagataggtgcctcactgattaagcattggtaactgtcagaccaagtttactcataacgcgtcaattcgagggggatcaattccgtgataggtgggctgcccttcctggttggcttggtttcatcagccatccgcttgccctcatctgttacgccggcggtagccggccagcctcgcagagcaggattcccgttgagcaccgccaggtgcgaataagggacagtgaagaaggaacacccgctcgcgggtgggcctacttcacctatcctgcccggctgacgccgttggatacaccaaggaaagtctacacgaaccctttggcaaaatcctgtatatcgtgcgaaaaaggatggatataccgaaaaaatcgctataatgaccccgaagcagggttatgcagcggaaaacggaattgatccggccacgatgcgtccggcgtagaggatctgaagatcagcagttcaacctgttgatagtacgtactaagctctcatgtttcacgtactaagctctcatgtttaacgtactaagctctcatgtttaacgaactaaaccctcatggctaacgtactaagctctcatggctaacgtactaagctctcatgtttcacgtactaagctctcatgtttgaacaataaaattaatataaatcagcaacttaaatagcctctaaggttttaagttttataagaaaaaaaagaatatataaggcttttaaagcttttaag

>pBH208

gtttaacggttgtggacaacaagccagggatgtaacgcactgagaagcccttagagcctctcaaagcaattttgagtgacacaggaacacttaacggctgacatgggaattcccctccaccgcggtggCCCTgtacaaagaaaattcgacaaactgttatttttctatctatttatttgaattTTAAGCGCTTAAaatttacctttgtcggcaattTTAAGCGCTTAAaattaaataaagatattctcgtcaaacaaatataaataatataaacatggtttttactctggaagattttgttggcgattggcgtcagaccgcgggttataatttggatcaagtcctggaacagggtggcgtaagctctctgttccagaacctgggtgtgagcgtgacgccgattcagcgcatcgttctgtccggcgagaacggtctgaaaattgatattcatgtgatcatcccgtacgaaggcctgagcggtgaccaaatgggtcaaatcgagaaaatctttaaagtcgtctacccagttgacgatcaccacttcaaggttatcttgcattacggtacgctggtgattgatggtgtgaccccgaatatgattgactatttcggccgtccgtatgaaggcattgccgtttttgacggtaaaaagatcaccgtcaccggtaccctgtggaatggcaataagattattgacgagcgtctgattaacccggacggcagcctgctgttccgcgtgaccatcaacggtgtcacgggttggcgtctgtgcgagcgcatcctggcataatgaactgcacttgctttgataattaatgataaacaatctaaaagcactctaatcgttatcggagtgcttttagattactaatcaaattgcttctactaattgcctatcttccagtgatggaacagcatttgAACGcattggctgcaacaatcagccttgatctggaagaagcaatgaaagctgctgttaagtctccgaatcaggtattgttcctgacaggtgtattcccatccggtaaacgcggatactttgcagttgatctgactcaggaataaattataaattaaggtaagaagattgtaggataagctaatgaaatagaaaaaggatgccgtcacacaacttgtcggcattcttttttgttttattagttgaaaatatagtgaaaaagttgcctaaatatgtatgttaacaaattatttgtcgtaactttgcactccaaatctgtttttaacatatggcactagtgAAACCAGTAACGTTATACGATGTCGCAGAGTATGCCGGTGTCTCTACCGCGACCGTTTCCAACGTGGTGAACCAGGCCAGCCACGTTTCTGCGAAAACGCGGGAAAAAGTGGAAGCGGCGATGGCGGAGCTGAATTACATTCCCAACCGCGTGGCACAACAACTGGCGGGCAAACAGTCGTTGCTGATTGGCGTTGCCACCTCCAGTCTGGCCCTGCACGCGCCGTCGCAAATTGTCGCGGCGATTAAATCTCGCGCCGATCAACTGGGTGCCAGCGTGGTGGTGTCGATGGTAGAACGAAGCGGCGTCGAAGCCTGTAAAACGGCGGTGCACAATCTTCTCGCGCAACGCGTCAGTGGGCTGATCATTAACTATCCGCTGGATGACCAGGATGCCATTGCTGTGGAAGCTGCCTGCACTAATGTTCCGGCGTTATTTCTTGATGTCTCTGACCAGACACCCATCAACAGTATTATTTTCTCCCATGAAGACGGTACGCGACTGGGCGTGGAGCATCTGGTCGCATTGGGTCACCAGCAAATCGCGCTGTTAGCGGGCCCATTAAGTTCTGTCTCGGCGCGTCTGCGTCTGGCTGGCTGGCATAAATATCTCACTCGCAATCAAATTCAGCCGATAGCGGAACGGGAAGGCGACTGGAGTGCCATGTCCGGTTTTCAACAAACCATGCAAATGCTGAATGAGGGCATCGTTCCCACTGCGATGCTGGTTGCCAACGATCAGATGGCGCTGGGCGCAATGCGCGCCATTACCGAGTCCGGGCTGCGCGTTGGTGCGGATATCTCGGTAGTGGGATACGACGATACCGAAGACAGCTCATGTTATATCCCGCCGTTAACCACCATCAAACAGGATTTTCGCCTGCTGGGGCAAACCAGCGTGGACCGCTTGCTGCAACTCTCTCAGGGCCAGGCGGTGAAGGGCAATCAGCTGTTGCCCGTCTCACTGGTGAAAAGAAAAACCACCCTGGCGCCCAATACGCAAACCGCCTCTCCCCGCGCGTTGGCCGATTCATTAATGCAGCTGGCACGACAGGTTTCCCGACTGGAAAGCGGGCAGtgagtgcaacaatcagccttgatctggaagaagcaatgaaagctgctgttaagtctccgaatcaggtattgttcctgacaggtgtattcccatccggtaaacgcggatactttgcagttgatctgactcaggaataaattataaattaaggtaagaagattgtaggataagctaatgaaatagaaaaaggatgccgtcacacaacttgtcggcattcttttttgttttattagttgaaaatatagtgaaaaagttgcctaaatatgtatgttaacaaattatttgtcgtaactttgcactccaaatctgtttttaacatatggcactagtgAAACCAGTAACGTTATACGATGTCGCAGAGTATGCCGGTGTCTCTACCGCGACCGTTTCCAACGTGGTGAACCAGGCCAGCCACGTTTCTGCGAAAACGCGGGAAAAAGTGGAAGCGGCGATGGCGGAGCTCAATTACATTCCCAACCGCGTGGCACAACAACTGGCGGGCAAAGCGTCGCATACCATTGGCATGTTGATCACTGCCAGTACCAATCCTTTCTATTCAGAACTGGTGCGTGGCGTTGAACGCAGCTGCTTCGAACGCGGTTATAGTCTCGatCTTTGCAATACCGAAGGCGATGAACAGCGGATGAATCGCAATCTGGAAACGCTGATGCAAAAACGCGTTGATGGCTTGCTGTTACTGTGCACCGAAACGCATCAACCTTCGCGTGAAATCATGCAACGTTATCCGACAGTGCCTACTGTGATGATGGACTGGGCTCCGTTCGATGGCGACAGCGATCTTATTCAGGATAACTCGTTGCTGGGCGGAGACTTAGCAACGCAATATCTGATCGATAAAGGTCATACCCGTATCGCCTGTATTACCGGCCCGCTGGATAAAACTCCGGCGCGCCTGCGGTTGGAAGGTTATCGGGCGGCGATGAAACGTGCGGGTCTCAACATTCCTGATGGCTATGAAGTCACTGGTGATTTTGAATTTAACGGCGGGTTTGACGCTATGCGCCAACTGCTATCACATCCGCTGCGTCCTCAGGCCGTCTTTACCGGAAATGACGCTATGGCTGTTGGCGTTTACCAGGCGTTATATCAGGCAGAGTTACAGGTTCCGCAGGATATCGCGGTGATTGGCTATGACGATATCGAACTGGCAAGCTTTATGACGCCACCATTAACCACTATCCACCAACCGAAAGATGAACTGGGGGAGCTGGCGATTGATGTACTCATCCATCGGATAACCCAGCCGACCCTTCAGCAACAACGATTACAACTTACTCCGATTCTGATGGAACGCGGTTCGGCTTAGCTGGTGAAAAGAAAAACCACCCTGGCGCCCAATACGCAAACCGCCTCTCCCCGCGCGTTGGCCGATTCATTAATGCAGCTGGCACGACAGGTTTCCCGACTGGAAAGCGGGCAGTGAgctttcctcggtaccaaattccagaaaagaggcctcccgaaaggggggccttttttcgttttggtcctacttgtgcctgttctatttccATCCcggttcctggccttttgctggccttttgctcacatgttctttcctgcgttatcccctgattctgtggataaccgtattaccgcctttgagtgagctgatacCCGAtacttgtgcctgttctatttccgaaccgaccgcttgtatgaatccatcaaaattcgttttctctatgttggattccttgttgctcatattgtgatgataatttctacaaatatagtcattggtaactatctatgaaactgtttgatacttttatagttgattaaacttgttcatggcatttgccttaatatcatccgctatgtcaatgtagggtttcatagctttgtagtcgctgtgtcccgtccatttcatgaccacctgtgccgggattccgagagccagcgcattgcagatgaatgtccttcttcctgcatgggtactgagcaaagcgtatttgggtgtgacttcatcaatacgttcatttcccttgtagtaggtttcccgtacaggctcgttgatttctgccagttcgcccagctctttcaggtaatcgttcatcttctggttgctgatgacgggcagagccatgtaattctcgaaatggatgtccttgtatttgtccagtatggctttgctgtatttgttcagttcaatcgtcaggctgtcggcagtcttgactgtggttatttcgatgtggtcggacttcacatcgcttcttttcagattgcgaacatccgaataccgcaaactcgtaaagcagcagaacaggaaaacatcacgcacacgttccaggtattgcttatccttgggtatctggtagtctttcagcttgttcagttcatcccaagtcaggaagattacttttttcgaggtggttttcagtttcggtttgaacgtatcgtatgcaatgttctgatgatgtcctttcctgaagctccagcgcaggaaccatttgaggaatcccatttgcttgccgatggtgctgtttctcatatccttggtgtcacgcaggaagttgacgtattcgttcaatccaaactcgttgaaatagttgaacgttgcatcctccttgaactctttgaggtggttcctcactgctgcaaatttttcataggtggatgccgtccagttattctggttaccgcactcttttacaaactcatcgaacacctcccaaaagctgacaggggcttcttccggctgttcttcactggtgtctttcattctcatgttgaaagcttccttcaactgttgggtcgttggcatgacctcctgcacctcaaattccttgaaaatattctggatttcggcatagtatttcagcaagtccgtattgatttcggctgcactttgctttagcttgttggtacatccgttctttacccgctgcttatctacatcccatttggctacgtcaatccggtagcccgttgtaaactcgatacgttggctggcaaagatgacacgcatacggatgggtacgttctctacgattggcacaccgttctttttccggctctccaatgcaaaaatgatgttgcgcttgatattcataattgggtgcgtttgaaattctacacccaaatatacacccaattattgagatagcaaaagacatttagaaacatttacttttactctatattgtaatttacacttgattatcagtcgtttgcagtTttatgatattctgtgaaagtataagttcgagagcctgtctctccgcaaaaaacgctgaaaatcagcagattgcaaaacaaacaccctgttttacacccaagaatgtaaagtcggctgtttttgttttatttaagataatacaaccactacataataaaagagtagcgatattaaaagaatccgatgagaaaagactaatatttatctatccattcagtttgatttttcaggactttacatcgtcctgaaagtatttgttggtaccggtaccgaggacgcgtaaacatttacagttgcatgtggcctattgtttttagccgttaaatattttataactattaaatagcgatacaaattgttcgaaactaatattgtttatatcatatattctcgcatgttttaaagctttattaaattgattttttgtaaacagtttttcgtactctttgttaacccatttcattacaaaagtttcatatttttttctctctttaaatgccatttttgctggctttctttttaatacaattaatgtgctatccactttaggttttggatggaaataatacctaggaatttttgctaatatagaaatatctacctctgccattaacagcaatgctagtgatctgtttgtatctaataacattttagcaaaaccatattccactattaaataacttattgtggctgaactttcaaaaacaatttttcgaattatatttgtgcttatgttgtaaggtatgctgccaaatattttatatggattgtggctaggaaatgtaaatttcagtatatcatcatttactatttgatagttaggataatttaagagcttattacgagttacctcacataatttagaatcaatttctatcgccgttacaaaattacatctctttaccaatccagcagtaaaatgacctttccctgcacctatttcaaagatgttatctttttcatctaaacttatgcaattcattattttttctatgtgatattttgaagtaataaaattttgactatcttttatatttactttgttcattataacctctccttaatttattgcatctcttttcgaatatttatgttttttgagaaaagaacgtactcatggttcatcccgatatgcgtatcggtctgtatatcagcaactttctatgtgtttcaactacaatagtcatctattctcatctttctgagtccaccccctgcaaagcccctctttacgacataaaaattcggtcggaaaaggtatgcaaaagatgtttctctctttaagagaaactcttcgggatgcaaaaatatgaaaataactccaattcaccaaattatatagcgacttttttacaaaatgctaaaatttgttgatttccgtcaagcaattgttgagcaaaaatgtcttttacgataaaatgatacctcaatatcaactgtttagcaaaacgatatttctcttaaagagagaaacacctttttgttcaccaatccccgacttttaatcccgcggccatgattgaaaaaggaagagtatgagtattcaacatttccgtgtcgcccttattcccttttttgcggcattttgccttcctgtttttgctcacccagaaacgctggtgaaagtaaaagatgctgaagatcagttgggtgcacgagtgggttacatcgaactggatctcaacagcggtaagatccttgagagttttcgccccgaagaacgttttccaatgatgagcacttttaaagttctgctatgtggcgcggtattatcccgtattgacgccgggcaagagcaactcggtcgccgcatacactattctcagaatgacttggttgagtactcaccagtcacagaaaagcatcttacggatggcatgacagtaagagaattatgcagtgctgccataaccatgagtgataacactgcggccaacttacttctgacaacgatcggaggaccgaaggagctaaccgcttttttgcacaacatgggggatcatgtaactcgccttgatcgttgggaaccggagctgaatgaagccataccaaacgacgagcgtgacaccacgatgcctgtagcaatggcaacaacgttgcgcaaactattaactggcgaactacttactctagcttcccggcaacaattaatagactggatggaggcggataaagttgcaggaccacttctgcgctcggcccttccggctggctggtttattgctgataaatctggagccggtgagcgtgggtctcgcggtatcattgcagcactggggccagatggtaagccctcccgtatcgtagttatctacacgacggggagtcaggcaactatggatgaacgaaatagacagatcgctgagataggtgcctcactgattaagcattggtaactgtcagaccaagtttactcataacgcgtcaattcgagggggatcaattccgtgataggtgggctgcccttcctggttggcttggtttcatcagccatccgcttgccctcatctgttacgccggcggtagccggccagcctcgcagagcaggattcccgttgagcaccgccaggtgcgaataagggacagtgaagaaggaacacccgctcgcgggtgggcctacttcacctatcctgcccggctgacgccgttggatacaccaaggaaagtctacacgaaccctttggcaaaatcctgtatatcgtgcgaaaaaggatggatataccgaaaaaatcgctataatgaccccgaagcagggttatgcagcggaaaacggaattgatccggccacgatgcgtccggcgtagaggatctgaagatcagcagttcaacctgttgatagtacgtactaagctctcatgtttcacgtactaagctctcatgtttaacgtactaagctctcatgtttaacgaactaaaccctcatggctaacgtactaagctctcatggctaacgtactaagctctcatgtttcacgtactaagctctcatgtttgaacaataaaattaatataaatcagcaacttaaatagcctctaaggttttaagttttataagaaaaaaaagaatatataaggcttttaaagcttttaag

>pBH209

gtttaacggttgtggacaacaagccagggatgtaacgcactgagaagcccttagagcctctcaaagcaattttgagtgacacaggaacacttaacggctgacatgggaattcccctccaccgcggtggCCCTgtacaaagaaaattcgacaaactgttatttttctatctatttatttgAATTaggAGCGCTcctAATTtacctttgtcggcAATTaggAGCGCTcctAATTaaataaagatattctcgtcaaacaaatataaataatataaacatggtttttactctggaagattttgttggcgattggcgtcagaccgcgggttataatttggatcaagtcctggaacagggtggcgtaagctctctgttccagaacctgggtgtgagcgtgacgccgattcagcgcatcgttctgtccggcgagaacggtctgaaaattgatattcatgtgatcatcccgtacgaaggcctgagcggtgaccaaatgggtcaaatcgagaaaatctttaaagtcgtctacccagttgacgatcaccacttcaaggttatcttgcattacggtacgctggtgattgatggtgtgaccccgaatatgattgactatttcggccgtccgtatgaaggcattgccgtttttgacggtaaaaagatcaccgtcaccggtaccctgtggaatggcaataagattattgacgagcgtctgattaacccggacggcagcctgctgttccgcgtgaccatcaacggtgtcacgggttggcgtctgtgcgagcgcatcctggcataatgaactgcacttgctttgataattaatgataaacaatctaaaagcactctaatcgttatcggagtgcttttagattactaatcaaattgcttctactaattgcctatcttccagtgatggaacagcatttgAACGcattggctgcaacaatcagccttgatctggaagaagcaatgaaagctgctgttaagtctccgaatcaggtattgttcctgacaggtgtattcccatccggtaaacgcggatactttgcagttgatctgactcaggaataaattataaattaaggtaagaagattgtaggataagctaatgaaatagaaaaaggatgccgtcacacaacttgtcggcattcttttttgttttattagttgaaaatatagtgaaaaagttgcctaaatatgtatgttaacaaattatttgtcgtaactttgcactccaaatctgtttttaacatatggcactagtgAAACCAGTAACGTTATACGATGTCGCAGAGTATGCCGGTGTCTCTAAAAGCACCGTTTCCCTGGTGGTGAACCAGGCCAGCCACGTTTCTGCGAAAACGCGGGAAAAAGTGGAAGCGGCGATGGCGGAGCTGAATTACATTCCCAACCGCGTGGCACAACAACTGGCGGGCAAACAGTCGTTGCTGATTGGCGTTGCCACCTCCAGTCTGGCCCTGCACGCGCCGTCGCAAATTGTCGCGGCGATTAAATCTCGCGCCGATCAACTGGGTGCCAGCGTGGTGGTGTCGATGGTAGAACGAAGCGGCGTCGAAGCCTGTAAAACGGCGGTGCACAATCTTCTCGCGCAACGCGTCAGTGGGCTGATCATTAACTATCCGCTGGATGACCAGGATGCCATTGCTGTGGAAGCTGCCTGCACTAATGTTCCGGCGTTATTTCTTGATGTCTCTGACCAGACACCCATCAACAGTATTATTTTCTCCCATGAAGACGGTACGCGACTGGGCGTGGAGCATCTGGTCGCATTGGGTCACCAGCAAATCGCGCTGTTAGCGGGCCCATTAAGTTCTGTCTCGGCGCGTCTGCGTCTGGCTGGCTGGCATAAATATCTCACTCGCAATCAAATTCAGCCGATAGCGGAACGGGAAGGCGACTGGAGTGCCATGTCCGGTTTTCAACAAACCATGCAAATGCTGAATGAGGGCATCGTTCCCACTGCGATGCTGGTTGCCAACGATCAGATGGCGCTGGGCGCAATGCGCGCCATTACCGAGTCCGGGCTGCGCGTTGGTGCGGATATCTCGGTAGTGGGATACGACGATACCGAAGACAGCTCATGTTATATCCCGCCGTTAACCACCATCAAACAGGATTTTCGCCTGCTGGGGCAAACCAGCGTGGACCGCTTGCTGCAACTCTCTCAGGGCCAGGCGGTGAAGGGCAATCAGCTGTTGCCCGTCTCACTGGTGAAAAGAAAAACCACCCTGGCGCCCAATACGCAAACCGCCTCTCCCCGCGCGTTGGCCGATTCATTAATGCAGCTGGCACGACAGGTTTCCCGACTGGAAAGCGGGCAGtgagtgcaacaatcagccttgatctggaagaagcaatgaaagctgctgttaagtctccgaatcaggtattgttcctgacaggtgtattcccatccggtaaacgcggatactttgcagttgatctgactcaggaataaattataaattaaggtaagaagattgtaggataagctaatgaaatagaaaaaggatgccgtcacacaacttgtcggcattcttttttgttttattagttgaaaatatagtgaaaaagttgcctaaatatgtatgttaacaaattatttgtcgtaactttgcactccaaatctgtttttaacatatggcactagtgAAACCAGTAACGTTATACGATGTCGCAGAGTATGCCGGTGTCTCTAAAAGCACCGTTTCCCTGGTGGTGAACCAGGCCAGCCACGTTTCTGCGAAAACGCGGGAAAAAGTGGAAGCGGCGATGGCGGAGCTCAATTACATTCCCAACCGCGTGGCACAACAACTGGCGGGCAAAGCGTCGCATACCATTGGCATGTTGATCACTGCCAGTACCAATCCTTTCTATTCAGAACTGGTGCGTGGCGTTGAACGCAGCTGCTTCGAACGCGGTTATAGTCTCGatCTTTGCAATACCGAAGGCGATGAACAGCGGATGAATCGCAATCTGGAAACGCTGATGCAAAAACGCGTTGATGGCTTGCTGTTACTGTGCACCGAAACGCATCAACCTTCGCGTGAAATCATGCAACGTTATCCGACAGTGCCTACTGTGATGATGGACTGGGCTCCGTTCGATGGCGACAGCGATCTTATTCAGGATAACTCGTTGCTGGGCGGAGACTTAGCAACGCAATATCTGATCGATAAAGGTCATACCCGTATCGCCTGTATTACCGGCCCGCTGGATAAAACTCCGGCGCGCCTGCGGTTGGAAGGTTATCGGGCGGCGATGAAACGTGCGGGTCTCAACATTCCTGATGGCTATGAAGTCACTGGTGATTTTGAATTTAACGGCGGGTTTGACGCTATGCGCCAACTGCTATCACATCCGCTGCGTCCTCAGGCCGTCTTTACCGGAAATGACGCTATGGCTGTTGGCGTTTACCAGGCGTTATATCAGGCAGAGTTACAGGTTCCGCAGGATATCGCGGTGATTGGCTATGACGATATCGAACTGGCAAGCTTTATGACGCCACCATTAACCACTATCCACCAACCGAAAGATGAACTGGGGGAGCTGGCGATTGATGTACTCATCCATCGGATAACCCAGCCGACCCTTCAGCAACAACGATTACAACTTACTCCGATTCTGATGGAACGCGGTTCGGCTTAGCTGGTGAAAAGAAAAACCACCCTGGCGCCCAATACGCAAACCGCCTCTCCCCGCGCGTTGGCCGATTCATTAATGCAGCTGGCACGACAGGTTTCCCGACTGGAAAGCGGGCAGTGAgctttcctcggtaccaaattccagaaaagaggcctcccgaaaggggggccttttttcgttttggtcctacttgtgcctgttctatttccATCCcggttcctggccttttgctggccttttgctcacatgttctttcctgcgttatcccctgattctgtggataaccgtattaccgcctttgagtgagctgatacCCGAtacttgtgcctgttctatttccgaaccgaccgcttgtatgaatccatcaaaattcgttttctctatgttggattccttgttgctcatattgtgatgataatttctacaaatatagtcattggtaactatctatgaaactgtttgatacttttatagttgattaaacttgttcatggcatttgccttaatatcatccgctatgtcaatgtagggtttcatagctttgtagtcgctgtgtcccgtccatttcatgaccacctgtgccgggattccgagagccagcgcattgcagatgaatgtccttcttcctgcatgggtactgagcaaagcgtatttgggtgtgacttcatcaatacgttcatttcccttgtagtaggtttcccgtacaggctcgttgatttctgccagttcgcccagctctttcaggtaatcgttcatcttctggttgctgatgacgggcagagccatgtaattctcgaaatggatgtccttgtatttgtccagtatggctttgctgtatttgttcagttcaatcgtcaggctgtcggcagtcttgactgtggttatttcgatgtggtcggacttcacatcgcttcttttcagattgcgaacatccgaataccgcaaactcgtaaagcagcagaacaggaaaacatcacgcacacgttccaggtattgcttatccttgggtatctggtagtctttcagcttgttcagttcatcccaagtcaggaagattacttttttcgaggtggttttcagtttcggtttgaacgtatcgtatgcaatgttctgatgatgtcctttcctgaagctccagcgcaggaaccatttgaggaatcccatttgcttgccgatggtgctgtttctcatatccttggtgtcacgcaggaagttgacgtattcgttcaatccaaactcgttgaaatagttgaacgttgcatcctccttgaactctttgaggtggttcctcactgctgcaaatttttcataggtggatgccgtccagttattctggttaccgcactcttttacaaactcatcgaacacctcccaaaagctgacaggggcttcttccggctgttcttcactggtgtctttcattctcatgttgaaagcttccttcaactgttgggtcgttggcatgacctcctgcacctcaaattccttgaaaatattctggatttcggcatagtatttcagcaagtccgtattgatttcggctgcactttgctttagcttgttggtacatccgttctttacccgctgcttatctacatcccatttggctacgtcaatccggtagcccgttgtaaactcgatacgttggctggcaaagatgacacgcatacggatgggtacgttctctacgattggcacaccgttctttttccggctctccaatgcaaaaatgatgttgcgcttgatattcataattgggtgcgtttgaaattctacacccaaatatacacccaattattgagatagcaaaagacatttagaaacatttacttttactctatattgtaatttacacttgattatcagtcgtttgcagtTttatgatattctgtgaaagtataagttcgagagcctgtctctccgcaaaaaacgctgaaaatcagcagattgcaaaacaaacaccctgttttacacccaagaatgtaaagtcggctgtttttgttttatttaagataatacaaccactacataataaaagagtagcgatattaaaagaatccgatgagaaaagactaatatttatctatccattcagtttgatttttcaggactttacatcgtcctgaaagtatttgttggtaccggtaccgaggacgcgtaaacatttacagttgcatgtggcctattgtttttagccgttaaatattttataactattaaatagcgatacaaattgttcgaaactaatattgtttatatcatatattctcgcatgttttaaagctttattaaattgattttttgtaaacagtttttcgtactctttgttaacccatttcattacaaaagtttcatatttttttctctctttaaatgccatttttgctggctttctttttaatacaattaatgtgctatccactttaggttttggatggaaataatacctaggaatttttgctaatatagaaatatctacctctgccattaacagcaatgctagtgatctgtttgtatctaataacattttagcaaaaccatattccactattaaataacttattgtggctgaactttcaaaaacaatttttcgaattatatttgtgcttatgttgtaaggtatgctgccaaatattttatatggattgtggctaggaaatgtaaatttcagtatatcatcatttactatttgatagttaggataatttaagagcttattacgagttacctcacataatttagaatcaatttctatcgccgttacaaaattacatctctttaccaatccagcagtaaaatgacctttccctgcacctatttcaaagatgttatctttttcatctaaacttatgcaattcattattttttctatgtgatattttgaagtaataaaattttgactatcttttatatttactttgttcattataacctctccttaatttattgcatctcttttcgaatatttatgttttttgagaaaagaacgtactcatggttcatcccgatatgcgtatcggtctgtatatcagcaactttctatgtgtttcaactacaatagtcatctattctcatctttctgagtccaccccctgcaaagcccctctttacgacataaaaattcggtcggaaaaggtatgcaaaagatgtttctctctttaagagaaactcttcgggatgcaaaaatatgaaaataactccaattcaccaaattatatagcgacttttttacaaaatgctaaaatttgttgatttccgtcaagcaattgttgagcaaaaatgtcttttacgataaaatgatacctcaatatcaactgtttagcaaaacgatatttctcttaaagagagaaacacctttttgttcaccaatccccgacttttaatcccgcggccatgattgaaaaaggaagagtatgagtattcaacatttccgtgtcgcccttattcccttttttgcggcattttgccttcctgtttttgctcacccagaaacgctggtgaaagtaaaagatgctgaagatcagttgggtgcacgagtgggttacatcgaactggatctcaacagcggtaagatccttgagagttttcgccccgaagaacgttttccaatgatgagcacttttaaagttctgctatgtggcgcggtattatcccgtattgacgccgggcaagagcaactcggtcgccgcatacactattctcagaatgacttggttgagtactcaccagtcacagaaaagcatcttacggatggcatgacagtaagagaattatgcagtgctgccataaccatgagtgataacactgcggccaacttacttctgacaacgatcggaggaccgaaggagctaaccgcttttttgcacaacatgggggatcatgtaactcgccttgatcgttgggaaccggagctgaatgaagccataccaaacgacgagcgtgacaccacgatgcctgtagcaatggcaacaacgttgcgcaaactattaactggcgaactacttactctagcttcccggcaacaattaatagactggatggaggcggataaagttgcaggaccacttctgcgctcggcccttccggctggctggtttattgctgataaatctggagccggtgagcgtgggtctcgcggtatcattgcagcactggggccagatggtaagccctcccgtatcgtagttatctacacgacggggagtcaggcaactatggatgaacgaaatagacagatcgctgagataggtgcctcactgattaagcattggtaactgtcagaccaagtttactcataacgcgtcaattcgagggggatcaattccgtgataggtgggctgcccttcctggttggcttggtttcatcagccatccgcttgccctcatctgttacgccggcggtagccggccagcctcgcagagcaggattcccgttgagcaccgccaggtgcgaataagggacagtgaagaaggaacacccgctcgcgggtgggcctacttcacctatcctgcccggctgacgccgttggatacaccaaggaaagtctacacgaaccctttggcaaaatcctgtatatcgtgcgaaaaaggatggatataccgaaaaaatcgctataatgaccccgaagcagggttatgcagcggaaaacggaattgatccggccacgatgcgtccggcgtagaggatctgaagatcagcagttcaacctgttgatagtacgtactaagctctcatgtttcacgtactaagctctcatgtttaacgtactaagctctcatgtttaacgaactaaaccctcatggctaacgtactaagctctcatggctaacgtactaagctctcatgtttcacgtactaagctctcatgtttgaacaataaaattaatataaatcagcaacttaaatagcctctaaggttttaagttttataagaaaaaaaagaatatataaggcttttaaagcttttaag

>pBH210

gtttaacggttgtggacaacaagccagggatgtaacgcactgagaagcccttagagcctctcaaagcaattttgagtgacacaggaacacttaacggctgacatgggaattcccctccaccgcggtggCCCTgtacaaagaaaattcgacaaactgttatttttctatctatttatttgaattgtgagcggataacaattacctttgtcggcaattgtgagcggataacaattaaataaagatattctcgtcaaacaaatataaataatataaacatggtttttactctggaagattttgttggcgattggcgtcagaccgcgggttataatttggatcaagtcctggaacagggtggcgtaagctctctgttccagaacctgggtgtgagcgtgacgccgattcagcgcatcgttctgtccggcgagaacggtctgaaaattgatattcatgtgatcatcccgtacgaaggcctgagcggtgaccaaatgggtcaaatcgagaaaatctttaaagtcgtctacccagttgacgatcaccacttcaaggttatcttgcattacggtacgctggtgattgatggtgtgaccccgaatatgattgactatttcggccgtccgtatgaaggcattgccgtttttgacggtaaaaagatcaccgtcaccggtaccctgtggaatggcaataagattattgacgagcgtctgattaacccggacggcagcctgctgttccgcgtgaccatcaacggtgtcacgggttggcgtctgtgcgagcgcatcctggcataatgaactgcacttgctttgataattaatgataaacaatctaaaagcactctaatcgttatcggagtgcttttagattactaatcaaattgcttctactaattgcctatcttccagtgatggaacagcatttgAACGcattggctgcaacaatcagccttgatctggaagaagcaatgaaagctgctgttaagtctccgaatcaggtattgttcctgacaggtgtattcccatccggtaaacgcggatactttgcagttgatctgactcaggaataaattataaattaaggtaagaagattgtaggataagctaatgaaatagaaaaaggatgccgtcacacaacttgtcggcattcttttttgttttattagttgaaaatatagtgaaaaagttgcctaaatatgtatgttaacaaattatttgtcgtaactttgcactccaaatctgtttttaacatatggcactagtgAAACCAGTAACGTTATACGATGTCGCAGAGTATGCCGGTGTCTCTTATCAGACCGTTTCCCGCGTGGTGAACCAGGCCAGCCACGTTTCTGCGAAAACGCGGGAAAAAGTGGAAGCGGCGATGGCGGAGCTGAATTACATTCCCAACCGCGTGGCACAACAACTGGCGGGCAAACAGTCGTTGCTGATTGGCGTTGCCACCTCCAGTCTGGCCCTGCACGCGCTGTCGCAAATTGTCGCGGCGATTAAATCTCGCGCCTATCAACTGGGTGCCAGCGTGTTCGTGTCGATGGTAGAACGAAGCGGCATCGAAGCCTGTAAAACGGCGGTGCACAATCTTCTCGCGCAACGCGTCAGTGGGCTGATCATTAACTATCCGCTGGATAACCAGGATGCCATTGCTGTGGAAGCTGCCTGCACTAATGTTCCGGCGTTATTTCTTGATGTCTCTGACCAGACACCCATCAACAGTATTATTTTCTCCCATGAAGACGGTACGCGACTGGGCGTGGAGCATCTGGTCGCATTGGGTCACCAGCAAATCGCGCTGTTAGCGGGCCCATTAAGTTCTGTCTCGGCGCGTCTGCGTCTGGCTGGCTGGCATAAATATCTCACTCGCAATCAAATTCAGCCGATAGCGGAACGGGAAGGCGACTGGAGTGCCATGTCCGGTTTTCAACAAACCATGCAAATGCTGAATGAGGGCATCGTTCCCACTGCGATGCTGGTTGCCAACGATCAGATGGCGCTGGGCGCAATGCGCGCCATTACCGAGACCGGGCTGCGCGTTGGTGCGGATATCTCGGTAGTGGGATACGACGATACCGAAGACAGCTCATGTTATATCCCGCCGTTAACCACCATCAAACAGGATTTTCGCCTGCTGGGGCAAACCAGCGTGGACCGCTTGCTGCAACTCTCTCAGGGCCAGGCGGTGAAGGGCAATCAGCTGTTGCCCGTCTCACTGGTGAAAAGAAAAACCACCCTGGCGCCCAATACGCAAACCGCCTCTCCCCGCGCGTTGGCCGATTCATTAATGCAGCTGGCACGACAGGTTTCCCGACTGGAAAGCGGGCAGtgagtgcaacaatcagccttgatctggaagaagcaatgaaagctgctgttaagtctccgaatcaggtattgttcctgacaggtgtattcccatccggtaaacgcggatactttgcagttgatctgactcaggaataaattataaattaaggtaagaagattgtaggataagctaatgaaatagaaaaaggatgccgtcacacaacttgtcggcattcttttttgttttattagttgaaaatatagtgaaaaagttgcctaaatatgtatgttaacaaattatttgtcgtaactttgcactccaaatctgtttttaacatatggcactaGTGAAACCAGTAACGTTATACGATGTCGCAGAGTATGCCGGTGTCTCTTATCAGACCGTTTCCCGCGTGGTGAACCAGGCCAGCCACGTTTCTGCGAAAACGCGGGAAAAAGTGGAAGCGGCGATGGCGGAGCTCAATTACATTCCCAACCGCGTGGCACAACAACTGGCGGGCAAAGCGTCGCATACCATTGGCATGTTGATCACTGCCAGTACCAATCCTTTCTATTCAGAACTGGTGCGTGGCGTTGAACGCAGCTGCTTCGAACGCGGTTATAGTCTCGtcCTTTGCAATACCGAAGGCGATGAACAGCGGATGAATCGCAATCTGGAAACGCTGATGCAAAAACGCGTTGATGGCTTGCTGTTACTGTGCACCGAAACGCATCAACCTTCGCGTGAAATCATGCAACGTTATCCGACAGTGCCTACTGTGATGATGGACTGGGCTCCGTTCGATGGCGACAGCGATCTTATTCAGGATAACTCGTTGCTGGGCGGAGACTTAGCAACGCAATATCTGATCGATAAAGGTCATACCCGTATCGCCTGTATTACCGGCCCGCTGGATAAAACTCCGGCGCGCCTGCGGTTGGAAGGTTATCGGGCGGCGATGAAACGTGCGGGTCTCAACATTCCTGATGGCTATGAAGTCACTGGTGATTTTGAATTTAACGGCGGGTTTGACGCTATGCGCCAACTGCTATCACATCCGCTGCGTCCTCAGGCCGTCTTTACCGGAAATGACGCTATGGCTGTTGGCGTTTACCAGGCGTTATATCAGGCAGAGTTACAGGTTCCGCAGGATATCGCGGTGATTGGCTATGACGATATCGAACTGGCAAGCTTTATGACGCCACCATTAACCACTATCCACCAACCGAAAGATGAACTGGGGGAGCTGGCGATTGATGTACTCATCCATCGGATAACCCAGCCGACCCTTCAGCAACAACGATTACAACTTACTCCGATTCTGATGGAACGCGGTTCGGCTTAGCTGGTGAAAAGAAAAACCACCCTGGCGCCCAATACGCAAACCGCCTCTCCCCGCGCGTTGGCCGATTCATTAATGCAGCTGGCACGACAGGTTTCCCGACTGGAAAGCGGGCAGTGAgctttcctcggtaccaaattccagaaaagaggcctcccgaaaggggggccttttttcgttttggtcctacttgtgcctgttctatttccATCCcggttcctggccttttgctggccttttgctcacatgttctttcctgcgttatcccctgattctgtggataaccgtattaccgcctttgagtgagctgatacCCGAtacttgtgcctgttctatttccgaaccgaccgcttgtatgaatccatcaaaattcgttttctctatgttggattccttgttgctcatattgtgatgataatttctacaaatatagtcattggtaactatctatgaaactgtttgatacttttatagttgattaaacttgttcatggcatttgccttaatatcatccgctatgtcaatgtagggtttcatagctttgtagtcgctgtgtcccgtccatttcatgaccacctgtgccgggattccgagagccagcgcattgcagatgaatgtccttcttcctgcatgggtactgagcaaagcgtatttgggtgtgacttcatcaatacgttcatttcccttgtagtaggtttcccgtacaggctcgttgatttctgccagttcgcccagctctttcaggtaatcgttcatcttctggttgctgatgacgggcagagccatgtaattctcgaaatggatgtccttgtatttgtccagtatggctttgctgtatttgttcagttcaatcgtcaggctgtcggcagtcttgactgtggttatttcgatgtggtcggacttcacatcgcttcttttcagattgcgaacatccgaataccgcaaactcgtaaagcagcagaacaggaaaacatcacgcacacgttccaggtattgcttatccttgggtatctggtagtctttcagcttgttcagttcatcccaagtcaggaagattacttttttcgaggtggttttcagtttcggtttgaacgtatcgtatgcaatgttctgatgatgtcctttcctgaagctccagcgcaggaaccatttgaggaatcccatttgcttgccgatggtgctgtttctcatatccttggtgtcacgcaggaagttgacgtattcgttcaatccaaactcgttgaaatagttgaacgttgcatcctccttgaactctttgaggtggttcctcactgctgcaaatttttcataggtggatgccgtccagttattctggttaccgcactcttttacaaactcatcgaacacctcccaaaagctgacaggggcttcttccggctgttcttcactggtgtctttcattctcatgttgaaagcttccttcaactgttgggtcgttggcatgacctcctgcacctcaaattccttgaaaatattctggatttcggcatagtatttcagcaagtccgtattgatttcggctgcactttgctttagcttgttggtacatccgttctttacccgctgcttatctacatcccatttggctacgtcaatccggtagcccgttgtaaactcgatacgttggctggcaaagatgacacgcatacggatgggtacgttctctacgattggcacaccgttctttttccggctctccaatgcaaaaatgatgttgcgcttgatattcataattgggtgcgtttgaaattctacacccaaatatacacccaattattgagatagcaaaagacatttagaaacatttacttttactctatattgtaatttacacttgattatcagtcgtttgcagtTttatgatattctgtgaaagtataagttcgagagcctgtctctccgcaaaaaacgctgaaaatcagcagattgcaaaacaaacaccctgttttacacccaagaatgtaaagtcggctgtttttgttttatttaagataatacaaccactacataataaaagagtagcgatattaaaagaatccgatgagaaaagactaatatttatctatccattcagtttgatttttcaggactttacatcgtcctgaaagtatttgttggtaccggtaccgaggacgcgtaaacatttacagttgcatgtggcctattgtttttagccgttaaatattttataactattaaatagcgatacaaattgttcgaaactaatattgtttatatcatatattctcgcatgttttaaagctttattaaattgattttttgtaaacagtttttcgtactctttgttaacccatttcattacaaaagtttcatatttttttctctctttaaatgccatttttgctggctttctttttaatacaattaatgtgctatccactttaggttttggatggaaataatacctaggaatttttgctaatatagaaatatctacctctgccattaacagcaatgctagtgatctgtttgtatctaataacattttagcaaaaccatattccactattaaataacttattgtggctgaactttcaaaaacaatttttcgaattatatttgtgcttatgttgtaaggtatgctgccaaatattttatatggattgtggctaggaaatgtaaatttcagtatatcatcatttactatttgatagttaggataatttaagagcttattacgagttacctcacataatttagaatcaatttctatcgccgttacaaaattacatctctttaccaatccagcagtaaaatgacctttccctgcacctatttcaaagatgttatctttttcatctaaacttatgcaattcattattttttctatgtgatattttgaagtaataaaattttgactatcttttatatttactttgttcattataacctctccttaatttattgcatctcttttcgaatatttatgttttttgagaaaagaacgtactcatggttcatcccgatatgcgtatcggtctgtatatcagcaactttctatgtgtttcaactacaatagtcatctattctcatctttctgagtccaccccctgcaaagcccctctttacgacataaaaattcggtcggaaaaggtatgcaaaagatgtttctctctttaagagaaactcttcgggatgcaaaaatatgaaaataactccaattcaccaaattatatagcgacttttttacaaaatgctaaaatttgttgatttccgtcaagcaattgttgagcaaaaatgtcttttacgataaaatgatacctcaatatcaactgtttagcaaaacgatatttctcttaaagagagaaacacctttttgttcaccaatccccgacttttaatcccgcggccatgattgaaaaaggaagagtatgagtattcaacatttccgtgtcgcccttattcccttttttgcggcattttgccttcctgtttttgctcacccagaaacgctggtgaaagtaaaagatgctgaagatcagttgggtgcacgagtgggttacatcgaactggatctcaacagcggtaagatccttgagagttttcgccccgaagaacgttttccaatgatgagcacttttaaagttctgctatgtggcgcggtattatcccgtattgacgccgggcaagagcaactcggtcgccgcatacactattctcagaatgacttggttgagtactcaccagtcacagaaaagcatcttacggatggcatgacagtaagagaattatgcagtgctgccataaccatgagtgataacactgcggccaacttacttctgacaacgatcggaggaccgaaggagctaaccgcttttttgcacaacatgggggatcatgtaactcgccttgatcgttgggaaccggagctgaatgaagccataccaaacgacgagcgtgacaccacgatgcctgtagcaatggcaacaacgttgcgcaaactattaactggcgaactacttactctagcttcccggcaacaattaatagactggatggaggcggataaagttgcaggaccacttctgcgctcggcccttccggctggctggtttattgctgataaatctggagccggtgagcgtgggtctcgcggtatcattgcagcactggggccagatggtaagccctcccgtatcgtagttatctacacgacggggagtcaggcaactatggatgaacgaaatagacagatcgctgagataggtgcctcactgattaagcattggtaactgtcagaccaagtttactcataacgcgtcaattcgagggggatcaattccgtgataggtgggctgcccttcctggttggcttggtttcatcagccatccgcttgccctcatctgttacgccggcggtagccggccagcctcgcagagcaggattcccgttgagcaccgccaggtgcgaataagggacagtgaagaaggaacacccgctcgcgggtgggcctacttcacctatcctgcccggctgacgccgttggatacaccaaggaaagtctacacgaaccctttggcaaaatcctgtatatcgtgcgaaaaaggatggatataccgaaaaaatcgctataatgaccccgaagcagggttatgcagcggaaaacggaattgatccggccacgatgcgtccggcgtagaggatctgaagatcagcagttcaacctgttgatagtacgtactaagctctcatgtttcacgtactaagctctcatgtttaacgtactaagctctcatgtttaacgaactaaaccctcatggctaacgtactaagctctcatggctaacgtactaagctctcatgtttcacgtactaagctctcatgtttgaacaataaaattaatataaatcagcaacttaaatagcctctaaggttttaagttttataagaaaaaaaagaatatataaggcttttaaagcttttaag

>pBH211

gtttaacggttgtggacaacaagccagggatgtaacgcactgagaagcccttagagcctctcaaagcaattttgagtgacacaggaacacttaacggctgacatgggaattcccctccaccgcggtggCCCTgtacaaagaaaattcgacaaactgttatttttctatctatttatttgaattTTAAGCGCTTAAaatttacctttgtcggcaattTTAAGCGCTTAAaattaaataaagatattctcgtcaaacaaatataaataatataaacatggtttttactctggaagattttgttggcgattggcgtcagaccgcgggttataatttggatcaagtcctggaacagggtggcgtaagctctctgttccagaacctgggtgtgagcgtgacgccgattcagcgcatcgttctgtccggcgagaacggtctgaaaattgatattcatgtgatcatcccgtacgaaggcctgagcggtgaccaaatgggtcaaatcgagaaaatctttaaagtcgtctacccagttgacgatcaccacttcaaggttatcttgcattacggtacgctggtgattgatggtgtgaccccgaatatgattgactatttcggccgtccgtatgaaggcattgccgtttttgacggtaaaaagatcaccgtcaccggtaccctgtggaatggcaataagattattgacgagcgtctgattaacccggacggcagcctgctgttccgcgtgaccatcaacggtgtcacgggttggcgtctgtgcgagcgcatcctggcataatgaactgcacttgctttgataattaatgataaacaatctaaaagcactctaatcgttatcggagtgcttttagattactaatcaaattgcttctactaattgcctatcttccagtgatggaacagcatttgAACGcattggctgcaacaatcagccttgatctggaagaagcaatgaaagctgctgttaagtctccgaatcaggtattgttcctgacaggtgtattcccatccggtaaacgcggatactttgcagttgatctgactcaggaataaattataaattaaggtaagaagattgtaggataagctaatgaaatagaaaaaggatgccgtcacacaacttgtcggcattcttttttgttttattagttgaaaatatagtgaaaaagttgcctaaatatgtatgttaacaaattatttgtcgtaactttgcactccaaatctgtttttaacatatggcactagtgAAACCAGTAACGTTATACGATGTCGCAGAGTATGCCGGTGTCTCTACCGCGACCGTTTCCAACGTGGTGAACCAGGCCAGCCACGTTTCTGCGAAAACGCGGGAAAAAGTGGAAGCGGCGATGGCGGAGCTGAATTACATTCCCAACCGCGTGGCACAACAACTGGCGGGCAAACAGTCGTTGCTGATTGGCGTTGCCACCTCCAGTCTGGCCCTGCACGCGCTGTCGCAAATTGTCGCGGCGATTAAATCTCGCGCCTATCAACTGGGTGCCAGCGTGTTCGTGTCGATGGTAGAACGAAGCGGCATCGAAGCCTGTAAAACGGCGGTGCACAATCTTCTCGCGCAACGCGTCAGTGGGCTGATCATTAACTATCCGCTGGATAACCAGGATGCCATTGCTGTGGAAGCTGCCTGCACTAATGTTCCGGCGTTATTTCTTGATGTCTCTGACCAGACACCCATCAACAGTATTATTTTCTCCCATGAAGACGGTACGCGACTGGGCGTGGAGCATCTGGTCGCATTGGGTCACCAGCAAATCGCGCTGTTAGCGGGCCCATTAAGTTCTGTCTCGGCGCGTCTGCGTCTGGCTGGCTGGCATAAATATCTCACTCGCAATCAAATTCAGCCGATAGCGGAACGGGAAGGCGACTGGAGTGCCATGTCCGGTTTTCAACAAACCATGCAAATGCTGAATGAGGGCATCGTTCCCACTGCGATGCTGGTTGCCAACGATCAGATGGCGCTGGGCGCAATGCGCGCCATTACCGAGACCGGGCTGCGCGTTGGTGCGGATATCTCGGTAGTGGGATACGACGATACCGAAGACAGCTCATGTTATATCCCGCCGTTAACCACCATCAAACAGGATTTTCGCCTGCTGGGGCAAACCAGCGTGGACCGCTTGCTGCAACTCTCTCAGGGCCAGGCGGTGAAGGGCAATCAGCTGTTGCCCGTCTCACTGGTGAAAAGAAAAACCACCCTGGCGCCCAATACGCAAACCGCCTCTCCCCGCGCGTTGGCCGATTCATTAATGCAGCTGGCACGACAGGTTTCCCGACTGGAAAGCGGGCAGtgagtgcaacaatcagccttgatctggaagaagcaatgaaagctgctgttaagtctccgaatcaggtattgttcctgacaggtgtattcccatccggtaaacgcggatactttgcagttgatctgactcaggaataaattataaattaaggtaagaagattgtaggataagctaatgaaatagaaaaaggatgccgtcacacaacttgtcggcattcttttttgttttattagttgaaaatatagtgaaaaagttgcctaaatatgtatgttaacaaattatttgtcgtaactttgcactccaaatctgtttttaacatatggcactagtgAAACCAGTAACGTTATACGATGTCGCAGAGTATGCCGGTGTCTCTACCGCGACCGTTTCCAACGTGGTGAACCAGGCCAGCCACGTTTCTGCGAAAACGCGGGAAAAAGTGGAAGCGGCGATGGCGGAGCTCAATTACATTCCCAACCGCGTGGCACAACAACTGGCGGGCAAAGCGTCGCATACCATTGGCATGTTGATCACTGCCAGTACCAATCCTTTCTATTCAGAACTGGTGCGTGGCGTTGAACGCAGCTGCTTCGAACGCGGTTATAGTCTCGtcCTTTGCAATACCGAAGGCGATGAACAGCGGATGAATCGCAATCTGGAAACGCTGATGCAAAAACGCGTTGATGGCTTGCTGTTACTGTGCACCGAAACGCATCAACCTTCGCGTGAAATCATGCAACGTTATCCGACAGTGCCTACTGTGATGATGGACTGGGCTCCGTTCGATGGCGACAGCGATCTTATTCAGGATAACTCGTTGCTGGGCGGAGACTTAGCAACGCAATATCTGATCGATAAAGGTCATACCCGTATCGCCTGTATTACCGGCCCGCTGGATAAAACTCCGGCGCGCCTGCGGTTGGAAGGTTATCGGGCGGCGATGAAACGTGCGGGTCTCAACATTCCTGATGGCTATGAAGTCACTGGTGATTTTGAATTTAACGGCGGGTTTGACGCTATGCGCCAACTGCTATCACATCCGCTGCGTCCTCAGGCCGTCTTTACCGGAAATGACGCTATGGCTGTTGGCGTTTACCAGGCGTTATATCAGGCAGAGTTACAGGTTCCGCAGGATATCGCGGTGATTGGCTATGACGATATCGAACTGGCAAGCTTTATGACGCCACCATTAACCACTATCCACCAACCGAAAGATGAACTGGGGGAGCTGGCGATTGATGTACTCATCCATCGGATAACCCAGCCGACCCTTCAGCAACAACGATTACAACTTACTCCGATTCTGATGGAACGCGGTTCGGCTTAGCTGGTGAAAAGAAAAACCACCCTGGCGCCCAATACGCAAACCGCCTCTCCCCGCGCGTTGGCCGATTCATTAATGCAGCTGGCACGACAGGTTTCCCGACTGGAAAGCGGGCAGTGAgctttcctcggtaccaaattccagaaaagaggcctcccgaaaggggggccttttttcgttttggtcctacttgtgcctgttctatttccATCCcggttcctggccttttgctggccttttgctcacatgttctttcctgcgttatcccctgattctgtggataaccgtattaccgcctttgagtgagctgatacCCGAtacttgtgcctgttctatttccgaaccgaccgcttgtatgaatccatcaaaattcgttttctctatgttggattccttgttgctcatattgtgatgataatttctacaaatatagtcattggtaactatctatgaaactgtttgatacttttatagttgattaaacttgttcatggcatttgccttaatatcatccgctatgtcaatgtagggtttcatagctttgtagtcgctgtgtcccgtccatttcatgaccacctgtgccgggattccgagagccagcgcattgcagatgaatgtccttcttcctgcatgggtactgagcaaagcgtatttgggtgtgacttcatcaatacgttcatttcccttgtagtaggtttcccgtacaggctcgttgatttctgccagttcgcccagctctttcaggtaatcgttcatcttctggttgctgatgacgggcagagccatgtaattctcgaaatggatgtccttgtatttgtccagtatggctttgctgtatttgttcagttcaatcgtcaggctgtcggcagtcttgactgtggttatttcgatgtggtcggacttcacatcgcttcttttcagattgcgaacatccgaataccgcaaactcgtaaagcagcagaacaggaaaacatcacgcacacgttccaggtattgcttatccttgggtatctggtagtctttcagcttgttcagttcatcccaagtcaggaagattacttttttcgaggtggttttcagtttcggtttgaacgtatcgtatgcaatgttctgatgatgtcctttcctgaagctccagcgcaggaaccatttgaggaatcccatttgcttgccgatggtgctgtttctcatatccttggtgtcacgcaggaagttgacgtattcgttcaatccaaactcgttgaaatagttgaacgttgcatcctccttgaactctttgaggtggttcctcactgctgcaaatttttcataggtggatgccgtccagttattctggttaccgcactcttttacaaactcatcgaacacctcccaaaagctgacaggggcttcttccggctgttcttcactggtgtctttcattctcatgttgaaagcttccttcaactgttgggtcgttggcatgacctcctgcacctcaaattccttgaaaatattctggatttcggcatagtatttcagcaagtccgtattgatttcggctgcactttgctttagcttgttggtacatccgttctttacccgctgcttatctacatcccatttggctacgtcaatccggtagcccgttgtaaactcgatacgttggctggcaaagatgacacgcatacggatgggtacgttctctacgattggcacaccgttctttttccggctctccaatgcaaaaatgatgttgcgcttgatattcataattgggtgcgtttgaaattctacacccaaatatacacccaattattgagatagcaaaagacatttagaaacatttacttttactctatattgtaatttacacttgattatcagtcgtttgcagtTttatgatattctgtgaaagtataagttcgagagcctgtctctccgcaaaaaacgctgaaaatcagcagattgcaaaacaaacaccctgttttacacccaagaatgtaaagtcggctgtttttgttttatttaagataatacaaccactacataataaaagagtagcgatattaaaagaatccgatgagaaaagactaatatttatctatccattcagtttgatttttcaggactttacatcgtcctgaaagtatttgttggtaccggtaccgaggacgcgtaaacatttacagttgcatgtggcctattgtttttagccgttaaatattttataactattaaatagcgatacaaattgttcgaaactaatattgtttatatcatatattctcgcatgttttaaagctttattaaattgattttttgtaaacagtttttcgtactctttgttaacccatttcattacaaaagtttcatatttttttctctctttaaatgccatttttgctggctttctttttaatacaattaatgtgctatccactttaggttttggatggaaataatacctaggaatttttgctaatatagaaatatctacctctgccattaacagcaatgctagtgatctgtttgtatctaataacattttagcaaaaccatattccactattaaataacttattgtggctgaactttcaaaaacaatttttcgaattatatttgtgcttatgttgtaaggtatgctgccaaatattttatatggattgtggctaggaaatgtaaatttcagtatatcatcatttactatttgatagttaggataatttaagagcttattacgagttacctcacataatttagaatcaatttctatcgccgttacaaaattacatctctttaccaatccagcagtaaaatgacctttccctgcacctatttcaaagatgttatctttttcatctaaacttatgcaattcattattttttctatgtgatattttgaagtaataaaattttgactatcttttatatttactttgttcattataacctctccttaatttattgcatctcttttcgaatatttatgttttttgagaaaagaacgtactcatggttcatcccgatatgcgtatcggtctgtatatcagcaactttctatgtgtttcaactacaatagtcatctattctcatctttctgagtccaccccctgcaaagcccctctttacgacataaaaattcggtcggaaaaggtatgcaaaagatgtttctctctttaagagaaactcttcgggatgcaaaaatatgaaaataactccaattcaccaaattatatagcgacttttttacaaaatgctaaaatttgttgatttccgtcaagcaattgttgagcaaaaatgtcttttacgataaaatgatacctcaatatcaactgtttagcaaaacgatatttctcttaaagagagaaacacctttttgttcaccaatccccgacttttaatcccgcggccatgattgaaaaaggaagagtatgagtattcaacatttccgtgtcgcccttattcccttttttgcggcattttgccttcctgtttttgctcacccagaaacgctggtgaaagtaaaagatgctgaagatcagttgggtgcacgagtgggttacatcgaactggatctcaacagcggtaagatccttgagagttttcgccccgaagaacgttttccaatgatgagcacttttaaagttctgctatgtggcgcggtattatcccgtattgacgccgggcaagagcaactcggtcgccgcatacactattctcagaatgacttggttgagtactcaccagtcacagaaaagcatcttacggatggcatgacagtaagagaattatgcagtgctgccataaccatgagtgataacactgcggccaacttacttctgacaacgatcggaggaccgaaggagctaaccgcttttttgcacaacatgggggatcatgtaactcgccttgatcgttgggaaccggagctgaatgaagccataccaaacgacgagcgtgacaccacgatgcctgtagcaatggcaacaacgttgcgcaaactattaactggcgaactacttactctagcttcccggcaacaattaatagactggatggaggcggataaagttgcaggaccacttctgcgctcggcccttccggctggctggtttattgctgataaatctggagccggtgagcgtgggtctcgcggtatcattgcagcactggggccagatggtaagccctcccgtatcgtagttatctacacgacggggagtcaggcaactatggatgaacgaaatagacagatcgctgagataggtgcctcactgattaagcattggtaactgtcagaccaagtttactcataacgcgtcaattcgagggggatcaattccgtgataggtgggctgcccttcctggttggcttggtttcatcagccatccgcttgccctcatctgttacgccggcggtagccggccagcctcgcagagcaggattcccgttgagcaccgccaggtgcgaataagggacagtgaagaaggaacacccgctcgcgggtgggcctacttcacctatcctgcccggctgacgccgttggatacaccaaggaaagtctacacgaaccctttggcaaaatcctgtatatcgtgcgaaaaaggatggatataccgaaaaaatcgctataatgaccccgaagcagggttatgcagcggaaaacggaattgatccggccacgatgcgtccggcgtagaggatctgaagatcagcagttcaacctgttgatagtacgtactaagctctcatgtttcacgtactaagctctcatgtttaacgtactaagctctcatgtttaacgaactaaaccctcatggctaacgtactaagctctcatggctaacgtactaagctctcatgtttcacgtactaagctctcatgtttgaacaataaaattaatataaatcagcaacttaaatagcctctaaggttttaagttttataagaaaaaaaagaatatataaggcttttaaagcttttaag

>pBH212

gtttaacggttgtggacaacaagccagggatgtaacgcactgagaagcccttagagcctctcaaagcaattttgagtgacacaggaacacttaacggctgacatgggaattcccctccaccgcggtggCCCTgtacaaagaaaattcgacaaactgttatttttctatctatttatttgAATTaggAGCGCTcctAATTtacctttgtcggcAATTaggAGCGCTcctAATTaaataaagatattctcgtcaaacaaatataaataatataaacatggtttttactctggaagattttgttggcgattggcgtcagaccgcgggttataatttggatcaagtcctggaacagggtggcgtaagctctctgttccagaacctgggtgtgagcgtgacgccgattcagcgcatcgttctgtccggcgagaacggtctgaaaattgatattcatgtgatcatcccgtacgaaggcctgagcggtgaccaaatgggtcaaatcgagaaaatctttaaagtcgtctacccagttgacgatcaccacttcaaggttatcttgcattacggtacgctggtgattgatggtgtgaccccgaatatgattgactatttcggccgtccgtatgaaggcattgccgtttttgacggtaaaaagatcaccgtcaccggtaccctgtggaatggcaataagattattgacgagcgtctgattaacccggacggcagcctgctgttccgcgtgaccatcaacggtgtcacgggttggcgtctgtgcgagcgcatcctggcataatgaactgcacttgctttgataattaatgataaacaatctaaaagcactctaatcgttatcggagtgcttttagattactaatcaaattgcttctactaattgcctatcttccagtgatggaacagcatttgAACGcattggctgcaacaatcagccttgatctggaagaagcaatgaaagctgctgttaagtctccgaatcaggtattgttcctgacaggtgtattcccatccggtaaacgcggatactttgcagttgatctgactcaggaataaattataaattaaggtaagaagattgtaggataagctaatgaaatagaaaaaggatgccgtcacacaacttgtcggcattcttttttgttttattagttgaaaatatagtgaaaaagttgcctaaatatgtatgttaacaaattatttgtcgtaactttgcactccaaatctgtttttaacatatggcactagtgAAACCAGTAACGTTATACGATGTCGCAGAGTATGCCGGTGTCTCTAAAAGCACCGTTTCCCTGGTGGTGAACCAGGCCAGCCACGTTTCTGCGAAAACGCGGGAAAAAGTGGAAGCGGCGATGGCGGAGCTGAATTACATTCCCAACCGCGTGGCACAACAACTGGCGGGCAAACAGTCGTTGCTGATTGGCGTTGCCACCTCCAGTCTGGCCCTGCACGCGCTGTCGCAAATTGTCGCGGCGATTAAATCTCGCGCCTATCAACTGGGTGCCAGCGTGTTCGTGTCGATGGTAGAACGAAGCGGCATCGAAGCCTGTAAAACGGCGGTGCACAATCTTCTCGCGCAACGCGTCAGTGGGCTGATCATTAACTATCCGCTGGATAACCAGGATGCCATTGCTGTGGAAGCTGCCTGCACTAATGTTCCGGCGTTATTTCTTGATGTCTCTGACCAGACACCCATCAACAGTATTATTTTCTCCCATGAAGACGGTACGCGACTGGGCGTGGAGCATCTGGTCGCATTGGGTCACCAGCAAATCGCGCTGTTAGCGGGCCCATTAAGTTCTGTCTCGGCGCGTCTGCGTCTGGCTGGCTGGCATAAATATCTCACTCGCAATCAAATTCAGCCGATAGCGGAACGGGAAGGCGACTGGAGTGCCATGTCCGGTTTTCAACAAACCATGCAAATGCTGAATGAGGGCATCGTTCCCACTGCGATGCTGGTTGCCAACGATCAGATGGCGCTGGGCGCAATGCGCGCCATTACCGAGACCGGGCTGCGCGTTGGTGCGGATATCTCGGTAGTGGGATACGACGATACCGAAGACAGCTCATGTTATATCCCGCCGTTAACCACCATCAAACAGGATTTTCGCCTGCTGGGGCAAACCAGCGTGGACCGCTTGCTGCAACTCTCTCAGGGCCAGGCGGTGAAGGGCAATCAGCTGTTGCCCGTCTCACTGGTGAAAAGAAAAACCACCCTGGCGCCCAATACGCAAACCGCCTCTCCCCGCGCGTTGGCCGATTCATTAATGCAGCTGGCACGACAGGTTTCCCGACTGGAAAGCGGGCAGtgagtgcaacaatcagccttgatctggaagaagcaatgaaagctgctgttaagtctccgaatcaggtattgttcctgacaggtgtattcccatccggtaaacgcggatactttgcagttgatctgactcaggaataaattataaattaaggtaagaagattgtaggataagctaatgaaatagaaaaaggatgccgtcacacaacttgtcggcattcttttttgttttattagttgaaaatatagtgaaaaagttgcctaaatatgtatgttaacaaattatttgtcgtaactttgcactccaaatctgtttttaacatatggcactagtgAAACCAGTAACGTTATACGATGTCGCAGAGTATGCCGGTGTCTCTAAAAGCACCGTTTCCCTGGTGGTGAACCAGGCCAGCCACGTTTCTGCGAAAACGCGGGAAAAAGTGGAAGCGGCGATGGCGGAGCTCAATTACATTCCCAACCGCGTGGCACAACAACTGGCGGGCAAAGCGTCGCATACCATTGGCATGTTGATCACTGCCAGTACCAATCCTTTCTATTCAGAACTGGTGCGTGGCGTTGAACGCAGCTGCTTCGAACGCGGTTATAGTCTCGtcCTTTGCAATACCGAAGGCGATGAACAGCGGATGAATCGCAATCTGGAAACGCTGATGCAAAAACGCGTTGATGGCTTGCTGTTACTGTGCACCGAAACGCATCAACCTTCGCGTGAAATCATGCAACGTTATCCGACAGTGCCTACTGTGATGATGGACTGGGCTCCGTTCGATGGCGACAGCGATCTTATTCAGGATAACTCGTTGCTGGGCGGAGACTTAGCAACGCAATATCTGATCGATAAAGGTCATACCCGTATCGCCTGTATTACCGGCCCGCTGGATAAAACTCCGGCGCGCCTGCGGTTGGAAGGTTATCGGGCGGCGATGAAACGTGCGGGTCTCAACATTCCTGATGGCTATGAAGTCACTGGTGATTTTGAATTTAACGGCGGGTTTGACGCTATGCGCCAACTGCTATCACATCCGCTGCGTCCTCAGGCCGTCTTTACCGGAAATGACGCTATGGCTGTTGGCGTTTACCAGGCGTTATATCAGGCAGAGTTACAGGTTCCGCAGGATATCGCGGTGATTGGCTATGACGATATCGAACTGGCAAGCTTTATGACGCCACCATTAACCACTATCCACCAACCGAAAGATGAACTGGGGGAGCTGGCGATTGATGTACTCATCCATCGGATAACCCAGCCGACCCTTCAGCAACAACGATTACAACTTACTCCGATTCTGATGGAACGCGGTTCGGCTTAGCTGGTGAAAAGAAAAACCACCCTGGCGCCCAATACGCAAACCGCCTCTCCCCGCGCGTTGGCCGATTCATTAATGCAGCTGGCACGACAGGTTTCCCGACTGGAAAGCGGGCAGTGAgctttcctcggtaccaaattccagaaaagaggcctcccgaaaggggggccttttttcgttttggtcctacttgtgcctgttctatttccATCCcggttcctggccttttgctggccttttgctcacatgttctttcctgcgttatcccctgattctgtggataaccgtattaccgcctttgagtgagctgatacCCGAtacttgtgcctgttctatttccgaaccgaccgcttgtatgaatccatcaaaattcgttttctctatgttggattccttgttgctcatattgtgatgataatttctacaaatatagtcattggtaactatctatgaaactgtttgatacttttatagttgattaaacttgttcatggcatttgccttaatatcatccgctatgtcaatgtagggtttcatagctttgtagtcgctgtgtcccgtccatttcatgaccacctgtgccgggattccgagagccagcgcattgcagatgaatgtccttcttcctgcatgggtactgagcaaagcgtatttgggtgtgacttcatcaatacgttcatttcccttgtagtaggtttcccgtacaggctcgttgatttctgccagttcgcccagctctttcaggtaatcgttcatcttctggttgctgatgacgggcagagccatgtaattctcgaaatggatgtccttgtatttgtccagtatggctttgctgtatttgttcagttcaatcgtcaggctgtcggcagtcttgactgtggttatttcgatgtggtcggacttcacatcgcttcttttcagattgcgaacatccgaataccgcaaactcgtaaagcagcagaacaggaaaacatcacgcacacgttccaggtattgcttatccttgggtatctggtagtctttcagcttgttcagttcatcccaagtcaggaagattacttttttcgaggtggttttcagtttcggtttgaacgtatcgtatgcaatgttctgatgatgtcctttcctgaagctccagcgcaggaaccatttgaggaatcccatttgcttgccgatggtgctgtttctcatatccttggtgtcacgcaggaagttgacgtattcgttcaatccaaactcgttgaaatagttgaacgttgcatcctccttgaactctttgaggtggttcctcactgctgcaaatttttcataggtggatgccgtccagttattctggttaccgcactcttttacaaactcatcgaacacctcccaaaagctgacaggggcttcttccggctgttcttcactggtgtctttcattctcatgttgaaagcttccttcaactgttgggtcgttggcatgacctcctgcacctcaaattccttgaaaatattctggatttcggcatagtatttcagcaagtccgtattgatttcggctgcactttgctttagcttgttggtacatccgttctttacccgctgcttatctacatcccatttggctacgtcaatccggtagcccgttgtaaactcgatacgttggctggcaaagatgacacgcatacggatgggtacgttctctacgattggcacaccgttctttttccggctctccaatgcaaaaatgatgttgcgcttgatattcataattgggtgcgtttgaaattctacacccaaatatacacccaattattgagatagcaaaagacatttagaaacatttacttttactctatattgtaatttacacttgattatcagtcgtttgcagtTttatgatattctgtgaaagtataagttcgagagcctgtctctccgcaaaaaacgctgaaaatcagcagattgcaaaacaaacaccctgttttacacccaagaatgtaaagtcggctgtttttgttttatttaagataatacaaccactacataataaaagagtagcgatattaaaagaatccgatgagaaaagactaatatttatctatccattcagtttgatttttcaggactttacatcgtcctgaaagtatttgttggtaccggtaccgaggacgcgtaaacatttacagttgcatgtggcctattgtttttagccgttaaatattttataactattaaatagcgatacaaattgttcgaaactaatattgtttatatcatatattctcgcatgttttaaagctttattaaattgattttttgtaaacagtttttcgtactctttgttaacccatttcattacaaaagtttcatatttttttctctctttaaatgccatttttgctggctttctttttaatacaattaatgtgctatccactttaggttttggatggaaataatacctaggaatttttgctaatatagaaatatctacctctgccattaacagcaatgctagtgatctgtttgtatctaataacattttagcaaaaccatattccactattaaataacttattgtggctgaactttcaaaaacaatttttcgaattatatttgtgcttatgttgtaaggtatgctgccaaatattttatatggattgtggctaggaaatgtaaatttcagtatatcatcatttactatttgatagttaggataatttaagagcttattacgagttacctcacataatttagaatcaatttctatcgccgttacaaaattacatctctttaccaatccagcagtaaaatgacctttccctgcacctatttcaaagatgttatctttttcatctaaacttatgcaattcattattttttctatgtgatattttgaagtaataaaattttgactatcttttatatttactttgttcattataacctctccttaatttattgcatctcttttcgaatatttatgttttttgagaaaagaacgtactcatggttcatcccgatatgcgtatcggtctgtatatcagcaactttctatgtgtttcaactacaatagtcatctattctcatctttctgagtccaccccctgcaaagcccctctttacgacataaaaattcggtcggaaaaggtatgcaaaagatgtttctctctttaagagaaactcttcgggatgcaaaaatatgaaaataactccaattcaccaaattatatagcgacttttttacaaaatgctaaaatttgttgatttccgtcaagcaattgttgagcaaaaatgtcttttacgataaaatgatacctcaatatcaactgtttagcaaaacgatatttctcttaaagagagaaacacctttttgttcaccaatccccgacttttaatcccgcggccatgattgaaaaaggaagagtatgagtattcaacatttccgtgtcgcccttattcccttttttgcggcattttgccttcctgtttttgctcacccagaaacgctggtgaaagtaaaagatgctgaagatcagttgggtgcacgagtgggttacatcgaactggatctcaacagcggtaagatccttgagagttttcgccccgaagaacgttttccaatgatgagcacttttaaagttctgctatgtggcgcggtattatcccgtattgacgccgggcaagagcaactcggtcgccgcatacactattctcagaatgacttggttgagtactcaccagtcacagaaaagcatcttacggatggcatgacagtaagagaattatgcagtgctgccataaccatgagtgataacactgcggccaacttacttctgacaacgatcggaggaccgaaggagctaaccgcttttttgcacaacatgggggatcatgtaactcgccttgatcgttgggaaccggagctgaatgaagccataccaaacgacgagcgtgacaccacgatgcctgtagcaatggcaacaacgttgcgcaaactattaactggcgaactacttactctagcttcccggcaacaattaatagactggatggaggcggataaagttgcaggaccacttctgcgctcggcccttccggctggctggtttattgctgataaatctggagccggtgagcgtgggtctcgcggtatcattgcagcactggggccagatggtaagccctcccgtatcgtagttatctacacgacggggagtcaggcaactatggatgaacgaaatagacagatcgctgagataggtgcctcactgattaagcattggtaactgtcagaccaagtttactcataacgcgtcaattcgagggggatcaattccgtgataggtgggctgcccttcctggttggcttggtttcatcagccatccgcttgccctcatctgttacgccggcggtagccggccagcctcgcagagcaggattcccgttgagcaccgccaggtgcgaataagggacagtgaagaaggaacacccgctcgcgggtgggcctacttcacctatcctgcccggctgacgccgttggatacaccaaggaaagtctacacgaaccctttggcaaaatcctgtatatcgtgcgaaaaaggatggatataccgaaaaaatcgctataatgaccccgaagcagggttatgcagcggaaaacggaattgatccggccacgatgcgtccggcgtagaggatctgaagatcagcagttcaacctgttgatagtacgtactaagctctcatgtttcacgtactaagctctcatgtttaacgtactaagctctcatgtttaacgaactaaaccctcatggctaacgtactaagctctcatggctaacgtactaagctctcatgtttcacgtactaagctctcatgtttgaacaataaaattaatataaatcagcaacttaaatagcctctaaggttttaagttttataagaaaaaaaagaatatataaggcttttaaagcttttaag

>pBH301

gtttaacggttgtggacaacaagccagggatgtaacgcactgagaagcccttagagcctctcaaagcaattttgagtgacacaggaacacttaacggctgacatgggaattcccctccaccgcggtggCCCTgtacaaagaaaattcgacaaactgttatttttctatctatttatttgAATTttgAGCGCTcaaAATTtacctttgtcggcAATTttgAGCGCTcaaAATTaaataaagatattctcgtcaaacaaatataaataatataaacatggtttttactctggaagattttgttggcgattggcgtcagaccgcgggttataatttggatcaagtcctggaacagggtggcgtaagctctctgttccagaacctgggtgtgagcgtgacgccgattcagcgcatcgttctgtccggcgagaacggtctgaaaattgatattcatgtgatcatcccgtacgaaggcctgagcggtgaccaaatgggtcaaatcgagaaaatctttaaagtcgtctacccagttgacgatcaccacttcaaggttatcttgcattacggtacgctggtgattgatggtgtgaccccgaatatgattgactatttcggccgtccgtatgaaggcattgccgtttttgacggtaaaaagatcaccgtcaccggtaccctgtggaatggcaataagattattgacgagcgtctgattaacccggacggcagcctgctgttccgcgtgaccatcaacggtgtcacgggttggcgtctgtgcgagcgcatcctggcataatgaactgcacttgctttgataattaatgataaacaatctaaaagcactctaatcgttatcggagtgcttttagattactaatcaaattgcttctactaattgcctatcttccagtgatggaacagcatttgAACGcattggctgcaacaatcagccttgatctggaagaagcaatgaaagctgctgttaagtctccgaatcaggtattgttcctgacaggtgtattcccatccggtaaacgcggatactttgcagttgatctgactcaggaataaattataaattaaggtaagaagattgtaggataagctaatgaaatagaaaaaggatgccgtcacacaacttgtcggcattcttttttgttttattagttgaaaatatagtgaaaaagttgcctaaatatgtatgttaacaaattatttgtcgtaactttgcactccaaatctgtttttaacatatggcactagtgAAACCAGTAACGTTATACGATGTCGCAGAGTATGCCGGTGTCTCTTATCAGACCGTTTCCCGCGTGGTGAACCAGGCCAGCCACGTTTCTGCGAAAACGCGGGAAAAAGTGGAAGCGGCGATGGCGGAGCTGAATTACATTCCCAACCGCGTGGCACAACAACTGGCGGGCAAACAGTCGTTGCTGATTGGCGTTGCCACCTCCAGTCTGGCCCTGCACGCGCTGTCGCAAATTGTCGCGGCGATTAAATCTCGCGCCTATCAACTGGGTGCCAGCGTGTTCGTGTCGATGGTAGAACGAAGCGGCATCGAAGCCTGTAAAACGGCGGTGCACAATCTTCTCGCGCAACGCGTCAGTGGGCTGATCATTAACTATCCGCTGGATAACCAGGATGCCATTGCTGTGGAAGCTGCCTGCACTAATGTTCCGGCGTTATTTCTTGATGTCTCTGACCAGACACCCATCAACAGTATTATTTTCTCCCATGAAGACGGTACGCGACTGGGCGTGGAGCATCTGGTCGCATTGGGTCACCAGCAAATCGCGCTGTTAGCGGGCCCATTAAGTTCTGTCTCGGCGCGTCTGCGTCTGGCTGGCTGGCATAAATATCTCACTCGCAATCAAATTCAGCCGATAGCGGAACGGGAAGGCGACTGGAGTGCCATGTCCGGTTTTCAACAAACCATGCAAATGCTGAATGAGGGCATCGTTCCCACTGCGATGCTGGTTGCCAACGATCAGATGGCGCTGGGCGCAATGCGCGCCATTACCGAGACCGGGCTGCGCGTTGGTGCGGATATCTCGGTAGTGGGATACGACGATACCGAAGACAGCTCATGTTATATCCCGCCGTTAACCACCATCAAACAGGATTTTCGCCTGCTGGGGCAAACCAGCGTGGACCGCTTGCTGCAACTCTCTCAGGGCCAGGCGGTGAAGGGCAATCAGCTGTTGCCCGTCTCACTGGTGAAAAGAAAAACCACCCTGGCGCCCAATACGCAAACCGCCTCTCCCCGCGCGTTGGCCGATTCATTAATGCAGCTGGCACGACAGGTTTCCCGACTGGAAAGCGGGCAGtgagctttcctcggtaccaaattccagaaaagaggcctcccgaaaggggggccttttttcgttttggtcctacttgtgcctgttctatttccTATGcggttcctggccttttgctggccttttgctcacatgttctttcctgcgttatcccctgattctgtggataaccgtattaccgcctttgagtgagctgatacATCCcaaatgctgttccatcactggaagataggcaattagtagaagcaatttgattagtaatctaaaagcactccgataacgattagagtgcttttagattgtttatcattaattatcaaagcaagtgcagttcaTCACTGCCCGCTTTCCAGTCGGGAAACCTGTCGTGCCAGCTGCATTAATGAATCGGCCAACGCGCGGGGAGAGGCGGTTTGCGTATTGGGCGCCAGGGTGGTTTTTCTTTTCACCAGCTAAGCCGAACCGCGTTCCATCAGAATCGGAGTAAGTTGTAATCGTTGTTGCTGAAGGGTCGGCTGGGTTATCCGATGGATGAGTACATCAATCGCCAGCTCCCCCAGTTCATCTTTCGGTTGGTGGATAGTGGTTAATGGTGGCGTCATAAAGCTTGCCAGTTCGATATCGTCATAGCCAATCACCGCGATATCCTGCGGAACCTGTAACTCTGCCTGATATAACGCCTGGTAAACGCCAACAGCCATAGCGTCATTTCCGGTAAAGACGGCCTGAGGACGCAGCGGATGTGATAGCAGTTGGCGCATAGCGTCAAACCCGCCGTTAAATTCAAAATCACCAGTGACTTCATAGCCATCAGGAATGTTGAGACCCGCACGTTTCATCGCCGCCCGATAACCTTCCAACCGCAGGCGCGCCGGAGTTTTATCCAGCGGGCCGGTAATACAGGCGATACGGGTATGACCTTTATCGATCAGATATTGCGTTGCTAAGTCTCCGCCCAGCAACGAGTTATCCTGAATAAGATCGCTGTCGCCATCGAACGGAGCCCAGTCCATCATCACAGTAGGCACTGTCGGATAACGTTGCATGATTTCACGCGAAGGTTGATGCGTTTCGGTGCACAGTAACAGCAAGCCATCAACGCGTTTTTGCATCAGCGTTTCCAGATTGCGATTCATCCGCTGTTCATCGCCTTCGGTATTGCAAAGGACGAGACTATAACCGCGTTCGAAGCAGCTGCGTTCAACGCCACGCACCAGTTCTGAATAGAAAGGATTGGTACTGGCAGTGATCAACATGCCAATGGTATGCGACGCTTTGCCCGCCAGTTGTTGTGCCACGCGGTTGGGAATGTAATTGAGCTCCGCCATCGCCGCTTCCACTTTTTCCCGCGTTTTCGCAGAAACGTGGCTGGCCTGGTTCACCACattGGAAACGGTctgatgAGAGACACCGGCATACTCTGCGACATCGTATAACGTTACTGGTTTcacgtttatattatttatatttgtttgacgagaatatctttatttaattgttatccgctcacaattgccgacaaaggtaattgttatccgctcacaattcaaataaatagatagaaaaataacagtttgtcgaattttctttgtacCCGAtacttgtgcctgttctatttccgaaccgaccgcttgtatgaatccatcaaaattcgttttctctatgttggattccttgttgctcatattgtgatgataatttctacaaatatagtcattggtaactatctatgaaactgtttgatacttttatagttgattaaacttgttcatggcatttgccttaatatcatccgctatgtcaatgtagggtttcatagctttgtagtcgctgtgtcccgtccatttcatgaccacctgtgccgggattccgagagccagcgcattgcagatgaatgtccttcttcctgcatgggtactgagcaaagcgtatttgggtgtgacttcatcaatacgttcatttcccttgtagtaggtttcccgtacaggctcgttgatttctgccagttcgcccagctctttcaggtaatcgttcatcttctggttgctgatgacgggcagagccatgtaattctcgaaatggatgtccttgtatttgtccagtatggctttgctgtatttgttcagttcaatcgtcaggctgtcggcagtcttgactgtggttatttcgatgtggtcggacttcacatcgcttcttttcagattgcgaacatccgaataccgcaaactcgtaaagcagcagaacaggaaaacatcacgcacacgttccaggtattgcttatccttgggtatctggtagtctttcagcttgttcagttcatcccaagtcaggaagattacttttttcgaggtggttttcagtttcggtttgaacgtatcgtatgcaatgttctgatgatgtcctttcctgaagctccagcgcaggaaccatttgaggaatcccatttgcttgccgatggtgctgtttctcatatccttggtgtcacgcaggaagttgacgtattcgttcaatccaaactcgttgaaatagttgaacgttgcatcctccttgaactctttgaggtggttcctcactgctgcaaatttttcataggtggatgccgtccagttattctggttaccgcactcttttacaaactcatcgaacacctcccaaaagctgacaggggcttcttccggctgttcttcactggtgtctttcattctcatgttgaaagcttccttcaactgttgggtcgttggcatgacctcctgcacctcaaattccttgaaaatattctggatttcggcatagtatttcagcaagtccgtattgatttcggctgcactttgctttagcttgttggtacatccgttctttacccgctgcttatctacatcccatttggctacgtcaatccggtagcccgttgtaaactcgatacgttggctggcaaagatgacacgcatacggatgggtacgttctctacgattggcacaccgttctttttccggctctccaatgcaaaaatgatgttgcgcttgatattcataattgggtgcgtttgaaattctacacccaaatatacacccaattattgagatagcaaaagacatttagaaacatttacttttactctatattgtaatttacacttgattatcagtcgtttgcagtTttatgatattctgtgaaagtataagttcgagagcctgtctctccgcaaaaaacgctgaaaatcagcagattgcaaaacaaacaccctgttttacacccaagaatgtaaagtcggctgtttttgttttatttaagataatacaaccactacataataaaagagtagcgatattaaaagaatccgatgagaaaagactaatatttatctatccattcagtttgatttttcaggactttacatcgtcctgaaagtatttgttggtaccggtaccgaggacgcgtaaacatttacagttgcatgtggcctattgtttttagccgttaaatattttataactattaaatagcgatacaaattgttcgaaactaatattgtttatatcatatattctcgcatgttttaaagctttattaaattgattttttgtaaacagtttttcgtactctttgttaacccatttcattacaaaagtttcatatttttttctctctttaaatgccatttttgctggctttctttttaatacaattaatgtgctatccactttaggttttggatggaaataatacctaggaatttttgctaatatagaaatatctacctctgccattaacagcaatgctagtgatctgtttgtatctaataacattttagcaaaaccatattccactattaaataacttattgtggctgaactttcaaaaacaatttttcgaattatatttgtgcttatgttgtaaggtatgctgccaaatattttatatggattgtggctaggaaatgtaaatttcagtatatcatcatttactatttgatagttaggataatttaagagcttattacgagttacctcacataatttagaatcaatttctatcgccgttacaaaattacatctctttaccaatccagcagtaaaatgacctttccctgcacctatttcaaagatgttatctttttcatctaaacttatgcaattcattattttttctatgtgatattttgaagtaataaaattttgactatcttttatatttactttgttcattataacctctccttaatttattgcatctcttttcgaatatttatgttttttgagaaaagaacgtactcatggttcatcccgatatgcgtatcggtctgtatatcagcaactttctatgtgtttcaactacaatagtcatctattctcatctttctgagtccaccccctgcaaagcccctctttacgacataaaaattcggtcggaaaaggtatgcaaaagatgtttctctctttaagagaaactcttcgggatgcaaaaatatgaaaataactccaattcaccaaattatatagcgacttttttacaaaatgctaaaatttgttgatttccgtcaagcaattgttgagcaaaaatgtcttttacgataaaatgatacctcaatatcaactgtttagcaaaacgatatttctcttaaagagagaaacacctttttgttcaccaatccccgacttttaatcccgcggccatgattgaaaaaggaagagtatgagtattcaacatttccgtgtcgcccttattcccttttttgcggcattttgccttcctgtttttgctcacccagaaacgctggtgaaagtaaaagatgctgaagatcagttgggtgcacgagtgggttacatcgaactggatctcaacagcggtaagatccttgagagttttcgccccgaagaacgttttccaatgatgagcacttttaaagttctgctatgtggcgcggtattatcccgtattgacgccgggcaagagcaactcggtcgccgcatacactattctcagaatgacttggttgagtactcaccagtcacagaaaagcatcttacggatggcatgacagtaagagaattatgcagtgctgccataaccatgagtgataacactgcggccaacttacttctgacaacgatcggaggaccgaaggagctaaccgcttttttgcacaacatgggggatcatgtaactcgccttgatcgttgggaaccggagctgaatgaagccataccaaacgacgagcgtgacaccacgatgcctgtagcaatggcaacaacgttgcgcaaactattaactggcgaactacttactctagcttcccggcaacaattaatagactggatggaggcggataaagttgcaggaccacttctgcgctcggcccttccggctggctggtttattgctgataaatctggagccggtgagcgtgggtctcgcggtatcattgcagcactggggccagatggtaagccctcccgtatcgtagttatctacacgacggggagtcaggcaactatggatgaacgaaatagacagatcgctgagataggtgcctcactgattaagcattggtaactgtcagaccaagtttactcataacgcgtcaattcgagggggatcaattccgtgataggtgggctgcccttcctggttggcttggtttcatcagccatccgcttgccctcatctgttacgccggcggtagccggccagcctcgcagagcaggattcccgttgagcaccgccaggtgcgaataagggacagtgaagaaggaacacccgctcgcgggtgggcctacttcacctatcctgcccggctgacgccgttggatacaccaaggaaagtctacacgaaccctttggcaaaatcctgtatatcgtgcgaaaaaggatggatataccgaaaaaatcgctataatgaccccgaagcagggttatgcagcggaaaacggaattgatccggccacgatgcgtccggcgtagaggatctgaagatcagcagttcaacctgttgatagtacgtactaagctctcatgtttcacgtactaagctctcatgtttaacgtactaagctctcatgtttaacgaactaaaccctcatggctaacgtactaagctctcatggctaacgtactaagctctcatgtttcacgtactaagctctcatgtttgaacaataaaattaatataaatcagcaacttaaatagcctctaaggttttaagttttataagaaaaaaaagaatatataaggcttttaaagcttttaag

>pBH302

gtttaacggttgtggacaacaagccagggatgtaacgcactgagaagcccttagagcctctcaaagcaattttgagtgacacaggaacacttaacggctgacatgggaattcccctccaccgcggtggCCCTgtacaaagaaaattcgacaaactgttatttttctatctatttatttgAATTttgAGCGCTcaaAATTtacctttgtcggcAATTttgAGCGCTcaaAATTaaataaagatattctcgtcaaacaaatataaataatataaacatggtttttactctggaagattttgttggcgattggcgtcagaccgcgggttataatttggatcaagtcctggaacagggtggcgtaagctctctgttccagaacctgggtgtgagcgtgacgccgattcagcgcatcgttctgtccggcgagaacggtctgaaaattgatattcatgtgatcatcccgtacgaaggcctgagcggtgaccaaatgggtcaaatcgagaaaatctttaaagtcgtctacccagttgacgatcaccacttcaaggttatcttgcattacggtacgctggtgattgatggtgtgaccccgaatatgattgactatttcggccgtccgtatgaaggcattgccgtttttgacggtaaaaagatcaccgtcaccggtaccctgtggaatggcaataagattattgacgagcgtctgattaacccggacggcagcctgctgttccgcgtgaccatcaacggtgtcacgggttggcgtctgtgcgagcgcatcctggcataatgaactgcacttgctttgataattaatgataaacaatctaaaagcactctaatcgttatcggagtgcttttagattactaatcaaattgcttctactaattgcctatcttccagtgatggaacagcatttgAACGcattggctgcaacaatcagccttgatctggaagaagcaatgaaagctgctgttaagtctccgaatcaggtattgttcctgacaggtgtattcccatccggtaaacgcggatactttgcagttgatctgactcaggaataaattataaattaaggtaagaagattgtaggataagctaatgaaatagaaaaaggatgccgtcacacaacttgtcggcattcttttttgttttattagttgaaaatatagtgaaaaagttgcctaaatatgtatgttaacaaattatttgtcgtaactttgcactccaaatctgtttttaacatatggcactagtgAAACCAGTAACGTTATACGATGTCGCAGAGTATGCCGGTGTCTCTACCGCGACCGTTTCCAACGTGGTGAACCAGGCCAGCCACGTTTCTGCGAAAACGCGGGAAAAAGTGGAAGCGGCGATGGCGGAGCTCAATTACATTCCCAACCGCGTGGCACAACAACTGGCGGGCAAAGCGTCGCATACCATTGGCATGTTGATCACTGCCAGTACCAATCCTTTCTATTCAGAACTGGTGCGTGGCGTTGAACGCAGCTGCTTCGAACGCGGTTATAGTCTCGTCCTTTGCAATACCGAAGGCGATGAACAGCGGATGAATCGCAATCTGGAAACGCTGATGCAAAAACGCGTTGATGGCTTGCTGTTACTGTGCACCGAAACGCATCAACCTTCGCGTGAAATCATGCAACGTTATCCGACAGTGCCTACTGTGATGATGGACTGGGCTCCGTTCGATGGCGACAGCGATCTTATTCAGGATAACTCGTTGCTGGGCGGAGACTTAGCAACGCAATATCTGATCGATAAAGGTCATACCCGTATCGCCTGTATTACCGGCCCGCTGGATAAAACTCCGGCGCGCCTGCGGTTGGAAGGTTATCGGGCGGCGATGAAACGTGCGGGTCTCAACATTCCTGATGGCTATGAAGTCACTGGTGATTTTGAATTTAACGGCGGGTTTGACGCTATGCGCCAACTGCTATCACATCCGCTGCGTCCTCAGGCCGTCTTTACCGGAAATGACGCTATGGCTGTTGGCGTTTACCAGGCGTTATATCAGGCAGAGTTACAGGTTCCGCAGGATATCGCGGTGATTGGCTATGACGATATCGAACTGGCAAGCTTTATGACGCCACCATTAACCACTATCCACCAACCGAAAGATGAACTGGGGGAGCTGGCGATTGATGTACTCATCCATCGGATAACCCAGCCGACCCTTCAGCAACAACGATTACAACTTACTCCGATTCTGATGGAACGCGGTTCGGCTTAGCTGGTGAAAAGAAAAACCACCCTGGCGCCCAATACGCAAACCGCCTCTCCCCGCGCGTTGGCCGATTCATTAATGCAGCTGGCACGACAGGTTTCCCGACTGGAAAGCGGGCAGTGAgctttcctcggtaccaaattccagaaaagaggcctcccgaaaggggggccttttttcgttttggtcctacttgtgcctgttctatttccTATGcggttcctggccttttgctggccttttgctcacatgttctttcctgcgttatcccctgattctgtggataaccgtattaccgcctttgagtgagctgatacATCCcaaatgctgttccatcactggaagataggcaattagtagaagcaatttgattagtaatctaaaagcactccgataacgattagagtgcttttagattgtttatcattaattatcaaagcaagtgcagttcatcaCTGCCCGCTTTCCAGTCGGGAAACCTGTCGTGCCAGCTGCATTAATGAATCGGCCAACGCGCGGGGAGAGGCGGTTTGCGTATTGGGCGCCAGGGTGGTTTTTCTTTTCACCAGTGAGACGGGCAACAGCTGATTGCCCTTCACCGCCTGGCCCTGAGAGAGTTGCAGCAAGCGGTCCACGCTGGTTTGCCCCAGCAGGCGAAAATCCTGTTTGATGGTGGTTAACGGCGGGATATAACATGAGCTGTCTTCGGTATCGTCGTATCCCACTACCGAGATATCCGCACCAACGCGCAGCCCGGTCTCGGTAATGGCGCGCATTGCGCCCAGCGCCATCTGATCGTTGGCAACCAGCATCGCAGTGGGAACGATGCCCTCATTCAGCATTTGCATGGTTTGTTGAAAACCGGACATGGCACTCCAGTCGCCTTCCCGTTCCGCTATCGGCTGAATTTGATTGCGAGTGAGATATTTATGCCAGCCAGCCAGACGCAGACGCGCCGAGACAGAACTTAATGGGCCCGCTAACAGCGCGATTTGCTGGTGACCCAATGCGACCAGATGCTCCACGCCCAGTCGCGTACCGTCTTCATGGGAGAAAATAATACTGTTGATGGGTGTCTGGTCAGAGACATCAAGAAATAACGCCGGAACATTAGTGCAGGCAGCTTCCACAGCAATGGCATCCTGGTTATCCAGCGGATAGTTAATGATCAGCCCACTGACGCGTTGCGCGAGAAGATTGTGCACCGCCGTTTTACAGGCTTCGATGCCGCTTCGTTCTACCATCGACACGAACACGCTGGCACCCAGTTGATAGGCGCGAGATTTAATCGCCGCGACAATTTGCGACAGCGCGTGCAGGGCCAGACTGGAGGTGGCAACGCCAATCAGCAACGACTGTTTGCCCGCCAGTTGTTGTGCCACGCGGTTGGGAATGTAATTCAGCTCCGCCATCGCCGCTTCCACTTTTTCCCGCGTTTTCGCAGAAACGTGGCTGGCCTGGTTCACCACattGGAAACGGTctgatgAGAGACACCGGCATACTCTGCGACATCGTATAACGTTACTGGTTTcacgtttatattatttatatttgtttgacgagaatatctttatttaattTTAAGCGCTTAAaattgccgacaaaggtaaattTTAAGCGCTTAAaattcaaataaatagatagaaaaataacagtttgtcgaattttctttgtacCCGAtacttgtgcctgttctatttccgaaccgaccgcttgtatgaatccatcaaaattcgttttctctatgttggattccttgttgctcatattgtgatgataatttctacaaatatagtcattggtaactatctatgaaactgtttgatacttttatagttgattaaacttgttcatggcatttgccttaatatcatccgctatgtcaatgtagggtttcatagctttgtagtcgctgtgtcccgtccatttcatgaccacctgtgccgggattccgagagccagcgcattgcagatgaatgtccttcttcctgcatgggtactgagcaaagcgtatttgggtgtgacttcatcaatacgttcatttcccttgtagtaggtttcccgtacaggctcgttgatttctgccagttcgcccagctctttcaggtaatcgttcatcttctggttgctgatgacgggcagagccatgtaattctcgaaatggatgtccttgtatttgtccagtatggctttgctgtatttgttcagttcaatcgtcaggctgtcggcagtcttgactgtggttatttcgatgtggtcggacttcacatcgcttcttttcagattgcgaacatccgaataccgcaaactcgtaaagcagcagaacaggaaaacatcacgcacacgttccaggtattgcttatccttgggtatctggtagtctttcagcttgttcagttcatcccaagtcaggaagattacttttttcgaggtggttttcagtttcggtttgaacgtatcgtatgcaatgttctgatgatgtcctttcctgaagctccagcgcaggaaccatttgaggaatcccatttgcttgccgatggtgctgtttctcatatccttggtgtcacgcaggaagttgacgtattcgttcaatccaaactcgttgaaatagttgaacgttgcatcctccttgaactctttgaggtggttcctcactgctgcaaatttttcataggtggatgccgtccagttattctggttaccgcactcttttacaaactcatcgaacacctcccaaaagctgacaggggcttcttccggctgttcttcactggtgtctttcattctcatgttgaaagcttccttcaactgttgggtcgttggcatgacctcctgcacctcaaattccttgaaaatattctggatttcggcatagtatttcagcaagtccgtattgatttcggctgcactttgctttagcttgttggtacatccgttctttacccgctgcttatctacatcccatttggctacgtcaatccggtagcccgttgtaaactcgatacgttggctggcaaagatgacacgcatacggatgggtacgttctctacgattggcacaccgttctttttccggctctccaatgcaaaaatgatgttgcgcttgatattcataattgggtgcgtttgaaattctacacccaaatatacacccaattattgagatagcaaaagacatttagaaacatttacttttactctatattgtaatttacacttgattatcagtcgtttgcagtTttatgatattctgtgaaagtataagttcgagagcctgtctctccgcaaaaaacgctgaaaatcagcagattgcaaaacaaacaccctgttttacacccaagaatgtaaagtcggctgtttttgttttatttaagataatacaaccactacataataaaagagtagcgatattaaaagaatccgatgagaaaagactaatatttatctatccattcagtttgatttttcaggactttacatcgtcctgaaagtatttgttggtaccggtaccgaggacgcgtaaacatttacagttgcatgtggcctattgtttttagccgttaaatattttataactattaaatagcgatacaaattgttcgaaactaatattgtttatatcatatattctcgcatgttttaaagctttattaaattgattttttgtaaacagtttttcgtactctttgttaacccatttcattacaaaagtttcatatttttttctctctttaaatgccatttttgctggctttctttttaatacaattaatgtgctatccactttaggttttggatggaaataatacctaggaatttttgctaatatagaaatatctacctctgccattaacagcaatgctagtgatctgtttgtatctaataacattttagcaaaaccatattccactattaaataacttattgtggctgaactttcaaaaacaatttttcgaattatatttgtgcttatgttgtaaggtatgctgccaaatattttatatggattgtggctaggaaatgtaaatttcagtatatcatcatttactatttgatagttaggataatttaagagcttattacgagttacctcacataatttagaatcaatttctatcgccgttacaaaattacatctctttaccaatccagcagtaaaatgacctttccctgcacctatttcaaagatgttatctttttcatctaaacttatgcaattcattattttttctatgtgatattttgaagtaataaaattttgactatcttttatatttactttgttcattataacctctccttaatttattgcatctcttttcgaatatttatgttttttgagaaaagaacgtactcatggttcatcccgatatgcgtatcggtctgtatatcagcaactttctatgtgtttcaactacaatagtcatctattctcatctttctgagtccaccccctgcaaagcccctctttacgacataaaaattcggtcggaaaaggtatgcaaaagatgtttctctctttaagagaaactcttcgggatgcaaaaatatgaaaataactccaattcaccaaattatatagcgacttttttacaaaatgctaaaatttgttgatttccgtcaagcaattgttgagcaaaaatgtcttttacgataaaatgatacctcaatatcaactgtttagcaaaacgatatttctcttaaagagagaaacacctttttgttcaccaatccccgacttttaatcccgcggccatgattgaaaaaggaagagtatgagtattcaacatttccgtgtcgcccttattcccttttttgcggcattttgccttcctgtttttgctcacccagaaacgctggtgaaagtaaaagatgctgaagatcagttgggtgcacgagtgggttacatcgaactggatctcaacagcggtaagatccttgagagttttcgccccgaagaacgttttccaatgatgagcacttttaaagttctgctatgtggcgcggtattatcccgtattgacgccgggcaagagcaactcggtcgccgcatacactattctcagaatgacttggttgagtactcaccagtcacagaaaagcatcttacggatggcatgacagtaagagaattatgcagtgctgccataaccatgagtgataacactgcggccaacttacttctgacaacgatcggaggaccgaaggagctaaccgcttttttgcacaacatgggggatcatgtaactcgccttgatcgttgggaaccggagctgaatgaagccataccaaacgacgagcgtgacaccacgatgcctgtagcaatggcaacaacgttgcgcaaactattaactggcgaactacttactctagcttcccggcaacaattaatagactggatggaggcggataaagttgcaggaccacttctgcgctcggcccttccggctggctggtttattgctgataaatctggagccggtgagcgtgggtctcgcggtatcattgcagcactggggccagatggtaagccctcccgtatcgtagttatctacacgacggggagtcaggcaactatggatgaacgaaatagacagatcgctgagataggtgcctcactgattaagcattggtaactgtcagaccaagtttactcataacgcgtcaattcgagggggatcaattccgtgataggtgggctgcccttcctggttggcttggtttcatcagccatccgcttgccctcatctgttacgccggcggtagccggccagcctcgcagagcaggattcccgttgagcaccgccaggtgcgaataagggacagtgaagaaggaacacccgctcgcgggtgggcctacttcacctatcctgcccggctgacgccgttggatacaccaaggaaagtctacacgaaccctttggcaaaatcctgtatatcgtgcgaaaaaggatggatataccgaaaaaatcgctataatgaccccgaagcagggttatgcagcggaaaacggaattgatccggccacgatgcgtccggcgtagaggatctgaagatcagcagttcaacctgttgatagtacgtactaagctctcatgtttcacgtactaagctctcatgtttaacgtactaagctctcatgtttaacgaactaaaccctcatggctaacgtactaagctctcatggctaacgtactaagctctcatgtttcacgtactaagctctcatgtttgaacaataaaattaatataaatcagcaacttaaatagcctctaaggttttaagttttataagaaaaaaaagaatatataaggcttttaaagcttttaag

>pBH303

gtttaacggttgtggacaacaagccagggatgtaacgcactgagaagcccttagagcctctcaaagcaattttgagtgacacaggaacacttaacggctgacatgggaattcccctccaccgcggtggCCCTgtacaaagaaaattcgacaaactgttatttttctatctatttatttgAATTttgAGCGCTcaaAATTtacctttgtcggcAATTttgAGCGCTcaaAATTaaataaagatattctcgtcaaacaaatataaataatataaacatggtttttactctggaagattttgttggcgattggcgtcagaccgcgggttataatttggatcaagtcctggaacagggtggcgtaagctctctgttccagaacctgggtgtgagcgtgacgccgattcagcgcatcgttctgtccggcgagaacggtctgaaaattgatattcatgtgatcatcccgtacgaaggcctgagcggtgaccaaatgggtcaaatcgagaaaatctttaaagtcgtctacccagttgacgatcaccacttcaaggttatcttgcattacggtacgctggtgattgatggtgtgaccccgaatatgattgactatttcggccgtccgtatgaaggcattgccgtttttgacggtaaaaagatcaccgtcaccggtaccctgtggaatggcaataagattattgacgagcgtctgattaacccggacggcagcctgctgttccgcgtgaccatcaacggtgtcacgggttggcgtctgtgcgagcgcatcctggcataatgaactgcacttgctttgataattaatgataaacaatctaaaagcactctaatcgttatcggagtgcttttagattactaatcaaattgcttctactaattgcctatcttccagtgatggaacagcatttgAACGcattggctgcaacaatcagccttgatctggaagaagcaatgaaagctgctgttaagtctccgaatcaggtattgttcctgacaggtgtattcccatccggtaaacgcggatactttgcagttgatctgactcaggaataaattataaattaaggtaagaagattgtaggataagctaatgaaatagaaaaaggatgccgtcacacaacttgtcggcattcttttttgttttattagttgaaaatatagtgaaaaagttgcctaaatatgtatgttaacaaattatttgtcgtaactttgcactccaaatctgtttttaacatatggcactagtgAAACCAGTAACGTTATACGATGTCGCAGAGTATGCCGGTGTCTCTTATCAGACCGTTTCCCGCGTGGTGAACCAGGCCAGCCACGTTTCTGCGAAAACGCGGGAAAAAGTGGAAGCGGCGATGGCGGAGCTGAATTACATTCCCAACCGCGTGGCACAACAACTGGCGGGCAAACAGTCGTTGCTGATTGGCGTTGCCACCTCCAGTCTGGCCCTGCACGCGCCGTCGCAAATTGTCGCGGCGATTAAATCTCGCGCCGATCAACTGGGTGCCAGCGTGGTGGTGTCGATGGTAGAACGAAGCGGCGTCGAAGCCTGTAAAACGGCGGTGCACAATCTTCTCGCGCAACGCGTCAGTGGGCTGATCATTAACTATCCGCTGGATGACCAGGATGCCATTGCTGTGGAAGCTGCCTGCACTAATGTTCCGGCGTTATTTCTTGATGTCTCTGACCAGACACCCATCAACAGTATTATTTTCTCCCATGAAGACGGTACGCGACTGGGCGTGGAGCATCTGGTCGCATTGGGTCACCAGCAAATCGCGCTGTTAGCGGGCCCATTAAGTTCTGTCTCGGCGCGTCTGCGTCTGGCTGGCTGGCATAAATATCTCACTCGCAATCAAATTCAGCCGATAGCGGAACGGGAAGGCGACTGGAGTGCCATGTCCGGTTTTCAACAAACCATGCAAATGCTGAATGAGGGCATCGTTCCCACTGCGATGCTGGTTGCCAACGATCAGATGGCGCTGGGCGCAATGCGCGCCATTACCGAGTCCGGGCTGCGCGTTGGTGCGGATATCTCGGTAGTGGGATACGACGATACCGAAGACAGCTCATGTTATATCCCGCCGTTAACCACCATCAAACAGGATTTTCGCCTGCTGGGGCAAACCAGCGTGGACCGCTTGCTGCAACTCTCTCAGGGCCAGGCGGTGAAGGGCAATCAGCTGTTGCCCGTCTCACTGGTGAAAAGAAAAACCACCCTGGCGCCCAATACGCAAACCGCCTCTCCCCGCGCGTTGGCCGATTCATTAATGCAGCTGGCACGACAGGTTTCCCGACTGGAAAGCGGGCAGtgagctttcctcggtaccaaattccagaaaagaggcctcccgaaaggggggccttttttcgttttggtcctacttgtgcctgttctatttccTATGcggttcctggccttttgctggccttttgctcacatgttctttcctgcgttatcccctgattctgtggataaccgtattaccgcctttgagtgagctgatacATCCcaaatgctgttccatcactggaagataggcaattagtagaagcaatttgattagtaatctaaaagcactccgataacgattagagtgcttttagattgtttatcattaattatcaaagcaagtgcagttcaTCACTGCCCGCTTTCCAGTCGGGAAACCTGTCGTGCCAGCTGCATTAATGAATCGGCCAACGCGCGGGGAGAGGCGGTTTGCGTATTGGGCGCCAGGGTGGTTTTTCTTTTCACCAGCTAAGCCGAACCGCGTTCCATCAGAATCGGAGTAAGTTGTAATCGTTGTTGCTGAAGGGTCGGCTGGGTTATCCGATGGATGAGTACATCAATCGCCAGCTCCCCCAGTTCATCTTTCGGTTGGTGGATAGTGGTTAATGGTGGCGTCATAAAGCTTGCCAGTTCGATATCGTCATAGCCAATCACCGCGATATCCTGCGGAACCTGTAACTCTGCCTGATATAACGCCTGGTAAACGCCAACAGCCATAGCGTCATTTCCGGTAAAGACGGCCTGAGGACGCAGCGGATGTGATAGCAGTTGGCGCATAGCGTCAAACCCGCCGTTAAATTCAAAATCACCAGTGACTTCATAGCCATCAGGAATGTTGAGACCCGCACGTTTCATCGCCGCCCGATAACCTTCCAACCGCAGGCGCGCCGGAGTTTTATCCAGCGGGCCGGTAATACAGGCGATACGGGTATGACCTTTATCGATCAGATATTGCGTTGCTAAGTCTCCGCCCAGCAACGAGTTATCCTGAATAAGATCGCTGTCGCCATCGAACGGAGCCCAGTCCATCATCACAGTAGGCACTGTCGGATAACGTTGCATGATTTCACGCGAAGGTTGATGCGTTTCGGTGCACAGTAACAGCAAGCCATCAACGCGTTTTTGCATCAGCGTTTCCAGATTGCGATTCATCCGCTGTTCATCGCCTTCGGTATTGCAAAGGACGAGACTATAACCGCGTTCGAAGCAGCTGCGTTCAACGCCACGCACCAGTTCTGAATAGAAAGGATTGGTACTGGCAGTGATCAACATGCCAATGGTATGCGACGCTTTGCCCGCCAGTTGTTGTGCCACGCGGTTGGGAATGTAATTGAGCTCCGCCATCGCCGCTTCCACTTTTTCCCGCGTTTTCGCAGAAACGTGGCTGGCCTGGTTCACCACattGGAAACGGTctgatgAGAGACACCGGCATACTCTGCGACATCGTATAACGTTACTGGTTTcacgtttatattatttatatttgtttgacgagaatatctttatttaattgttatccgctcacaattgccgacaaaggtaattgttatccgctcacaattcaaataaatagatagaaaaataacagtttgtcgaattttctttgtacCCGAtacttgtgcctgttctatttccgaaccgaccgcttgtatgaatccatcaaaattcgttttctctatgttggattccttgttgctcatattgtgatgataatttctacaaatatagtcattggtaactatctatgaaactgtttgatacttttatagttgattaaacttgttcatggcatttgccttaatatcatccgctatgtcaatgtagggtttcatagctttgtagtcgctgtgtcccgtccatttcatgaccacctgtgccgggattccgagagccagcgcattgcagatgaatgtccttcttcctgcatgggtactgagcaaagcgtatttgggtgtgacttcatcaatacgttcatttcccttgtagtaggtttcccgtacaggctcgttgatttctgccagttcgcccagctctttcaggtaatcgttcatcttctggttgctgatgacgggcagagccatgtaattctcgaaatggatgtccttgtatttgtccagtatggctttgctgtatttgttcagttcaatcgtcaggctgtcggcagtcttgactgtggttatttcgatgtggtcggacttcacatcgcttcttttcagattgcgaacatccgaataccgcaaactcgtaaagcagcagaacaggaaaacatcacgcacacgttccaggtattgcttatccttgggtatctggtagtctttcagcttgttcagttcatcccaagtcaggaagattacttttttcgaggtggttttcagtttcggtttgaacgtatcgtatgcaatgttctgatgatgtcctttcctgaagctccagcgcaggaaccatttgaggaatcccatttgcttgccgatggtgctgtttctcatatccttggtgtcacgcaggaagttgacgtattcgttcaatccaaactcgttgaaatagttgaacgttgcatcctccttgaactctttgaggtggttcctcactgctgcaaatttttcataggtggatgccgtccagttattctggttaccgcactcttttacaaactcatcgaacacctcccaaaagctgacaggggcttcttccggctgttcttcactggtgtctttcattctcatgttgaaagcttccttcaactgttgggtcgttggcatgacctcctgcacctcaaattccttgaaaatattctggatttcggcatagtatttcagcaagtccgtattgatttcggctgcactttgctttagcttgttggtacatccgttctttacccgctgcttatctacatcccatttggctacgtcaatccggtagcccgttgtaaactcgatacgttggctggcaaagatgacacgcatacggatgggtacgttctctacgattggcacaccgttctttttccggctctccaatgcaaaaatgatgttgcgcttgatattcataattgggtgcgtttgaaattctacacccaaatatacacccaattattgagatagcaaaagacatttagaaacatttacttttactctatattgtaatttacacttgattatcagtcgtttgcagtTttatgatattctgtgaaagtataagttcgagagcctgtctctccgcaaaaaacgctgaaaatcagcagattgcaaaacaaacaccctgttttacacccaagaatgtaaagtcggctgtttttgttttatttaagataatacaaccactacataataaaagagtagcgatattaaaagaatccgatgagaaaagactaatatttatctatccattcagtttgatttttcaggactttacatcgtcctgaaagtatttgttggtaccggtaccgaggacgcgtaaacatttacagttgcatgtggcctattgtttttagccgttaaatattttataactattaaatagcgatacaaattgttcgaaactaatattgtttatatcatatattctcgcatgttttaaagctttattaaattgattttttgtaaacagtttttcgtactctttgttaacccatttcattacaaaagtttcatatttttttctctctttaaatgccatttttgctggctttctttttaatacaattaatgtgctatccactttaggttttggatggaaataatacctaggaatttttgctaatatagaaatatctacctctgccattaacagcaatgctagtgatctgtttgtatctaataacattttagcaaaaccatattccactattaaataacttattgtggctgaactttcaaaaacaatttttcgaattatatttgtgcttatgttgtaaggtatgctgccaaatattttatatggattgtggctaggaaatgtaaatttcagtatatcatcatttactatttgatagttaggataatttaagagcttattacgagttacctcacataatttagaatcaatttctatcgccgttacaaaattacatctctttaccaatccagcagtaaaatgacctttccctgcacctatttcaaagatgttatctttttcatctaaacttatgcaattcattattttttctatgtgatattttgaagtaataaaattttgactatcttttatatttactttgttcattataacctctccttaatttattgcatctcttttcgaatatttatgttttttgagaaaagaacgtactcatggttcatcccgatatgcgtatcggtctgtatatcagcaactttctatgtgtttcaactacaatagtcatctattctcatctttctgagtccaccccctgcaaagcccctctttacgacataaaaattcggtcggaaaaggtatgcaaaagatgtttctctctttaagagaaactcttcgggatgcaaaaatatgaaaataactccaattcaccaaattatatagcgacttttttacaaaatgctaaaatttgttgatttccgtcaagcaattgttgagcaaaaatgtcttttacgataaaatgatacctcaatatcaactgtttagcaaaacgatatttctcttaaagagagaaacacctttttgttcaccaatccccgacttttaatcccgcggccatgattgaaaaaggaagagtatgagtattcaacatttccgtgtcgcccttattcccttttttgcggcattttgccttcctgtttttgctcacccagaaacgctggtgaaagtaaaagatgctgaagatcagttgggtgcacgagtgggttacatcgaactggatctcaacagcggtaagatccttgagagttttcgccccgaagaacgttttccaatgatgagcacttttaaagttctgctatgtggcgcggtattatcccgtattgacgccgggcaagagcaactcggtcgccgcatacactattctcagaatgacttggttgagtactcaccagtcacagaaaagcatcttacggatggcatgacagtaagagaattatgcagtgctgccataaccatgagtgataacactgcggccaacttacttctgacaacgatcggaggaccgaaggagctaaccgcttttttgcacaacatgggggatcatgtaactcgccttgatcgttgggaaccggagctgaatgaagccataccaaacgacgagcgtgacaccacgatgcctgtagcaatggcaacaacgttgcgcaaactattaactggcgaactacttactctagcttcccggcaacaattaatagactggatggaggcggataaagttgcaggaccacttctgcgctcggcccttccggctggctggtttattgctgataaatctggagccggtgagcgtgggtctcgcggtatcattgcagcactggggccagatggtaagccctcccgtatcgtagttatctacacgacggggagtcaggcaactatggatgaacgaaatagacagatcgctgagataggtgcctcactgattaagcattggtaactgtcagaccaagtttactcataacgcgtcaattcgagggggatcaattccgtgataggtgggctgcccttcctggttggcttggtttcatcagccatccgcttgccctcatctgttacgccggcggtagccggccagcctcgcagagcaggattcccgttgagcaccgccaggtgcgaataagggacagtgaagaaggaacacccgctcgcgggtgggcctacttcacctatcctgcccggctgacgccgttggatacaccaaggaaagtctacacgaaccctttggcaaaatcctgtatatcgtgcgaaaaaggatggatataccgaaaaaatcgctataatgaccccgaagcagggttatgcagcggaaaacggaattgatccggccacgatgcgtccggcgtagaggatctgaagatcagcagttcaacctgttgatagtacgtactaagctctcatgtttcacgtactaagctctcatgtttaacgtactaagctctcatgtttaacgaactaaaccctcatggctaacgtactaagctctcatggctaacgtactaagctctcatgtttcacgtactaagctctcatgtttgaacaataaaattaatataaatcagcaacttaaatagcctctaaggttttaagttttataagaaaaaaaagaatatataaggcttttaaagcttttaag

>pBH304

gtttaacggttgtggacaacaagccagggatgtaacgcactgagaagcccttagagcctctcaaagcaattttgagtgacacaggaacacttaacggctgacatgggaattcccctccaccgcggtggCCCTgtacaaagaaaattcgacaaactgttatttttctatctatttatttgAATTttgAGCGCTcaaAATTtacctttgtcggcAATTttgAGCGCTcaaAATTaaataaagatattctcgtcaaacaaatataaataatataaacatggtttttactctggaagattttgttggcgattggcgtcagaccgcgggttataatttggatcaagtcctggaacagggtggcgtaagctctctgttccagaacctgggtgtgagcgtgacgccgattcagcgcatcgttctgtccggcgagaacggtctgaaaattgatattcatgtgatcatcccgtacgaaggcctgagcggtgaccaaatgggtcaaatcgagaaaatctttaaagtcgtctacccagttgacgatcaccacttcaaggttatcttgcattacggtacgctggtgattgatggtgtgaccccgaatatgattgactatttcggccgtccgtatgaaggcattgccgtttttgacggtaaaaagatcaccgtcaccggtaccctgtggaatggcaataagattattgacgagcgtctgattaacccggacggcagcctgctgttccgcgtgaccatcaacggtgtcacgggttggcgtctgtgcgagcgcatcctggcataatgaactgcacttgctttgataattaatgataaacaatctaaaagcactctaatcgttatcggagtgcttttagattactaatcaaattgcttctactaattgcctatcttccagtgatggaacagcatttgAACGcattggctgcaacaatcagccttgatctggaagaagcaatgaaagctgctgttaagtctccgaatcaggtattgttcctgacaggtgtattcccatccggtaaacgcggatactttgcagttgatctgactcaggaataaattataaattaaggtaagaagattgtaggataagctaatgaaatagaaaaaggatgccgtcacacaacttgtcggcattcttttttgttttattagttgaaaatatagtgaaaaagttgcctaaatatgtatgttaacaaattatttgtcgtaactttgcactccaaatctgtttttaacatatggcactagtgAAACCAGTAACGTTATACGATGTCGCAGAGTATGCCGGTGTCTCTACCGCGACCGTTTCCAACGTGGTGAACCAGGCCAGCCACGTTTCTGCGAAAACGCGGGAAAAAGTGGAAGCGGCGATGGCGGAGCTCAATTACATTCCCAACCGCGTGGCACAACAACTGGCGGGCAAAGCGTCGCATACCATTGGCATGTTGATCACTGCCAGTACCAATCCTTTCTATTCAGAACTGGTGCGTGGCGTTGAACGCAGCTGCTTCGAACGCGGTTATAGTCTCGTCCTTTGCAATACCGAAGGCGATGAACAGCGGATGAATCGCAATCTGGAAACGCTGATGCAAAAACGCGTTGATGGCTTGCTGTTACTGTGCACCGAAACGCATCAACCTTCGCGTGAAATCATGCAACGTTATCCGACAGTGCCTACTGTGATGATGGACTGGGCTCCGTTCGATGGCGACAGCGATCTTATTCAGGATAACTCGTTGCTGGGCGGAGACTTAGCAACGCAATATCTGATCGATAAAGGTCATACCCGTATCGCCTGTATTACCGGCCCGCTGGATAAAACTCCGGCGCGCCTGCGGTTGGAAGGTTATCGGGCGGCGATGAAACGTGCGGGTCTCAACATTCCTGATGGCTATGAAGTCACTGGTGATTTTGAATTTAACGGCGGGTTTGACGCTATGCGCCAACTGCTATCACATCCGCTGCGTCCTCAGGCCGTCTTTACCGGAAATGACGCTATGGCTGTTGGCGTTTACCAGGCGTTATATCAGGCAGAGTTACAGGTTCCGCAGGATATCGCGGTGATTGGCTATGACGATATCGAACTGGCAAGCTTTATGACGCCACCATTAACCACTATCCACCAACCGAAAGATGAACTGGGGGAGCTGGCGATTGATGTACTCATCCATCGGATAACCCAGCCGACCCTTCAGCAACAACGATTACAACTTACTCCGATTCTGATGGAACGCGGTTCGGCTTAGCTGGTGAAAAGAAAAACCACCCTGGCGCCCAATACGCAAACCGCCTCTCCCCGCGCGTTGGCCGATTCATTAATGCAGCTGGCACGACAGGTTTCCCGACTGGAAAGCGGGCAGTGAgctttcctcggtaccaaattccagaaaagaggcctcccgaaaggggggccttttttcgttttggtcctacttgtgcctgttctatttccTATGcggttcctggccttttgctggccttttgctcacatgttctttcctgcgttatcccctgattctgtggataaccgtattaccgcctttgagtgagctgatacATCCcaaatgctgttccatcactggaagataggcaattagtagaagcaatttgattagtaatctaaaagcactccgataacgattagagtgcttttagattgtttatcattaattatcaaagcaagtgcagttcatcaCTGCCCGCTTTCCAGTCGGGAAACCTGTCGTGCCAGCTGCATTAATGAATCGGCCAACGCGCGGGGAGAGGCGGTTTGCGTATTGGGCGCCAGGGTGGTTTTTCTTTTCACCAGTGAGACGGGCAACAGCTGATTGCCCTTCACCGCCTGGCCCTGAGAGAGTTGCAGCAAGCGGTCCACGCTGGTTTGCCCCAGCAGGCGAAAATCCTGTTTGATGGTGGTTAACGGCGGGATATAACATGAGCTGTCTTCGGTATCGTCGTATCCCACTACCGAGATATCCGCACCAACGCGCAGCCCGGACTCGGTAATGGCGCGCATTGCGCCCAGCGCCATCTGATCGTTGGCAACCAGCATCGCAGTGGGAACGATGCCCTCATTCAGCATTTGCATGGTTTGTTGAAAACCGGACATGGCACTCCAGTCGCCTTCCCGTTCCGCTATCGGCTGAATTTGATTGCGAGTGAGATATTTATGCCAGCCAGCCAGACGCAGACGCGCCGAGACAGAACTTAATGGGCCCGCTAACAGCGCGATTTGCTGGTGACCCAATGCGACCAGATGCTCCACGCCCAGTCGCGTACCGTCTTCATGGGAGAAAATAATACTGTTGATGGGTGTCTGGTCAGAGACATCAAGAAATAACGCCGGAACATTAGTGCAGGCAGCTTCCACAGCAATGGCATCCTGGTCATCCAGCGGATAGTTAATGATCAGCCCACTGACGCGTTGCGCGAGAAGATTGTGCACCGCCGTTTTACAGGCTTCGACGCCGCTTCGTTCTACCATCGACACCACCACGCTGGCACCCAGTTGATCGGCGCGAGATTTAATCGCCGCGACAATTTGCGACGGCGCGTGCAGGGCCAGACTGGAGGTGGCAACGCCAATCAGCAACGACTGTTTGCCCGCCAGTTGTTGTGCCACGCGGTTGGGAATGTAATTCAGCTCCGCCATCGCCGCTTCCACTTTTTCCCGCGTTTTCGCAGAAACGTGGCTGGCCTGGTTCACCACGCGGGAAACGGTCTGATAAGAGACACCGGCATACTCTGCGACATCGTATAACGTTACTGGTTTcacgtttatattatttatatttgtttgacgagaatatctttatttaattTTAAGCGCTTAAaattgccgacaaaggtaaattTTAAGCGCTTAAaattcaaataaatagatagaaaaataacagtttgtcgaattttctttgtacCCGAtacttgtgcctgttctatttccgaaccgaccgcttgtatgaatccatcaaaattcgttttctctatgttggattccttgttgctcatattgtgatgataatttctacaaatatagtcattggtaactatctatgaaactgtttgatacttttatagttgattaaacttgttcatggcatttgccttaatatcatccgctatgtcaatgtagggtttcatagctttgtagtcgctgtgtcccgtccatttcatgaccacctgtgccgggattccgagagccagcgcattgcagatgaatgtccttcttcctgcatgggtactgagcaaagcgtatttgggtgtgacttcatcaatacgttcatttcccttgtagtaggtttcccgtacaggctcgttgatttctgccagttcgcccagctctttcaggtaatcgttcatcttctggttgctgatgacgggcagagccatgtaattctcgaaatggatgtccttgtatttgtccagtatggctttgctgtatttgttcagttcaatcgtcaggctgtcggcagtcttgactgtggttatttcgatgtggtcggacttcacatcgcttcttttcagattgcgaacatccgaataccgcaaactcgtaaagcagcagaacaggaaaacatcacgcacacgttccaggtattgcttatccttgggtatctggtagtctttcagcttgttcagttcatcccaagtcaggaagattacttttttcgaggtggttttcagtttcggtttgaacgtatcgtatgcaatgttctgatgatgtcctttcctgaagctccagcgcaggaaccatttgaggaatcccatttgcttgccgatggtgctgtttctcatatccttggtgtcacgcaggaagttgacgtattcgttcaatccaaactcgttgaaatagttgaacgttgcatcctccttgaactctttgaggtggttcctcactgctgcaaatttttcataggtggatgccgtccagttattctggttaccgcactcttttacaaactcatcgaacacctcccaaaagctgacaggggcttcttccggctgttcttcactggtgtctttcattctcatgttgaaagcttccttcaactgttgggtcgttggcatgacctcctgcacctcaaattccttgaaaatattctggatttcggcatagtatttcagcaagtccgtattgatttcggctgcactttgctttagcttgttggtacatccgttctttacccgctgcttatctacatcccatttggctacgtcaatccggtagcccgttgtaaactcgatacgttggctggcaaagatgacacgcatacggatgggtacgttctctacgattggcacaccgttctttttccggctctccaatgcaaaaatgatgttgcgcttgatattcataattgggtgcgtttgaaattctacacccaaatatacacccaattattgagatagcaaaagacatttagaaacatttacttttactctatattgtaatttacacttgattatcagtcgtttgcagtTttatgatattctgtgaaagtataagttcgagagcctgtctctccgcaaaaaacgctgaaaatcagcagattgcaaaacaaacaccctgttttacacccaagaatgtaaagtcggctgtttttgttttatttaagataatacaaccactacataataaaagagtagcgatattaaaagaatccgatgagaaaagactaatatttatctatccattcagtttgatttttcaggactttacatcgtcctgaaagtatttgttggtaccggtaccgaggacgcgtaaacatttacagttgcatgtggcctattgtttttagccgttaaatattttataactattaaatagcgatacaaattgttcgaaactaatattgtttatatcatatattctcgcatgttttaaagctttattaaattgattttttgtaaacagtttttcgtactctttgttaacccatttcattacaaaagtttcatatttttttctctctttaaatgccatttttgctggctttctttttaatacaattaatgtgctatccactttaggttttggatggaaataatacctaggaatttttgctaatatagaaatatctacctctgccattaacagcaatgctagtgatctgtttgtatctaataacattttagcaaaaccatattccactattaaataacttattgtggctgaactttcaaaaacaatttttcgaattatatttgtgcttatgttgtaaggtatgctgccaaatattttatatggattgtggctaggaaatgtaaatttcagtatatcatcatttactatttgatagttaggataatttaagagcttattacgagttacctcacataatttagaatcaatttctatcgccgttacaaaattacatctctttaccaatccagcagtaaaatgacctttccctgcacctatttcaaagatgttatctttttcatctaaacttatgcaattcattattttttctatgtgatattttgaagtaataaaattttgactatcttttatatttactttgttcattataacctctccttaatttattgcatctcttttcgaatatttatgttttttgagaaaagaacgtactcatggttcatcccgatatgcgtatcggtctgtatatcagcaactttctatgtgtttcaactacaatagtcatctattctcatctttctgagtccaccccctgcaaagcccctctttacgacataaaaattcggtcggaaaaggtatgcaaaagatgtttctctctttaagagaaactcttcgggatgcaaaaatatgaaaataactccaattcaccaaattatatagcgacttttttacaaaatgctaaaatttgttgatttccgtcaagcaattgttgagcaaaaatgtcttttacgataaaatgatacctcaatatcaactgtttagcaaaacgatatttctcttaaagagagaaacacctttttgttcaccaatccccgacttttaatcccgcggccatgattgaaaaaggaagagtatgagtattcaacatttccgtgtcgcccttattcccttttttgcggcattttgccttcctgtttttgctcacccagaaacgctggtgaaagtaaaagatgctgaagatcagttgggtgcacgagtgggttacatcgaactggatctcaacagcggtaagatccttgagagttttcgccccgaagaacgttttccaatgatgagcacttttaaagttctgctatgtggcgcggtattatcccgtattgacgccgggcaagagcaactcggtcgccgcatacactattctcagaatgacttggttgagtactcaccagtcacagaaaagcatcttacggatggcatgacagtaagagaattatgcagtgctgccataaccatgagtgataacactgcggccaacttacttctgacaacgatcggaggaccgaaggagctaaccgcttttttgcacaacatgggggatcatgtaactcgccttgatcgttgggaaccggagctgaatgaagccataccaaacgacgagcgtgacaccacgatgcctgtagcaatggcaacaacgttgcgcaaactattaactggcgaactacttactctagcttcccggcaacaattaatagactggatggaggcggataaagttgcaggaccacttctgcgctcggcccttccggctggctggtttattgctgataaatctggagccggtgagcgtgggtctcgcggtatcattgcagcactggggccagatggtaagccctcccgtatcgtagttatctacacgacggggagtcaggcaactatggatgaacgaaatagacagatcgctgagataggtgcctcactgattaagcattggtaactgtcagaccaagtttactcataacgcgtcaattcgagggggatcaattccgtgataggtgggctgcccttcctggttggcttggtttcatcagccatccgcttgccctcatctgttacgccggcggtagccggccagcctcgcagagcaggattcccgttgagcaccgccaggtgcgaataagggacagtgaagaaggaacacccgctcgcgggtgggcctacttcacctatcctgcccggctgacgccgttggatacaccaaggaaagtctacacgaaccctttggcaaaatcctgtatatcgtgcgaaaaaggatggatataccgaaaaaatcgctataatgaccccgaagcagggttatgcagcggaaaacggaattgatccggccacgatgcgtccggcgtagaggatctgaagatcagcagttcaacctgttgatagtacgtactaagctctcatgtttcacgtactaagctctcatgtttaacgtactaagctctcatgtttaacgaactaaaccctcatggctaacgtactaagctctcatggctaacgtactaagctctcatgtttcacgtactaagctctcatgtttgaacaataaaattaatataaatcagcaacttaaatagcctctaaggttttaagttttataagaaaaaaaagaatatataaggcttttaaagcttttaag

>pBH305

gatgtaacgcactgagaagcccttagagcctctcaaagcaattttgagtgacacaggaacacttaacggctgacatgggaattcccctccaccgcggtgtattaatgcggctgcCCCTcggttcctggccttttgctggccttttgctcacatgttctttcctgcgttatcccctgattctgtggataaccgtattaccgcctttgagtgagctgatacAACGcattggctgcaacaatcagccttgatctggaagaagcaatgaaagctgctgttaagtctccgaatcaggtattgttcctgacaggtgtattcccatccggtaaacgcggatactttgcagttgatctgactcaggaataaattataaattaaggtaagaagattgtaggataagctaatgaaatagaaaaaggatgccgtcacacaacttgtcggcattcttttttgttttattagttgaaaatatagtgaaaaagttgcctaaatatgtatgttaacaaattatttgtcgtaactttgcactccaaatctgtttttaacatatggcactagtgAAACCAGTAACGTTATACGATGTCGCAGAGTATGCCGGTGTCTCTTATCAGACCGTTTCCCGCGTGGTGAACCAGGCCAGCCACGTTTCTGCGAAAACGCGGGAAAAAGTGGAAGCGGCGATGGCGGAGCTGAATTACATTCCCAACCGCGTGGCACAACAACTGGCGGGCAAACAGTCGTTGCTGATTGGCGTTGCCACCTCCAGTCTGGCCCTGCACGCGCCGTCGCAAATTGTCGCGGCGATTAAATCTCGCGCCGATCAACTGGGTGCCAGCGTGGTGGTGTCGATGGTAGAACGAAGCGGCGTCGAAGCCTGTAAAACGGCGGTGCACAATCTTCTCGCGCAACGCGTCAGTGGGCTGATCATTAACTATCCGCTGGATGACCAGGATGCCATTGCTGTGGAAGCTGCCTGCACTAATGTTCCGGCGTTATTTCTTGATGTCTCTGACCAGACACCCATCAACAGTATTATTTTCTCCCATGAAGACGGTACGCGACTGGGCGTGGAGCATCTGGTCGCATTGGGTCACCAGCAAATCGCGCTGTTAGCGGGCCCATTAAGTTCTGTCTCGGCGCGTCTGCGTCTGGCTGGCTGGCATAAATATCTCACTCGCAATCAAATTCAGCCGATAGCGGAACGGGAAGGCGACTGGAGTGCCATGTCCGGTTTTCAACAAACCATGCAAATGCTGAATGAGGGCATCGTTCCCACTGCGATGCTGGTTGCCAACGATCAGATGGCGCTGGGCGCAATGCGCGCCATTACCGAGTCCGGGCTGCGCGTTGGTGCGGATATCTCGGTAGTGGGATACGACGATACCGAAGACAGCTCATGTTATATCCCGCCGTTAACCACCATCAAACAGGATTTTCGCCTGCTGGGGCAAACCAGCGTGGACCGCTTGCTGCAACTCTCTCAGGGCCAGGCGGTGAAGGGCAATCAGCTGTTGCCCGTCTCACTGGTGAAAAGAAAAACCACCCTGGCGCCCAATACGCAAACCGCCTCTCCCCGCGCGTTGGCCGATTCATTAATGCAGCTGGCACGACAGGTTTCCCGACTGGAAAGCGGGCAGtgagctttcctcggtaccaaattccagaaaagaggcctcccgaaaggggggccttttttcgttttggtcctacttgtgcctgttctatttccTATGcggttcctggccttttgctggccttttgctcacatgttctttcctgcgttatcccctgattctgtggataaccgtattaccgcctttgagtgagctgatacATCCcaaatgctgttccatcactggaagataggcaattagtagaagcaatttgattagtaatctaaaagcactccgataacgattagagtgcttttagattgtttatcattaattatcaaagcaagtgcagttcaTCACTGCCCGCTTTCCAGTCGGGAAACCTGTCGTGCCAGCTGCATTAATGAATCGGCCAACGCGCGGGGAGAGGCGGTTTGCGTATTGGGCGCCAGGGTGGTTTTTCTTTTCACCAGCTAAGCCGAACCGCGTTCCATCAGAATCGGAGTAAGTTGTAATCGTTGTTGCTGAAGGGTCGGCTGGGTTATCCGATGGATGAGTACATCAATCGCCAGCTCCCCCAGTTCATCTTTCGGTTGGTGGATAGTGGTTAATGGTGGCGTCATAAAGCTTGCCAGTTCGATATCGTCATAGCCAATCACCGCGATATCCTGCGGAACCTGTAACTCTGCCTGATATAACGCCTGGTAAACGCCAACAGCCATAGCGTCATTTCCGGTAAAGACGGCCTGAGGACGCAGCGGATGTGATAGCAGTTGGCGCATAGCGTCAAACCCGCCGTTAAATTCAAAATCACCAGTGACTTCATAGCCATCAGGAATGTTGAGACCCGCACGTTTCATCGCCGCCCGATAACCTTCCAACCGCAGGCGCGCCGGAGTTTTATCCAGCGGGCCGGTAATACAGGCGATACGGGTATGACCTTTATCGATCAGATATTGCGTTGCTAAGTCTCCGCCCAGCAACGAGTTATCCTGAATAAGATCGCTGTCGCCATCGAACGGAGCCCAGTCCATCATCACAGTAGGCACTGTCGGATAACGTTGCATGATTTCACGCGAAGGTTGATGCGTTTCGGTGCACAGTAACAGCAAGCCATCAACGCGTTTTTGCATCAGCGTTTCCAGATTGCGATTCATCCGCTGTTCATCGCCTTCGGTATTGCAAAGGACGAGACTATAACCGCGTTCGAAGCAGCTGCGTTCAACGCCACGCACCAGTTCTGAATAGAAAGGATTGGTACTGGCAGTGATCAACATGCCAATGGTATGCGACGCTTTGCCCGCCAGTTGTTGTGCCACGCGGTTGGGAATGTAATTGAGCTCCGCCATCGCCGCTTCCACTTTTTCCCGCGTTTTCGCAGAAACGTGGCTGGCCTGGTTCACCACattGGAAACGGTctgatgAGAGACACCGGCATACTCTGCGACATCGTATAACGTTACTGGTTTcacgtttatattatttatatttgtttgacgagaatatctttatttaattgttatccgctcacaattgccgacaaaggtaattgttatccgctcacaattcaaataaatagatagaaaaataacagtttgtcgaattttctttgtacCCGAtaattgcctatcttccagtgatggaacagcatttgtgcattggctgcaacaatcagccttacttgtgcctgttctatttccgaaccgaccgcttgtatgaatGcatcaaaattcgttttctctaCgttggattccttgttgctcatattgtgatgataatttctacaaatatagtcattggtaactatctatgaaactgtttgatacttttatcagtctaatagttttacaaggtctttcttcatttcttcgtcaatatccctgtatcgtctgaaagctttgcttccctccttgtgtcccgacagtgcggaaacaaggttcgggtctttcacttttttatagatattgccgataaacgtacgtcttgccagatggctgcttgccacttcataaataggtcgtttgatttcgttgtgcgtcaacgggtctaagattgttacgatgcggtcaactccagctaatttgaatatcttttttatggcatcattgtacttttgctcggatatgaacggcaacagttttccctcatattctttgtagcgttcaaggatttctttcgctttgtcgttaagtggaacacgtaccgtaaccggattcccctctttggttttcttgggaatatattctatggcttcattgaccacatttagtttggtcattcggtacaggtcgctcaccctgcatcctatcagtgtctgaaatatgaatatatccctctgtattgccagttgtggggtggcagaaaggtctgcattaaaaatcctgtccctttcttcgagtgttatataataaggtgtaccatatgtacactcctctatcggaaacttgtcgaaaggtctgtttgtggtgcgtttgttatcgaagcaccacaggaagaatgtgcgtattcttgaaaaacagtctatcagcgtgtttttgcttctgggctgtggtgtcctcttttcgggaatggcttcataaatgctcgggtaaagttcataatactggtattcgttctgaaagaaatcccacatatcccgaagcgtgtcaggtgttaccaaatccacatcaaggataaagcccttttgtcctctctttgtagcccttacatatagttcataacgcagtaaggctcttttgacaacccggaaatttttctttcgtacttccgacaaagggtgcttgtttagaaattcatcgaatagttctccaatggtaggcttgataaccacttcctccggcaaaaaatatttttcaggatggtaaaatttatcaagtgttgtttttagccattctttgtctattgcttccttttcttgttgatataccttctcgatataggttttcaactggcgtatctcttcatttatatgggtacgcatttcttcattgcagacagctttcgtttttacacattcgtctttatcatcccatagattgggattgatggctaactgagtaggagcaacggagtctaactgccttccgtttctaaaacggacatagattgtagccatggattctgtatcatatcgtttggctgcttttttaatgataaaggttactttcatagactttcaggttgaattttactctgctgcaaatataaatattttccccagcattttccccacatctgctaaatattttgcaattcgattaaacttggattaaaatttaataggattataacatattgaaatacagtgtaattgtggcgtttttccgcatttttctttttacccacattttccccactttaattatttcaaatgcggggtctgggtacaagaaagaaagctaagtatttgatagttcaatacttagctttttcttttgcttgaattttccccacattttccccacacgtgcaaaaaatatagcagtaagtcattatttcttttggttgaacgtagagagtagcgatattaaaagaatccgatgagaaaagactaatatttatctatccattcagtttgatttttcaggactttacatcgtcctgaaagtatttgttggtaccggtaccgaggacgcgtaaacatttacaGTTGCATGTGGCCTATTGTTTaggacgcgttatctccttaacgtacgttttcgttccattggccctcaaaccccgttatatacattcatgtccatttatgtaaaaaatcctgctgaccttgtttatgtcttgtcagtcaccatttgcaaaaccatatttgaccctcaaagaggctgaatttgataagcaacttgctacatactcataataaggagctaaatagaacacgaatgggaaatactcaaatgccaaactaaagaagatattggccaaaataaacgctataccgagagagaaacttgatttttcaacttcctaaccaacagtgttgttcaaacatttctacttatttgtacttaccagttgaacctacgtttccctaataaaatgtctatggtaaaaagttaaaaaatcctcctacttttgttagatatatttttttgtgtaattttgtaatcgttatgcggcagtaataatatacatattaatacgagttaggaatcctgtagttctcatatgctacgaggaggtattaaaaggtgcgtttcgacaatgcatctattgtagtatattattgcttaatccaaatgaatattataaatttaggaattcttgctcacattgatgcaggaaaaacttccgtaaccgagaatctgctgtttgccagtggagcaacggaaaagtgcggctgtgtggataatggtgacaccataacggactctatggatatagagaaacgtagaggaattactgttcgggcttctacgacatctattatctggaatggtgtgaaatgcaatatcattgacactccgggacacatggattttattgcggaagtggagcggacattcaaaatgcttgatggagcagtcctcatcttatccgcaaaggaaggcatacaagcgcagacaaagttgctgttcaatactttacagaagctgcaaatcccgacaattatatttatcaataagattgaccgagccggtgtgaatttggagcgtttgtatctggatataaaagcaaatctgtctcaagatgtcctgtttatgcaaaatgttgtcgatggatcggtttatccggtttgctcccaaacatatataaaggaagaatacaaagaatttgtatgcaaccatgacgacaatatattagaacgatatttggcggatagcgaaatttcaccggctgattattggaatacgataatcgctcttgtggcaaaagccaaagtctatccggtgctacatggatcagcaatgttcaatatcggtatcaatgagttgttggacgccatcacttcttttatacttcctccggcatcggtctcaaacagactttcatcttatctttataagatagagcatgaccccaaaggacataaaagaagttttctaaaaataattgacggaagtctgagacttcgagacgttgtaagaatcaacgattcggaaaaattcatcaagattaaaaatctaaaaactatcaatcagggcagagagataaatgttgatgaagtgggcgccaatgatatcgcgattgtagaggatatggatgattttcgaatcggaaattatttaggtgctgaaccttgtttgattcaaggattatcgcatcagcatcccgctctcaaatcctccgtccggccagacaggcccgaagagagaagcaaggtgatatccgctctgaatacattgtggattgaagacccgtctttgtccttttccataaactcatatagtgatgaattggaaatctcgttatatggtttaacccaaaaggaaatcatacagacattgctggaagaacgattttccgtaaaggtccattttgatgagatcaagactatatacaaagaacgacctgtaaaaaaggtcaataagattattcagatcgaagtgccgcccaacccttattgggccacaatagggctgactcttgaacccttaccgttagggacagggttgcaaatcgaaagtgacatctcctatggttatctgaaccattcttttcaaaatgccgtttttgaagggattcgtatgtcttgccaatccgggttacatggatgggaagtgactgatctgaaagtaacttttactcaagccgagtattatagcccggtaagtacacctgctgatttcagacagctgaccccttatgtcttcaggctggccttgcaacagtcaggtgtggacattctcgaaccgatgctctattttgagttgcagataccccaagcggcaagttccaaagctattacagatttgcaaaaaatgatgtctgagattgaagacatcagttgcaataatgagtggtgtcatattaaagggaaagttccattaaatacaagtaaagactatgcatcagaagtaagttcatacactaagggcttaggcatttttatggttaagccatgcgggtatcaaataacaaaaggcggttattctgataatatccgcatgaacgaaaaagataaacttttattcatgttccaaaaatcaatgtcatcaaaataatggtataacctctccttaatttattgcatctcttttcgaatatttatgttttttgagaaaagaacgtactcatggttcatcccgatatgcgtatcggtctgtatatcagcaactttctatgtgtttcaactacaatagtcatctattctcatctttctgagtccaccccctgcaaagcccctctttacgacataaaaattcggtcggaaaaggtatgcaaaagatgtttctctctttaagagaaactcttcgggatgcaaaaatatgaaaataactccaattcaccaaattatatagcgacttttttacaaaatgctaaaatttgttgatttccgtcaagcaattgttgagcaaaaatgtcttttacgataaaatgatacctcaatatcaactgtttagcaaaacgatatttctcttaaagagagaaacacctttttgttcaccaatccccgacttttaatcccgcggccatgattgaaaaaggaagagtatgagtattcaacatttccgtgtcgcccttattcccttttttgcggcattttgccttcctgtttttgctcacccagaaacgctggtgaaagtaaaagatgctgaagatcagttgggtgcacgagtgggttacatcgaactggatctcaacagcggtaagatccttgagagttttcgccccgaagaacgttttccaatgatgagcacttttaaagttctgctatgtggcgcggtattatcccgtattgacgccgggcaagagcaactcggtcgccgcatacactattctcagaatgacttggttgagtactcaccagtcacagaaaagcatcttacggatggcatgacagtaagagaattatgcagtgctgccataaccatgagtgataacactgcggccaacttacttctgacaacgatcggaggaccgaaggagctaaccgcttttttgcacaacatgggggatcatgtaactcgccttgatcgttgggaaccggagctgaatgaagccataccaaacgacgagcgtgacaccacgatgcctgtagcaatggcaacaacgttgcgcaaactattaactggcgaactacttactctagcttcccggcaacaattaatagactggatggaggcggataaagttgcaggaccacttctgcgctcggcccttccggctggctggtttattgctgataaatctggagccggtgagcgtgggtcacgcggtatcattgcagcactggggccagatggtaagccctcccgtatcgtagttatctacacgacggggagtcaggcaactatggatgaacgaaatagacagatcgctgagataggtgcctcactgattaagcattggtaactgtcagaccaagtttactcataacgcgtcaattcgagggggatcaattccgtgataggtgggctgcccttcctggttggcttggtttcatcagccatccgcttgccctcatctgttacgccggcggtagccggccagcctcgcagagcaggattcccgttgagcaccgccaggtgcgaataagggacagtgaagaaggaacacccgctcgcgggtgggcctacttcacctatcctgcccggctgacgccgttggatacaccaaggaaagtctacacgaaccctttggcaaaatcctgtatatcgtgcgaaaaaggatggatataccgaaaaaatcgctataatgaccccgaagcagggttatgcagcggaaaacggaattgatccggccacgatgcgtccggcgtagaggatctgaagatcagcagttcaacctgttgatagtacgtactaagctctcatgtttcacgtactaagctctcatgtttaacgtactaagctctcatgtttaacgaactaaaccctcatggctaacgtactaagctctcatggctaacgtactaagctctcatgtttcacgtactaagctctcatgtttgaacaataaaattaatataaatcagcaacttaaatagcctctaaggttttaagttttataagaaaaaaaagaatatataaggcttttaaagcttttaaggtttaacggttgtggacaacaagccagg

>pBH306

gatgtaacgcactgagaagcccttagagcctctcaaagcaattttgagtgacacaggaacacttaacggctgacatgggaattcccctccaccgcggtgtattaatgcggctgcCCCTcggttcctggccttttgctggccttttgctcacatgttctttcctgcgttatcccctgattctgtggataaccgtattaccgcctttgagtgagctgatacAACGcattggctgcaacaatcagccttgatctggaagaagcaatgaaagctgctgttaagtctccgaatcaggtattgttcctgacaggtgtattcccatccggtaaacgcggatactttgcagttgatctgactcaggaataaattataaattaaggtaagaagattgtaggataagctaatgaaatagaaaaaggatgccgtcacacaacttgtcggcattcttttttgttttattagttgaaaatatagtgaaaaagttgcctaaatatgtatgttaacaaattatttgtcgtaactttgcactccaaatctgtttttaacatatggcactagtgAAACCAGTAACGTTATACGATGTCGCAGAGTATGCCGGTGTCTCTACCGCGACCGTTTCCAACGTGGTGAACCAGGCCAGCCACGTTTCTGCGAAAACGCGGGAAAAAGTGGAAGCGGCGATGGCGGAGCTCAATTACATTCCCAACCGCGTGGCACAACAACTGGCGGGCAAAGCGTCGCATACCATTGGCATGTTGATCACTGCCAGTACCAATCCTTTCTATTCAGAACTGGTGCGTGGCGTTGAACGCAGCTGCTTCGAACGCGGTTATAGTCTCGTCCTTTGCAATACCGAAGGCGATGAACAGCGGATGAATCGCAATCTGGAAACGCTGATGCAAAAACGCGTTGATGGCTTGCTGTTACTGTGCACCGAAACGCATCAACCTTCGCGTGAAATCATGCAACGTTATCCGACAGTGCCTACTGTGATGATGGACTGGGCTCCGTTCGATGGCGACAGCGATCTTATTCAGGATAACTCGTTGCTGGGCGGAGACTTAGCAACGCAATATCTGATCGATAAAGGTCATACCCGTATCGCCTGTATTACCGGCCCGCTGGATAAAACTCCGGCGCGCCTGCGGTTGGAAGGTTATCGGGCGGCGATGAAACGTGCGGGTCTCAACATTCCTGATGGCTATGAAGTCACTGGTGATTTTGAATTTAACGGCGGGTTTGACGCTATGCGCCAACTGCTATCACATCCGCTGCGTCCTCAGGCCGTCTTTACCGGAAATGACGCTATGGCTGTTGGCGTTTACCAGGCGTTATATCAGGCAGAGTTACAGGTTCCGCAGGATATCGCGGTGATTGGCTATGACGATATCGAACTGGCAAGCTTTATGACGCCACCATTAACCACTATCCACCAACCGAAAGATGAACTGGGGGAGCTGGCGATTGATGTACTCATCCATCGGATAACCCAGCCGACCCTTCAGCAACAACGATTACAACTTACTCCGATTCTGATGGAACGCGGTTCGGCTTAGCTGGTGAAAAGAAAAACCACCCTGGCGCCCAATACGCAAACCGCCTCTCCCCGCGCGTTGGCCGATTCATTAATGCAGCTGGCACGACAGGTTTCCCGACTGGAAAGCGGGCAGTGAgctttcctcggtaccaaattccagaaaagaggcctcccgaaaggggggccttttttcgttttggtcctacttgtgcctgttctatttccTATGcggttcctggccttttgctggccttttgctcacatgttctttcctgcgttatcccctgattctgtggataaccgtattaccgcctttgagtgagctgatacATCCcaaatgctgttccatcactggaagataggcaattagtagaagcaatttgattagtaatctaaaagcactccgataacgattagagtgcttttagattgtttatcattaattatcaaagcaagtgcagttcatcaCTGCCCGCTTTCCAGTCGGGAAACCTGTCGTGCCAGCTGCATTAATGAATCGGCCAACGCGCGGGGAGAGGCGGTTTGCGTATTGGGCGCCAGGGTGGTTTTTCTTTTCACCAGTGAGACGGGCAACAGCTGATTGCCCTTCACCGCCTGGCCCTGAGAGAGTTGCAGCAAGCGGTCCACGCTGGTTTGCCCCAGCAGGCGAAAATCCTGTTTGATGGTGGTTAACGGCGGGATATAACATGAGCTGTCTTCGGTATCGTCGTATCCCACTACCGAGATATCCGCACCAACGCGCAGCCCGGTCTCGGTAATGGCGCGCATTGCGCCCAGCGCCATCTGATCGTTGGCAACCAGCATCGCAGTGGGAACGATGCCCTCATTCAGCATTTGCATGGTTTGTTGAAAACCGGACATGGCACTCCAGTCGCCTTCCCGTTCCGCTATCGGCTGAATTTGATTGCGAGTGAGATATTTATGCCAGCCAGCCAGACGCAGACGCGCCGAGACAGAACTTAATGGGCCCGCTAACAGCGCGATTTGCTGGTGACCCAATGCGACCAGATGCTCCACGCCCAGTCGCGTACCGTCTTCATGGGAGAAAATAATACTGTTGATGGGTGTCTGGTCAGAGACATCAAGAAATAACGCCGGAACATTAGTGCAGGCAGCTTCCACAGCAATGGCATCCTGGTTATCCAGCGGATAGTTAATGATCAGCCCACTGACGCGTTGCGCGAGAAGATTGTGCACCGCCGTTTTACAGGCTTCGATGCCGCTTCGTTCTACCATCGACACGAACACGCTGGCACCCAGTTGATAGGCGCGAGATTTAATCGCCGCGACAATTTGCGACAGCGCGTGCAGGGCCAGACTGGAGGTGGCAACGCCAATCAGCAACGACTGTTTGCCCGCCAGTTGTTGTGCCACGCGGTTGGGAATGTAATTCAGCTCCGCCATCGCCGCTTCCACTTTTTCCCGCGTTTTCGCAGAAACGTGGCTGGCCTGGTTCACCACattGGAAACGGTctgatgAGAGACACCGGCATACTCTGCGACATCGTATAACGTTACTGGTTTcacgtttatattatttatatttgtttgacgagaatatctttatttAATTttaAGCGCTtaaAATTgccgacaaaggtaAATTttaAGCGCTtaaAATTcaaataaatagatagaaaaataacagtttgtcgaattttctttgtacCCGAtaattgcctatcttccagtgatggaacagcatttgtgcattggctgcaacaatcagccttacttgtgcctgttctatttccgaaccgaccgcttgtatgaatGcatcaaaattcgttttctctaCgttggattccttgttgctcatattgtgatgataatttctacaaatatagtcattggtaactatctatgaaactgtttgatacttttatcagtctaatagttttacaaggtctttcttcatttcttcgtcaatatccctgtatcgtctgaaagctttgcttccctccttgtgtcccgacagtgcggaaacaaggttcgggtctttcacttttttatagatattgccgataaacgtacgtcttgccagatggctgcttgccacttcataaataggtcgtttgatttcgttgtgcgtcaacgggtctaagattgttacgatgcggtcaactccagctaatttgaatatcttttttatggcatcattgtacttttgctcggatatgaacggcaacagttttccctcatattctttgtagcgttcaaggatttctttcgctttgtcgttaagtggaacacgtaccgtaaccggattcccctctttggttttcttgggaatatattctatggcttcattgaccacatttagtttggtcattcggtacaggtcgctcaccctgcatcctatcagtgtctgaaatatgaatatatccctctgtattgccagttgtggggtggcagaaaggtctgcattaaaaatcctgtccctttcttcgagtgttatataataaggtgtaccatatgtacactcctctatcggaaacttgtcgaaaggtctgtttgtggtgcgtttgttatcgaagcaccacaggaagaatgtgcgtattcttgaaaaacagtctatcagcgtgtttttgcttctgggctgtggtgtcctcttttcgggaatggcttcataaatgctcgggtaaagttcataatactggtattcgttctgaaagaaatcccacatatcccgaagcgtgtcaggtgttaccaaatccacatcaaggataaagcccttttgtcctctctttgtagcccttacatatagttcataacgcagtaaggctcttttgacaacccggaaatttttctttcgtacttccgacaaagggtgcttgtttagaaattcatcgaatagttctccaatggtaggcttgataaccacttcctccggcaaaaaatatttttcaggatggtaaaatttatcaagtgttgtttttagccattctttgtctattgcttccttttcttgttgatataccttctcgatataggttttcaactggcgtatctcttcatttatatgggtacgcatttcttcattgcagacagctttcgtttttacacattcgtctttatcatcccatagattgggattgatggctaactgagtaggagcaacggagtctaactgccttccgtttctaaaacggacatagattgtagccatggattctgtatcatatcgtttggctgcttttttaatgataaaggttactttcatagactttcaggttgaattttactctgctgcaaatataaatattttccccagcattttccccacatctgctaaatattttgcaattcgattaaacttggattaaaatttaataggattataacatattgaaatacagtgtaattgtggcgtttttccgcatttttctttttacccacattttccccactttaattatttcaaatgcggggtctgggtacaagaaagaaagctaagtatttgatagttcaatacttagctttttcttttgcttgaattttccccacattttccccacacgtgcaaaaaatatagcagtaagtcattatttcttttggttgaacgtagagagtagcgatattaaaagaatccgatgagaaaagactaatatttatctatccattcagtttgatttttcaggactttacatcgtcctgaaagtatttgttggtaccggtaccgaggacgcgtaaacatttacaGTTGCATGTGGCCTATTGTTTaggacgcgttatctccttaacgtacgttttcgttccattggccctcaaaccccgttatatacattcatgtccatttatgtaaaaaatcctgctgaccttgtttatgtcttgtcagtcaccatttgcaaaaccatatttgaccctcaaagaggctgaatttgataagcaacttgctacatactcataataaggagctaaatagaacacgaatgggaaatactcaaatgccaaactaaagaagatattggccaaaataaacgctataccgagagagaaacttgatttttcaacttcctaaccaacagtgttgttcaaacatttctacttatttgtacttaccagttgaacctacgtttccctaataaaatgtctatggtaaaaagttaaaaaatcctcctacttttgttagatatatttttttgtgtaattttgtaatcgttatgcggcagtaataatatacatattaatacgagttaggaatcctgtagttctcatatgctacgaggaggtattaaaaggtgcgtttcgacaatgcatctattgtagtatattattgcttaatccaaatgaatattataaatttaggaattcttgctcacattgatgcaggaaaaacttccgtaaccgagaatctgctgtttgccagtggagcaacggaaaagtgcggctgtgtggataatggtgacaccataacggactctatggatatagagaaacgtagaggaattactgttcgggcttctacgacatctattatctggaatggtgtgaaatgcaatatcattgacactccgggacacatggattttattgcggaagtggagcggacattcaaaatgcttgatggagcagtcctcatcttatccgcaaaggaaggcatacaagcgcagacaaagttgctgttcaatactttacagaagctgcaaatcccgacaattatatttatcaataagattgaccgagccggtgtgaatttggagcgtttgtatctggatataaaagcaaatctgtctcaagatgtcctgtttatgcaaaatgttgtcgatggatcggtttatccggtttgctcccaaacatatataaaggaagaatacaaagaatttgtatgcaaccatgacgacaatatattagaacgatatttggcggatagcgaaatttcaccggctgattattggaatacgataatcgctcttgtggcaaaagccaaagtctatccggtgctacatggatcagcaatgttcaatatcggtatcaatgagttgttggacgccatcacttcttttatacttcctccggcatcggtctcaaacagactttcatcttatctttataagatagagcatgaccccaaaggacataaaagaagttttctaaaaataattgacggaagtctgagacttcgagacgttgtaagaatcaacgattcggaaaaattcatcaagattaaaaatctaaaaactatcaatcagggcagagagataaatgttgatgaagtgggcgccaatgatatcgcgattgtagaggatatggatgattttcgaatcggaaattatttaggtgctgaaccttgtttgattcaaggattatcgcatcagcatcccgctctcaaatcctccgtccggccagacaggcccgaagagagaagcaaggtgatatccgctctgaatacattgtggattgaagacccgtctttgtccttttccataaactcatatagtgatgaattggaaatctcgttatatggtttaacccaaaaggaaatcatacagacattgctggaagaacgattttccgtaaaggtccattttgatgagatcaagactatatacaaagaacgacctgtaaaaaaggtcaataagattattcagatcgaagtgccgcccaacccttattgggccacaatagggctgactcttgaacccttaccgttagggacagggttgcaaatcgaaagtgacatctcctatggttatctgaaccattcttttcaaaatgccgtttttgaagggattcgtatgtcttgccaatccgggttacatggatgggaagtgactgatctgaaagtaacttttactcaagccgagtattatagcccggtaagtacacctgctgatttcagacagctgaccccttatgtcttcaggctggccttgcaacagtcaggtgtggacattctcgaaccgatgctctattttgagttgcagataccccaagcggcaagttccaaagctattacagatttgcaaaaaatgatgtctgagattgaagacatcagttgcaataatgagtggtgtcatattaaagggaaagttccattaaatacaagtaaagactatgcatcagaagtaagttcatacactaagggcttaggcatttttatggttaagccatgcgggtatcaaataacaaaaggcggttattctgataatatccgcatgaacgaaaaagataaacttttattcatgttccaaaaatcaatgtcatcaaaataatggtataacctctccttaatttattgcatctcttttcgaatatttatgttttttgagaaaagaacgtactcatggttcatcccgatatgcgtatcggtctgtatatcagcaactttctatgtgtttcaactacaatagtcatctattctcatctttctgagtccaccccctgcaaagcccctctttacgacataaaaattcggtcggaaaaggtatgcaaaagatgtttctctctttaagagaaactcttcgggatgcaaaaatatgaaaataactccaattcaccaaattatatagcgacttttttacaaaatgctaaaatttgttgatttccgtcaagcaattgttgagcaaaaatgtcttttacgataaaatgatacctcaatatcaactgtttagcaaaacgatatttctcttaaagagagaaacacctttttgttcaccaatccccgacttttaatcccgcggccatgattgaaaaaggaagagtatgagtattcaacatttccgtgtcgcccttattcccttttttgcggcattttgccttcctgtttttgctcacccagaaacgctggtgaaagtaaaagatgctgaagatcagttgggtgcacgagtgggttacatcgaactggatctcaacagcggtaagatccttgagagttttcgccccgaagaacgttttccaatgatgagcacttttaaagttctgctatgtggcgcggtattatcccgtattgacgccgggcaagagcaactcggtcgccgcatacactattctcagaatgacttggttgagtactcaccagtcacagaaaagcatcttacggatggcatgacagtaagagaattatgcagtgctgccataaccatgagtgataacactgcggccaacttacttctgacaacgatcggaggaccgaaggagctaaccgcttttttgcacaacatgggggatcatgtaactcgccttgatcgttgggaaccggagctgaatgaagccataccaaacgacgagcgtgacaccacgatgcctgtagcaatggcaacaacgttgcgcaaactattaactggcgaactacttactctagcttcccggcaacaattaatagactggatggaggcggataaagttgcaggaccacttctgcgctcggcccttccggctggctggtttattgctgataaatctggagccggtgagcgtgggtcacgcggtatcattgcagcactggggccagatggtaagccctcccgtatcgtagttatctacacgacggggagtcaggcaactatggatgaacgaaatagacagatcgctgagataggtgcctcactgattaagcattggtaactgtcagaccaagtttactcataacgcgtcaattcgagggggatcaattccgtgataggtgggctgcccttcctggttggcttggtttcatcagccatccgcttgccctcatctgttacgccggcggtagccggccagcctcgcagagcaggattcccgttgagcaccgccaggtgcgaataagggacagtgaagaaggaacacccgctcgcgggtgggcctacttcacctatcctgcccggctgacgccgttggatacaccaaggaaagtctacacgaaccctttggcaaaatcctgtatatcgtgcgaaaaaggatggatataccgaaaaaatcgctataatgaccccgaagcagggttatgcagcggaaaacggaattgatccggccacgatgcgtccggcgtagaggatctgaagatcagcagttcaacctgttgatagtacgtactaagctctcatgtttcacgtactaagctctcatgtttaacgtactaagctctcatgtttaacgaactaaaccctcatggctaacgtactaagctctcatggctaacgtactaagctctcatgtttcacgtactaagctctcatgtttgaacaataaaattaatataaatcagcaacttaaatagcctctaaggttttaagttttataagaaaaaaaagaatatataaggcttttaaagcttttaaggtttaacggttgtggacaacaagccagg

>pBH501

gatgtaacgcactgagaagcccttagagcctctcaaagcaattttgagtgacacaggaacacttaacggctgacatgggaattcccctccaccgcggtgtattaatgcggctgcCCCTgcacctgggtagctcaatgcgcgcaataaaaaagcccccggaaggtgatcttccgggggctttctcatgcgttggtgttcactcccattcTATGcggttcctggccttttgctggccttttgctcacatgttctttcctgcgttatcccctgattctgtggataaccgtattaccgcctttgagtgagctgatacatcccaaatgctgctcggtaccaaagacgaaCaataagacgctgaaaagcgtcttttttcgttttggtccgataaagtttggaagataaagctaaaagttcttatctttgcagtccgaaataaagacatataaaagaaaagacaccatggataagaaatactcaataggcttagctatcggcacaaatagcgtcggatgggcggtgatcactgatgaatataaggttccgtctaaaaagttcaaggttctgggaaatacagaccgccacagtatcaaaaaaaatcttataggggctcttttatttgacagtggagagacagcggaagcgactcgtctcaaacggacagctcgtagaaggtatacacgtcggaagaatcgtatttgttatctacaggagattttttcaaatgagatggcgaaagtagatgatagtttctttcatcgacttgaagagtcttttttggtggaagaagacaagaagcatgaacgtcatcctatttttggaaatatagtagatgaagttgcttatcatgagaaatatccaactatctatcatctgcgaaaaaaattggtagattctactgataaagcggatttgcgcttaatctatttggccttagcgcatatgattaagtttcgtggtcattttttgattgagggagatttaaatcctgataatagtgatgtggacaaactatttatccagttggtacaaacctacaatcaattatttgaagaaaaccctattaacgcaagtggagtagatgctaaagcgattctttctgcacgattgagtaaatcaagacgattagaaaatctcattgctcagctccccggtgagaagaaaaatggcttatttgggaatctcattgctttgtcattgggtttgacccctaattttaaatcaaattttgatttggcagaagatgctaaattacagctttcaaaagatacttacgatgatgatttagataatttattggcgcaaattggagatcaatatgctgatttgtttttggcagctaagaatttatcagatgctattttactttcagatatcctaagagtaaatactgaaataactaaggctcccctatcagcttcaatgattaaacgctacgatgaacatcatcaagacttgactcttttaaaagctttagttcgacaacaacttccagaaaagtataaagaaatcttttttgatcaatcaaaaaacggatatgcaggttatattgatgggggagctagccaagaagaattttataaatttatcaaaccaattttagaaaaaatggatggtactgaggaattattggtgaaactaaatcgtgaagatttgctgcgcaagcaacggacctttgacaacggctctattccccatcaaattcacttgggtgagctgcatgctattttgagaagacaagaagacttttatccatttttaaaagacaatcgtgagaagattgaaaaaatcttgacttttcgaattccttattatgttggtccattggcgcgtggcaatagtcgttttgcatggatgactcggaagtctgaagaaacaattaccccatggaattttgaagaagttgtcgataaaggtgcttcagctcaatcatttattgaacgcatgacaaactttgataaaaatcttccaaatgaaaaagtactaccaaaacatagtttgctttatgagtattttacggtttataacgaattgacaaaggtcaaatatgttactgaaggaatgcgaaaaccagcatttctttcaggtgaacagaagaaagccattgttgatttactcttcaaaacaaatcgaaaagtaaccgttaagcaattaaaagaagattatttcaaaaaaatagaatgttttgatagtgttgaaatttcaggagttgaagatagatttaatgcttcattaggtacctaccatgatttgctaaaaattattaaagataaagattttttggataatgaagaaaatgaagatatcttagaggatattgttttaacattgaccttatttgaagatagggagatgattgaggaaagacttaaaacatatgctcacctctttgatgataaggtgatgaaacagcttaaacgtcgccgttatactggttggggacgtttgtctcgaaaattgattaatggtattagggataagcaatctggcaaaacaatattagattttttgaaatcagatggttttgccaatcgcaattttatgcagctgatccatgatgatagtttgacatttaaagaagacattcaaaaagcacaagtgtctggacaaggcgatagtttacatgaacatattgcaaatttagctggtagccctgctattaaaaaaggtattttacagactgtaaaagttgttgatgaattggtcaaagtaatggggcggcataagccagaaaatatcgttattgaaatggcacgtgaaaatcagacaactcaaaagggccagaaaaattcgcgagagcgtatgaaacgaatcgaagaaggtatcaaagaattaggaagtcagattcttaaagagcatcctgttgaaaatactcaattgcaaaatgaaaagctctatctctattatctccaaaatggaagagacatgtatgtggaccaagaattagatattaatcgtttaagtgattatgatgtcgatgccattgttccacaaagtttccttaaagacgattcaatagacaataaggtcttaacgcgttctgataaaaatcgtggtaaatcggataacgttccaagtgaagaagtagtcaaaaagatgaaaaactattggagacaacttctaaacgccaagttaatcactcaacgtaagtttgataatttaacgaaagctgaacgtggtggtttgagtgaacttgataaagctggttttatcaaacgccaattggttgaaactcgccaaatcactaagcatgtggcacaaattttggatagtcgcatgaatactaaatacgatgaaaatgataaacttattcgagaggttaaagtgattaccttaaaatctaaattagtttctgacttccgaaaagatttccaattctataaagtacgtgagattaacaattaccatcatgcccatgatgcgtatctaaatgccgtcgttggaactgctttgattaagaaatatccaaaacttgaatcggagtttgtctatggtgattataaagtttatgatgttcgtaaaatgattgctaagtctgagcaagaaataggcaaagcaaccgcaaaatatttcttttactctaatatcatgaacttcttcaaaacagaaattacacttgcaaatggagagattcgcaaacgccctctaatcgaaactaatggggaaactggagaaattgtctgggataaagggcgagattttgccacagtgcgcaaagtattgtccatgccccaagtcaatattgtcaagaaaacagaagtacagacaggcgaattctccaaggagtcaattttaccaaaaagaaattcggacaagcttattgctcgtaaaaaagactgggatccaaaaaaatatggtggttttgatagtccaacggtagcttattcagtcctagtggttgctaaggtggaaaaagggaaatcgaagaagttaaaatccgttaaagagttactagggatcacaattatggaaagaagttcctttgaaaaaaatccgattgactttttagaagctaaaggatataaggaagttaaaaaagacttaatcattaaactacctaaatatagtctttttgagttagaaaacggtcgtaaacggatgctggctagtgccggagaattacaaaaaggaaatgagctggctctgccaagcaaatatgtgaattttttatatttagctagtcattatgaaaagttgaagggtagtccagaagataacgaacaaaaacaattgtttgtggagcagcataagcattatttagatgagattattgagcaaatcagtgaattttctaagcgtgttattttagcagatgccaatttagataaagttcttagtgcatataacaaacatagagacaaaccaatacgtgaacaagcagaaaatattattcatttatttacgttgacgaatcttggagctcccgctgcttttaaatattttgatacaacaattgatcgtaaacgatatacgtctacaaaagaagttttagatgccactcttatccatcaatccatcactggtctttatgaaacacgcattgatttgagtcagctaggaggtgactgattctttgtacCCGAtaattgcctatcttccagtgatggaacagcatttgtgcattggctgcaacaatcagccttacttgtgcctgttctatttccgaaccgaccgcttgtatgaatGcatcaaaattcgttttctctaCgttggattccttgttgctcatattgtgatgataatttctacaaatatagtcattggtaactatctatgaaactgtttgatacttttatcagtctaatagttttacaaggtctttcttcatttcttcgtcaatatccctgtatcgtctgaaagctttgcttccctccttgtgtcccgacagtgcggaaacaaggttcgggtctttcacttttttatagatattgccgataaacgtacgtcttgccagatggctgcttgccacttcataaataggtcgtttgatttcgttgtgcgtcaacgggtctaagattgttacgatgcggtcaactccagctaatttgaatatcttttttatggcatcattgtacttttgctcggatatgaacggcaacagttttccctcatattctttgtagcgttcaaggatttctttcgctttgtcgttaagtggaacacgtaccgtaaccggattcccctctttggttttcttgggaatatattctatggcttcattgaccacatttagtttggtcattcggtacaggtcgctcaccctgcatcctatcagtgtctgaaatatgaatatatccctctgtattgccagttgtggggtggcagaaaggtctgcattaaaaatcctgtccctttcttcgagtgttatataataaggtgtaccatatgtacactcctctatcggaaacttgtcgaaaggtctgtttgtggtgcgtttgttatcgaagcaccacaggaagaatgtgcgtattcttgaaaaacagtctatcagcgtgtttttgcttctgggctgtggtgtcctcttttcgggaatggcttcataaatgctcgggtaaagttcataatactggtattcgttctgaaagaaatcccacatatcccgaagcgtgtcaggtgttaccaaatccacatcaaggataaagcccttttgtcctctctttgtagcccttacatatagttcataacgcagtaaggctcttttgacaacccggaaatttttctttcgtacttccgacaaagggtgcttgtttagaaattcatcgaatagttctccaatggtaggcttgataaccacttcctccggcaaaaaatatttttcaggatggtaaaatttatcaagtgttgtttttagccattctttgtctattgcttccttttcttgttgatataccttctcgatataggttttcaactggcgtatctcttcatttatatgggtacgcatttcttcattgcagacagctttcgtttttacacattcgtctttatcatcccatagattgggattgatggctaactgagtaggagcaacggagtctaactgccttccgtttctaaaacggacatagattgtagccatggattctgtatcatatcgtttggctgcttttttaatgataaaggttactttcatagactttcaggttgaattttactctgctgcaaatataaatattttccccagcattttccccacatctgctaaatattttgcaattcgattaaacttggattaaaatttaataggattataacatattgaaatacagtgtaattgtggcgtttttccgcatttttctttttacccacattttccccactttaattatttcaaatgcggggtctgggtacaagaaagaaagctaagtatttgatagttcaatacttagctttttcttttgcttgaattttccccacattttccccacacgtgcaaaaaatatagcagtaagtcattatttcttttggttgaacgtagagagtagcgatattaaaagaatccgatgagaaaagactaatatttatctatccattcagtttgatttttcaggactttacatcgtcctgaaagtatttgttggtaccggtaccgaggacgcgtaaacatttacaGTTGCATGTGGCCTATTGTTTaggacgcgttatctccttaacgtacgttttcgttccattggccctcaaaccccgttatatacattcatgtccatttatgtaaaaaatcctgctgaccttgtttatgtcttgtcagtcaccatttgcaaaaccatatttgaccctcaaagaggctgaatttgataagcaacttgctacatactcataataaggagctaaatagaacacgaatgggaaatactcaaatgccaaactaaagaagatattggccaaaataaacgctataccgagagagaaacttgatttttcaacttcctaaccaacagtgttgttcaaacatttctacttatttgtacttaccagttgaacctacgtttccctaataaaatgtctatggtaaaaagttaaaaaatcctcctacttttgttagatatatttttttgtgtaattttgtaatcgttatgcggcagtaataatatacatattaatacgagttaggaatcctgtagttctcatatgctacgaggaggtattaaaaggtgcgtttcgacaatgcatctattgtagtatattattgcttaatccaaatgaatattataaatttaggaattcttgctcacattgatgcaggaaaaacttccgtaaccgagaatctgctgtttgccagtggagcaacggaaaagtgcggctgtgtggataatggtgacaccataacggactctatggatatagagaaacgtagaggaattactgttcgggcttctacgacatctattatctggaatggtgtgaaatgcaatatcattgacactccgggacacatggattttattgcggaagtggagcggacattcaaaatgcttgatggagcagtcctcatcttatccgcaaaggaaggcatacaagcgcagacaaagttgctgttcaatactttacagaagctgcaaatcccgacaattatatttatcaataagattgaccgagccggtgtgaatttggagcgtttgtatctggatataaaagcaaatctgtctcaagatgtcctgtttatgcaaaatgttgtcgatggatcggtttatccggtttgctcccaaacatatataaaggaagaatacaaagaatttgtatgcaaccatgacgacaatatattagaacgatatttggcggatagcgaaatttcaccggctgattattggaatacgataatcgctcttgtggcaaaagccaaagtctatccggtgctacatggatcagcaatgttcaatatcggtatcaatgagttgttggacgccatcacttcttttatacttcctccggcatcggtctcaaacagactttcatcttatctttataagatagagcatgaccccaaaggacataaaagaagttttctaaaaataattgacggaagtctgagacttcgagacgttgtaagaatcaacgattcggaaaaattcatcaagattaaaaatctaaaaactatcaatcagggcagagagataaatgttgatgaagtgggcgccaatgatatcgcgattgtagaggatatggatgattttcgaatcggaaattatttaggtgctgaaccttgtttgattcaaggattatcgcatcagcatcccgctctcaaatcctccgtccggccagacaggcccgaagagagaagcaaggtgatatccgctctgaatacattgtggattgaagacccgtctttgtccttttccataaactcatatagtgatgaattggaaatctcgttatatggtttaacccaaaaggaaatcatacagacattgctggaagaacgattttccgtaaaggtccattttgatgagatcaagactatatacaaagaacgacctgtaaaaaaggtcaataagattattcagatcgaagtgccgcccaacccttattgggccacaatagggctgactcttgaacccttaccgttagggacagggttgcaaatcgaaagtgacatctcctatggttatctgaaccattcttttcaaaatgccgtttttgaagggattcgtatgtcttgccaatccgggttacatggatgggaagtgactgatctgaaagtaacttttactcaagccgagtattatagcccggtaagtacacctgctgatttcagacagctgaccccttatgtcttcaggctggccttgcaacagtcaggtgtggacattctcgaaccgatgctctattttgagttgcagataccccaagcggcaagttccaaagctattacagatttgcaaaaaatgatgtctgagattgaagacatcagttgcaataatgagtggtgtcatattaaagggaaagttccattaaatacaagtaaagactatgcatcagaagtaagttcatacactaagggcttaggcatttttatggttaagccatgcgggtatcaaataacaaaaggcggttattctgataatatccgcatgaacgaaaaagataaacttttattcatgttccaaaaatcaatgtcatcaaaataatggtataacctctccttaatttattgcatctcttttcgaatatttatgttttttgagaaaagaacgtactcatggttcatcccgatatgcgtatcggtctgtatatcagcaactttctatgtgtttcaactacaatagtcatctattctcatctttctgagtccaccccctgcaaagcccctctttacgacataaaaattcggtcggaaaaggtatgcaaaagatgtttctctctttaagagaaactcttcgggatgcaaaaatatgaaaataactccaattcaccaaattatatagcgacttttttacaaaatgctaaaatttgttgatttccgtcaagcaattgttgagcaaaaatgtcttttacgataaaatgatacctcaatatcaactgtttagcaaaacgatatttctcttaaagagagaaacacctttttgttcaccaatccccgacttttaatcccgcggccatgattgaaaaaggaagagtatgagtattcaacatttccgtgtcgcccttattcccttttttgcggcattttgccttcctgtttttgctcacccagaaacgctggtgaaagtaaaagatgctgaagatcagttgggtgcacgagtgggttacatcgaactggatctcaacagcggtaagatccttgagagttttcgccccgaagaacgttttccaatgatgagcacttttaaagttctgctatgtggcgcggtattatcccgtattgacgccgggcaagagcaactcggtcgccgcatacactattctcagaatgacttggttgagtactcaccagtcacagaaaagcatcttacggatggcatgacagtaagagaattatgcagtgctgccataaccatgagtgataacactgcggccaacttacttctgacaacgatcggaggaccgaaggagctaaccgcttttttgcacaacatgggggatcatgtaactcgccttgatcgttgggaaccggagctgaatgaagccataccaaacgacgagcgtgacaccacgatgcctgtagcaatggcaacaacgttgcgcaaactattaactggcgaactacttactctagcttcccggcaacaattaatagactggatggaggcggataaagttgcaggaccacttctgcgctcggcccttccggctggctggtttattgctgataaatctggagccggtgagcgtgggtcacgcggtatcattgcagcactggggccagatggtaagccctcccgtatcgtagttatctacacgacggggagtcaggcaactatggatgaacgaaatagacagatcgctgagataggtgcctcactgattaagcattggtaactgtcagaccaagtttactcataacgcgtcaattcgagggggatcaattccgtgataggtgggctgcccttcctggttggcttggtttcatcagccatccgcttgccctcatctgttacgccggcggtagccggccagcctcgcagagcaggattcccgttgagcaccgccaggtgcgaataagggacagtgaagaaggaacacccgctcgcgggtgggcctacttcacctatcctgcccggctgacgccgttggatacaccaaggaaagtctacacgaaccctttggcaaaatcctgtatatcgtgcgaaaaaggatggatataccgaaaaaatcgctataatgaccccgaagcagggttatgcagcggaaaacggaattgatccggccacgatgcgtccggcgtagaggatctgaagatcagcagttcaacctgttgatagtacgtactaagctctcatgtttcacgtactaagctctcatgtttaacgtactaagctctcatgtttaacgaactaaaccctcatggctaacgtactaagctctcatggctaacgtactaagctctcatgtttcacgtactaagctctcatgtttgaacaataaaattaatataaatcagcaacttaaatagcctctaaggttttaagttttataagaaaaaaaagaatatataaggcttttaaagcttttaaggtttaacggttgtggacaacaagccagg

>pBH502

gtttaacggttgtggacaacaagccagggatgtaacgcactgagaagcccttagagcctctcaaagcaattttgagtgacacaggaacacttaacggctgacatgggaattcccctccaccgcggtggCCCTgtacaaagaaaattcgacaaactgttatttttctatctatttatttgaattgtgagcggataacaattacctttgtcggcaattgtgagcggataacaattATGATCTGTCCTGATGAGTCCGTGAGGACGAAACGAGTAAGCTCGTCGACAGAACGATGCGCTGAATGTTTTAGAGCTAGAAATAGCAAGTTAAAATAAGGCTAGTCCGTTATCAACTTGAAAAAGTGGCACCGAGTCGGTGCTTTTttGGCCGGCATGGTCCCAGCCTCCTCGCTGGCGCCGGCTGGGCAACATGCTTCGGCATGGCGAATGGGACgcactctaatcgttatcggagtgcttttagattactaatcaaattgcttctactaattgcctatcttccagtgatggaacagcatttgAACGcattggctgcaacaatcagccttgatctggaagaagcaatgaaagctgctgttaagtctccgaatcaggtattgttcctgacaggtgtattcccatccggtaaacgcggatactttgcagttgatctgactcaggaataaattataaattaaggtaagaagattgtaggataagctaatgaaatagaaaaaggatgccgtcacacaacttgtcggcattcttttttgttttattagttgaaaatatagtgaaaaagttgcctaaatatgtatgttaacaaattatttgtcgtaactttgcactccaaatctgtttttaacatatggcactagtgAAACCAGTAACGTTATACGATGTCGCAGAGTATGCCGGTGTCTCTTATCAGACCGTTTCCCGCGTGGTGAACCAGGCCAGCCACGTTTCTGCGAAAACGCGGGAAAAAGTGGAAGCGGCGATGGCGGAGCTGAATTACATTCCCAACCGCGTGGCACAACAACTGGCGGGCAAACAGTCGTTGCTGATTGGCGTTGCCACCTCCAGTCTGGCCCTGCACGCGCCGTCGCAAATTGTCGCGGCGATTAAATCTCGCGCCGATCAACTGGGTGCCAGCGTGGTGGTGTCGATGGTAGAACGAAGCGGCGTCGAAGCCTGTAAAACGGCGGTGCACAATCTTCTCGCGCAACGCGTCAGTGGGCTGATCATTAACTATCCGCTGGATGACCAGGATGCCATTGCTGTGGAAGCTGCCTGCACTAATGTTCCGGCGTTATTTCTTGATGTCTCTGACCAGACACCCATCAACAGTATTATTTTCTCCCATGAAGACGGTACGCGACTGGGCGTGGAGCATCTGGTCGCATTGGGTCACCAGCAAATCGCGCTGTTAGCGGGCCCATTAAGTTCTGTCTCGGCGCGTCTGCGTCTGGCTGGCTGGCATAAATATCTCACTCGCAATCAAATTCAGCCGATAGCGGAACGGGAAGGCGACTGGAGTGCCATGTCCGGTTTTCAACAAACCATGCAAATGCTGAATGAGGGCATCGTTCCCACTGCGATGCTGGTTGCCAACGATCAGATGGCGCTGGGCGCAATGCGCGCCATTACCGAGTCCGGGCTGCGCGTTGGTGCGGATATCTCGGTAGTGGGATACGACGATACCGAAGACAGCTCATGTTATATCCCGCCGTTAACCACCATCAAACAGGATTTTCGCCTGCTGGGGCAAACCAGCGTGGACCGCTTGCTGCAACTCTCTCAGGGCCAGGCGGTGAAGGGCAATCAGCTGTTGCCCGTCTCACTGGTGAAAAGAAAAACCACCCTGGCGCCCAATACGCAAACCGCCTCTCCCCGCGCGTTGGCCGATTCATTAATGCAGCTGGCACGACAGGTTTCCCGACTGGAAAGCGGGCAGtgagctttcctcggtaccaaattccagaaaagaggcctcccgaaaggggggccttttttcgttttggtcctacttgtgcctgttctatttccTATGcggttcctggccttttgctggccttttgctcacatgttctttcctgcgttatcccctgattctgtggataaccgtattaccgcctttgagtgagctgatacATCCcaaatgctgttccatcactggaagataggcaattagtagaagcaatttgattagtaatctaaaagcactccgataacgattagagtgcttttagattgtttatcattaattatcaaagcaagtgcagttcattatgccaggatgcgctcgcacagacgccaacccgtgacaccgttgatggtcacgcggaacagcaggctgccgtccgggttaatcagacgctcgtcaataatcttattgccattccacagggtaccggtgacggtgatctttttaccgtcaaaaacggcaatgccttcatacggacggccgaaatagtcaatcatattcggggtcacaccatcaatcaccagcgtaccgtaatgcaagataaccttgaagtggtgatcgtcaactgggtagacgactttaaagattttctcgatttgacccatttggtcaccgctcaggccttcgtacgggatgatcacatgaatatcaattttcagaccgttctcgccggacagaacgatgcgctgaatcggcgtcacgctcacacccaggttctggaacagagagcttacgccaccctgttccaggacttgatccaaattataacccgcggtctgacgccaatcgccaacaaaatcttccagagtaaaaaccatgtttatattatttatatttgtttgacgagaatatctttatttgccgacaaaggtaCATAACTAAAGTTTCCCACCcaaataaatagatagaaaaataacagtttgtcgaattttctttgtacCCGAtacttgtgcctgttctatttccgaaccgaccgcttgtatgaatccatcaaaattcgttttctctatgttggattccttgttgctcatattgtgatgataatttctacaaatatagtcattggtaactatctatgaaactgtttgatacttttatagttgattaaacttgttcatggcatttgccttaatatcatccgctatgtcaatgtagggtttcatagctttgtagtcgctgtgtcccgtccatttcatgaccacctgtgccgggattccgagagccagcgcattgcagatgaatgtccttcttcctgcatgggtactgagcaaagcgtatttgggtgtgacttcatcaatacgttcatttcccttgtagtaggtttcccgtacaggctcgttgatttctgccagttcgcccagctctttcaggtaatcgttcatcttctggttgctgatgacgggcagagccatgtaattctcgaaatggatgtccttgtatttgtccagtatggctttgctgtatttgttcagttcaatcgtcaggctgtcggcagtcttgactgtggttatttcgatgtggtcggacttcacatcgcttcttttcagattgcgaacatccgaataccgcaaactcgtaaagcagcagaacaggaaaacatcacgcacacgttccaggtattgcttatccttgggtatctggtagtctttcagcttgttcagttcatcccaagtcaggaagattacttttttcgaggtggttttcagtttcggtttgaacgtatcgtatgcaatgttctgatgatgtcctttcctgaagctccagcgcaggaaccatttgaggaatcccatttgcttgccgatggtgctgtttctcatatccttggtgtcacgcaggaagttgacgtattcgttcaatccaaactcgttgaaatagttgaacgttgcatcctccttgaactctttgaggtggttcctcactgctgcaaatttttcataggtggatgccgtccagttattctggttaccgcactcttttacaaactcatcgaacacctcccaaaagctgacaggggcttcttccggctgttcttcactggtgtctttcattctcatgttgaaagcttccttcaactgttgggtcgttggcatgacctcctgcacctcaaattccttgaaaatattctggatttcggcatagtatttcagcaagtccgtattgatttcggctgcactttgctttagcttgttggtacatccgttctttacccgctgcttatctacatcccatttggctacgtcaatccggtagcccgttgtaaactcgatacgttggctggcaaagatgacacgcatacggatgggtacgttctctacgattggcacaccgttctttttccggctctccaatgcaaaaatgatgttgcgcttgatattcataattgggtgcgtttgaaattctacacccaaatatacacccaattattgagatagcaaaagacatttagaaacatttacttttactctatattgtaatttacacttgattatcagtcgtttgcagtTttatgatattctgtgaaagtataagttcgagagcctgtctctccgcaaaaaacgctgaaaatcagcagattgcaaaacaaacaccctgttttacacccaagaatgtaaagtcggctgtttttgttttatttaagataatacaaccactacataataaaagagtagcgatattaaaagaatccgatgagaaaagactaatatttatctatccattcagtttgatttttcaggactttacatcgtcctgaaagtatttgttggtaccggtaccgaggacgcgtaaacatttacagttgcatgtggcctattgtttttagccgttaaatattttataactattaaatagcgatacaaattgttcgaaactaatattgtttatatcatatattctcgcatgttttaaagctttattaaattgattttttgtaaacagtttttcgtactctttgttaacccatttcattacaaaagtttcatatttttttctctctttaaatgccatttttgctggctttctttttaatacaattaatgtgctatccactttaggttttggatggaaataatacctaggaatttttgctaatatagaaatatctacctctgccattaacagcaatgctagtgatctgtttgtatctaataacattttagcaaaaccatattccactattaaataacttattgtggctgaactttcaaaaacaatttttcgaattatatttgtgcttatgttgtaaggtatgctgccaaatattttatatggattgtggctaggaaatgtaaatttcagtatatcatcatttactatttgatagttaggataatttaagagcttattacgagttacctcacataatttagaatcaatttctatcgccgttacaaaattacatctctttaccaatccagcagtaaaatgacctttccctgcacctatttcaaagatgttatctttttcatctaaacttatgcaattcattattttttctatgtgatattttgaagtaataaaattttgactatcttttatatttactttgttcattataacctctccttaatttattgcatctcttttcgaatatttatgttttttgagaaaagaacgtactcatggttcatcccgatatgcgtatcggtctgtatatcagcaactttctatgtgtttcaactacaatagtcatctattctcatctttctgagtccaccccctgcaaagcccctctttacgacataaaaattcggtcggaaaaggtatgcaaaagatgtttctctctttaagagaaactcttcgggatgcaaaaatatgaaaataactccaattcaccaaattatatagcgacttttttacaaaatgctaaaatttgttgatttccgtcaagcaattgttgagcaaaaatgtcttttacgataaaatgatacctcaatatcaactgtttagcaaaacgatatttctcttaaagagagaaacacctttttgttcaccaatccccgacttttaatcccgcggccatgattgaaaaaggaagagtatgagtattcaacatttccgtgtcgcccttattcccttttttgcggcattttgccttcctgtttttgctcacccagaaacgctggtgaaagtaaaagatgctgaagatcagttgggtgcacgagtgggttacatcgaactggatctcaacagcggtaagatccttgagagttttcgccccgaagaacgttttccaatgatgagcacttttaaagttctgctatgtggcgcggtattatcccgtattgacgccgggcaagagcaactcggtcgccgcatacactattctcagaatgacttggttgagtactcaccagtcacagaaaagcatcttacggatggcatgacagtaagagaattatgcagtgctgccataaccatgagtgataacactgcggccaacttacttctgacaacgatcggaggaccgaaggagctaaccgcttttttgcacaacatgggggatcatgtaactcgccttgatcgttgggaaccggagctgaatgaagccataccaaacgacgagcgtgacaccacgatgcctgtagcaatggcaacaacgttgcgcaaactattaactggcgaactacttactctagcttcccggcaacaattaatagactggatggaggcggataaagttgcaggaccacttctgcgctcggcccttccggctggctggtttattgctgataaatctggagccggtgagcgtgggtctcgcggtatcattgcagcactggggccagatggtaagccctcccgtatcgtagttatctacacgacggggagtcaggcaactatggatgaacgaaatagacagatcgctgagataggtgcctcactgattaagcattggtaactgtcagaccaagtttactcataacgcgtcaattcgagggggatcaattccgtgataggtgggctgcccttcctggttggcttggtttcatcagccatccgcttgccctcatctgttacgccggcggtagccggccagcctcgcagagcaggattcccgttgagcaccgccaggtgcgaataagggacagtgaagaaggaacacccgctcgcgggtgggcctacttcacctatcctgcccggctgacgccgttggatacaccaaggaaagtctacacgaaccctttggcaaaatcctgtatatcgtgcgaaaaaggatggatataccgaaaaaatcgctataatgaccccgaagcagggttatgcagcggaaaacggaattgatccggccacgatgcgtccggcgtagaggatctgaagatcagcagttcaacctgttgatagtacgtactaagctctcatgtttcacgtactaagctctcatgtttaacgtactaagctctcatgtttaacgaactaaaccctcatggctaacgtactaagctctcatggctaacgtactaagctctcatgtttcacgtactaagctctcatgtttgaacaataaaattaatataaatcagcaacttaaatagcctctaaggttttaagttttataagaaaaaaaagaatatataaggcttttaaagcttttaag

>pBH503

gtttaacggttgtggacaacaagccagggatgtaacgcactgagaagcccttagagcctctcaaagcaattttgagtgacacaggaacacttaacggctgacatgggaattcccctccaccgcggtggCCCTgtacaaagaaaattcgacaaactgttatttttctatctatttatttgaattgtgagcggataacaattacctttgtcggcaattgtgagcggataacaattATGATCTGTCCTGATGAGTCCGTGAGGACGAAACGAGTAAGCTCGTCGACAGAACGATGCGCTGAATGTTTTAGAGCTAGAAATAGCAAGTTAAAATAAGGCTAGTCCGTTATCAACTTGAAAAAGTGGCACCGAGTCGGTGCTTTTttGGCCGGCATGGTCCCAGCCTCCTCGCTGGCGCCGGCTGGGCAACATGCTTCGGCATGGCGAATGGGACgcactctaatcgttatcggagtgcttttagattactaatcaaattgcttctactaattgcctatcttccagtgatggaacagcatttgAACGcattggctgcaacaatcagccttgatctggaagaagcaatgaaagctgctgttaagtctccgaatcaggtattgttcctgacaggtgtattcccatccggtaaacgcggatactttgcagttgatctgactcaggaataaattataaattaaggtaagaagattgtaggataagctaatgaaatagaaaaaggatgccgtcacacaacttgtcggcattcttttttgttttattagttgaaaatatagtgaaaaagttgcctaaatatgtatgttaacaaattatttgtcgtaactttgcactccaaatctgtttttaacatatggcactagtgAAACCAGTAACGTTATACGATGTCGCAGAGTATGCCGGTGTCTCTTATCAGACCGTTTCCCGCGTGGTGAACCAGGCCAGCCACGTTTCTGCGAAAACGCGGGAAAAAGTGGAAGCGGCGATGGCGGAGCTGAATTACATTCCCAACCGCGTGGCACAACAACTGGCGGGCAAACAGTCGTTGCTGATTGGCGTTGCCACCTCCAGTCTGGCCCTGCACGCGCTGTCGCAAATTGTCGCGGCGATTAAATCTCGCGCCTATCAACTGGGTGCCAGCGTGTTCGTGTCGATGGTAGAACGAAGCGGCATCGAAGCCTGTAAAACGGCGGTGCACAATCTTCTCGCGCAACGCGTCAGTGGGCTGATCATTAACTATCCGCTGGATAACCAGGATGCCATTGCTGTGGAAGCTGCCTGCACTAATGTTCCGGCGTTATTTCTTGATGTCTCTGACCAGACACCCATCAACAGTATTATTTTCTCCCATGAAGACGGTACGCGACTGGGCGTGGAGCATCTGGTCGCATTGGGTCACCAGCAAATCGCGCTGTTAGCGGGCCCATTAAGTTCTGTCTCGGCGCGTCTGCGTCTGGCTGGCTGGCATAAATATCTCACTCGCAATCAAATTCAGCCGATAGCGGAACGGGAAGGCGACTGGAGTGCCATGTCCGGTTTTCAACAAACCATGCAAATGCTGAATGAGGGCATCGTTCCCACTGCGATGCTGGTTGCCAACGATCAGATGGCGCTGGGCGCAATGCGCGCCATTACCGAGACCGGGCTGCGCGTTGGTGCGGATATCTCGGTAGTGGGATACGACGATACCGAAGACAGCTCATGTTATATCCCGCCGTTAACCACCATCAAACAGGATTTTCGCCTGCTGGGGCAAACCAGCGTGGACCGCTTGCTGCAACTCTCTCAGGGCCAGGCGGTGAAGGGCAATCAGCTGTTGCCCGTCTCACTGGTGAAAAGAAAAACCACCCTGGCGCCCAATACGCAAACCGCCTCTCCCCGCGCGTTGGCCGATTCATTAATGCAGCTGGCACGACAGGTTTCCCGACTGGAAAGCGGGCAGtgagctttcctcggtaccaaattccagaaaagaggcctcccgaaaggggggccttttttcgttttggtcctacttgtgcctgttctatttccTATGcggttcctggccttttgctggccttttgctcacatgttctttcctgcgttatcccctgattctgtggataaccgtattaccgcctttgagtgagctgatacATCCcaaatgctgttccatcactggaagataggcaattagtagaagcaatttgattagtaatctaaaagcactccgataacgattagagtgcttttagattgtttatcattaattatcaaagcaagtgcagttcattatgccaggatgcgctcgcacagacgccaacccgtgacaccgttgatggtcacgcggaacagcaggctgccgtccgggttaatcagacgctcgtcaataatcttattgccattccacagggtaccggtgacggtgatctttttaccgtcaaaaacggcaatgccttcatacggacggccgaaatagtcaatcatattcggggtcacaccatcaatcaccagcgtaccgtaatgcaagataaccttgaagtggtgatcgtcaactgggtagacgactttaaagattttctcgatttgacccatttggtcaccgctcaggccttcgtacgggatgatcacatgaatatcaattttcagaccgttctcgccggacagaacgatgcgctgaatcggcgtcacgctcacacccaggttctggaacagagagcttacgccaccctgttccaggacttgatccaaattataacccgcggtctgacgccaatcgccaacaaaatcttccagagtaaaaaccatgtttatattatttatatttgtttgacgagaatatctttatttgccgacaaaggtaCATAACTAAAGTTTCCCACCcaaataaatagatagaaaaataacagtttgtcgaattttctttgtacCCGAtacttgtgcctgttctatttccgaaccgaccgcttgtatgaatccatcaaaattcgttttctctatgttggattccttgttgctcatattgtgatgataatttctacaaatatagtcattggtaactatctatgaaactgtttgatacttttatagttgattaaacttgttcatggcatttgccttaatatcatccgctatgtcaatgtagggtttcatagctttgtagtcgctgtgtcccgtccatttcatgaccacctgtgccgggattccgagagccagcgcattgcagatgaatgtccttcttcctgcatgggtactgagcaaagcgtatttgggtgtgacttcatcaatacgttcatttcccttgtagtaggtttcccgtacaggctcgttgatttctgccagttcgcccagctctttcaggtaatcgttcatcttctggttgctgatgacgggcagagccatgtaattctcgaaatggatgtccttgtatttgtccagtatggctttgctgtatttgttcagttcaatcgtcaggctgtcggcagtcttgactgtggttatttcgatgtggtcggacttcacatcgcttcttttcagattgcgaacatccgaataccgcaaactcgtaaagcagcagaacaggaaaacatcacgcacacgttccaggtattgcttatccttgggtatctggtagtctttcagcttgttcagttcatcccaagtcaggaagattacttttttcgaggtggttttcagtttcggtttgaacgtatcgtatgcaatgttctgatgatgtcctttcctgaagctccagcgcaggaaccatttgaggaatcccatttgcttgccgatggtgctgtttctcatatccttggtgtcacgcaggaagttgacgtattcgttcaatccaaactcgttgaaatagttgaacgttgcatcctccttgaactctttgaggtggttcctcactgctgcaaatttttcataggtggatgccgtccagttattctggttaccgcactcttttacaaactcatcgaacacctcccaaaagctgacaggggcttcttccggctgttcttcactggtgtctttcattctcatgttgaaagcttccttcaactgttgggtcgttggcatgacctcctgcacctcaaattccttgaaaatattctggatttcggcatagtatttcagcaagtccgtattgatttcggctgcactttgctttagcttgttggtacatccgttctttacccgctgcttatctacatcccatttggctacgtcaatccggtagcccgttgtaaactcgatacgttggctggcaaagatgacacgcatacggatgggtacgttctctacgattggcacaccgttctttttccggctctccaatgcaaaaatgatgttgcgcttgatattcataattgggtgcgtttgaaattctacacccaaatatacacccaattattgagatagcaaaagacatttagaaacatttacttttactctatattgtaatttacacttgattatcagtcgtttgcagtTttatgatattctgtgaaagtataagttcgagagcctgtctctccgcaaaaaacgctgaaaatcagcagattgcaaaacaaacaccctgttttacacccaagaatgtaaagtcggctgtttttgttttatttaagataatacaaccactacataataaaagagtagcgatattaaaagaatccgatgagaaaagactaatatttatctatccattcagtttgatttttcaggactttacatcgtcctgaaagtatttgttggtaccggtaccgaggacgcgtaaacatttacagttgcatgtggcctattgtttttagccgttaaatattttataactattaaatagcgatacaaattgttcgaaactaatattgtttatatcatatattctcgcatgttttaaagctttattaaattgattttttgtaaacagtttttcgtactctttgttaacccatttcattacaaaagtttcatatttttttctctctttaaatgccatttttgctggctttctttttaatacaattaatgtgctatccactttaggttttggatggaaataatacctaggaatttttgctaatatagaaatatctacctctgccattaacagcaatgctagtgatctgtttgtatctaataacattttagcaaaaccatattccactattaaataacttattgtggctgaactttcaaaaacaatttttcgaattatatttgtgcttatgttgtaaggtatgctgccaaatattttatatggattgtggctaggaaatgtaaatttcagtatatcatcatttactatttgatagttaggataatttaagagcttattacgagttacctcacataatttagaatcaatttctatcgccgttacaaaattacatctctttaccaatccagcagtaaaatgacctttccctgcacctatttcaaagatgttatctttttcatctaaacttatgcaattcattattttttctatgtgatattttgaagtaataaaattttgactatcttttatatttactttgttcattataacctctccttaatttattgcatctcttttcgaatatttatgttttttgagaaaagaacgtactcatggttcatcccgatatgcgtatcggtctgtatatcagcaactttctatgtgtttcaactacaatagtcatctattctcatctttctgagtccaccccctgcaaagcccctctttacgacataaaaattcggtcggaaaaggtatgcaaaagatgtttctctctttaagagaaactcttcgggatgcaaaaatatgaaaataactccaattcaccaaattatatagcgacttttttacaaaatgctaaaatttgttgatttccgtcaagcaattgttgagcaaaaatgtcttttacgataaaatgatacctcaatatcaactgtttagcaaaacgatatttctcttaaagagagaaacacctttttgttcaccaatccccgacttttaatcccgcggccatgattgaaaaaggaagagtatgagtattcaacatttccgtgtcgcccttattcccttttttgcggcattttgccttcctgtttttgctcacccagaaacgctggtgaaagtaaaagatgctgaagatcagttgggtgcacgagtgggttacatcgaactggatctcaacagcggtaagatccttgagagttttcgccccgaagaacgttttccaatgatgagcacttttaaagttctgctatgtggcgcggtattatcccgtattgacgccgggcaagagcaactcggtcgccgcatacactattctcagaatgacttggttgagtactcaccagtcacagaaaagcatcttacggatggcatgacagtaagagaattatgcagtgctgccataaccatgagtgataacactgcggccaacttacttctgacaacgatcggaggaccgaaggagctaaccgcttttttgcacaacatgggggatcatgtaactcgccttgatcgttgggaaccggagctgaatgaagccataccaaacgacgagcgtgacaccacgatgcctgtagcaatggcaacaacgttgcgcaaactattaactggcgaactacttactctagcttcccggcaacaattaatagactggatggaggcggataaagttgcaggaccacttctgcgctcggcccttccggctggctggtttattgctgataaatctggagccggtgagcgtgggtctcgcggtatcattgcagcactggggccagatggtaagccctcccgtatcgtagttatctacacgacggggagtcaggcaactatggatgaacgaaatagacagatcgctgagataggtgcctcactgattaagcattggtaactgtcagaccaagtttactcataacgcgtcaattcgagggggatcaattccgtgataggtgggctgcccttcctggttggcttggtttcatcagccatccgcttgccctcatctgttacgccggcggtagccggccagcctcgcagagcaggattcccgttgagcaccgccaggtgcgaataagggacagtgaagaaggaacacccgctcgcgggtgggcctacttcacctatcctgcccggctgacgccgttggatacaccaaggaaagtctacacgaaccctttggcaaaatcctgtatatcgtgcgaaaaaggatggatataccgaaaaaatcgctataatgaccccgaagcagggttatgcagcggaaaacggaattgatccggccacgatgcgtccggcgtagaggatctgaagatcagcagttcaacctgttgatagtacgtactaagctctcatgtttcacgtactaagctctcatgtttaacgtactaagctctcatgtttaacgaactaaaccctcatggctaacgtactaagctctcatggctaacgtactaagctctcatgtttcacgtactaagctctcatgtttgaacaataaaattaatataaatcagcaacttaaatagcctctaaggttttaagttttataagaaaaaaaagaatatataaggcttttaaagcttttaag

>pBH504

gatgtaacgcactgagaagcccttagagcctctcaaagcaattttgagtgacacaggaacacttaacggctgacatgggaattcccctccaccgcggtgtattaatgcggctgcCCCTgtacaaagaaaattcgacaaactgttatttttctatctatttatttgaattgtgagcggataacaattacctttgtcggcaattgtgagcggataacaattATGAtgttgtCTGATGAGTCCGTGAGGACGAAACGAGTAAGCTCGTCacaacattggcaccgataacGTTTTAGAGCTAGAAATAGCAAGTTAAAATAAGGCTAGTCCGTTATCAACTTGAAAAAGTGGCACCGAGTCGGTGCTTTTttGGCCGGCATGGTCCCAGCCTCCTCGCTGGCGCCGGCTGGGCAACATGCTTCGGCATGGCGAATGGGACgcactctaatcgttatcggagtgcttttagattactaatcaaattgcttctactaattgcctatcttccagtgatggaacagcatttgAACGcattggctgcaacaatcagccttgatctggaagaagcaatgaaagctgctgttaagtctccgaatcaggtattgttcctgacaggtgtattcccatccggtaaacgcggatactttgcagttgatctgactcaggaataaattataaattaaggtaagaagattgtaggataagctaatgaaatagaaaaaggatgccgtcacacaacttgtcggcattcttttttgttttattagttgaaaatatagtgaaaaagttgcctaaatatgtatgttaacaaattatttgtcgtaactttgcactccaaatctgtttttaacatatggcactagtgAAACCAGTAACGTTATACGATGTCGCAGAGTATGCCGGTGTCTCTTATCAGACCGTTTCCCGCGTGGTGAACCAGGCCAGCCACGTTTCTGCGAAAACGCGGGAAAAAGTGGAAGCGGCGATGGCGGAGCTGAATTACATTCCCAACCGCGTGGCACAACAACTGGCGGGCAAACAGTCGTTGCTGATTGGCGTTGCCACCTCCAGTCTGGCCCTGCACGCGCCGTCGCAAATTGTCGCGGCGATTAAATCTCGCGCCGATCAACTGGGTGCCAGCGTGGTGGTGTCGATGGTAGAACGAAGCGGCGTCGAAGCCTGTAAAACGGCGGTGCACAATCTTCTCGCGCAACGCGTCAGTGGGCTGATCATTAACTATCCGCTGGATGACCAGGATGCCATTGCTGTGGAAGCTGCCTGCACTAATGTTCCGGCGTTATTTCTTGATGTCTCTGACCAGACACCCATCAACAGTATTATTTTCTCCCATGAAGACGGTACGCGACTGGGCGTGGAGCATCTGGTCGCATTGGGTCACCAGCAAATCGCGCTGTTAGCGGGCCCATTAAGTTCTGTCTCGGCGCGTCTGCGTCTGGCTGGCTGGCATAAATATCTCACTCGCAATCAAATTCAGCCGATAGCGGAACGGGAAGGCGACTGGAGTGCCATGTCCGGTTTTCAACAAACCATGCAAATGCTGAATGAGGGCATCGTTCCCACTGCGATGCTGGTTGCCAACGATCAGATGGCGCTGGGCGCAATGCGCGCCATTACCGAGTCCGGGCTGCGCGTTGGTGCGGATATCTCGGTAGTGGGATACGACGATACCGAAGACAGCTCATGTTATATCCCGCCGTTAACCACCATCAAACAGGATTTTCGCCTGCTGGGGCAAACCAGCGTGGACCGCTTGCTGCAACTCTCTCAGGGCCAGGCGGTGAAGGGCAATCAGCTGTTGCCCGTCTCACTGGTGAAAAGAAAAACCACCCTGGCGCCCAATACGCAAACCGCCTCTCCCCGCGCGTTGGCCGATTCATTAATGCAGCTGGCACGACAGGTTTCCCGACTGGAAAGCGGGCAGtgagctttcctcggtaccaaattccagaaaagaggcctcccgaaaggggggccttttttcgttttggtcctacttgtgcctgttctatttccTATGcggttcctggccttttgctggccttttgctcacatgttctttcctgcgttatcccctgattctgtggataaccgtattaccgcctttgagtgagctgatacatcccaaatgctgctcggtaccaaagacgaaCaataagacgctgaaaagcgtcttttttcgttttggtccgataaagtttggaagataaagctaaaagttcttatctttgcagtccgaaataaagacatataaaagaaaagacaccatggataagaaatactcaataggcttagctatcggcacaaatagcgtcggatgggcggtgatcactgatgaatataaggttccgtctaaaaagttcaaggttctgggaaatacagaccgccacagtatcaaaaaaaatcttataggggctcttttatttgacagtggagagacagcggaagcgactcgtctcaaacggacagctcgtagaaggtatacacgtcggaagaatcgtatttgttatctacaggagattttttcaaatgagatggcgaaagtagatgatagtttctttcatcgacttgaagagtcttttttggtggaagaagacaagaagcatgaacgtcatcctatttttggaaatatagtagatgaagttgcttatcatgagaaatatccaactatctatcatctgcgaaaaaaattggtagattctactgataaagcggatttgcgcttaatctatttggccttagcgcatatgattaagtttcgtggtcattttttgattgagggagatttaaatcctgataatagtgatgtggacaaactatttatccagttggtacaaacctacaatcaattatttgaagaaaaccctattaacgcaagtggagtagatgctaaagcgattctttctgcacgattgagtaaatcaagacgattagaaaatctcattgctcagctccccggtgagaagaaaaatggcttatttgggaatctcattgctttgtcattgggtttgacccctaattttaaatcaaattttgatttggcagaagatgctaaattacagctttcaaaagatacttacgatgatgatttagataatttattggcgcaaattggagatcaatatgctgatttgtttttggcagctaagaatttatcagatgctattttactttcagatatcctaagagtaaatactgaaataactaaggctcccctatcagcttcaatgattaaacgctacgatgaacatcatcaagacttgactcttttaaaagctttagttcgacaacaacttccagaaaagtataaagaaatcttttttgatcaatcaaaaaacggatatgcaggttatattgatgggggagctagccaagaagaattttataaatttatcaaaccaattttagaaaaaatggatggtactgaggaattattggtgaaactaaatcgtgaagatttgctgcgcaagcaacggacctttgacaacggctctattccccatcaaattcacttgggtgagctgcatgctattttgagaagacaagaagacttttatccatttttaaaagacaatcgtgagaagattgaaaaaatcttgacttttcgaattccttattatgttggtccattggcgcgtggcaatagtcgttttgcatggatgactcggaagtctgaagaaacaattaccccatggaattttgaagaagttgtcgataaaggtgcttcagctcaatcatttattgaacgcatgacaaactttgataaaaatcttccaaatgaaaaagtactaccaaaacatagtttgctttatgagtattttacggtttataacgaattgacaaaggtcaaatatgttactgaaggaatgcgaaaaccagcatttctttcaggtgaacagaagaaagccattgttgatttactcttcaaaacaaatcgaaaagtaaccgttaagcaattaaaagaagattatttcaaaaaaatagaatgttttgatagtgttgaaatttcaggagttgaagatagatttaatgcttcattaggtacctaccatgatttgctaaaaattattaaagataaagattttttggataatgaagaaaatgaagatatcttagaggatattgttttaacattgaccttatttgaagatagggagatgattgaggaaagacttaaaacatatgctcacctctttgatgataaggtgatgaaacagcttaaacgtcgccgttatactggttggggacgtttgtctcgaaaattgattaatggtattagggataagcaatctggcaaaacaatattagattttttgaaatcagatggttttgccaatcgcaattttatgcagctgatccatgatgatagtttgacatttaaagaagacattcaaaaagcacaagtgtctggacaaggcgatagtttacatgaacatattgcaaatttagctggtagccctgctattaaaaaaggtattttacagactgtaaaagttgttgatgaattggtcaaagtaatggggcggcataagccagaaaatatcgttattgaaatggcacgtgaaaatcagacaactcaaaagggccagaaaaattcgcgagagcgtatgaaacgaatcgaagaaggtatcaaagaattaggaagtcagattcttaaagagcatcctgttgaaaatactcaattgcaaaatgaaaagctctatctctattatctccaaaatggaagagacatgtatgtggaccaagaattagatattaatcgtttaagtgattatgatgtcgatgccattgttccacaaagtttccttaaagacgattcaatagacaataaggtcttaacgcgttctgataaaaatcgtggtaaatcggataacgttccaagtgaagaagtagtcaaaaagatgaaaaactattggagacaacttctaaacgccaagttaatcactcaacgtaagtttgataatttaacgaaagctgaacgtggtggtttgagtgaacttgataaagctggttttatcaaacgccaattggttgaaactcgccaaatcactaagcatgtggcacaaattttggatagtcgcatgaatactaaatacgatgaaaatgataaacttattcgagaggttaaagtgattaccttaaaatctaaattagtttctgacttccgaaaagatttccaattctataaagtacgtgagattaacaattaccatcatgcccatgatgcgtatctaaatgccgtcgttggaactgctttgattaagaaatatccaaaacttgaatcggagtttgtctatggtgattataaagtttatgatgttcgtaaaatgattgctaagtctgagcaagaaataggcaaagcaaccgcaaaatatttcttttactctaatatcatgaacttcttcaaaacagaaattacacttgcaaatggagagattcgcaaacgccctctaatcgaaactaatggggaaactggagaaattgtctgggataaagggcgagattttgccacagtgcgcaaagtattgtccatgccccaagtcaatattgtcaagaaaacagaagtacagacaggcgaattctccaaggagtcaattttaccaaaaagaaattcggacaagcttattgctcgtaaaaaagactgggatccaaaaaaatatggtggttttgatagtccaacggtagcttattcagtcctagtggttgctaaggtggaaaaagggaaatcgaagaagttaaaatccgttaaagagttactagggatcacaattatggaaagaagttcctttgaaaaaaatccgattgactttttagaagctaaaggatataaggaagttaaaaaagacttaatcattaaactacctaaatatagtctttttgagttagaaaacggtcgtaaacggatgctggctagtgccggagaattacaaaaaggaaatgagctggctctgccaagcaaatatgtgaattttttatatttagctagtcattatgaaaagttgaagggtagtccagaagataacgaacaaaaacaattgtttgtggagcagcataagcattatttagatgagattattgagcaaatcagtgaattttctaagcgtgttattttagcagatgccaatttagataaagttcttagtgcatataacaaacatagagacaaaccaatacgtgaacaagcagaaaatattattcatttatttacgttgacgaatcttggagctcccgctgcttttaaatattttgatacaacaattgatcgtaaacgatatacgtctacaaaagaagttttagatgccactcttatccatcaatccatcactggtctttatgaaacacgcattgatttgagtcagctaggaggtgactgattctttgtacCCGAtaattgcctatcttccagtgatggaacagcatttgtgcattggctgcaacaatcagccttacttgtgcctgttctatttccgaaccgaccgcttgtatgaatGcatcaaaattcgttttctctaCgttggattccttgttgctcatattgtgatgataatttctacaaatatagtcattggtaactatctatgaaactgtttgatacttttatcagtctaatagttttacaaggtctttcttcatttcttcgtcaatatccctgtatcgtctgaaagctttgcttccctccttgtgtcccgacagtgcggaaacaaggttcgggtctttcacttttttatagatattgccgataaacgtacgtcttgccagatggctgcttgccacttcataaataggtcgtttgatttcgttgtgcgtcaacgggtctaagattgttacgatgcggtcaactccagctaatttgaatatcttttttatggcatcattgtacttttgctcggatatgaacggcaacagttttccctcatattctttgtagcgttcaaggatttctttcgctttgtcgttaagtggaacacgtaccgtaaccggattcccctctttggttttcttgggaatatattctatggcttcattgaccacatttagtttggtcattcggtacaggtcgctcaccctgcatcctatcagtgtctgaaatatgaatatatccctctgtattgccagttgtggggtggcagaaaggtctgcattaaaaatcctgtccctttcttcgagtgttatataataaggtgtaccatatgtacactcctctatcggaaacttgtcgaaaggtctgtttgtggtgcgtttgttatcgaagcaccacaggaagaatgtgcgtattcttgaaaaacagtctatcagcgtgtttttgcttctgggctgtggtgtcctcttttcgggaatggcttcataaatgctcgggtaaagttcataatactggtattcgttctgaaagaaatcccacatatcccgaagcgtgtcaggtgttaccaaatccacatcaaggataaagcccttttgtcctctctttgtagcccttacatatagttcataacgcagtaaggctcttttgacaacccggaaatttttctttcgtacttccgacaaagggtgcttgtttagaaattcatcgaatagttctccaatggtaggcttgataaccacttcctccggcaaaaaatatttttcaggatggtaaaatttatcaagtgttgtttttagccattctttgtctattgcttccttttcttgttgatataccttctcgatataggttttcaactggcgtatctcttcatttatatgggtacgcatttcttcattgcagacagctttcgtttttacacattcgtctttatcatcccatagattgggattgatggctaactgagtaggagcaacggagtctaactgccttccgtttctaaaacggacatagattgtagccatggattctgtatcatatcgtttggctgcttttttaatgataaaggttactttcatagactttcaggttgaattttactctgctgcaaatataaatattttccccagcattttccccacatctgctaaatattttgcaattcgattaaacttggattaaaatttaataggattataacatattgaaatacagtgtaattgtggcgtttttccgcatttttctttttacccacattttccccactttaattatttcaaatgcggggtctgggtacaagaaagaaagctaagtatttgatagttcaatacttagctttttcttttgcttgaattttccccacattttccccacacgtgcaaaaaatatagcagtaagtcattatttcttttggttgaacgtagagagtagcgatattaaaagaatccgatgagaaaagactaatatttatctatccattcagtttgatttttcaggactttacatcgtcctgaaagtatttgttggtaccggtaccgaggacgcgtaaacatttacaGTTGCATGTGGCCTATTGTTTaggacgcgttatctccttaacgtacgttttcgttccattggccctcaaaccccgttatatacattcatgtccatttatgtaaaaaatcctgctgaccttgtttatgtcttgtcagtcaccatttgcaaaaccatatttgaccctcaaagaggctgaatttgataagcaacttgctacatactcataataaggagctaaatagaacacgaatgggaaatactcaaatgccaaactaaagaagatattggccaaaataaacgctataccgagagagaaacttgatttttcaacttcctaaccaacagtgttgttcaaacatttctacttatttgtacttaccagttgaacctacgtttccctaataaaatgtctatggtaaaaagttaaaaaatcctcctacttttgttagatatatttttttgtgtaattttgtaatcgttatgcggcagtaataatatacatattaatacgagttaggaatcctgtagttctcatatgctacgaggaggtattaaaaggtgcgtttcgacaatgcatctattgtagtatattattgcttaatccaaatgaatattataaatttaggaattcttgctcacattgatgcaggaaaaacttccgtaaccgagaatctgctgtttgccagtggagcaacggaaaagtgcggctgtgtggataatggtgacaccataacggactctatggatatagagaaacgtagaggaattactgttcgggcttctacgacatctattatctggaatggtgtgaaatgcaatatcattgacactccgggacacatggattttattgcggaagtggagcggacattcaaaatgcttgatggagcagtcctcatcttatccgcaaaggaaggcatacaagcgcagacaaagttgctgttcaatactttacagaagctgcaaatcccgacaattatatttatcaataagattgaccgagccggtgtgaatttggagcgtttgtatctggatataaaagcaaatctgtctcaagatgtcctgtttatgcaaaatgttgtcgatggatcggtttatccggtttgctcccaaacatatataaaggaagaatacaaagaatttgtatgcaaccatgacgacaatatattagaacgatatttggcggatagcgaaatttcaccggctgattattggaatacgataatcgctcttgtggcaaaagccaaagtctatccggtgctacatggatcagcaatgttcaatatcggtatcaatgagttgttggacgccatcacttcttttatacttcctccggcatcggtctcaaacagactttcatcttatctttataagatagagcatgaccccaaaggacataaaagaagttttctaaaaataattgacggaagtctgagacttcgagacgttgtaagaatcaacgattcggaaaaattcatcaagattaaaaatctaaaaactatcaatcagggcagagagataaatgttgatgaagtgggcgccaatgatatcgcgattgtagaggatatggatgattttcgaatcggaaattatttaggtgctgaaccttgtttgattcaaggattatcgcatcagcatcccgctctcaaatcctccgtccggccagacaggcccgaagagagaagcaaggtgatatccgctctgaatacattgtggattgaagacccgtctttgtccttttccataaactcatatagtgatgaattggaaatctcgttatatggtttaacccaaaaggaaatcatacagacattgctggaagaacgattttccgtaaaggtccattttgatgagatcaagactatatacaaagaacgacctgtaaaaaaggtcaataagattattcagatcgaagtgccgcccaacccttattgggccacaatagggctgactcttgaacccttaccgttagggacagggttgcaaatcgaaagtgacatctcctatggttatctgaaccattcttttcaaaatgccgtttttgaagggattcgtatgtcttgccaatccgggttacatggatgggaagtgactgatctgaaagtaacttttactcaagccgagtattatagcccggtaagtacacctgctgatttcagacagctgaccccttatgtcttcaggctggccttgcaacagtcaggtgtggacattctcgaaccgatgctctattttgagttgcagataccccaagcggcaagttccaaagctattacagatttgcaaaaaatgatgtctgagattgaagacatcagttgcaataatgagtggtgtcatattaaagggaaagttccattaaatacaagtaaagactatgcatcagaagtaagttcatacactaagggcttaggcatttttatggttaagccatgcgggtatcaaataacaaaaggcggttattctgataatatccgcatgaacgaaaaagataaacttttattcatgttccaaaaatcaatgtcatcaaaataatggtataacctctccttaatttattgcatctcttttcgaatatttatgttttttgagaaaagaacgtactcatggttcatcccgatatgcgtatcggtctgtatatcagcaactttctatgtgtttcaactacaatagtcatctattctcatctttctgagtccaccccctgcaaagcccctctttacgacataaaaattcggtcggaaaaggtatgcaaaagatgtttctctctttaagagaaactcttcgggatgcaaaaatatgaaaataactccaattcaccaaattatatagcgacttttttacaaaatgctaaaatttgttgatttccgtcaagcaattgttgagcaaaaatgtcttttacgataaaatgatacctcaatatcaactgtttagcaaaacgatatttctcttaaagagagaaacacctttttgttcaccaatccccgacttttaatcccgcggccatgattgaaaaaggaagagtatgagtattcaacatttccgtgtcgcccttattcccttttttgcggcattttgccttcctgtttttgctcacccagaaacgctggtgaaagtaaaagatgctgaagatcagttgggtgcacgagtgggttacatcgaactggatctcaacagcggtaagatccttgagagttttcgccccgaagaacgttttccaatgatgagcacttttaaagttctgctatgtggcgcggtattatcccgtattgacgccgggcaagagcaactcggtcgccgcatacactattctcagaatgacttggttgagtactcaccagtcacagaaaagcatcttacggatggcatgacagtaagagaattatgcagtgctgccataaccatgagtgataacactgcggccaacttacttctgacaacgatcggaggaccgaaggagctaaccgcttttttgcacaacatgggggatcatgtaactcgccttgatcgttgggaaccggagctgaatgaagccataccaaacgacgagcgtgacaccacgatgcctgtagcaatggcaacaacgttgcgcaaactattaactggcgaactacttactctagcttcccggcaacaattaatagactggatggaggcggataaagttgcaggaccacttctgcgctcggcccttccggctggctggtttattgctgataaatctggagccggtgagcgtgggtcacgcggtatcattgcagcactggggccagatggtaagccctcccgtatcgtagttatctacacgacggggagtcaggcaactatggatgaacgaaatagacagatcgctgagataggtgcctcactgattaagcattggtaactgtcagaccaagtttactcataacgcgtcaattcgagggggatcaattccgtgataggtgggctgcccttcctggttggcttggtttcatcagccatccgcttgccctcatctgttacgccggcggtagccggccagcctcgcagagcaggattcccgttgagcaccgccaggtgcgaataagggacagtgaagaaggaacacccgctcgcgggtgggcctacttcacctatcctgcccggctgacgccgttggatacaccaaggaaagtctacacgaaccctttggcaaaatcctgtatatcgtgcgaaaaaggatggatataccgaaaaaatcgctataatgaccccgaagcagggttatgcagcggaaaacggaattgatccggccacgatgcgtccggcgtagaggatctgaagatcagcagttcaacctgttgatagtacgtactaagctctcatgtttcacgtactaagctctcatgtttaacgtactaagctctcatgtttaacgaactaaaccctcatggctaacgtactaagctctcatggctaacgtactaagctctcatgtttcacgtactaagctctcatgtttgaacaataaaattaatataaatcagcaacttaaatagcctctaaggttttaagttttataagaaaaaaaagaatatataaggcttttaaagcttttaaggtttaacggttgtggacaacaagccagg

>pBH505

gatgtaacgcactgagaagcccttagagcctctcaaagcaattttgagtgacacaggaacacttaacggctgacatgggaattcccctccaccgcggtgtattaatgcggctgcCCCTgtacaaagaaaattcgacaaactgttatttttctatctatttatttgaattgtgagcggataacaattacctttgtcggcaattgtgagcggataacaattATGAtgttgtCTGATGAGTCCGTGAGGACGAAACGAGTAAGCTCGTCacaacattggcaccgataacGTTTTAGAGCTAGAAATAGCAAGTTAAAATAAGGCTAGTCCGTTATCAACTTGAAAAAGTGGCACCGAGTCGGTGCTTTTttGGCCGGCATGGTCCCAGCCTCCTCGCTGGCGCCGGCTGGGCAACATGCTTCGGCATGGCGAATGGGACgcactctaatcgttatcggagtgcttttagattactaatcaaattgcttctactaattgcctatcttccagtgatggaacagcatttgAACGcattggctgcaacaatcagccttgatctggaagaagcaatgaaagctgctgttaagtctccgaatcaggtattgttcctgacaggtgtattcccatccggtaaacgcggatactttgcagttgatctgactcaggaataaattataaattaaggtaagaagattgtaggataagctaatgaaatagaaaaaggatgccgtcacacaacttgtcggcattcttttttgttttattagttgaaaatatagtgaaaaagttgcctaaatatgtatgttaacaaattatttgtcgtaactttgcactccaaatctgtttttaacatatggcactagtgAAACCAGTAACGTTATACGATGTCGCAGAGTATGCCGGTGTCTCTTATCAGACCGTTTCCCGCGTGGTGAACCAGGCCAGCCACGTTTCTGCGAAAACGCGGGAAAAAGTGGAAGCGGCGATGGCGGAGCTCAATTACATTCCCAACCGCGTGGCACAACAACTGGCGGGCAAAGCGTCGCATACCATTGGCATGTTGATCACTGCCAGTACCAATCCTTTCTATTCAGAACTGGTGCGTGGCGTTGAACGCAGCTGCTTCGAACGCGGTTATAGTCTCGTCCTTTGCAATACCGAAGGCGATGAACAGCGGATGAATCGCAATCTGGAAACGCTGATGCAAAAACGCGTTGATGGCTTGCTGTTACTGTGCACCGAAACGCATCAACCTTCGCGTGAAATCATGCAACGTTATCCGACAGTGCCTACTGTGATGATGGACTGGGCTCCGTTCGATGGCGACAGCGATCTTATTCAGGATAACTCGTTGCTGGGCGGAGACTTAGCAACGCAATATCTGATCGATAAAGGTCATACCCGTATCGCCTGTATTACCGGCCCGCTGGATAAAACTCCGGCGCGCCTGCGGTTGGAAGGTTATCGGGCGGCGATGAAACGTGCGGGTCTCAACATTCCTGATGGCTATGAAGTCACTGGTGATTTTGAATTTAACGGCGGGTTTGACGCTATGCGCCAACTGCTATCACATCCGCTGCGTCCTCAGGCCGTCTTTACCGGAAATGACGCTATGGCTGTTGGCGTTTACCAGGCGTTATATCAGGCAGAGTTACAGGTTCCGCAGGATATCGCGGTGATTGGCTATGACGATATCGAACTGGCAAGCTTTATGACGCCACCATTAACCACTATCCACCAACCGAAAGATGAACTGGGGGAGCTGGCGATTGATGTACTCATCCATCGGATAACCCAGCCGACCCTTCAGCAACAACGATTACAACTTACTCCGATTCTGATGGAACGCGGTTCGGCTTAGCTGGTGAAAAGAAAAACCACCCTGGCGCCCAATACGCAAACCGCCTCTCCCCGCGCGTTGGCCGATTCATTAATGCAGCTGGCACGACAGGTTTCCCGACTGGAAAGCGGGCAGTGAgctttcctcggtaccaaattccagaaaagaggcctcccgaaaggggggccttttttcgttttggtcctacttgtgcctgttctatttccTATGcggttcctggccttttgctggccttttgctcacatgttctttcctgcgttatcccctgattctgtggataaccgtattaccgcctttgagtgagctgatacatcccaaatgctgctcggtaccaaagacgaaCaataagacgctgaaaagcgtcttttttcgttttggtccgataaagtttggaagataaagctaaaagttcttatctttgcagtccgaaataaagacatataaaagaaaagacaccatggataagaaatactcaataggcttagctatcggcacaaatagcgtcggatgggcggtgatcactgatgaatataaggttccgtctaaaaagttcaaggttctgggaaatacagaccgccacagtatcaaaaaaaatcttataggggctcttttatttgacagtggagagacagcggaagcgactcgtctcaaacggacagctcgtagaaggtatacacgtcggaagaatcgtatttgttatctacaggagattttttcaaatgagatggcgaaagtagatgatagtttctttcatcgacttgaagagtcttttttggtggaagaagacaagaagcatgaacgtcatcctatttttggaaatatagtagatgaagttgcttatcatgagaaatatccaactatctatcatctgcgaaaaaaattggtagattctactgataaagcggatttgcgcttaatctatttggccttagcgcatatgattaagtttcgtggtcattttttgattgagggagatttaaatcctgataatagtgatgtggacaaactatttatccagttggtacaaacctacaatcaattatttgaagaaaaccctattaacgcaagtggagtagatgctaaagcgattctttctgcacgattgagtaaatcaagacgattagaaaatctcattgctcagctccccggtgagaagaaaaatggcttatttgggaatctcattgctttgtcattgggtttgacccctaattttaaatcaaattttgatttggcagaagatgctaaattacagctttcaaaagatacttacgatgatgatttagataatttattggcgcaaattggagatcaatatgctgatttgtttttggcagctaagaatttatcagatgctattttactttcagatatcctaagagtaaatactgaaataactaaggctcccctatcagcttcaatgattaaacgctacgatgaacatcatcaagacttgactcttttaaaagctttagttcgacaacaacttccagaaaagtataaagaaatcttttttgatcaatcaaaaaacggatatgcaggttatattgatgggggagctagccaagaagaattttataaatttatcaaaccaattttagaaaaaatggatggtactgaggaattattggtgaaactaaatcgtgaagatttgctgcgcaagcaacggacctttgacaacggctctattccccatcaaattcacttgggtgagctgcatgctattttgagaagacaagaagacttttatccatttttaaaagacaatcgtgagaagattgaaaaaatcttgacttttcgaattccttattatgttggtccattggcgcgtggcaatagtcgttttgcatggatgactcggaagtctgaagaaacaattaccccatggaattttgaagaagttgtcgataaaggtgcttcagctcaatcatttattgaacgcatgacaaactttgataaaaatcttccaaatgaaaaagtactaccaaaacatagtttgctttatgagtattttacggtttataacgaattgacaaaggtcaaatatgttactgaaggaatgcgaaaaccagcatttctttcaggtgaacagaagaaagccattgttgatttactcttcaaaacaaatcgaaaagtaaccgttaagcaattaaaagaagattatttcaaaaaaatagaatgttttgatagtgttgaaatttcaggagttgaagatagatttaatgcttcattaggtacctaccatgatttgctaaaaattattaaagataaagattttttggataatgaagaaaatgaagatatcttagaggatattgttttaacattgaccttatttgaagatagggagatgattgaggaaagacttaaaacatatgctcacctctttgatgataaggtgatgaaacagcttaaacgtcgccgttatactggttggggacgtttgtctcgaaaattgattaatggtattagggataagcaatctggcaaaacaatattagattttttgaaatcagatggttttgccaatcgcaattttatgcagctgatccatgatgatagtttgacatttaaagaagacattcaaaaagcacaagtgtctggacaaggcgatagtttacatgaacatattgcaaatttagctggtagccctgctattaaaaaaggtattttacagactgtaaaagttgttgatgaattggtcaaagtaatggggcggcataagccagaaaatatcgttattgaaatggcacgtgaaaatcagacaactcaaaagggccagaaaaattcgcgagagcgtatgaaacgaatcgaagaaggtatcaaagaattaggaagtcagattcttaaagagcatcctgttgaaaatactcaattgcaaaatgaaaagctctatctctattatctccaaaatggaagagacatgtatgtggaccaagaattagatattaatcgtttaagtgattatgatgtcgatgccattgttccacaaagtttccttaaagacgattcaatagacaataaggtcttaacgcgttctgataaaaatcgtggtaaatcggataacgttccaagtgaagaagtagtcaaaaagatgaaaaactattggagacaacttctaaacgccaagttaatcactcaacgtaagtttgataatttaacgaaagctgaacgtggtggtttgagtgaacttgataaagctggttttatcaaacgccaattggttgaaactcgccaaatcactaagcatgtggcacaaattttggatagtcgcatgaatactaaatacgatgaaaatgataaacttattcgagaggttaaagtgattaccttaaaatctaaattagtttctgacttccgaaaagatttccaattctataaagtacgtgagattaacaattaccatcatgcccatgatgcgtatctaaatgccgtcgttggaactgctttgattaagaaatatccaaaacttgaatcggagtttgtctatggtgattataaagtttatgatgttcgtaaaatgattgctaagtctgagcaagaaataggcaaagcaaccgcaaaatatttcttttactctaatatcatgaacttcttcaaaacagaaattacacttgcaaatggagagattcgcaaacgccctctaatcgaaactaatggggaaactggagaaattgtctgggataaagggcgagattttgccacagtgcgcaaagtattgtccatgccccaagtcaatattgtcaagaaaacagaagtacagacaggcgaattctccaaggagtcaattttaccaaaaagaaattcggacaagcttattgctcgtaaaaaagactgggatccaaaaaaatatggtggttttgatagtccaacggtagcttattcagtcctagtggttgctaaggtggaaaaagggaaatcgaagaagttaaaatccgttaaagagttactagggatcacaattatggaaagaagttcctttgaaaaaaatccgattgactttttagaagctaaaggatataaggaagttaaaaaagacttaatcattaaactacctaaatatagtctttttgagttagaaaacggtcgtaaacggatgctggctagtgccggagaattacaaaaaggaaatgagctggctctgccaagcaaatatgtgaattttttatatttagctagtcattatgaaaagttgaagggtagtccagaagataacgaacaaaaacaattgtttgtggagcagcataagcattatttagatgagattattgagcaaatcagtgaattttctaagcgtgttattttagcagatgccaatttagataaagttcttagtgcatataacaaacatagagacaaaccaatacgtgaacaagcagaaaatattattcatttatttacgttgacgaatcttggagctcccgctgcttttaaatattttgatacaacaattgatcgtaaacgatatacgtctacaaaagaagttttagatgccactcttatccatcaatccatcactggtctttatgaaacacgcattgatttgagtcagctaggaggtgactgattctttgtacCCGAtaattgcctatcttccagtgatggaacagcatttgtgcattggctgcaacaatcagccttacttgtgcctgttctatttccgaaccgaccgcttgtatgaatGcatcaaaattcgttttctctaCgttggattccttgttgctcatattgtgatgataatttctacaaatatagtcattggtaactatctatgaaactgtttgatacttttatcagtctaatagttttacaaggtctttcttcatttcttcgtcaatatccctgtatcgtctgaaagctttgcttccctccttgtgtcccgacagtgcggaaacaaggttcgggtctttcacttttttatagatattgccgataaacgtacgtcttgccagatggctgcttgccacttcataaataggtcgtttgatttcgttgtgcgtcaacgggtctaagattgttacgatgcggtcaactccagctaatttgaatatcttttttatggcatcattgtacttttgctcggatatgaacggcaacagttttccctcatattctttgtagcgttcaaggatttctttcgctttgtcgttaagtggaacacgtaccgtaaccggattcccctctttggttttcttgggaatatattctatggcttcattgaccacatttagtttggtcattcggtacaggtcgctcaccctgcatcctatcagtgtctgaaatatgaatatatccctctgtattgccagttgtggggtggcagaaaggtctgcattaaaaatcctgtccctttcttcgagtgttatataataaggtgtaccatatgtacactcctctatcggaaacttgtcgaaaggtctgtttgtggtgcgtttgttatcgaagcaccacaggaagaatgtgcgtattcttgaaaaacagtctatcagcgtgtttttgcttctgggctgtggtgtcctcttttcgggaatggcttcataaatgctcgggtaaagttcataatactggtattcgttctgaaagaaatcccacatatcccgaagcgtgtcaggtgttaccaaatccacatcaaggataaagcccttttgtcctctctttgtagcccttacatatagttcataacgcagtaaggctcttttgacaacccggaaatttttctttcgtacttccgacaaagggtgcttgtttagaaattcatcgaatagttctccaatggtaggcttgataaccacttcctccggcaaaaaatatttttcaggatggtaaaatttatcaagtgttgtttttagccattctttgtctattgcttccttttcttgttgatataccttctcgatataggttttcaactggcgtatctcttcatttatatgggtacgcatttcttcattgcagacagctttcgtttttacacattcgtctttatcatcccatagattgggattgatggctaactgagtaggagcaacggagtctaactgccttccgtttctaaaacggacatagattgtagccatggattctgtatcatatcgtttggctgcttttttaatgataaaggttactttcatagactttcaggttgaattttactctgctgcaaatataaatattttccccagcattttccccacatctgctaaatattttgcaattcgattaaacttggattaaaatttaataggattataacatattgaaatacagtgtaattgtggcgtttttccgcatttttctttttacccacattttccccactttaattatttcaaatgcggggtctgggtacaagaaagaaagctaagtatttgatagttcaatacttagctttttcttttgcttgaattttccccacattttccccacacgtgcaaaaaatatagcagtaagtcattatttcttttggttgaacgtagagagtagcgatattaaaagaatccgatgagaaaagactaatatttatctatccattcagtttgatttttcaggactttacatcgtcctgaaagtatttgttggtaccggtaccgaggacgcgtaaacatttacaGTTGCATGTGGCCTATTGTTTaggacgcgttatctccttaacgtacgttttcgttccattggccctcaaaccccgttatatacattcatgtccatttatgtaaaaaatcctgctgaccttgtttatgtcttgtcagtcaccatttgcaaaaccatatttgaccctcaaagaggctgaatttgataagcaacttgctacatactcataataaggagctaaatagaacacgaatgggaaatactcaaatgccaaactaaagaagatattggccaaaataaacgctataccgagagagaaacttgatttttcaacttcctaaccaacagtgttgttcaaacatttctacttatttgtacttaccagttgaacctacgtttccctaataaaatgtctatggtaaaaagttaaaaaatcctcctacttttgttagatatatttttttgtgtaattttgtaatcgttatgcggcagtaataatatacatattaatacgagttaggaatcctgtagttctcatatgctacgaggaggtattaaaaggtgcgtttcgacaatgcatctattgtagtatattattgcttaatccaaatgaatattataaatttaggaattcttgctcacattgatgcaggaaaaacttccgtaaccgagaatctgctgtttgccagtggagcaacggaaaagtgcggctgtgtggataatggtgacaccataacggactctatggatatagagaaacgtagaggaattactgttcgggcttctacgacatctattatctggaatggtgtgaaatgcaatatcattgacactccgggacacatggattttattgcggaagtggagcggacattcaaaatgcttgatggagcagtcctcatcttatccgcaaaggaaggcatacaagcgcagacaaagttgctgttcaatactttacagaagctgcaaatcccgacaattatatttatcaataagattgaccgagccggtgtgaatttggagcgtttgtatctggatataaaagcaaatctgtctcaagatgtcctgtttatgcaaaatgttgtcgatggatcggtttatccggtttgctcccaaacatatataaaggaagaatacaaagaatttgtatgcaaccatgacgacaatatattagaacgatatttggcggatagcgaaatttcaccggctgattattggaatacgataatcgctcttgtggcaaaagccaaagtctatccggtgctacatggatcagcaatgttcaatatcggtatcaatgagttgttggacgccatcacttcttttatacttcctccggcatcggtctcaaacagactttcatcttatctttataagatagagcatgaccccaaaggacataaaagaagttttctaaaaataattgacggaagtctgagacttcgagacgttgtaagaatcaacgattcggaaaaattcatcaagattaaaaatctaaaaactatcaatcagggcagagagataaatgttgatgaagtgggcgccaatgatatcgcgattgtagaggatatggatgattttcgaatcggaaattatttaggtgctgaaccttgtttgattcaaggattatcgcatcagcatcccgctctcaaatcctccgtccggccagacaggcccgaagagagaagcaaggtgatatccgctctgaatacattgtggattgaagacccgtctttgtccttttccataaactcatatagtgatgaattggaaatctcgttatatggtttaacccaaaaggaaatcatacagacattgctggaagaacgattttccgtaaaggtccattttgatgagatcaagactatatacaaagaacgacctgtaaaaaaggtcaataagattattcagatcgaagtgccgcccaacccttattgggccacaatagggctgactcttgaacccttaccgttagggacagggttgcaaatcgaaagtgacatctcctatggttatctgaaccattcttttcaaaatgccgtttttgaagggattcgtatgtcttgccaatccgggttacatggatgggaagtgactgatctgaaagtaacttttactcaagccgagtattatagcccggtaagtacacctgctgatttcagacagctgaccccttatgtcttcaggctggccttgcaacagtcaggtgtggacattctcgaaccgatgctctattttgagttgcagataccccaagcggcaagttccaaagctattacagatttgcaaaaaatgatgtctgagattgaagacatcagttgcaataatgagtggtgtcatattaaagggaaagttccattaaatacaagtaaagactatgcatcagaagtaagttcatacactaagggcttaggcatttttatggttaagccatgcgggtatcaaataacaaaaggcggttattctgataatatccgcatgaacgaaaaagataaacttttattcatgttccaaaaatcaatgtcatcaaaataatggtataacctctccttaatttattgcatctcttttcgaatatttatgttttttgagaaaagaacgtactcatggttcatcccgatatgcgtatcggtctgtatatcagcaactttctatgtgtttcaactacaatagtcatctattctcatctttctgagtccaccccctgcaaagcccctctttacgacataaaaattcggtcggaaaaggtatgcaaaagatgtttctctctttaagagaaactcttcgggatgcaaaaatatgaaaataactccaattcaccaaattatatagcgacttttttacaaaatgctaaaatttgttgatttccgtcaagcaattgttgagcaaaaatgtcttttacgataaaatgatacctcaatatcaactgtttagcaaaacgatatttctcttaaagagagaaacacctttttgttcaccaatccccgacttttaatcccgcggccatgattgaaaaaggaagagtatgagtattcaacatttccgtgtcgcccttattcccttttttgcggcattttgccttcctgtttttgctcacccagaaacgctggtgaaagtaaaagatgctgaagatcagttgggtgcacgagtgggttacatcgaactggatctcaacagcggtaagatccttgagagttttcgccccgaagaacgttttccaatgatgagcacttttaaagttctgctatgtggcgcggtattatcccgtattgacgccgggcaagagcaactcggtcgccgcatacactattctcagaatgacttggttgagtactcaccagtcacagaaaagcatcttacggatggcatgacagtaagagaattatgcagtgctgccataaccatgagtgataacactgcggccaacttacttctgacaacgatcggaggaccgaaggagctaaccgcttttttgcacaacatgggggatcatgtaactcgccttgatcgttgggaaccggagctgaatgaagccataccaaacgacgagcgtgacaccacgatgcctgtagcaatggcaacaacgttgcgcaaactattaactggcgaactacttactctagcttcccggcaacaattaatagactggatggaggcggataaagttgcaggaccacttctgcgctcggcccttccggctggctggtttattgctgataaatctggagccggtgagcgtgggtcacgcggtatcattgcagcactggggccagatggtaagccctcccgtatcgtagttatctacacgacggggagtcaggcaactatggatgaacgaaatagacagatcgctgagataggtgcctcactgattaagcattggtaactgtcagaccaagtttactcataacgcgtcaattcgagggggatcaattccgtgataggtgggctgcccttcctggttggcttggtttcatcagccatccgcttgccctcatctgttacgccggcggtagccggccagcctcgcagagcaggattcccgttgagcaccgccaggtgcgaataagggacagtgaagaaggaacacccgctcgcgggtgggcctacttcacctatcctgcccggctgacgccgttggatacaccaaggaaagtctacacgaaccctttggcaaaatcctgtatatcgtgcgaaaaaggatggatataccgaaaaaatcgctataatgaccccgaagcagggttatgcagcggaaaacggaattgatccggccacgatgcgtccggcgtagaggatctgaagatcagcagttcaacctgttgatagtacgtactaagctctcatgtttcacgtactaagctctcatgtttaacgtactaagctctcatgtttaacgaactaaaccctcatggctaacgtactaagctctcatggctaacgtactaagctctcatgtttcacgtactaagctctcatgtttgaacaataaaattaatataaatcagcaacttaaatagcctctaaggttttaagttttataagaaaaaaaagaatatataaggcttttaaagcttttaaggtttaacggttgtggacaacaagccagg

>pBH506

gatgtaacgcactgagaagcccttagagcctctcaaagcaattttgagtgacacaggaacacttaacggctgacatgggaattcccctccaccgcggtgtattaatgcggctgcCCCTgtacaaagaaaattcgacaaactgttatttttctatctatttatttgaattgtgagcggataacaattacctttgtcggcaattgtgagcggataacaattATGAgtcaggCTGATGAGTCCGTGAGGACGAAACGAGTAAGCTCGTCcctgacatcacattaccagtGTTTTAGAGCTAGAAATAGCAAGTTAAAATAAGGCTAGTCCGTTATCAACTTGAAAAAGTGGCACCGAGTCGGTGCTTTTttGGCCGGCATGGTCCCAGCCTCCTCGCTGGCGCCGGCTGGGCAACATGCTTCGGCATGGCGAATGGGACgcactctaatcgttatcggagtgcttttagattactaatcaaattgcttctactaattgcctatcttccagtgatggaacagcatttgAACGcattggctgcaacaatcagccttgatctggaagaagcaatgaaagctgctgttaagtctccgaatcaggtattgttcctgacaggtgtattcccatccggtaaacgcggatactttgcagttgatctgactcaggaataaattataaattaaggtaagaagattgtaggataagctaatgaaatagaaaaaggatgccgtcacacaacttgtcggcattcttttttgttttattagttgaaaatatagtgaaaaagttgcctaaatatgtatgttaacaaattatttgtcgtaactttgcactccaaatctgtttttaacatatggcactagtgAAACCAGTAACGTTATACGATGTCGCAGAGTATGCCGGTGTCTCTTATCAGACCGTTTCCCGCGTGGTGAACCAGGCCAGCCACGTTTCTGCGAAAACGCGGGAAAAAGTGGAAGCGGCGATGGCGGAGCTGAATTACATTCCCAACCGCGTGGCACAACAACTGGCGGGCAAACAGTCGTTGCTGATTGGCGTTGCCACCTCCAGTCTGGCCCTGCACGCGCCGTCGCAAATTGTCGCGGCGATTAAATCTCGCGCCGATCAACTGGGTGCCAGCGTGGTGGTGTCGATGGTAGAACGAAGCGGCGTCGAAGCCTGTAAAACGGCGGTGCACAATCTTCTCGCGCAACGCGTCAGTGGGCTGATCATTAACTATCCGCTGGATGACCAGGATGCCATTGCTGTGGAAGCTGCCTGCACTAATGTTCCGGCGTTATTTCTTGATGTCTCTGACCAGACACCCATCAACAGTATTATTTTCTCCCATGAAGACGGTACGCGACTGGGCGTGGAGCATCTGGTCGCATTGGGTCACCAGCAAATCGCGCTGTTAGCGGGCCCATTAAGTTCTGTCTCGGCGCGTCTGCGTCTGGCTGGCTGGCATAAATATCTCACTCGCAATCAAATTCAGCCGATAGCGGAACGGGAAGGCGACTGGAGTGCCATGTCCGGTTTTCAACAAACCATGCAAATGCTGAATGAGGGCATCGTTCCCACTGCGATGCTGGTTGCCAACGATCAGATGGCGCTGGGCGCAATGCGCGCCATTACCGAGTCCGGGCTGCGCGTTGGTGCGGATATCTCGGTAGTGGGATACGACGATACCGAAGACAGCTCATGTTATATCCCGCCGTTAACCACCATCAAACAGGATTTTCGCCTGCTGGGGCAAACCAGCGTGGACCGCTTGCTGCAACTCTCTCAGGGCCAGGCGGTGAAGGGCAATCAGCTGTTGCCCGTCTCACTGGTGAAAAGAAAAACCACCCTGGCGCCCAATACGCAAACCGCCTCTCCCCGCGCGTTGGCCGATTCATTAATGCAGCTGGCACGACAGGTTTCCCGACTGGAAAGCGGGCAGtgagctttcctcggtaccaaattccagaaaagaggcctcccgaaaggggggccttttttcgttttggtcctacttgtgcctgttctatttccTATGcggttcctggccttttgctggccttttgctcacatgttctttcctgcgttatcccctgattctgtggataaccgtattaccgcctttgagtgagctgatacatcccaaatgctgctcggtaccaaagacgaaCaataagacgctgaaaagcgtcttttttcgttttggtccgataaagtttggaagataaagctaaaagttcttatctttgcagtccgaaataaagacatataaaagaaaagacaccatggataagaaatactcaataggcttagctatcggcacaaatagcgtcggatgggcggtgatcactgatgaatataaggttccgtctaaaaagttcaaggttctgggaaatacagaccgccacagtatcaaaaaaaatcttataggggctcttttatttgacagtggagagacagcggaagcgactcgtctcaaacggacagctcgtagaaggtatacacgtcggaagaatcgtatttgttatctacaggagattttttcaaatgagatggcgaaagtagatgatagtttctttcatcgacttgaagagtcttttttggtggaagaagacaagaagcatgaacgtcatcctatttttggaaatatagtagatgaagttgcttatcatgagaaatatccaactatctatcatctgcgaaaaaaattggtagattctactgataaagcggatttgcgcttaatctatttggccttagcgcatatgattaagtttcgtggtcattttttgattgagggagatttaaatcctgataatagtgatgtggacaaactatttatccagttggtacaaacctacaatcaattatttgaagaaaaccctattaacgcaagtggagtagatgctaaagcgattctttctgcacgattgagtaaatcaagacgattagaaaatctcattgctcagctccccggtgagaagaaaaatggcttatttgggaatctcattgctttgtcattgggtttgacccctaattttaaatcaaattttgatttggcagaagatgctaaattacagctttcaaaagatacttacgatgatgatttagataatttattggcgcaaattggagatcaatatgctgatttgtttttggcagctaagaatttatcagatgctattttactttcagatatcctaagagtaaatactgaaataactaaggctcccctatcagcttcaatgattaaacgctacgatgaacatcatcaagacttgactcttttaaaagctttagttcgacaacaacttccagaaaagtataaagaaatcttttttgatcaatcaaaaaacggatatgcaggttatattgatgggggagctagccaagaagaattttataaatttatcaaaccaattttagaaaaaatggatggtactgaggaattattggtgaaactaaatcgtgaagatttgctgcgcaagcaacggacctttgacaacggctctattccccatcaaattcacttgggtgagctgcatgctattttgagaagacaagaagacttttatccatttttaaaagacaatcgtgagaagattgaaaaaatcttgacttttcgaattccttattatgttggtccattggcgcgtggcaatagtcgttttgcatggatgactcggaagtctgaagaaacaattaccccatggaattttgaagaagttgtcgataaaggtgcttcagctcaatcatttattgaacgcatgacaaactttgataaaaatcttccaaatgaaaaagtactaccaaaacatagtttgctttatgagtattttacggtttataacgaattgacaaaggtcaaatatgttactgaaggaatgcgaaaaccagcatttctttcaggtgaacagaagaaagccattgttgatttactcttcaaaacaaatcgaaaagtaaccgttaagcaattaaaagaagattatttcaaaaaaatagaatgttttgatagtgttgaaatttcaggagttgaagatagatttaatgcttcattaggtacctaccatgatttgctaaaaattattaaagataaagattttttggataatgaagaaaatgaagatatcttagaggatattgttttaacattgaccttatttgaagatagggagatgattgaggaaagacttaaaacatatgctcacctctttgatgataaggtgatgaaacagcttaaacgtcgccgttatactggttggggacgtttgtctcgaaaattgattaatggtattagggataagcaatctggcaaaacaatattagattttttgaaatcagatggttttgccaatcgcaattttatgcagctgatccatgatgatagtttgacatttaaagaagacattcaaaaagcacaagtgtctggacaaggcgatagtttacatgaacatattgcaaatttagctggtagccctgctattaaaaaaggtattttacagactgtaaaagttgttgatgaattggtcaaagtaatggggcggcataagccagaaaatatcgttattgaaatggcacgtgaaaatcagacaactcaaaagggccagaaaaattcgcgagagcgtatgaaacgaatcgaagaaggtatcaaagaattaggaagtcagattcttaaagagcatcctgttgaaaatactcaattgcaaaatgaaaagctctatctctattatctccaaaatggaagagacatgtatgtggaccaagaattagatattaatcgtttaagtgattatgatgtcgatgccattgttccacaaagtttccttaaagacgattcaatagacaataaggtcttaacgcgttctgataaaaatcgtggtaaatcggataacgttccaagtgaagaagtagtcaaaaagatgaaaaactattggagacaacttctaaacgccaagttaatcactcaacgtaagtttgataatttaacgaaagctgaacgtggtggtttgagtgaacttgataaagctggttttatcaaacgccaattggttgaaactcgccaaatcactaagcatgtggcacaaattttggatagtcgcatgaatactaaatacgatgaaaatgataaacttattcgagaggttaaagtgattaccttaaaatctaaattagtttctgacttccgaaaagatttccaattctataaagtacgtgagattaacaattaccatcatgcccatgatgcgtatctaaatgccgtcgttggaactgctttgattaagaaatatccaaaacttgaatcggagtttgtctatggtgattataaagtttatgatgttcgtaaaatgattgctaagtctgagcaagaaataggcaaagcaaccgcaaaatatttcttttactctaatatcatgaacttcttcaaaacagaaattacacttgcaaatggagagattcgcaaacgccctctaatcgaaactaatggggaaactggagaaattgtctgggataaagggcgagattttgccacagtgcgcaaagtattgtccatgccccaagtcaatattgtcaagaaaacagaagtacagacaggcgaattctccaaggagtcaattttaccaaaaagaaattcggacaagcttattgctcgtaaaaaagactgggatccaaaaaaatatggtggttttgatagtccaacggtagcttattcagtcctagtggttgctaaggtggaaaaagggaaatcgaagaagttaaaatccgttaaagagttactagggatcacaattatggaaagaagttcctttgaaaaaaatccgattgactttttagaagctaaaggatataaggaagttaaaaaagacttaatcattaaactacctaaatatagtctttttgagttagaaaacggtcgtaaacggatgctggctagtgccggagaattacaaaaaggaaatgagctggctctgccaagcaaatatgtgaattttttatatttagctagtcattatgaaaagttgaagggtagtccagaagataacgaacaaaaacaattgtttgtggagcagcataagcattatttagatgagattattgagcaaatcagtgaattttctaagcgtgttattttagcagatgccaatttagataaagttcttagtgcatataacaaacatagagacaaaccaatacgtgaacaagcagaaaatattattcatttatttacgttgacgaatcttggagctcccgctgcttttaaatattttgatacaacaattgatcgtaaacgatatacgtctacaaaagaagttttagatgccactcttatccatcaatccatcactggtctttatgaaacacgcattgatttgagtcagctaggaggtgactgattctttgtacCCGAtaattgcctatcttccagtgatggaacagcatttgtgcattggctgcaacaatcagccttacttgtgcctgttctatttccgaaccgaccgcttgtatgaatGcatcaaaattcgttttctctaCgttggattccttgttgctcatattgtgatgataatttctacaaatatagtcattggtaactatctatgaaactgtttgatacttttatcagtctaatagttttacaaggtctttcttcatttcttcgtcaatatccctgtatcgtctgaaagctttgcttccctccttgtgtcccgacagtgcggaaacaaggttcgggtctttcacttttttatagatattgccgataaacgtacgtcttgccagatggctgcttgccacttcataaataggtcgtttgatttcgttgtgcgtcaacgggtctaagattgttacgatgcggtcaactccagctaatttgaatatcttttttatggcatcattgtacttttgctcggatatgaacggcaacagttttccctcatattctttgtagcgttcaaggatttctttcgctttgtcgttaagtggaacacgtaccgtaaccggattcccctctttggttttcttgggaatatattctatggcttcattgaccacatttagtttggtcattcggtacaggtcgctcaccctgcatcctatcagtgtctgaaatatgaatatatccctctgtattgccagttgtggggtggcagaaaggtctgcattaaaaatcctgtccctttcttcgagtgttatataataaggtgtaccatatgtacactcctctatcggaaacttgtcgaaaggtctgtttgtggtgcgtttgttatcgaagcaccacaggaagaatgtgcgtattcttgaaaaacagtctatcagcgtgtttttgcttctgggctgtggtgtcctcttttcgggaatggcttcataaatgctcgggtaaagttcataatactggtattcgttctgaaagaaatcccacatatcccgaagcgtgtcaggtgttaccaaatccacatcaaggataaagcccttttgtcctctctttgtagcccttacatatagttcataacgcagtaaggctcttttgacaacccggaaatttttctttcgtacttccgacaaagggtgcttgtttagaaattcatcgaatagttctccaatggtaggcttgataaccacttcctccggcaaaaaatatttttcaggatggtaaaatttatcaagtgttgtttttagccattctttgtctattgcttccttttcttgttgatataccttctcgatataggttttcaactggcgtatctcttcatttatatgggtacgcatttcttcattgcagacagctttcgtttttacacattcgtctttatcatcccatagattgggattgatggctaactgagtaggagcaacggagtctaactgccttccgtttctaaaacggacatagattgtagccatggattctgtatcatatcgtttggctgcttttttaatgataaaggttactttcatagactttcaggttgaattttactctgctgcaaatataaatattttccccagcattttccccacatctgctaaatattttgcaattcgattaaacttggattaaaatttaataggattataacatattgaaatacagtgtaattgtggcgtttttccgcatttttctttttacccacattttccccactttaattatttcaaatgcggggtctgggtacaagaaagaaagctaagtatttgatagttcaatacttagctttttcttttgcttgaattttccccacattttccccacacgtgcaaaaaatatagcagtaagtcattatttcttttggttgaacgtagagagtagcgatattaaaagaatccgatgagaaaagactaatatttatctatccattcagtttgatttttcaggactttacatcgtcctgaaagtatttgttggtaccggtaccgaggacgcgtaaacatttacaGTTGCATGTGGCCTATTGTTTaggacgcgttatctccttaacgtacgttttcgttccattggccctcaaaccccgttatatacattcatgtccatttatgtaaaaaatcctgctgaccttgtttatgtcttgtcagtcaccatttgcaaaaccatatttgaccctcaaagaggctgaatttgataagcaacttgctacatactcataataaggagctaaatagaacacgaatgggaaatactcaaatgccaaactaaagaagatattggccaaaataaacgctataccgagagagaaacttgatttttcaacttcctaaccaacagtgttgttcaaacatttctacttatttgtacttaccagttgaacctacgtttccctaataaaatgtctatggtaaaaagttaaaaaatcctcctacttttgttagatatatttttttgtgtaattttgtaatcgttatgcggcagtaataatatacatattaatacgagttaggaatcctgtagttctcatatgctacgaggaggtattaaaaggtgcgtttcgacaatgcatctattgtagtatattattgcttaatccaaatgaatattataaatttaggaattcttgctcacattgatgcaggaaaaacttccgtaaccgagaatctgctgtttgccagtggagcaacggaaaagtgcggctgtgtggataatggtgacaccataacggactctatggatatagagaaacgtagaggaattactgttcgggcttctacgacatctattatctggaatggtgtgaaatgcaatatcattgacactccgggacacatggattttattgcggaagtggagcggacattcaaaatgcttgatggagcagtcctcatcttatccgcaaaggaaggcatacaagcgcagacaaagttgctgttcaatactttacagaagctgcaaatcccgacaattatatttatcaataagattgaccgagccggtgtgaatttggagcgtttgtatctggatataaaagcaaatctgtctcaagatgtcctgtttatgcaaaatgttgtcgatggatcggtttatccggtttgctcccaaacatatataaaggaagaatacaaagaatttgtatgcaaccatgacgacaatatattagaacgatatttggcggatagcgaaatttcaccggctgattattggaatacgataatcgctcttgtggcaaaagccaaagtctatccggtgctacatggatcagcaatgttcaatatcggtatcaatgagttgttggacgccatcacttcttttatacttcctccggcatcggtctcaaacagactttcatcttatctttataagatagagcatgaccccaaaggacataaaagaagttttctaaaaataattgacggaagtctgagacttcgagacgttgtaagaatcaacgattcggaaaaattcatcaagattaaaaatctaaaaactatcaatcagggcagagagataaatgttgatgaagtgggcgccaatgatatcgcgattgtagaggatatggatgattttcgaatcggaaattatttaggtgctgaaccttgtttgattcaaggattatcgcatcagcatcccgctctcaaatcctccgtccggccagacaggcccgaagagagaagcaaggtgatatccgctctgaatacattgtggattgaagacccgtctttgtccttttccataaactcatatagtgatgaattggaaatctcgttatatggtttaacccaaaaggaaatcatacagacattgctggaagaacgattttccgtaaaggtccattttgatgagatcaagactatatacaaagaacgacctgtaaaaaaggtcaataagattattcagatcgaagtgccgcccaacccttattgggccacaatagggctgactcttgaacccttaccgttagggacagggttgcaaatcgaaagtgacatctcctatggttatctgaaccattcttttcaaaatgccgtttttgaagggattcgtatgtcttgccaatccgggttacatggatgggaagtgactgatctgaaagtaacttttactcaagccgagtattatagcccggtaagtacacctgctgatttcagacagctgaccccttatgtcttcaggctggccttgcaacagtcaggtgtggacattctcgaaccgatgctctattttgagttgcagataccccaagcggcaagttccaaagctattacagatttgcaaaaaatgatgtctgagattgaagacatcagttgcaataatgagtggtgtcatattaaagggaaagttccattaaatacaagtaaagactatgcatcagaagtaagttcatacactaagggcttaggcatttttatggttaagccatgcgggtatcaaataacaaaaggcggttattctgataatatccgcatgaacgaaaaagataaacttttattcatgttccaaaaatcaatgtcatcaaaataatggtataacctctccttaatttattgcatctcttttcgaatatttatgttttttgagaaaagaacgtactcatggttcatcccgatatgcgtatcggtctgtatatcagcaactttctatgtgtttcaactacaatagtcatctattctcatctttctgagtccaccccctgcaaagcccctctttacgacataaaaattcggtcggaaaaggtatgcaaaagatgtttctctctttaagagaaactcttcgggatgcaaaaatatgaaaataactccaattcaccaaattatatagcgacttttttacaaaatgctaaaatttgttgatttccgtcaagcaattgttgagcaaaaatgtcttttacgataaaatgatacctcaatatcaactgtttagcaaaacgatatttctcttaaagagagaaacacctttttgttcaccaatccccgacttttaatcccgcggccatgattgaaaaaggaagagtatgagtattcaacatttccgtgtcgcccttattcccttttttgcggcattttgccttcctgtttttgctcacccagaaacgctggtgaaagtaaaagatgctgaagatcagttgggtgcacgagtgggttacatcgaactggatctcaacagcggtaagatccttgagagttttcgccccgaagaacgttttccaatgatgagcacttttaaagttctgctatgtggcgcggtattatcccgtattgacgccgggcaagagcaactcggtcgccgcatacactattctcagaatgacttggttgagtactcaccagtcacagaaaagcatcttacggatggcatgacagtaagagaattatgcagtgctgccataaccatgagtgataacactgcggccaacttacttctgacaacgatcggaggaccgaaggagctaaccgcttttttgcacaacatgggggatcatgtaactcgccttgatcgttgggaaccggagctgaatgaagccataccaaacgacgagcgtgacaccacgatgcctgtagcaatggcaacaacgttgcgcaaactattaactggcgaactacttactctagcttcccggcaacaattaatagactggatggaggcggataaagttgcaggaccacttctgcgctcggcccttccggctggctggtttattgctgataaatctggagccggtgagcgtgggtcacgcggtatcattgcagcactggggccagatggtaagccctcccgtatcgtagttatctacacgacggggagtcaggcaactatggatgaacgaaatagacagatcgctgagataggtgcctcactgattaagcattggtaactgtcagaccaagtttactcataacgcgtcaattcgagggggatcaattccgtgataggtgggctgcccttcctggttggcttggtttcatcagccatccgcttgccctcatctgttacgccggcggtagccggccagcctcgcagagcaggattcccgttgagcaccgccaggtgcgaataagggacagtgaagaaggaacacccgctcgcgggtgggcctacttcacctatcctgcccggctgacgccgttggatacaccaaggaaagtctacacgaaccctttggcaaaatcctgtatatcgtgcgaaaaaggatggatataccgaaaaaatcgctataatgaccccgaagcagggttatgcagcggaaaacggaattgatccggccacgatgcgtccggcgtagaggatctgaagatcagcagttcaacctgttgatagtacgtactaagctctcatgtttcacgtactaagctctcatgtttaacgtactaagctctcatgtttaacgaactaaaccctcatggctaacgtactaagctctcatggctaacgtactaagctctcatgtttcacgtactaagctctcatgtttgaacaataaaattaatataaatcagcaacttaaatagcctctaaggttttaagttttataagaaaaaaaagaatatataaggcttttaaagcttttaaggtttaacggttgtggacaacaagccagg

>pBH507

gatgtaacgcactgagaagcccttagagcctctcaaagcaattttgagtgacacaggaacacttaacggctgacatgggaattcccctccaccgcggtgtattaatgcggctgcCCCTgtacaaagaaaattcgacaaactgttatttttctatctatttatttgaattgtgagcggataacaattacctttgtcggcaattgtgagcggataacaattATGAgtcaggCTGATGAGTCCGTGAGGACGAAACGAGTAAGCTCGTCcctgacatcacattaccagtGTTTTAGAGCTAGAAATAGCAAGTTAAAATAAGGCTAGTCCGTTATCAACTTGAAAAAGTGGCACCGAGTCGGTGCTTTTttGGCCGGCATGGTCCCAGCCTCCTCGCTGGCGCCGGCTGGGCAACATGCTTCGGCATGGCGAATGGGACgcactctaatcgttatcggagtgcttttagattactaatcaaattgcttctactaattgcctatcttccagtgatggaacagcatttgAACGcattggctgcaacaatcagccttgatctggaagaagcaatgaaagctgctgttaagtctccgaatcaggtattgttcctgacaggtgtattcccatccggtaaacgcggatactttgcagttgatctgactcaggaataaattataaattaaggtaagaagattgtaggataagctaatgaaatagaaaaaggatgccgtcacacaacttgtcggcattcttttttgttttattagttgaaaatatagtgaaaaagttgcctaaatatgtatgttaacaaattatttgtcgtaactttgcactccaaatctgtttttaacatatggcactagtgAAACCAGTAACGTTATACGATGTCGCAGAGTATGCCGGTGTCTCTTATCAGACCGTTTCCCGCGTGGTGAACCAGGCCAGCCACGTTTCTGCGAAAACGCGGGAAAAAGTGGAAGCGGCGATGGCGGAGCTCAATTACATTCCCAACCGCGTGGCACAACAACTGGCGGGCAAAGCGTCGCATACCATTGGCATGTTGATCACTGCCAGTACCAATCCTTTCTATTCAGAACTGGTGCGTGGCGTTGAACGCAGCTGCTTCGAACGCGGTTATAGTCTCGTCCTTTGCAATACCGAAGGCGATGAACAGCGGATGAATCGCAATCTGGAAACGCTGATGCAAAAACGCGTTGATGGCTTGCTGTTACTGTGCACCGAAACGCATCAACCTTCGCGTGAAATCATGCAACGTTATCCGACAGTGCCTACTGTGATGATGGACTGGGCTCCGTTCGATGGCGACAGCGATCTTATTCAGGATAACTCGTTGCTGGGCGGAGACTTAGCAACGCAATATCTGATCGATAAAGGTCATACCCGTATCGCCTGTATTACCGGCCCGCTGGATAAAACTCCGGCGCGCCTGCGGTTGGAAGGTTATCGGGCGGCGATGAAACGTGCGGGTCTCAACATTCCTGATGGCTATGAAGTCACTGGTGATTTTGAATTTAACGGCGGGTTTGACGCTATGCGCCAACTGCTATCACATCCGCTGCGTCCTCAGGCCGTCTTTACCGGAAATGACGCTATGGCTGTTGGCGTTTACCAGGCGTTATATCAGGCAGAGTTACAGGTTCCGCAGGATATCGCGGTGATTGGCTATGACGATATCGAACTGGCAAGCTTTATGACGCCACCATTAACCACTATCCACCAACCGAAAGATGAACTGGGGGAGCTGGCGATTGATGTACTCATCCATCGGATAACCCAGCCGACCCTTCAGCAACAACGATTACAACTTACTCCGATTCTGATGGAACGCGGTTCGGCTTAGCTGGTGAAAAGAAAAACCACCCTGGCGCCCAATACGCAAACCGCCTCTCCCCGCGCGTTGGCCGATTCATTAATGCAGCTGGCACGACAGGTTTCCCGACTGGAAAGCGGGCAGTGAgctttcctcggtaccaaattccagaaaagaggcctcccgaaaggggggccttttttcgttttggtcctacttgtgcctgttctatttccTATGcggttcctggccttttgctggccttttgctcacatgttctttcctgcgttatcccctgattctgtggataaccgtattaccgcctttgagtgagctgatacatcccaaatgctgctcggtaccaaagacgaaCaataagacgctgaaaagcgtcttttttcgttttggtccgataaagtttggaagataaagctaaaagttcttatctttgcagtccgaaataaagacatataaaagaaaagacaccatggataagaaatactcaataggcttagctatcggcacaaatagcgtcggatgggcggtgatcactgatgaatataaggttccgtctaaaaagttcaaggttctgggaaatacagaccgccacagtatcaaaaaaaatcttataggggctcttttatttgacagtggagagacagcggaagcgactcgtctcaaacggacagctcgtagaaggtatacacgtcggaagaatcgtatttgttatctacaggagattttttcaaatgagatggcgaaagtagatgatagtttctttcatcgacttgaagagtcttttttggtggaagaagacaagaagcatgaacgtcatcctatttttggaaatatagtagatgaagttgcttatcatgagaaatatccaactatctatcatctgcgaaaaaaattggtagattctactgataaagcggatttgcgcttaatctatttggccttagcgcatatgattaagtttcgtggtcattttttgattgagggagatttaaatcctgataatagtgatgtggacaaactatttatccagttggtacaaacctacaatcaattatttgaagaaaaccctattaacgcaagtggagtagatgctaaagcgattctttctgcacgattgagtaaatcaagacgattagaaaatctcattgctcagctccccggtgagaagaaaaatggcttatttgggaatctcattgctttgtcattgggtttgacccctaattttaaatcaaattttgatttggcagaagatgctaaattacagctttcaaaagatacttacgatgatgatttagataatttattggcgcaaattggagatcaatatgctgatttgtttttggcagctaagaatttatcagatgctattttactttcagatatcctaagagtaaatactgaaataactaaggctcccctatcagcttcaatgattaaacgctacgatgaacatcatcaagacttgactcttttaaaagctttagttcgacaacaacttccagaaaagtataaagaaatcttttttgatcaatcaaaaaacggatatgcaggttatattgatgggggagctagccaagaagaattttataaatttatcaaaccaattttagaaaaaatggatggtactgaggaattattggtgaaactaaatcgtgaagatttgctgcgcaagcaacggacctttgacaacggctctattccccatcaaattcacttgggtgagctgcatgctattttgagaagacaagaagacttttatccatttttaaaagacaatcgtgagaagattgaaaaaatcttgacttttcgaattccttattatgttggtccattggcgcgtggcaatagtcgttttgcatggatgactcggaagtctgaagaaacaattaccccatggaattttgaagaagttgtcgataaaggtgcttcagctcaatcatttattgaacgcatgacaaactttgataaaaatcttccaaatgaaaaagtactaccaaaacatagtttgctttatgagtattttacggtttataacgaattgacaaaggtcaaatatgttactgaaggaatgcgaaaaccagcatttctttcaggtgaacagaagaaagccattgttgatttactcttcaaaacaaatcgaaaagtaaccgttaagcaattaaaagaagattatttcaaaaaaatagaatgttttgatagtgttgaaatttcaggagttgaagatagatttaatgcttcattaggtacctaccatgatttgctaaaaattattaaagataaagattttttggataatgaagaaaatgaagatatcttagaggatattgttttaacattgaccttatttgaagatagggagatgattgaggaaagacttaaaacatatgctcacctctttgatgataaggtgatgaaacagcttaaacgtcgccgttatactggttggggacgtttgtctcgaaaattgattaatggtattagggataagcaatctggcaaaacaatattagattttttgaaatcagatggttttgccaatcgcaattttatgcagctgatccatgatgatagtttgacatttaaagaagacattcaaaaagcacaagtgtctggacaaggcgatagtttacatgaacatattgcaaatttagctggtagccctgctattaaaaaaggtattttacagactgtaaaagttgttgatgaattggtcaaagtaatggggcggcataagccagaaaatatcgttattgaaatggcacgtgaaaatcagacaactcaaaagggccagaaaaattcgcgagagcgtatgaaacgaatcgaagaaggtatcaaagaattaggaagtcagattcttaaagagcatcctgttgaaaatactcaattgcaaaatgaaaagctctatctctattatctccaaaatggaagagacatgtatgtggaccaagaattagatattaatcgtttaagtgattatgatgtcgatgccattgttccacaaagtttccttaaagacgattcaatagacaataaggtcttaacgcgttctgataaaaatcgtggtaaatcggataacgttccaagtgaagaagtagtcaaaaagatgaaaaactattggagacaacttctaaacgccaagttaatcactcaacgtaagtttgataatttaacgaaagctgaacgtggtggtttgagtgaacttgataaagctggttttatcaaacgccaattggttgaaactcgccaaatcactaagcatgtggcacaaattttggatagtcgcatgaatactaaatacgatgaaaatgataaacttattcgagaggttaaagtgattaccttaaaatctaaattagtttctgacttccgaaaagatttccaattctataaagtacgtgagattaacaattaccatcatgcccatgatgcgtatctaaatgccgtcgttggaactgctttgattaagaaatatccaaaacttgaatcggagtttgtctatggtgattataaagtttatgatgttcgtaaaatgattgctaagtctgagcaagaaataggcaaagcaaccgcaaaatatttcttttactctaatatcatgaacttcttcaaaacagaaattacacttgcaaatggagagattcgcaaacgccctctaatcgaaactaatggggaaactggagaaattgtctgggataaagggcgagattttgccacagtgcgcaaagtattgtccatgccccaagtcaatattgtcaagaaaacagaagtacagacaggcgaattctccaaggagtcaattttaccaaaaagaaattcggacaagcttattgctcgtaaaaaagactgggatccaaaaaaatatggtggttttgatagtccaacggtagcttattcagtcctagtggttgctaaggtggaaaaagggaaatcgaagaagttaaaatccgttaaagagttactagggatcacaattatggaaagaagttcctttgaaaaaaatccgattgactttttagaagctaaaggatataaggaagttaaaaaagacttaatcattaaactacctaaatatagtctttttgagttagaaaacggtcgtaaacggatgctggctagtgccggagaattacaaaaaggaaatgagctggctctgccaagcaaatatgtgaattttttatatttagctagtcattatgaaaagttgaagggtagtccagaagataacgaacaaaaacaattgtttgtggagcagcataagcattatttagatgagattattgagcaaatcagtgaattttctaagcgtgttattttagcagatgccaatttagataaagttcttagtgcatataacaaacatagagacaaaccaatacgtgaacaagcagaaaatattattcatttatttacgttgacgaatcttggagctcccgctgcttttaaatattttgatacaacaattgatcgtaaacgatatacgtctacaaaagaagttttagatgccactcttatccatcaatccatcactggtctttatgaaacacgcattgatttgagtcagctaggaggtgactgattctttgtacCCGAtaattgcctatcttccagtgatggaacagcatttgtgcattggctgcaacaatcagccttacttgtgcctgttctatttccgaaccgaccgcttgtatgaatGcatcaaaattcgttttctctaCgttggattccttgttgctcatattgtgatgataatttctacaaatatagtcattggtaactatctatgaaactgtttgatacttttatcagtctaatagttttacaaggtctttcttcatttcttcgtcaatatccctgtatcgtctgaaagctttgcttccctccttgtgtcccgacagtgcggaaacaaggttcgggtctttcacttttttatagatattgccgataaacgtacgtcttgccagatggctgcttgccacttcataaataggtcgtttgatttcgttgtgcgtcaacgggtctaagattgttacgatgcggtcaactccagctaatttgaatatcttttttatggcatcattgtacttttgctcggatatgaacggcaacagttttccctcatattctttgtagcgttcaaggatttctttcgctttgtcgttaagtggaacacgtaccgtaaccggattcccctctttggttttcttgggaatatattctatggcttcattgaccacatttagtttggtcattcggtacaggtcgctcaccctgcatcctatcagtgtctgaaatatgaatatatccctctgtattgccagttgtggggtggcagaaaggtctgcattaaaaatcctgtccctttcttcgagtgttatataataaggtgtaccatatgtacactcctctatcggaaacttgtcgaaaggtctgtttgtggtgcgtttgttatcgaagcaccacaggaagaatgtgcgtattcttgaaaaacagtctatcagcgtgtttttgcttctgggctgtggtgtcctcttttcgggaatggcttcataaatgctcgggtaaagttcataatactggtattcgttctgaaagaaatcccacatatcccgaagcgtgtcaggtgttaccaaatccacatcaaggataaagcccttttgtcctctctttgtagcccttacatatagttcataacgcagtaaggctcttttgacaacccggaaatttttctttcgtacttccgacaaagggtgcttgtttagaaattcatcgaatagttctccaatggtaggcttgataaccacttcctccggcaaaaaatatttttcaggatggtaaaatttatcaagtgttgtttttagccattctttgtctattgcttccttttcttgttgatataccttctcgatataggttttcaactggcgtatctcttcatttatatgggtacgcatttcttcattgcagacagctttcgtttttacacattcgtctttatcatcccatagattgggattgatggctaactgagtaggagcaacggagtctaactgccttccgtttctaaaacggacatagattgtagccatggattctgtatcatatcgtttggctgcttttttaatgataaaggttactttcatagactttcaggttgaattttactctgctgcaaatataaatattttccccagcattttccccacatctgctaaatattttgcaattcgattaaacttggattaaaatttaataggattataacatattgaaatacagtgtaattgtggcgtttttccgcatttttctttttacccacattttccccactttaattatttcaaatgcggggtctgggtacaagaaagaaagctaagtatttgatagttcaatacttagctttttcttttgcttgaattttccccacattttccccacacgtgcaaaaaatatagcagtaagtcattatttcttttggttgaacgtagagagtagcgatattaaaagaatccgatgagaaaagactaatatttatctatccattcagtttgatttttcaggactttacatcgtcctgaaagtatttgttggtaccggtaccgaggacgcgtaaacatttacaGTTGCATGTGGCCTATTGTTTaggacgcgttatctccttaacgtacgttttcgttccattggccctcaaaccccgttatatacattcatgtccatttatgtaaaaaatcctgctgaccttgtttatgtcttgtcagtcaccatttgcaaaaccatatttgaccctcaaagaggctgaatttgataagcaacttgctacatactcataataaggagctaaatagaacacgaatgggaaatactcaaatgccaaactaaagaagatattggccaaaataaacgctataccgagagagaaacttgatttttcaacttcctaaccaacagtgttgttcaaacatttctacttatttgtacttaccagttgaacctacgtttccctaataaaatgtctatggtaaaaagttaaaaaatcctcctacttttgttagatatatttttttgtgtaattttgtaatcgttatgcggcagtaataatatacatattaatacgagttaggaatcctgtagttctcatatgctacgaggaggtattaaaaggtgcgtttcgacaatgcatctattgtagtatattattgcttaatccaaatgaatattataaatttaggaattcttgctcacattgatgcaggaaaaacttccgtaaccgagaatctgctgtttgccagtggagcaacggaaaagtgcggctgtgtggataatggtgacaccataacggactctatggatatagagaaacgtagaggaattactgttcgggcttctacgacatctattatctggaatggtgtgaaatgcaatatcattgacactccgggacacatggattttattgcggaagtggagcggacattcaaaatgcttgatggagcagtcctcatcttatccgcaaaggaaggcatacaagcgcagacaaagttgctgttcaatactttacagaagctgcaaatcccgacaattatatttatcaataagattgaccgagccggtgtgaatttggagcgtttgtatctggatataaaagcaaatctgtctcaagatgtcctgtttatgcaaaatgttgtcgatggatcggtttatccggtttgctcccaaacatatataaaggaagaatacaaagaatttgtatgcaaccatgacgacaatatattagaacgatatttggcggatagcgaaatttcaccggctgattattggaatacgataatcgctcttgtggcaaaagccaaagtctatccggtgctacatggatcagcaatgttcaatatcggtatcaatgagttgttggacgccatcacttcttttatacttcctccggcatcggtctcaaacagactttcatcttatctttataagatagagcatgaccccaaaggacataaaagaagttttctaaaaataattgacggaagtctgagacttcgagacgttgtaagaatcaacgattcggaaaaattcatcaagattaaaaatctaaaaactatcaatcagggcagagagataaatgttgatgaagtgggcgccaatgatatcgcgattgtagaggatatggatgattttcgaatcggaaattatttaggtgctgaaccttgtttgattcaaggattatcgcatcagcatcccgctctcaaatcctccgtccggccagacaggcccgaagagagaagcaaggtgatatccgctctgaatacattgtggattgaagacccgtctttgtccttttccataaactcatatagtgatgaattggaaatctcgttatatggtttaacccaaaaggaaatcatacagacattgctggaagaacgattttccgtaaaggtccattttgatgagatcaagactatatacaaagaacgacctgtaaaaaaggtcaataagattattcagatcgaagtgccgcccaacccttattgggccacaatagggctgactcttgaacccttaccgttagggacagggttgcaaatcgaaagtgacatctcctatggttatctgaaccattcttttcaaaatgccgtttttgaagggattcgtatgtcttgccaatccgggttacatggatgggaagtgactgatctgaaagtaacttttactcaagccgagtattatagcccggtaagtacacctgctgatttcagacagctgaccccttatgtcttcaggctggccttgcaacagtcaggtgtggacattctcgaaccgatgctctattttgagttgcagataccccaagcggcaagttccaaagctattacagatttgcaaaaaatgatgtctgagattgaagacatcagttgcaataatgagtggtgtcatattaaagggaaagttccattaaatacaagtaaagactatgcatcagaagtaagttcatacactaagggcttaggcatttttatggttaagccatgcgggtatcaaataacaaaaggcggttattctgataatatccgcatgaacgaaaaagataaacttttattcatgttccaaaaatcaatgtcatcaaaataatggtataacctctccttaatttattgcatctcttttcgaatatttatgttttttgagaaaagaacgtactcatggttcatcccgatatgcgtatcggtctgtatatcagcaactttctatgtgtttcaactacaatagtcatctattctcatctttctgagtccaccccctgcaaagcccctctttacgacataaaaattcggtcggaaaaggtatgcaaaagatgtttctctctttaagagaaactcttcgggatgcaaaaatatgaaaataactccaattcaccaaattatatagcgacttttttacaaaatgctaaaatttgttgatttccgtcaagcaattgttgagcaaaaatgtcttttacgataaaatgatacctcaatatcaactgtttagcaaaacgatatttctcttaaagagagaaacacctttttgttcaccaatccccgacttttaatcccgcggccatgattgaaaaaggaagagtatgagtattcaacatttccgtgtcgcccttattcccttttttgcggcattttgccttcctgtttttgctcacccagaaacgctggtgaaagtaaaagatgctgaagatcagttgggtgcacgagtgggttacatcgaactggatctcaacagcggtaagatccttgagagttttcgccccgaagaacgttttccaatgatgagcacttttaaagttctgctatgtggcgcggtattatcccgtattgacgccgggcaagagcaactcggtcgccgcatacactattctcagaatgacttggttgagtactcaccagtcacagaaaagcatcttacggatggcatgacagtaagagaattatgcagtgctgccataaccatgagtgataacactgcggccaacttacttctgacaacgatcggaggaccgaaggagctaaccgcttttttgcacaacatgggggatcatgtaactcgccttgatcgttgggaaccggagctgaatgaagccataccaaacgacgagcgtgacaccacgatgcctgtagcaatggcaacaacgttgcgcaaactattaactggcgaactacttactctagcttcccggcaacaattaatagactggatggaggcggataaagttgcaggaccacttctgcgctcggcccttccggctggctggtttattgctgataaatctggagccggtgagcgtgggtcacgcggtatcattgcagcactggggccagatggtaagccctcccgtatcgtagttatctacacgacggggagtcaggcaactatggatgaacgaaatagacagatcgctgagataggtgcctcactgattaagcattggtaactgtcagaccaagtttactcataacgcgtcaattcgagggggatcaattccgtgataggtgggctgcccttcctggttggcttggtttcatcagccatccgcttgccctcatctgttacgccggcggtagccggccagcctcgcagagcaggattcccgttgagcaccgccaggtgcgaataagggacagtgaagaaggaacacccgctcgcgggtgggcctacttcacctatcctgcccggctgacgccgttggatacaccaaggaaagtctacacgaaccctttggcaaaatcctgtatatcgtgcgaaaaaggatggatataccgaaaaaatcgctataatgaccccgaagcagggttatgcagcggaaaacggaattgatccggccacgatgcgtccggcgtagaggatctgaagatcagcagttcaacctgttgatagtacgtactaagctctcatgtttcacgtactaagctctcatgtttaacgtactaagctctcatgtttaacgaactaaaccctcatggctaacgtactaagctctcatggctaacgtactaagctctcatgtttcacgtactaagctctcatgtttgaacaataaaattaatataaatcagcaacttaaatagcctctaaggttttaagttttataagaaaaaaaagaatatataaggcttttaaagcttttaaggtttaacggttgtggacaacaagccagg

>pBH508
[truncated: 79,519 more chars]
